# Latent Class Log‐Linear Models for Estimating Diagnostic Test Accuracy Without a Gold Standard: A Simulation Study

**DOI:** 10.1002/sim.70660

**Published:** 2026-07-06

**Authors:** Yasin Okkaoglu, Nicky J. Welton, A. E. Ades, Hayley E. Jones

**Affiliations:** ^1^ Population Health Sciences, Bristol Medical School University of Bristol Bristol UK

**Keywords:** Bayesian, conditional dependence, diagnostic accuracy, latent class, log‐linear, shrinkage priors

## Abstract

**Background:**

In the absence of a gold standard, latent class models can be used to estimate test accuracy from a study comparing results on multiple tests. Fixed‐effect and latent trait models are often used to account for conditional dependencies between tests, mitigating against biased accuracy estimates, but are difficult to implement. Latent class log‐linear models are an under‐evaluated alternative.

**Objectives:**

We evaluate the performance of Bayesian two‐class log‐linear models in estimating sensitivity, specificity, and prevalence under a variety of real‐world conditional dependence structures, and the use of shrinkage priors on interaction terms when dependence structures are unknown.

**Methods:**

Data were simulated from (i) latent trait, (ii) fixed‐effect, and (iii) log‐linear models, with dependence structures motivated by four real data sets and three sample sizes. We fitted conditional independence models, log‐linear models incorporating the “correct” pairwise interactions, and log‐linear models incorporating all interactions with shrinkage priors. We report bias, coverage, residual deviance, and DIC.

**Results:**

Log‐linear models incorporating the correct pairwise dependencies exhibited promising but variable performance (likely due to insufficient sample sizes) across data‐generating mechanisms. Improvements over conditional independence models were substantial. Shrinkage priors achieved reasonable performance when dependencies existed within a single disease class but showed poor convergence under complex dependence structures.

**Conclusions:**

Latent class log‐linear models offer a relatively robust alternative for estimating accuracy when the conditional dependence structure is known. When this is not the case, shrinkage priors show promise when dependencies are only within one disease state, but there are challenges related to convergence.

## 1 Introduction

Latent class models estimate diagnostic test accuracy when no gold standard is available, by modeling the overlap in individuals' results across multiple tests [1]. Early studies assumed that correlations between test results were solely due to the true disease status (conditional independence, CInd) [1, 2, 3], whereas more recent studies have shown that ignoring potential correlations between tests within the same disease state (conditional dependence, CDep) can bias estimates of test accuracy and disease prevalence [4, 5, 6, 7, 8].

To address this, several CDep models have been proposed, with fixed‐effect [4, 5, 6, 9, 10, 11, 12] and latent trait models [13] being the most widely used. Fixed‐effect models account for correlations by adding dependence terms to the joint probabilities of test combinations, while latent trait models use one or more random effects to represent individual‐level factors influencing test results. Various formulations of fixed‐effect models have been developed [4, 5, 6, 9, 10, 11, 12]. Since dependence terms are additive on the probability scale, constraints are needed to ensure probabilities of observing each combination of test results within each disease state are all estimated between zero and one. These constraints depend on the number of tests and the dependence structure [5], and closed‐form expressions are rarely available except for two‐test scenarios [9]. Constraints can be implemented using computational “tricks”, which involve excluding posterior samples associated with invalid probabilities, but this may substantially influence the implied prior distributions [14].

Implementation of latent trait models can also be challenging. These are typically fitted to individual‐level data using Bayesian Markov Chain Monte Carlo (MCMC) methods [6, 10, 15, 16], and run times can be very long, especially with many tests or large sample sizes on a standard computer. Further, the most widely used latent trait model [13] may be limited in specifying certain correlation structures. For instance, a 4‐test scenario where Tests 1 and 2 are correlated, and Tests 3 and 4 are also correlated but independent of the first pair cannot be specified using a single random effect. This limitation may be addressed by incorporating multiple random effects for different test pairs [17, 18], but this increases model complexity and computation time. In our experience fitting latent trait models via Bayesian MCMC, high autocorrelation in the chains is common, requiring a large number of iterations to achieve sufficiently low Monte Carlo error—often computationally impractical. To improve identifiability and convergence, a shared coefficient for the random effect has been widely adopted, reducing the number of parameters to estimate [10, 15, 16, 19, 20] but further limiting flexibility to model different correlation structures.

Latent class log‐linear (L‐L) models offer an alternative approach to account for CDep by incorporating interaction terms between test pairs within disease state [21, 22, 23, 24, 25]. L‐L models naturally produce valid probabilities between 0 and 1 without requiring constraints and are computationally efficient, as they are fitted to aggregated data (counts of test result patterns, in a contingency table). L‐L models are widely recognized as a standard method for analyzing contingency tables. Despite these advantages, there has been little use of, or investigation into the performance of, latent class L‐L models in this context. Xu et al. [26] conducted a simulation study using data generated from a Probit Latent Class model (see Xu and Craig [18] for the model definition), and fitted CInd, L‐L, and correct models. Both the L‐L and correct models produced similar biases, though lower than those from the CInd model. However, this evaluation was limited to a single data‐generating mechanism and parameter setting, and assumed the dependence structure was known a priori.

Mis‐specification of CDep model type and structure can lead to biased accuracy estimates [19]. However, information on the dependence structure among diagnostic tests within each disease state is often unavailable and inferring it from statistical indicators can be problematic. Subtil et al. [27] and Okkaoglu et al. [8] showed that commonly used methods based on CInd model misfit—such as residual correlation plots and chi‐squared statistics—are unreliable for identifying the CDep structure. Hanson et al. [24] employed a forward and backward selection strategy to identify associations between tests, but this approach can be time‐consuming, particularly with a large number of tests.

Simultaneously including all possible pairwise interactions may be infeasible due to non‐identifiability, overfitting, and increased computational burden [28, 29]. Penalized Bayesian regression methods using shrinkage priors have been proposed to address overfitting and non‐identifiability in other settings when the number of potential covariates is high [30, 31]. These priors shrink small coefficients toward zero [29, 30, 31, 32, 33, 34]. Use of these shrinkage priors in latent class diagnostic accuracy models appears to be unexplored.

This simulation study evaluates the performance of two‐latent‐class (diseased, disease‐free) Bayesian L‐L models with pairwise interactions in estimating diagnostic test accuracy (sensitivities and specificities) under various data generating mechanisms, CDep structures and parameter settings, reflecting real‐world scenarios. We additionally investigate the use of shrinkage priors to account for CDep without a priori specification of which tests are dependent. Although there are various parameterisations of L‐L models in the literature [21, 25], we use the parameterisation proposed by Espeland and Handelmann [21] throughout this study.

## 2 Latent Class Log‐Linear Models

Assume that binary results from multiple diagnostic tests are available on a number of individuals, n, each with latent disease state, D=0 (disease‐free) or D=1 (diseased). These results can be summarized in contingency tables showing the number of subjects with each specific test results pattern. These observed counts have a multinomial likelihood: $X=\left(X_{1},X_{2},\ldots ,X_{K}\right)∼\text{Multinomial}\;\left(n,(p_{1},p_{2},\ldots p_{K}\right))$, where J denotes the number of tests and $K=2^{J}$ is the number of possible response patterns.

The probabilities can be modeled with a latent class model with general form as follows, where $T_{j}$ (j=1,2,…,J) represent the result of the j th diagnostic test, with $T_{j}=1$ indicating a positive result and $T_{j}=0$ a negative result.

(1) $$\begin{array}{cc} & P\left(T_{1}=t_{1},T_{2}=t_{2},\ldots ,T_{J}=t_{J}\right)=P\left(T_{1}=t_{1},T_{2}=t_{2},\ldots ,T_{J}=t_{J}|D=1\right)\\ & \;P\left(D=1\right)+P\left(T_{1}=t_{1},T_{2}=t_{2},\ldots ,T_{J}=t_{J}|D=0\right)P\left(D=0\right), \end{array}$$

where P(D=1) is the prevalence (π) of the disease.

When the diagnostic tests under evaluation are conditionally independent, Equation (1) can be written as [1].

(2) $$\begin{array}{cc} & P\left(T_{1}=t_{1},T_{2}=t_{2},\ldots ,T_{J}=t_{J}\right)=\pi \prod_{j=1}^{J}{\mathrm{Se}}_{j}^{t_{j}}{\left(1-{\mathrm{Se}}_{j}\right)}^{1-t_{j}}+\left(1-\pi \right)\\ & \;\prod_{j=1}^{J}{\mathrm{Sp}}_{j}^{1-t_{j}}{\left(1-{\mathrm{Sp}}_{j}\right)}^{t_{j}}, \end{array}$$

where ${\mathrm{Se}}_{j}$ and ${\mathrm{Sp}}_{j}$ denote the sensitivity and specificity of the j th test. Equation (1) can also equivalently be written as:

$$p_{k}=\pi p_{k|d=1}+\left(1-\pi \right)p_{k|d=0},\;k=1,2,\ldots ,K$$

where $p_{k}$ is the marginal probability of each test pattern and $p_{k|d}$ is the conditional probability within disease state d (d=1 for the diseased state and d=0 for the disease‐free state).

In latent class log‐linear models, the multinomial likelihood is re‐written as $X_{k}∼\text{Poisson}\left(np_{k}\right)$, k=1,2,…,K. Let $y_{\mathrm{jk}}$ be an indicator variable denoting the j th test's result in row k. Then the CInd model (Equation 2) can alternatively be specified as an L‐L model incorporating only main effects [21] as follows:

$$\log\left(\mu_{k|d}\right)=\sum_{j=1}^{J}\lambda_{j|d}y_{\mathrm{jk}}\;$$

(3) $$p_{k|d}=\frac{\mu_{k|d}}{\sum_{k=1}^{K}\mu_{k|d}},$$

where $\lambda_{j|d}$ is the main effect of test j and $\mu_{k|d}$ is the unnormalised rate associated with response pattern k, within disease state d.

Sensitivities and specificities can then be obtained as the inverse logit transformations of the main effects from the model (Equation 3) as follows:

$${\mathrm{Se}}_{j}=\frac{e^{\lambda_{j|d=1}}}{1+e^{\lambda_{j|d=1}}}\;$$

$${\mathrm{Sp}}_{j}=1-\left(\frac{e^{\lambda_{j|d=0}}}{1+e^{\lambda_{j|d=0}}}\right).$$

The CInd assumption in Equation (3) can be relaxed by adding pairwise interaction terms [21, 22, 23, 25, 26, 35]. The general form including all possible pairwise interactions is as follows:

(4) $$\log\left(\mu_{k|d}\right)=\sum_{j=1}^{J}\lambda_{j|d}y_{\mathrm{jk}}+\sum_{j<l}\lambda_{\mathrm{jl}|d}y_{\mathrm{jk}}y_{\mathrm{lk}}$$

where the pairwise interaction term $\lambda_{\mathrm{jl}|d}$, j,l=1,2,…,J;j<l, is the log odds ratio representing agreement between the j th and l th tests within disease state d [22]. Note that main effects in the CDep model (Equation 4) can no longer be interpreted in terms of sensitivities and specificities [25]. Instead, sensitivities and specificities are now complex functions of main effects and the interaction terms that can be expressed as the sum of conditional probabilities

$${\mathrm{Se}}_{j}=\sum_{k:y_{\mathrm{jk}}=1}p_{k|d=1}\;$$

$${\mathrm{Sp}}_{j}=1-\sum_{k:y_{\mathrm{jk}}=1}p_{k|d=0}$$

Since there is no obvious evidence in the literature supporting the benefit of using higher‐order interaction terms in modeling the CDep between diagnostic tests and parameter identifiability is a challenge in latent class models, only L‐L models with pairwise interactions are considered in this paper.

## 3 Shrinkage Priors

Since researchers may not know the CDep structure for the tests in their study, as part of this simulation study we explored the performance of fitting latent class L‐L models incorporating all pairwise interactions, using shrinkage priors to account for an unknown CDep structure. Three shrinkage priors are evaluated: (i) hyperlasso [36], (ii) regularized horseshoe [37], and (iii) elastic net [38] (see van Erp et al. [29] for their characteristics). Prior specifications and hyperparameter settings are detailed in Appendix A in Supporting Information.

Simulation studies evaluating shrinkage priors applied to covariates have typically assessed both the predictive performance and variable selection ability of models [29, 30, 32, 34]. Various strategies have been used to identify relevant covariates, with a common approach being examination of whether the 95% credible interval (CrI) for each coefficient includes zero [30, 32]. Li and Pati [31] noted that this strategy can be problematic in high‐dimensional settings, as sparse data may lead to high uncertainty around parameter estimates. Moreover, refitting models after excluding covariates with posterior distributions centered near zero is not recommended, as such covariates may still hold relevance [30].

## 4 Simulation Study

The simulation study is described in the following sub‐sections following the ADEMP (Aims, Data‐generating mechanisms, Estimands, Methods, Performance measures) framework [39].

### 4.1 Aims

The simulation study has two objectives:

- To evaluate the performance of latent class L‐L models in estimating diagnostic test accuracy and disease prevalence under various data‐generating mechanisms (DGMs), with differing CDep structures.
- To assess whether including all pairwise interactions with shrinkage priors can avoid the need for a priori knowledge of test dependencies.

### 4.2 Data‐Generating Mechanisms (DGMs)

We evaluated the performance of latent class L‐L models under scenarios where the true DGM was latent trait, fixed‐effect, or L‐L. Given the wide range of possible DGMs and parameter values, we based our choices on prior analyses of published data sets to reflect real‐world conditions.

We selected three published data sets with different levels of complexity of CDep structure and for which at least two types of CDep latent class models (fixed‐effect, latent trait, or L‐L) had been previously applied. These data sets described results on four tests for HIV [40] and visceral leishmaniasis (VL) [41] and five tests for childhood pulmonary tuberculosis (CPTB) [42, 43].

Of the three, the HIV data set features the simplest assumed correlation structure, with CDep assumed only between a single pair of tests. The HIV data set consists of three antigen‐coated radioimmunoassays (ag121, p24 and gp120) for detecting anti‐HIV antibodies, together with an enzyme‐linked immunosorbent assay (ELISA). Previous analyses of this data set [13, 23, 28] assumed CDep between the p24 and gp120 assays (Tests 2 and 3), based on statistical model‐diagnostic procedures rather than underlying biological considerations. This dependence may be biologically plausible: p24 and gp120 are major structural HIV proteins that elicit closely related antibody responses, whereas the ag121 recombinant assay captures a broader antibody spectrum and the ELISA, as a whole‐virus assay, provides an even more composite signal [44, 45, 46]. These features together make dependence between the p24 and gp120 assays a plausible pairing from both statistical and biological perspectives.

The VL data set includes two antibody tests (Test 1: DAT and Test 2: rk39), an antigen test (Test 3: KAtex), and a parasitology test (Test 4). We follow a previous analysis of this data [10] in assuming CDep between Tests 1 and 2 (since both are influenced by the host's antibody load) and between Tests 3 and 4 (as both are more likely to be positive when circulating parasite levels are high) in the diseased state only.

The CPTB data set comprises three microbiological tests (Test 1: culture, Test 2: Xpert, and Test 3: microscopy), a skin test (Test 4), and radiography (Test 5). We assumed a CDep structure described by Schumacher et al. [16], involving dependence in the diseased state only. This assumes CDep among the three microbiological tests, on the basis that children with higher bacillary loads are more likely to yield positive results on all three tests. CDep was also assumed between the skin test (Test 4) and the microbiological tests, as it was considered that skin test sensitivity may be reduced in individuals with high bacillary burden. Radiography (Test 5) was assumed to be conditionally independent of all other tests [16].

We additionally explored a more complex scenario with CDep among four of five diagnostic tests in both latent classes. As most empirical studies assume CDep only within the diseased group, we simulated data for this scenario based on a dependence structure considered in a previous simulation study by Keddie et al. [7], referred to as the CDep in diseased positives and negatives (CDPN) setting. Keddie et al. [7] simulated CDPN data from latent trait models (Equation 5) based on a motivating example [47] consisting of four serological assays (Tests 1–4) and culture (Test 5) for diagnosis of Melioidosis. As serological tests all detect antibody responses, CDep among infected individuals is anticipated [47]. Following Keddie et al. [7], we extended this structure by also assuming CDep among serological tests within the disease‐free class to create a more challenging and general simulation scenario.

Table 1 summarizes the CDep structures and the ranges of prevalence, sensitivities, and specificities across different DGMs, along with the primary studies from which the data sets were derived. We simulated CDep data with these characteristics, based on the models fitted in the studies listed in Table 1. We treated the published parameter estimates and dependency structures as the truth. If necessary parameter estimates were not reported, we fitted the models described in these publications to the original data sets to obtain the values required.

**TABLE 1:** Parameter settings and correlated pairs for data‐generating mechanisms.

		Data			
		HIV^c^	VL^c^	CPTB^c^	CDPN
Parameter^a^	π	0.541–0.542	0.370–0.372	0.227–0.292	0.500
	${\mathrm{Se}}_{1}$	0.997	0.849–0.857	0.564–0.689	0.650
	${\mathrm{Se}}_{2}$	0.569–0.573	0.779–0.782	0.461–0.572	0.650
	${\mathrm{Se}}_{3}$	0.908–0.910	0.693–0.730	0.201–0.274	0.650
	${\mathrm{Se}}_{4}$	0.996	0.712–0.750	0.686–0.765	0.650
	${\mathrm{Se}}_{5}$	N/A	N/A	0.646–0.648	0.650
	${\mathrm{Sp}}_{1}$	0.969–0.972	0.977–0.982	0.997–0.999	0.900
	${\mathrm{Sp}}_{2}$	0.961–0.962	0.918	0.988–0.989	0.900
	${\mathrm{Sp}}_{3}$	0.995–0.997	0.981	0.997–1.000	0.900
	${\mathrm{Sp}}_{4}$	0.918–0.925	0.985	0.659–0.678	0.900
	${\mathrm{Sp}}_{5}$	N/A	N/A	0.760–0.797	0.990
Correlated Pairs^b^—Studies proposing the model	LT^d^ Model	Test 2 × Test 3 (D)	All pairs	All pairs excluding Test 5 (D)	All pairs excluding Test 5 (B)
	Study	Qu et al. [13]	Menten et al. [10]	Wang et al. [6]	Keddie et al. [7]
	FE^d^ Model	Test 2 × Test 3 (B)	Test 1 × Test 2 (D) Test 3 × Test 4 (D)	All pairs excluding Test 5 (D)	All pairs excluding Test 5 (B)
	Study	Wang et al. [28]	Menten et al. [10]	Wang et al. [6]	N/A
	L‐L Model	Test 2 × Test 3 (D)	Test 1 × Test 2 (D) Test 3 × Test 4 (D)	All pairs excluding Test 5 (D)	All pairs excluding Test 5 (B)
	Study	Sepulveda et al. [23]	N/A	N/A	N/A
Studies describing the data		Alvord et al. [40]	Boelaert et al. [41]	Nicol et al. [42] Zar et al. [43] Schumacher et al. [16]	Keddie et al. [7]

*Note:* Detailed parameter values, including dependence magnitudes (e.g., covariance terms, interaction parameters, or random effect coefficients (slope parameters)), are provided in Appendix B in Supporting Information.

^a^ Where parameter values varied across the three models (LT, FE, L‐L), ranges are shown. Values for each DGM are shown in Appendix B in Supporting Information.

^b^ D: Dependency within only Diseased state, B: Dependency within Both diseased and disease‐free states.

^c^ For the real‐world data sets, parameter settings may not be identical to those provided by original studies depending on how they were obtained (see Appendix B in Supporting Information).

^d^ LT: Latent trait, FE: Fixed‐effect.

The HIV data set is the only one for which we found applications of all three model types. For the VL and CPTB data sets, we only found applications of latent trait and fixed‐effect models, while for the CDPN data set only latent trait models were used to simulate data by Keddie et al. [7]. For each case where a DGM was not directly available from the literature, we constructed models to simulate data with a structure similar to that of a published DGM. However, it is important to note that the latent trait, fixed‐effect, and L‐L data‐generating mechanisms do not necessarily imply equivalent CDep structures due to inherent differences in how each model accounts for test dependencies. Where fixed‐effect or L‐L models were not available for one of the four data sets, we simulated data using models with similar prevalence and accuracy parameters, along with conceptually similar CDep structures. This is described in brief below; full details are provided in Appendix B in Supporting Information.

#### 4.2.1 Latent Trait DGMs

Let $T_{\mathrm{ij}}$ denote the result of test j, and $d_{i}$ the true disease state, for subject i. A generalised version of the latent trait models from which we simulated data can be given by

$$P\left(T_{\mathrm{ij}}=1|D_{i}=d,Z_{i}=z_{i}\right)=Ф\left(\alpha_{\mathrm{jd}}+\beta_{\mathrm{jd}}z_{i}\right),$$

(5) i=1,2,…,n;j=1,2,…,J;d=0,1,

where $Z_{i}∼N\left(0,1\right)$ is a random effect that represents some subject‐specific underlying characteristic related to the dependent test results, $\alpha_{\mathrm{jd}}$ and $\beta_{\mathrm{jd}}$ are parameters to be estimated, and Ф represents the cumulative distribution function of the N(0,1) distribution. This assumes CDep in disease state d between all tests with non‐zero $\beta_{\mathrm{id}}$.

#### 4.2.2 Fixed‐Effect DGMs

Although fixed‐effect models had been fitted to three of the data sets, studies had used different parameterisations [6, 9, 10], as follows:

##### 4.2.2.1 Parameterisation 1: HIV data set

Let ${\text{covse}}_{\mathrm{jl}}$ and ${\text{covsp}}_{\mathrm{jl}}$ denote the covariance between Tests j and l within the diseased and disease‐free subjects, respectively. The fixed‐effect model fitted to the HIV data used a parameterisation [28]:

(6) $$\begin{array}{cc} & P\left(T_{1}=t_{1},T_{2}=t_{2},T_{3}=t_{3},T_{4}=t_{4}\right)\\ & \;=\pi \left\{\prod_{j=1}^{4}{\mathrm{Se}}_{j}^{t_{j}}{\left(1-{\mathrm{Se}}_{j}\right)}^{1-t_{j}}+{\left(-1\right)}^{t_{2}+t_{3}}{\text{covse}}_{23}\prod_{j\neq 2,3}{\mathrm{Se}}_{j}^{t_{j}}{\left(1-{\mathrm{Se}}_{j}\right)}^{1-t_{j}}\right\}\\ & \;+\left(1-\pi \right)\left\{\prod_{j=1}^{4}{\mathrm{Sp}}_{j}^{1-t_{j}}{\left(1-{\mathrm{Sp}}_{j}\right)}^{t_{j}}+{\left(-1\right)}^{t_{2}+t_{3}}{\text{covsp}}_{23}\prod_{j\neq 2,3}{\mathrm{Sp}}_{j}^{1-t_{j}}{\left(1-{\mathrm{Sp}}_{j}\right)}^{t_{j}}\right\}. \end{array}$$

This implies an isolated dependency structure for Test 2 and Test 3 within both disease states in the HIV data. This type of parameterisation was proposed by Jones et al. [9], and the general form of it can be found in the original study [9].

##### 4.2.2.2 Parameterisation 2: VL data set

Menten et al. [10] fitted the following fixed‐effect model to the VL data set:

(7) $$\begin{array}{cc} & P\left(T_{1}=t_{1},T_{2}=t_{2},T_{3}=t_{3},T_{4}=t_{4}\right)\\ & \;=\pi \left\{\left(\prod_{j=1}^{2}{\mathrm{Se}}_{j}^{t_{j}}{\left(1-{\mathrm{Se}}_{j}\right)}^{1-t_{j}}\right)+{\left(-1\right)}^{t_{1}+t_{2}}{\text{covse}}_{12}\right\}\\ & \;\times \left\{\left(\prod_{j=3}^{4}{\mathrm{Se}}_{j}^{t_{j}}{\left(1-{\mathrm{Se}}_{j}\right)}^{1-t_{j}}\right)+{\left(-1\right)}^{t_{3}+t_{4}}{\text{covse}}_{34}\right\}\\ & \;+\left(1-\pi \right)\left\{\prod_{j=1}^{4}{\mathrm{Sp}}_{j}^{1-t_{j}}{\left(1-{\mathrm{Sp}}_{j}\right)}^{t_{j}}\right\}. \end{array}$$

This implies a disconnected dependency structure for Tests 1 × 2 and Tests 3 × 4, within the diseased state only.

##### 4.2.2.3 Parameterisation 3: CPTB data set

Let $\delta_{t_{1},t_{2},\ldots ,t_{J}|d}$ denote a dependency term for the test result pattern $T_{1}=t_{1},T_{2}=t_{2},\ldots ,T_{J}=t_{J}$ within the disease state d. Wang et al. [6] proposed a different type of fixed‐effect model, and applied it to the CPTB data:

(8) $$\begin{array}{cc} & P\left(T_{1}=t_{1},T_{2}=t_{2},T_{3}=t_{3},T_{4}=t_{4},T_{5}=t_{5}\right)\\ & \;=\pi \left\{\left(\prod_{j=1}^{4}{\mathrm{Se}}_{j}^{t_{j}}{\left(1-{\mathrm{Se}}_{j}\right)}^{1-t_{j}}\right)+\delta_{t_{1}t_{2}t_{3}t_{4}|d=1}\right\}\times {\mathrm{Se}}_{5}^{t_{5}}{\left(1-{\mathrm{Se}}_{5}\right)}^{1-t_{5}}\\ & \;+\left(1-\pi \right)\left\{\prod_{j=1}^{5}{\mathrm{Sp}}_{j}^{1-t_{j}}{\left(1-{\mathrm{Sp}}_{j}\right)}^{t_{j}}\right\} \end{array}$$

Unlike parameterisations 1 and 2, this implies overlapping dependencies for Tests 1, 2, 3, and 4.

For the CDPN data set, for which no fixed‐effect model was described, we simulated data from a generalization of Parameterisation 1 (Equation 6) to incorporate all pairwise covariance terms for Tests 1–4 [9]. The values of the covariance terms were chosen to reproduce a dependency structure similar to that of the latent trait DGM used by Keddie et al. [7].

#### 4.2.3 L‐L DGMs

For the HIV data set, we simulated from an L‐L model (Equation 4) incorporating a pairwise interaction between Tests 2 and 3 in the diseased state only, as described by Sepulveda et al. [23]. For the VL data set, we simulated from a model incorporating pairwise interactions for Tests 1 × 2 and Tests 3 × 4 in the diseased state.

For the CPTB data set, we simulated from an L‐L model incorporating all pairwise interactions between Tests 1–4 in the diseased state only, while for CDPN we simulated from a model incorporating all pairwise interactions between Tests 1–4 in both disease states. Since applications of L‐L models to these two data sets did not appear in the literature, we set the values of main effects and interactions to yield similar sensitivities, specificities and conditional dependencies to those of the fixed‐effect DGMs. For the CDPN data set, we simulated data using a shared interaction term, following a similar strategy to Keddie et al. [7], whose DGM implied a shared polychoric correlation (see Appendix B in Supporting Information for the definition) between correlated test pairs.

Data were simulated from each of the three types of CDep models for each of the four data sets, yielding 12 DGMs. With sample sizes of 500, 2000, and 5000, this resulted in 36 scenarios. Full details of model specifications, parameter settings, and the rationale behind the selections of the parameter values for each DGM are provided in Appendix B in Supporting Information.

### 4.3 Estimands

The estimands of interest were the sensitivity and specificity of each test, and prevalence. Posterior distributions of each parameter were summarized with posterior medians (point estimates) and 95% CrIs. Additionally, for L‐L models incorporating all pairwise interactions with shrinkage priors, we examined CrIs of various lengths (10% to 90% in 10% increments, and 95%) for the interaction terms. Posterior mean residual deviances (ResD) and deviance information criteria (DIC) [48] were also calculated.

### 4.4 Methods

Models were fitted in JAGS (Just Another Gibbs Sampler) via R [49], using the “R2jags” package [50]. For each of the 36 scenarios, the number of simulations ($n_{\mathrm{sim}}$) was set to 1250. Details of how this value was selected are provided in Appendix C in Supporting Information.

We fitted the CInd model using the L‐L parameterisation (Equation 3) and the L‐L model with relevant pairwise interaction terms (Equation 4, with other interaction terms set to zero) to all simulated data sets. When the data‐generating model was L‐L, this meant fitting the true model to the simulated data sets. For data sets simulated from other DGMs, we fitted L‐L models with pairwise interactions between tests that were CDep within the true DGM. These do not necessarily fully represent the true CDep structure, since some DGMs may imply higher‐order interaction terms on a log‐linear scale. Additionally, to reduce the number of parameters to be estimated, we made some assumptions about shared interaction terms across test pairs for the VL, CPTB and CDPN data sets, based on consideration of shared covariance terms in fixed‐effect DGMs or implied polychoric correlations in latent trait models. Full details are provided in Appendix B in Supporting Information.

For each simulated data set, three MCMC chains were run in parallel, with a burn‐in of 10 000 samples followed by 50 000 additional iterations. Models were deemed non‐convergent if any parameter had an $\hat{R}>1.1$ [51] or an effective sample size lower than 400 [8]. For scenarios where fewer than 90% of data sets achieved convergence, the burn‐in and iteration counts were doubled until this threshold was met. Results for data sets where convergence was not met were excluded.

To assess the performance of L‐L models with shrinkage priors, we initially aimed to fit these including all pairwise interaction terms in both disease states, for all settings. However, initial runs indicated this not to be generally feasible, with lack of identifiability and/or very high autocorrelations, producing inadequate effective sample sizes even after 400 000 iterations. We therefore made the pragmatic decision to only fit pairwise interactions within the diseased state (assuming CInd in the disease‐free state) for the HIV, VL, and CPTB data sets, and interactions in both disease classes for the CDPN data set only. Even with this simplification, autocorrelations were high, such that large numbers of iterations were required. Due to this computational burden, L‐L models with shrinkage priors were fitted only to data sets simulated from L‐L models and only to the first 500 (of 1250) data sets for each setting. We set the burn‐in as 40 000 samples, followed by 200 000 iterations. The same convergence criteria were applied as above, but no further analysis was conducted for scenarios with convergence below 90%. Results for data sets where convergence was not met were excluded.

In all models, the following vague priors were specified: logit(Beta(1,1)) priors for main effects and Beta(1,1) for prevalence. To address the label‐switching (mirror) issue, posterior samples where ${\mathrm{Se}}_{i}<1-{\mathrm{Sp}}_{i}$ were discarded for each model [7, 8, 10]. In L‐L models with vague (rather than shrinkage) priors for interaction terms, N(0,1) priors were assigned.

The R scripts used to simulate data from each model type, along with the JAGS scripts for the CInd model, L‐L models with vague priors, and L‐L models with shrinkage priors, are provided in Supporting Information 1 in Supporting Information.

### 4.5 Performance Measures

Biases of point estimates for each parameter were summarized using the mean, 2.5th, and 97.5th percentiles across data sets. We additionally report coverage of 95% CrIs, defined as the proportion of times the true parameter value was contained in the interval. Additionally, model fit was evaluated using ResD and DIC values.

For L‐L models with shrinkage priors, we additionally evaluated their ability to identify ‘correct’ interactions by calculating the correct inclusion rates (CIR) and false inclusion rates (FIR) of the interaction terms across CrIs of different lengths (10%, 20%, …, 90%, and 95%). A pairwise interaction was considered ‘included’ at a given CrI width if the corresponding CrI did not contain zero. This allowed us to assess whether the 95% CrI criterion [30, 32] was sufficiently sensitive and specific in identifying the true correlated pairs, or whether narrower CrIs offered enhanced performance. Following van Erp et al. [29], we applied the distance criterion [52], defined as $\sqrt{{\left(1-\mathrm{CIR}\right)}^{2}+{\mathrm{FIR}}^{2}}$, to identify the optimal CrI width with the smallest distance in each scenario.

### 4.6 Computational Performance

As an additional analysis to assess and compare computational performance across model classes, we used a representative simulated data set generated under the L‐L DGM for the VL setting with a sample size of 2000. For this data set, we fitted four models: (i) an L‐L model incorporating the correct pairwise interaction terms, (ii) an L‐L model including all pairwise interaction terms within the diseased state with RH shrinkage priors, (iii) a fixed‐effect model corresponding to Equation (7) as specified in Menten et al. [10], for which constraints on covariance terms were implemented using the “zeros trick” [53] to ensure valid probability estimates, following the approach described by Okkaoglu [14], and (iv) a latent trait model with shared slope parameters for Tests 1 and 2 and for Tests 3 and 4, consistent with the specification used in the original study [10]. Models were fitted in a Bayesian framework using JAGS via R, with three parallel MCMC chains. For each model, after 1000 burn‐in samples, the number of iterations was increased incrementally (in steps of 1000) until the convergence criteria described above were satisfied. All computations for this comparison were performed on a standard workstation (Intel Core i7‐1185G7, 4 cores, 16 GB RAM), and runtimes required to achieve convergence for each model were recorded.

## 5 Results

All 45 000 fitted CInd models (100%) across the 36 scenarios met the convergence criteria. Of the L‐L models where only relevant pairwise interaction terms were fitted, 98.0% (44 112/45 000) converged. Among the L‐L models incorporating all interaction terms with shrinkage priors only in the diseased state (HIV, VL, and CPTB settings) 99.5% (13 435/13500) achieved convergence. However, for the CDPN setting, in which we attempted to fit all pairwise interaction terms with shrinkage priors in both disease states, overall convergence was much lower, at 78.0% (3511/4500). Convergence was particularly poor at the largest sample size (5000), with between 43.4% and 59.2% of models converging. The number of converged models and corresponding convergence rates are presented in Appendix D: Tables D.1 and D.2 in the Supporting Information.

The following subsections present results for scenarios with a sample size of 2000. For L‐L models with shrinkage priors, only results from models using regularized horseshoe priors are reported, as the alternative priors showed no substantial differences. Complete results for all sample sizes and all shrinkage priors are provided in Appendix D: Tables D.3–D.50 in the Supporting Information.

### 5.1 Bias

Overall, L‐L models incorporating all relevant pairwise interactions, as well as those using shrinkage priors for all pairwise interactions, yielded lower biases than the CInd model. These differences became more pronounced with larger sample sizes, as L‐L models with interactions tended to produce lower biases under those conditions. For parameters exhibiting bias under the CInd model, the corresponding 95% empirical intervals often did not include zero, indicating systematic bias in those estimates. In contrast, for the L‐L models, the 95% empirical intervals for bias generally contained zero (Appendix D in Supporting Information).

**FIGURE 1:**
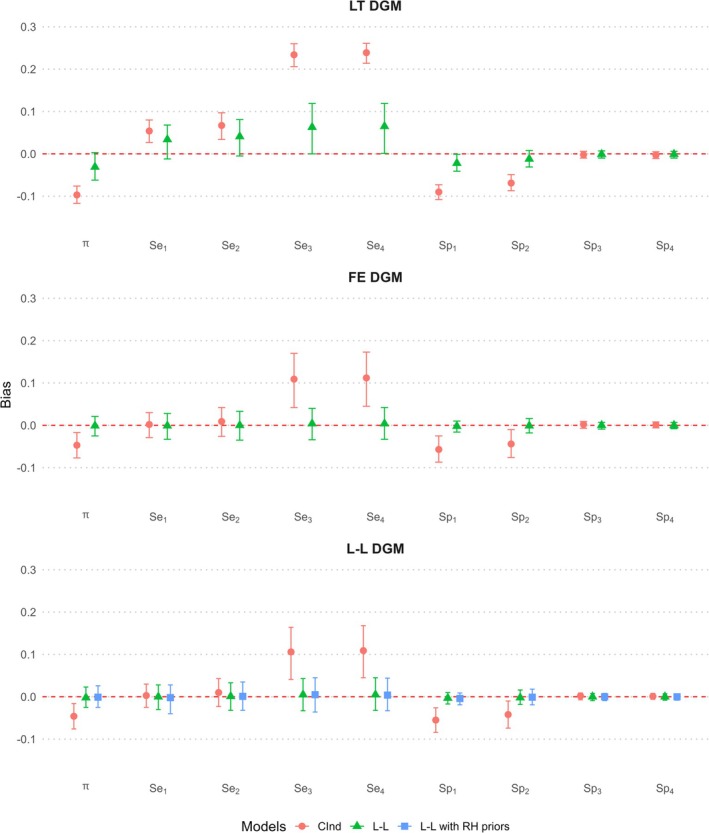
Absolute biases: Means and 2.5th and 97.5th percentiles across models fitted to 1250 (CInd and L‐L with correct pairwise interactions) or 500 (L‐L with regularized horseshoe (RH) priors) data sets simulated based on the VL data set. Sample size = 2000.

**FIGURE 2:**
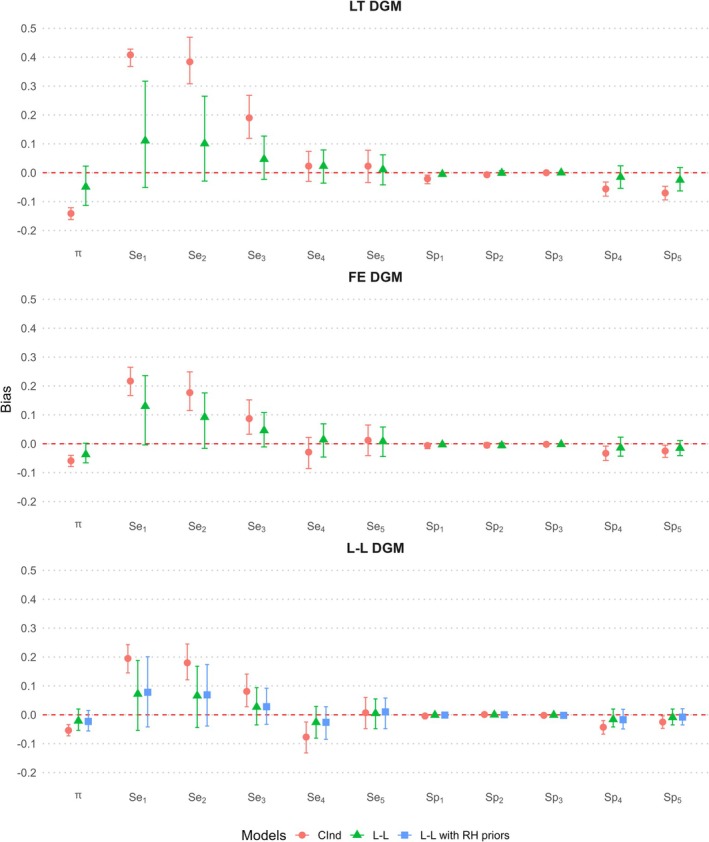
Absolute biases: Means and 2.5th and 97.5th percentiles across models fitted to 1250 (CInd and L‐L with correct pairwise interactions) or 500 (L‐L with regularized horseshoe (RH) priors) data sets simulated based on the CPTB data set. Sample size = 2000.

**FIGURE 3:**
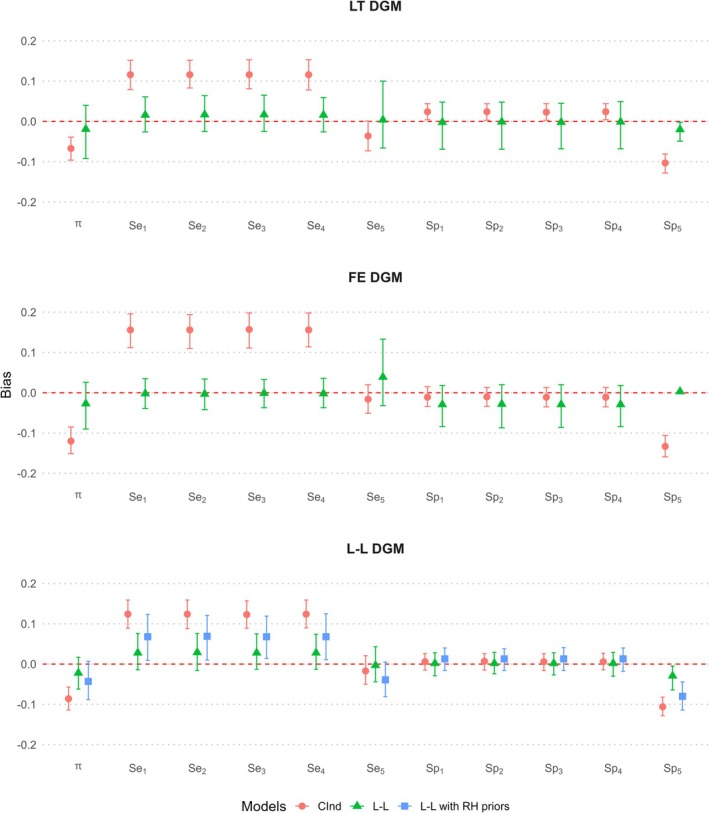
Absolute biases: Means and 2.5th and 97.5th percentiles across models fitted to 1250 (CInd and L‐L with correct pairwise interactions) or 500 (L‐L with regularized horseshoe (RH) priors) data sets simulated based on the CDPN data set. Sample size = 2000.

Figures 1, 2, 3 show absolute biases across simulated data sets based on the VL, CPTB, and CDPN data sets for a sample size of 2000. Results are shown from fitting CInd and L‐L models to data simulated from each DGM, and from fitting L‐L models with regularized horseshoe priors to data simulated from L‐L models. As all fitted models produced unbiased estimates for the HIV data, detailed bias results are not presented in the main text, but are provided in Appendix F in Supporting Information for completeness. In addition, an overall summary of biases across all sample sizes for the fitted CInd model, the L‐L model with the correct interaction terms, and the L‐L model with all interactions under shrinkage priors for the four data sets is provided in Appendix G (Supporting Information).

#### 5.1.1 Simulations Based on the VL Data Set

As we would expect, L‐L models produced unbiased estimates when the true DGM was also L‐L (Figure 1). Notably, L‐L models also performed well under the fixed‐effect DGM, providing unbiased parameter estimates. When data were generated from a latent trait model, L‐L models incorporating relevant pairwise dependencies produced lower biases than the CInd model, but with some bias on average in estimates of prevalence (mean bias: −0.031), the sensitivity of each test (mean biases: 0.034 to 0.065), and specificity of Test 1 (mean bias: 0.022). Nevertheless, the 95% empirical intervals of the biases included or were very close to zero for all of these parameters (Figure 1), that is, fitted L‐L models produced unbiased or almost unbiased parameter estimates for some of the simulated data sets. Additionally, with a larger sample size (5000) average biases were nearly halved for all parameters, while the biases from the CInd model remained almost unchanged (Table D.15 in Appendix D in the Supporting Information).

#### 5.1.2 Simulations Based on the CPTB Data Set

L‐L models incorporating all relevant pairwise interactions yielded reduced absolute biases for all parameters across all DGMs, compared with the CInd model (Figure 2). However, under the latent trait and fixed‐effect DGMs, L‐L models still underestimated prevalence (mean absolute biases: −0.037 and −0.049, respectively) and substantially overestimated the sensitivities of Test 1 (0.111 and 0.130) and Test 2 (0.101 and 0.092), respectively. Even in data sets simulated from the L‐L DGM, both the L‐L models with ‘correct’ interactions (true model) and those including all interactions with regularized horseshoe priors within the diseased state tended to overestimate the sensitivity of Test 1 (mean biases: 0.072 and 0.078, respectively) and Test 2 (0.066 and 0.069, respectively). Despite these high mean absolute biases, the 95% empirical intervals for bias were particularly wide and crossed zero for these parameters, for each of the DGMs. As with the HIV and VL data sets, the 95% empirical intervals for bias in all other parameters also either included or came very close to zero when L‐L models were fitted, for all DGMs (Figure 2).

Increasing the sample size to 5000 substantially reduced the mean biases produced by the L‐L models with ‘correct’ pairwise interactions across all DGMs, whereas bias in estimates from the CInd model remained similar (Tables D.27, D.29, D.31 in Appendix D in the Supporting Information). The L‐L model with regularized horseshoe priors produced biases closely matching those from the L‐L model with correct interactions only (Figure 2), including a similar reduction in bias with increased sample size (Tables D.33, D.35, D.37 in Appendix D in the Supporting Information).

#### 5.1.3 Simulations Based on the CDPN Data Set

L‐L models with interactions again reduced biases, relative to the CInd model, across data sets simulated from different DGMs (Figure 3). L‐L models including only the relevant pairwise interactions slightly underestimated prevalence on average, with mean bias ranging from −0.019 (latent trait) to −0.027 (fixed‐effect) across all DGMs.

When data were simulated from the latent trait model, L‐L models with interactions produced low biases, with none exceeding a mean of 0.020, for all sensitivities and specificities (Figure 3). Increasing the sample size from 2000 to 5000 reduced the mean biases for the sensitivities of Tests 1–4 and the specificity of Test 5, but increased the mean biases for the prevalence, sensitivity of Test 5, and specificities of Tests 1–4 (Table D.39 in Appendix D in the Supporting Information).

Across data sets simulated from the fixed‐effect DGM, the sensitivity of the independent test (Test 5) tended to be overestimated (mean bias 0.039) and the specificities of Tests 1–4 slightly underestimated (mean biases of around −0.029), although with 95% empirical intervals for these biases all including or very close to zero (Figure 3). Unlike all other settings and DGMs, increasing the sample size from 2000 to 5000 led to increased absolute mean biases for each parameter (Table D.41 in Appendix D in the Supporting Information).

When the DGM was L‐L, some biases tended to remain even when the true L‐L model was fitted: mean biases for the sensitivities of the dependent tests = 0.028, mean bias for the specificity of the independent test = −0.029. However, the 95% empirical intervals for bias included zero for all parameters except the specificity of Test 5 and came very close to zero for this additional parameter. These biases were reduced by around 0.010 when sample sizes were increased to 5000 (Table D.43 in Appendix D in the Supporting Information).

Across all sample sizes, the fitted L‐L models produced higher mean biases for the sensitivities of Tests 1–4 (the correlated tests), but lower mean bias for the sensitivity of Test 5, when the DGM was also L‐L, compared to the latent trait and fixed‐effect DGMs (Tables D.39, D.41, and D.43 in Appendix D in the Supporting Information). In contrast, across all sample sizes, the L‐L model with the correct interactions tended to produce unbiased estimates of specificities of Tests 1–4 when data were simulated from the L‐L DGM (Table D.43 in Appendix D in the Supporting Information). However, it yielded higher bias for the specificity of Test 5 compared to models fitted to data generated from the fixed‐effect or latent trait DGMs regardless of the sample size (Tables D.39, D.41, and D.43 in Appendix D in the Supporting Information).

The L‐L model with all pairwise interactions and regularized horseshoe priors produced substantially larger biases for most parameters compared to the correct L‐L model, except for the specificities of the conditionally dependent tests (Tests 1–4) (Figure 3). Additionally, the 95% empirical intervals did not include zero for the sensitivities of Tests 1–4 and the specificity of Test 5. This finding is in contrast to the findings from the HIV, VL and CPTB data sets, but notably the L‐L models that we fitted here included substantially more pairwise interaction terms (all pairwise interactions in both disease states, rather than within the diseased state only). Nonetheless, the biases from the L‐L model with shrinkage priors were lower than those from the CInd model (Figure 3).

### 5.2 Coverage

Both the CInd and L‐L models generally achieved coverage close to the nominal 95% across each DGM for the HIV data (Tables D.4, D.6, D.8 in the Supporting Information). For each of the other data sets, the 95% CrI coverages from the L‐L models were both more consistent and generally closer to the nominal 95% than those from the CInd models (Appendix D in Supporting Information). L‐L models with shrinkage priors tended to produce coverages exceeding the nominal 95%, which likely reflects the increased uncertainty in estimating sensitivities, specificities, and prevalence when all interaction terms were included in the model. More detailed coverage results for a sample size of 2000 for each data set are provided in Appendix E (Supporting Information).

### 5.3 Model Fit

Table 2 presents mean residual deviances and DIC values for the data sets with a sample size of 2000. The CInd model is seen to fit poorly throughout, with mean residual deviance far exceeding the number of unconstrained data points. Both the residual deviance and DIC are seen to reduce when L‐L models are fitted, consistently across data sets and DGMs. These differences became more pronounced when sample size was increased (see Appendix D in Supporting Information). Notably, this improvement in model fit is observed even for the HIV data scenario, contrary to the unbiased parameter estimates and similar coverages provided by the CInd and L‐L models (Table 2). Except for the fixed‐effect DGM based on the CDPN data set, the L‐L model with correct interaction terms produced ResDs close to the number of unconstrained data points in each setting.

**TABLE 2:** Mean of the posterior mean residual deviances (ResD) and mean DIC values across converged CInd and L‐L models with correct pairwise interactions fitted to the 1250 data sets simulated from each DGM and L‐L models with regularized horseshoe (RH) priors fitted to the 500 data sets simulated from the L‐L DGMs in all data settings (sample size = 2000).

Data set	DGM	Model	ResD	DIC	Number of unconstrained cells^a^
HIV	Latent trait	CInd	79.42 (51.80, 113.22)	88.91 (61.25, 123.01)	15
		L‐L	15.81 (11.35, 22.55)	26.63 (21.98, 33.29)	
	Fixed‐effect	CInd	76.56 (47.32, 110.80)	85.95 (56.98, 120.25)	
		L‐L	18.24 (12.38, 26.26)	31.32 (23.04, 42.63)	
	L‐L	CInd	54.13 (31.92, 79.28)	63.57 (41.17, 88.62)	
		L‐L	15.57 (11.10, 23.20)	26.08 (21.42, 33.55)	
		L‐L with RH priors	15.02 (11.75, 20.78)	26.30 (22.34, 33.29)	
VL	Latent trait	CInd	163.62 (114.70, 216.91)	172.65 (123.73, 226.01)	15
		L‐L	17.97 (13.74, 26.01)	33.01 (27.19, 42.13)	
	Fixed‐effect	CInd	215.70 (156.10, 281.76)	224.67 (165.08, 290.38)	
		L‐L	15.87 (12.18, 23.05)	28.22 (24.31, 35.46)	
	L‐L	CInd	209.42 (150.55, 274.34)	218.39 (159.43, 283.10)	
		L‐L	15.81 (12.16, 22.58)	28.09 (24.24, 34.98)	
		L‐L with RH priors	14.69 (12.65, 17.76)	28.01 (24.35, 34.01)	
CPTB	Latent trait	CInd	98.85 (70.81, 131.93)	100.82 (81.65, 142.79)	31
		L‐L	33.67 (25.40, 45.44)	53.10 (43.63, 66.31)	
	Fixed‐effect	CInd	194.60 (148.48, 245.02)	205.77 (159.65, 257.15)	
		L‐L	39.96 (28.82, 56.06)	60.44 (47.17, 78.70)	
	L‐L	CInd	191.52 (146.89, 242.45)	202.89 (158.27, 254.22)	
		L‐L	35.39 (25.23, 48.23)	55.72 (43.95, 70.21)	
		L‐L with RH priors	34.76 (26.40, 46.51)	57.97 (48.00, 70.87)	
CDPN	Latent trait	CInd	127.74 (90.57, 170.11)	138.70 (101.60, 181.02)	31
		L‐L	35.46 (23.70, 50.69)	49.81 (37.79, 65.40)	
	Fixed‐effect	CInd	219.58 (168.86, 272.31)	230.55 (179.87, 283.29)	
		L‐L	71.64 (46.29, 98.87)	86.66 (61.63, 114.12)	
	L‐L	CInd	94.21 (64.07, 129.88)	105.17 (75.03, 140.86)	
		L‐L	32.18 (21.44, 45.83)	44.67 (33.82, 58.28)	
		L‐L with RH priors	31.43 (26.73, 38.53)	58.53 (51.85, 69.37)	

*Note:* 2.5th and 97.5th percentiles are given in brackets.

^a^ Number of unconstrained cells in a contingency table, that is, $2^{J}-1$ where J is the number of diagnostic tests under evaluation.

‘Correct’ L‐L models and L‐L models including all interaction terms with regularized horseshoe priors produced very similar ResDs for all four data sets, but with a 12‐point increase in DIC for the CDPN data set, reflecting the increased complexity of a model with 20 interaction terms, even with shrinkage priors. As different DGMs simulate different data sets, the mean DIC and ResD values obtained from the fitted models are not directly comparable across DGMs.

### 5.4 Selection Performance of Shrinkage Priors for Pairwise Dependencies

Table 3 shows correct and false inclusion rates (CIR, FIR) for pairwise interaction terms, for the L‐L DGM with a sample size of 2000. For each type of shrinkage prior and data setting, the table shows the CIR and FIR based on the 95% CrI criteria (i.e., proportion of simulated data sets for which the 95% CrIs for interaction terms excluded zero, correctly or incorrectly, respectively), and based on ‘optimal’ CrI criteria as described in Section 4.5. There was no clear pattern indicating that any one type of shrinkage prior outperformed the others in terms of achieving lower distances.

**TABLE 3:** Distances, correct inclusion rates (CIR), and false inclusion rates (FIR) for CrIs providing minimum distance and 95% CrIs obtained using hyperlasso (HL), elastic net (EN), and regularized horseshoe (RH) priors on interaction terms across 500 simulated data sets simulated from L‐L DGM with the sample size of 2000 in each data setting.

Setting	Prior	CrI with the minimum distance				95% CrI		
		Selected CrI	Distance	CIR	FIR	Distance	CIR	FIR
HIV	HL	95%	0.167	1.000	0.167	0.167	1.000	0.167
	EN	95%	0.146	1.000	0.146	0.146	1.000	0.146
	RH	95%	0.127	1.000	0.127	0.127	1.000	0.127
VL	HL	60%	0.333	0.693	0.129	0.478	0.522	0.006
	EN	70%	0.334	0.724	0.188	0.441	0.559	0.017
	RH	60%	0.335	0.692	0.131	0.478	0.522	0.005
CPTB	HL	60%	0.350	0.762	0.256	0.539	0.462	0.018
	EN	80%	0.342	0.711	0.184	0.462	0.539	0.042
	RH	70%	0.351	0706	0.192	0.526	0.474	0.020
CDPN	HL	60%	0.537	0.492	0.173	0.870	0.130	0.006
	EN	60%	0.539	0.523	0.250	0.853	0.147	0.013
	RH	60%	0.531	0.526	0.239	0.868	0.132	0.011

For ease of comparison, results in this paragraph are based on the 95% CrI exclusion criteria and the sample size of 2000 (Table 3). For the simplest CDep structure (HIV data), the interaction term for the true correlated pair (Test 2 × Test 3) was always correctly identified, but interaction terms for other test pairs were also (incorrectly) identified 12%–17% of the time. For the VL data, true pairwise interactions were identified less reliably (CIR 53%–56%), but incorrect pairwise dependencies were very rarely included (FIR 1%–2%). Performance of the shrinkage priors was broadly similar but slightly poorer for the CPTB data (CIR 46%–54%, FIR 2%–4%). For the most complex CDPN data set, FIRs were very low (∼1%) but CIRs dropped markedly, with correct interaction terms only identified 13%–15% of the time.

CIRs and FIRs for other sample sizes are available in the Supporting Information (Appendix D). As expected, CIRs increased with larger sample sizes. Notably, with a sample size of 5000, the ability of the 95% CrI criterion to identify the ‘correct’ pairwise interactions improved substantially for the VL, CPTB, and CDPN data sets (Tables D.25, D.37, and D.49 in the Supporting Information).

As the correlation structure became more complex, the width of the optimal CrIs decreased, while the distances increased. In other words, narrower CrIs provided a better trade‐off between CIRs and FIRs for the VL, CPTB, and CDPN data sets (Table 3). As expected, adopting a narrower CrI criterion increased the FIRs for these data sets because narrower intervals are more likely to exclude zero, thereby increasing the chance of falsely identifying interactions that are not present (Table 3).

### 5.5 Computational Performance

The L‐L model incorporating the correct pairwise interaction terms required approximately 1.3 s to achieve convergence, while the L‐L model with shrinkage priors required approximately 6.6 s. The fixed‐effect model corresponding to Equation (7) required approximately 4.1 s. The L‐L model with correct interactions and the fixed‐effect model converged within 1000 iterations, whereas the L‐L model with shrinkage priors and the latent trait model required 10 000 iterations to achieve convergence under the same criteria. The latent trait model required substantially longer runtime, taking approximately 50.7 min.

## 6 Discussion

We evaluated the performance of latent class log‐linear (L‐L) models with pairwise interaction terms across a range of plausible data‐generating mechanisms (DGMs) reflecting real‐world diagnostic settings. Our findings indicate that L‐L models provide a flexible and generally robust approach for estimating diagnostic test accuracy in the presence of conditional dependence, yielding good model fit, low bias—with 95% empirical intervals including or very close to zero in the vast majority of scenarios—and appropriate coverage across a variety of settings.

When the assumed dependence structure was correctly specified, L‐L models performed well across all DGMs considered. When applied to data generated from latent trait or fixed‐effect models, L‐L models incorporating relevant pairwise interactions still produced relatively low bias and good model fit, although some bias remained in certain scenarios. This is not unexpected, as these DGMs may imply higher‐order dependencies that are not fully captured by pairwise interactions on the log‐linear scale. For example, the fixed‐effect DGM used for the CPTB data set [6] explicitly allows for higher‐order dependencies among Tests 1–4, and therefore cannot be fully represented by pairwise interactions alone. In addition, some simplifying assumptions were made in specifying L‐L models, such as sharing interaction terms across test pairs to reduce the number of parameters. While this improves identifiability and computational feasibility, it may not fully reflect the underlying dependence structure, particularly when dependencies differ in magnitude across test pairs. As a result, some degree of bias may arise when these assumptions do not align with the true data‐generating mechanism.

Excluding the most complex synthetic scenario (CDPN), L‐L models with pairwise interactions provided good model fit based on residual deviance. However, one finding was initially somewhat surprising and disappointing: with a sample size of 2000, some bias persisted under the CPTB and CDPN data sets, even when the true DGM was L‐L and the correct L‐L model was fitted. These biases were, however, substantially reduced when sample size was increased to 5000. On reflection, this likely highlights a broader challenge with fitting CDep latent class models, where the effects of CDep and sampling error may be difficult to distinguish at low sample sizes [54]. For these two data sets, when the sample size was 2000, the 95% empirical intervals—particularly for biased parameters—were wide across all DGMs, suggesting that the sample size was likely insufficient for precise estimation. These intervals generally narrowed when the sample size increased to 5000.

Insufficient sample size likely also contributed to the biases observed with a sample size of 2000 for L‐L models fitted to data simulated from latent trait models for the VL and CPTB data sets and from fixed‐effect models for the CPTB data set. As noted above, these biases tended to reduce when sample size was increased to 5000. These CDep models each described quite complex correlation structures among Test 1–4 within the diseased state, alongside lower prevalences compared to the HIV and CDPN settings. The combination of relatively low prevalences and high correlations within the diseased state may lead to very little information with which to estimate dependence terms, unless sample size is large. Similar findings have been reported in several studies involving different CDep models, where parameter estimates remained somewhat biased despite fitting the true data‐generating CDep models [6, 18, 19, 26]. This highlights that even correctly specified latent class models may yield biased results under limited sample sizes or complex dependency structures. Hence, the higher bias with smaller sample sizes in this study may be attributed to limited information—specifically, sparse contingency tables that provide insufficient data to estimate dependence terms under complex CDep structures—rather than to any inherent weakness of L‐L models at smaller sample sizes.

Since pre‐specifying which interaction terms need to be included in an L‐L model may often be challenging, use of shrinkage priors on interaction parameters is appealing. Our ability to evaluate use of these in this simulation study was limited due to identifiability issues, high autocorrelations in chains and subsequent long run times. We subsequently evaluated their use only for L‐L DGMs. For three of our four data sets, in which we only allowed for possible pairwise interactions within the diseased state, satisfactory convergence and Monte Carlo error were observed, and models with shrinkage priors produced model fits and biases similar to L‐L models with relevant interactions. For the fourth data set (CDPN), we attempted to allow for all possible pairwise interactions in both disease states using shrinkage priors, but convergence and/or chain mixing was observed to be poor. We conclude that shrinkage priors may be a useful tool in this setting if CDep is only believed to occur in one disease state—and of course conditional on chains converging and sufficiently low levels of Monte Carlo error being obtained. The performance of the shrinkage priors in identifying the correct pairwise correlations was limited for smaller sample sizes (but improved for a sample size of 5000) when the CDep structure became more complex, particularly when the 95% CrI criterion was used. However, it is worth noting that, under this same criterion, ‘wrong’ interactions were rarely included. For these scenarios, the use of narrower CrIs offered a better trade‐off between correct and false inclusion. However, depending on the context, the inclusion or exclusion of specific interaction terms may be of limited practical importance. If the primary aim is to obtain unbiased estimates of diagnostic accuracy, rather than to characterize the underlying dependence structure, then accurate estimation of the key parameters—sensitivity and specificity—may be sufficient.

Latent class L‐L models are an attractive option because they can be fitted to aggregated data sets (substantially reducing run times) and naturally ensure that all estimated probabilities lie within the [0,1] range, rather than requiring explicit enforcement of potentially complex constraints. The computational results highlighted clear differences in efficiency across model classes. L‐L models with correctly specified interaction terms achieved convergence rapidly and with relatively few iterations, while models incorporating shrinkage priors required increased iterations due to slower mixing. In contrast, latent trait models required substantially longer runtimes and a much larger number of iterations to achieve comparable convergence criteria. Fixed‐effect models exhibited intermediate computational performance but required additional implementation steps to enforce valid probability constraints. These findings indicate that, while more complex models may offer additional flexibility, L‐L models provide a favorable balance between computational efficiency and modeling capability.

A key strength of this study is that the data were generated from different types of CDep latent class models, each inducing distinct correlation structures based on real‐world scenarios in three settings. This approach mirrors the strategy used by van Smeden et al. [55] to investigate the performance of chi‐squared statistics in detecting misfit in diagnostic latent class models. Additionally, a hypothetical scenario [7] assuming dependence within both disease states was included to evaluate the performance of latent class L‐L models in the context of complex correlation structures. The number of simulated data sets (1250) from each DGM and the total number of fitted models (108 000: 45 000 CInd models, 45 000 L‐L models with known correlation structures, and 18 000 L‐L models with shrinkage priors) were substantial, reflecting the scale of this comprehensive simulation study with 36 unique scenarios.

This study also has several limitations. Although the DGMs were informed by real diagnostic applications, the true data‐generating processes for these data sets are unknown. Therefore, the DGMs simulated from represent plausible rather than definitive mechanisms. Nonetheless, this does not undermine the ability of this study to evaluate the performance of L‐L models and shrinkage priors under varied and plausible dependence structures. As noted above, when fitting L‐L models, we made some simplifying assumptions about shared interaction terms, which may not have correctly represented the pairwise dependencies implied by the DGMs. Additionally, we only fitted L‐L models with pairwise interaction terms, whereas some DGMs likely imply higher‐order interaction terms on the logit probability scale. Relaxing these assumptions may have reduced or removed bias in fitted L‐L models but—on the other hand—would substantially increase the number of parameters to be estimated, such that some models may not have been identifiable. When fitting L‐L models with shrinkage priors, we observed high autocorrelations in the MCMC process, even after assuming dependency only within the correct disease states. For this reason, we increased the number of iterations when fitting these L‐L models and consequently limited the fitting of these models to 500 data sets simulated from the L‐L DGM in each setting, due to long run times. The convergence issues with the L‐L models, particularly those with all interactions (for the CDPN data), likely stem from model non‐identifiability due to the excessive number of parameters. A potential solution to this issue could be to use informative priors on the prevalence or some accuracy parameters, to reduce the number of parameters to be estimated.

Future work could involve comparing the performance of latent class L‐L models in estimating diagnostic test accuracy with that of alternative CDep models (e.g., latent trait and/or fixed‐effect models), with all models fitted to the same data sets simulated from different DGMs. This would help determine whether the biases observed with some L‐L models arise from limited information to estimate complex dependence structures or from limitations inherent to the L‐L formulation itself. However, such a comparison was beyond the scope of the present study due to substantial practical challenges. In particular, latent trait models are computationally demanding and often require very large numbers of MCMC iterations to achieve adequate mixing and effective sample sizes, while fixed‐effect models present additional difficulties related to the need for complex constraints to ensure valid probability estimates. Addressing these challenges in a comprehensive simulation framework remains an important direction for future research.

Future work could also explore whether incorporating higher‐order interaction terms in an L‐L model could be valuable, as pairwise interactions may not fully reflect the complexity of some real DGMs. Furthermore, using Stan instead of JAGS for fitting models with shrinkage priors may be preferable in future studies due to its potential in providing better mixing and convergence properties.

## Funding

This study was funded by the NIHR Health Protection Research Unit in Behavioral Science and Evaluation at University of Bristol, in partnership with UK Health Security Agency (UKHSA) and the Turkish Ministry of National Education. HEJ also acknowledges support from an MRC‐NIHR New Investigator Research Grant (MR/T044594/1). The views expressed are those of the authors and not necessarily those of the NIHR, the Department of Health and Social Care, or UKHSA.

## Conflicts of Interest

The authors declare no conflicts of interest.

## Supporting information


**Appendix A–G** Supporting information.
**Table B.1** Parameter values for the sensitivities, specificities, slope parameters, and prevalence to simulate data from the latent trait model based on the HIV data.
**Table B.2** Parameter values for the sensitivities, specificities, covariances, and prevalence to simulate data from the fixed effect model based on the HIV data.
**Table B.3** Parameter values for the sensitivities, specificities, main effects, interaction, and prevalence to simulate data from the log‐linear model based on the HIV data.
**Table B.4** Parameter values for the sensitivities, specificities, slope parameters, and prevalence to simulate data from the latent trait model based on the VL data.
**Table B.5** Parameter values for the sensitivities, specificities, covariances, and prevalence to simulate data from the fixed effect model based on the VL data.
**Table B.6** Parameter values for the sensitivities, specificities, main effects, interaction, and prevalence to simulate data from the log‐linear model based on the VL data.
**Table B.7** Parameter values for the sensitivities, specificities, slope parameters, and prevalence to simulate data from the latent trait model based on the CPTB data.
**Table B.8** Parameter values for the sensitivities, specificities, dependency terms, and prevalence to simulate data from the fixed effect model based on the CPTB data.
**Table B.9** Parameter values for the sensitivities, specificities, main effects, interaction, and prevalence to simulate data from the log‐linear model based on the CPTB data.
**Table B.10** Parameter values for the sensitivities, specificities, slope parameters, and prevalence to simulate data from the latent trait model based on the CDPN scenario.
**Table B.11** Parameter values for the sensitivities, specificities, covariances, and prevalence to simulate data from the fixed effect model based on the CDPN scenario.
**Table B.12** Parameter values for the sensitivities, specificities, main effects, interaction, and prevalence to simulate data from the log‐linear model based on the CPTB data.
**Figure C.1** Required number of simulations (*n*_req_) for each maximum value of Monte Carlo Standard Error (MCSE) of biases, based on the maximum empirical variance estimate from the initial 100 simulation runs.
**Table D.1** Number of converged CInd and L‐L models with known correlation structure fitted to the 1,250 data sets simulated from three different DGMs (LT, FE, and L‐L) in four different data settings (HIV, VL, CPTB, and CDPN) for different sample sizes. The percentages of converged models in each scenario are also presented in brackets.
**Table D.2** Number of converged L‐L models with three different shrinkage priors (HL, EN, RH) fitted to the 500 data sets simulated from L‐L DGMs in four different data settings (HIV, VL, CPTB, and CDPN) for different sample sizes. The percentages of converged models in each scenario are also presented in brackets.
**Table D.3** Mean absolute biases of posterior median estimates for prevalence, sensitivities, and specificities with mean residual deviances and DIC values across converged CInd and L‐L models with known correlation structures fitted to the 1,250 data sets simulated from LT DGM in HIV data setting. Estimates are given with 2.5th and 97.5th percentiles.
**Table D.4** 95% CrI coverages for prevalence, sensitivities, and specificities across converged CInd and L‐L models with known correlation structures fitted to the 1,250 data sets simulated from LT DGM in HIV data setting. Coverages are presented as percentages along with 95% Monte Carlo confidence intervals.
**Table D.5** Mean absolute biases of posterior median estimates for prevalence, sensitivities, and specificities with mean residual deviances and DIC values across converged CInd and L‐L models with known correlation structures fitted to the 1,250 data sets simulated from FE DGM in HIV data setting. Estimates are given with 2.5th and 97.5th percentiles.
**Table D.6** 95% CrI coverages for prevalence, sensitivities, and specificities across converged CInd and L‐L models with known correlation structures fitted to the 1,250 data sets simulated from FE DGM in HIV data setting. Coverages are presented as percentages along with 95% Monte Carlo confidence intervals.
**Table D.7** Mean absolute biases of posterior median estimates for prevalence, sensitivities, and specificities with mean residual deviances and DIC values across converged CInd and L‐L models with known correlation structures fitted to the 1,250 data sets simulated from L‐L DGM in HIV data setting. Estimates are given with 2.5th and 97.5th percentiles.
**Table D.8** 95% CrI coverages for prevalence, sensitivities, and specificities across converged CInd and L‐L models with known correlation structures fitted to the 1,250 data sets simulated from L‐L DGM in HIV data setting. Coverages are presented as percentages along with 95% Monte Carlo confidence intervals.
**Table D.9** Mean absolute biases of posterior median estimates for prevalence, sensitivities, and specificities with mean residual deviances, DIC values, CrI widths providing minimum distances, corresponding correct inclusion rates (CIR) and false inclusion rates (FIR) of the interaction terms, as well as 95% CrI distances, CIRs and FIRs across converged L‐L models with all interaction terms within the diseased state with three different priors (Hyplerlasso, Elastic Net, Regularized Horseshoe) fitted to the 500 data sets simulated from L‐L DGM in HIV data setting with the sample sizes of 500. Estimates are given with 2.5th and 97.5th percentiles.
**Table D.10** 95% CrI coverages for prevalence, sensitivities, and specificities across converged L‐L models with all interaction terms within the diseased state with three different priors (Hyplerlasso, Elastic Net, Regularized Horseshoe) fitted to the 500 data sets simulated from L‐L DGM in HIV data setting with the sample sizes of 500. Coverages are presented as percentages along with 95% Monte Carlo confidence intervals.
**Table D.11** Mean absolute biases of posterior median estimates for prevalence, sensitivities, and specificities with mean residual deviances, DIC values, CrI widths providing minimum distances, corresponding correct inclusion rates (CIR) and false inclusion rates (FIR) of the interaction terms, as well as 95% CrI distances, CIRs and FIRs across converged L‐L models with all interaction terms within the diseased state with three different priors (Hyplerlasso, Elastic Net, Regularized Horseshoe) fitted to the 500 data sets simulated from L‐L DGM in HIV data setting with the sample sizes of 2000. Estimates are given with 2.5th and 97.5th percentiles.
**Table D.12** 95% CrI coverages for prevalence, sensitivities, and specificities across converged L‐L models with all interaction terms within the diseased state with three different priors (Hyplerlasso, Elastic Net, Regularized Horseshoe) fitted to the 500 data sets simulated from L‐L DGM in HIV data setting with the sample sizes of 2000. Coverages are presented as percentages along with 95% Monte Carlo confidence intervals.
**Table D.13** Mean absolute biases of posterior median estimates for prevalence, sensitivities, and specificities with mean residual deviances, DIC values, CrI widths providing minimum distances, corresponding correct inclusion rates (CIR) and false inclusion rates (FIR) of the interaction terms, as well as 95% CrI distances, CIRs and FIRs across converged L‐L models with all interaction terms within the diseased state with three different priors (Hyplerlasso, Elastic Net, Regularized Horseshoe) fitted to the 500 data sets simulated from L‐L DGM in HIV data setting with the sample sizes of 5000. Estimates are given with 2.5th and 97.5th percentiles.
**Table D.14** 95% CrI coverages for prevalence, sensitivities, and specificities across converged L‐L models with all interaction terms within the diseased state with three different priors (Hyplerlasso, Elastic Net, Regularized Horseshoe) fitted to the 500 data sets simulated from L‐L DGM in HIV data setting with the sample sizes of 5000. Coverages are presented as percentages along with 95% Monte Carlo confidence intervals.
**Table D.15** Mean absolute biases of posterior median estimates for prevalence, sensitivities, and specificities with mean residual deviances and DIC values across converged CInd and L‐L models with known correlation structures fitted to the 1,250 data sets simulated from LT DGM in VL data setting. Estimates are given with 2.5th and 97.5th percentiles.
**Table D.16** 95% CrI coverages for prevalence, sensitivities, and specificities across converged CInd and L‐L models with known correlation structures fitted to the 1,250 data sets simulated from LT DGM in VL data setting. Coverages are presented as percentages along with 95% Monte Carlo confidence intervals.
**Table D.17** Mean absolute biases of posterior median estimates for prevalence, sensitivities, and specificities with mean residual deviances and DIC values across converged CInd and L‐L models with known correlation structures fitted to the 1,250 data sets simulated from FE DGM in VL data setting. Estimates are given with 2.5th and 97.5th percentiles.
**Table D.18** 95% CrI coverages for prevalence, sensitivities, and specificities across converged CInd and L‐L models with known correlation structures fitted to the 1,250 data sets simulated from FE DGM in VL data setting. Coverages are presented as percentages along with 95% Monte Carlo confidence intervals.
**Table D.19** Mean absolute biases of posterior median estimates for prevalence, sensitivities, and specificities with mean residual deviances and DIC values across converged CInd and L‐L models with known correlation structures fitted to the 1,250 data sets simulated from L‐L DGM in VL data setting. Estimates are given with 2.5th and 97.5th percentiles.
**Table D.20** 95% CrI coverages for prevalence, sensitivities, and specificities across converged CInd and L‐L models with known correlation structures fitted to the 1,250 data sets simulated from L‐L DGM in VL data setting. Coverages are presented as percentages along with 95% Monte Carlo confidence intervals.
**Table D.21** Mean absolute biases of posterior median estimates for prevalence, sensitivities, and specificities with mean residual deviances, DIC values, CrI widths providing minimum distances, corresponding correct inclusion rates (CIR) and false inclusion rates (FIR) of the interaction terms, as well as 95% CrI distances, CIRs and FIRs across converged L‐L models with all interaction terms within the diseased state with three different priors (Hyplerlasso, Elastic Net, Regularized Horseshoe) fitted to the 500 data sets simulated from L‐L DGM in VL data setting with the sample sizes of 500. Estimates are given with 2.5th and 97.5th percentiles.
**Table D.22** 95% CrI coverages for prevalence, sensitivities, and specificities across converged L‐L models with all interaction terms within the diseased state with three different priors (Hyplerlasso, Elastic Net, Regularized Horseshoe) fitted to the 500 data sets simulated from L‐L DGM in VL data setting with the sample sizes of 500. Coverages are presented as percentages along with 95% Monte Carlo confidence intervals.
**Table D.23** Mean absolute biases of posterior median estimates for prevalence, sensitivities, and specificities with mean residual deviances, DIC values, CrI widths providing minimum distances, corresponding correct inclusion rates (CIR) and false inclusion rates (FIR) of the interaction terms, as well as 95% CrI distances, CIRs and FIRs across converged L‐L models with all interaction terms within the diseased state with three different priors (Hyplerlasso, Elastic Net, Regularized Horseshoe) fitted to the 500 data sets simulated from L‐L DGM in VL data setting with the sample sizes of 2000. Estimates are given with 2.5th and 97.5th percentiles.
**Table D.24** 95% CrI coverages for prevalence, sensitivities, and specificities across converged L‐L models with all interaction terms within the diseased state with three different priors (Hyplerlasso, Elastic Net, Regularized Horseshoe) fitted to the 500 data sets simulated from L‐L DGM in VL data setting with the sample sizes of 2000. Coverages are presented as percentages along with 95% Monte Carlo confidence intervals.
**Table D.25** Mean absolute biases of posterior median estimates for prevalence, sensitivities, and specificities with mean residual deviances, DIC values, CrI widths providing minimum distances, corresponding correct inclusion rates (CIR) and false inclusion rates (FIR) of the interaction terms, as well as 95% CrI distances, CIRs and FIRs across converged L‐L models with all interaction terms within the diseased state with three different priors (Hyplerlasso, Elastic Net, Regularized Horseshoe) fitted to the 500 data sets simulated from L‐L DGM in VL data setting with the sample sizes of 5000. Estimates are given with 2.5th and 97.5th percentiles.
**Table D.26** 95% CrI coverages for prevalence, sensitivities, and specificities across converged L‐L models with all interaction terms within the diseased state with three different priors (Hyplerlasso, Elastic Net, Regularized Horseshoe) fitted to the 500 data sets simulated from L‐L DGM in VL data setting with the sample sizes of 5000. Coverages are presented as percentages along with 95% Monte Carlo confidence intervals.
**Table D.27** Mean absolute biases of posterior median estimates for prevalence, sensitivities, and specificities with mean residual deviances and DIC values across converged CInd and L‐L models with known correlation structures fitted to the 1,250 data sets simulated from LT DGM in CPTB data setting. Estimates are given with 2.5th and 97.5th percentiles.
**Table D.28** 95% CrI coverages for prevalence, sensitivities, and specificities across converged CInd and L‐L models with known correlation structures fitted to the 1,250 data sets simulated from LT DGM in CPTB data setting. Coverages are presented as percentages along with 95% Monte Carlo confidence intervals.
**Table D.29** Mean absolute biases of posterior median estimates for prevalence, sensitivities, and specificities with mean residual deviances and DIC values across converged CInd and L‐L models with known correlation structures fitted to the 1,250 data sets simulated from FE DGM in CPTB data setting. Estimates are given with 2.5th and 97.5th percentiles.
**Table D.30** 95% CrI coverages for prevalence, sensitivities, and specificities across converged CInd and L‐L models with known correlation structures fitted to the 1,250 data sets simulated from FE DGM in CPTB data setting. Coverages are presented as percentages along with 95% Monte Carlo confidence intervals.
**Table D.31** Mean absolute biases of posterior median estimates for prevalence, sensitivities, and specificities with mean residual deviances and DIC values across converged CInd and L‐L models with known correlation structures fitted to the 1,250 data sets simulated from L‐L DGM in CPTB data setting. Estimates are given with 2.5th and 97.5th percentiles.
**Table D.32** 95% CrI coverages for prevalence, sensitivities, and specificities across converged CInd and L‐L models with known correlation structures fitted to the 1,250 data sets simulated from L‐L DGM in CPTB data setting. Coverages are presented as percentages along with 95% Monte Carlo confidence intervals.
**Table D.33** Mean absolute biases of posterior median estimates for prevalence, sensitivities, and specificities with mean residual deviances, DIC values, CrI widths providing minimum distances, corresponding correct inclusion rates (CIR) and false inclusion rates (FIR) of the interaction terms, as well as 95% CrI distances, CIRs and FIRs across converged L‐L models with all interaction terms within the diseased state with three different priors (Hyplerlasso, Elastic Net, Regularized Horseshoe) fitted to the 500 data sets simulated from L‐L DGM in CPTB data setting with the sample sizes of 500. Estimates are given with 2.5th and 97.5th percentiles.
**Table D.34** 95% CrI coverages for prevalence, sensitivities, and specificities across converged L‐L models with all interaction terms within the diseased state with three different priors (Hyplerlasso, Elastic Net, Regularized Horseshoe) fitted to the 500 data sets simulated from L‐L DGM in CPTB data setting with the sample sizes of 500. Coverages are presented as percentages along with 95% Monte Carlo confidence intervals.
**Table D.35** Mean absolute biases of posterior median estimates for prevalence, sensitivities, and specificities with mean residual deviances, DIC values, CrI widths providing minimum distances, corresponding correct inclusion rates (CIR) and false inclusion rates (FIR) of the interaction terms, as well as 95% CrI distances, CIRs and FIRs across converged L‐L models with all interaction terms within the diseased state with three different priors (Hyplerlasso, Elastic Net, Regularized Horseshoe) fitted to the 500 data sets simulated from L‐L DGM in CPTB data setting with the sample sizes of 2000. Estimates are given with 2.5th and 97.5th percentiles.
**Table D.36** 95% CrI coverages for prevalence, sensitivities, and specificities across converged L‐L models with all interaction terms within the diseased state with three different priors (Hyplerlasso, Elastic Net, Regularized Horseshoe) fitted to the 500 data sets simulated from L‐L DGM in CPTB data setting with the sample sizes of 2000. Coverages are presented as percentages along with 95% Monte Carlo confidence intervals.
**Table D.37** Mean absolute biases of posterior median estimates for prevalence, sensitivities, and specificities with mean residual deviances, DIC values, CrI widths providing minimum distances, corresponding correct inclusion rates (CIR) and false inclusion rates (FIR) of the interaction terms, as well as 95% CrI distances, CIRs and FIRs across converged L‐L models with all interaction terms within the diseased state with three different priors (Hyplerlasso, Elastic Net, Regularized Horseshoe) fitted to the 500 data sets simulated from L‐L DGM in CPTB data setting with the sample sizes of 5000. Estimates are given with 2.5th and 97.5th percentiles.
**Table D.38** 95% CrI coverages for prevalence, sensitivities, and specificities across converged L‐L models with all interaction terms within the diseased state with three different priors (Hyplerlasso, Elastic Net, Regularized Horseshoe) fitted to the 500 data sets simulated from L‐L DGM in CPTB data setting with the sample sizes of 5000. Coverages are presented as percentages along with 95% Monte Carlo confidence intervals.
**Table D.39** Mean absolute biases of posterior median estimates for prevalence, sensitivities, and specificities with mean residual deviances and DIC values across converged CInd and L‐L models with known correlation structures fitted to the 1,250 data sets simulated from LT DGM in CDPN data setting. Estimates are given with 2.5th and 97.5th percentiles.
**Table D.40** 95% CrI coverages for prevalence, sensitivities, and specificities across converged CInd and L‐L models with known correlation structures fitted to the 1,250 data sets simulated from LT DGM in CDPN data setting. Coverages are presented as percentages along with 95% Monte Carlo confidence intervals.
**Table D.41** Mean absolute biases of posterior median estimates for prevalence, sensitivities, and specificities with mean residual deviances and DIC values across converged CInd and L‐L models with known correlation structures fitted to the 1,250 data sets simulated from FE DGM in CDPN data setting. Estimates are given with 2.5th and 97.5th percentiles.
**Table D.42** 95% CrI coverages for prevalence, sensitivities, and specificities across converged CInd and L‐L models with known correlation structures fitted to the 1,250 data sets simulated from FE DGM in CDPN data setting. Coverages are presented as percentages along with 95% Monte Carlo confidence intervals.
**Table D.43** Mean absolute biases of posterior median estimates for prevalence, sensitivities, and specificities with mean residual deviances and DIC values across converged CInd and L‐L models with known correlation structures fitted to the 1,250 data sets simulated from L‐L DGM in CDPN data setting. Estimates are given with 2.5th and 97.5th percentiles.
**Table D.44** 95% CrI coverages for prevalence, sensitivities, and specificities across converged CInd and L‐L models with known correlation structures fitted to the 1,250 data sets simulated from L‐L DGM in CDPN data setting. Coverages are presented as percentages along with 95% Monte Carlo confidence intervals.
**Table D.45** Mean absolute biases of posterior median estimates for prevalence, sensitivities, and specificities with mean residual deviances, DIC values, CrI widths providing minimum distances, corresponding correct inclusion rates (CIR) and false inclusion rates (FIR) of the interaction terms, as well as 95% CrI distances, CIRs and FIRs across converged L‐L models with all interaction terms within both diseased and disease‐free states with three different priors (Hyplerlasso, Elastic Net, Regularized Horseshoe) fitted to the 500 data sets simulated from L‐L DGM in CDPN data setting with the sample sizes of 500. Estimates are given with 2.5th and 97.5th percentiles.
**Table D.46** 95% CrI coverages for prevalence, sensitivities, and specificities across converged L‐L models with all interaction terms within both diseased and disease‐free states with three different priors (Hyplerlasso, Elastic Net, Regularized Horseshoe) fitted to the 500 data sets simulated from L‐L DGM in CDPN data setting with the sample sizes of 500. Coverages are presented as percentages along with 95% Monte Carlo confidence intervals.
**Table D.47** Mean absolute biases of posterior median estimates for prevalence, sensitivities, and specificities with mean residual deviances, DIC values, CrI widths providing minimum distances, corresponding correct inclusion rates (CIR) and false inclusion rates (FIR) of the interaction terms, as well as 95% CrI distances, CIRs and FIRs across converged L‐L models with all interaction terms within both diseased and disease‐free states with three different priors (Hyplerlasso, Elastic Net, Regularized Horseshoe) fitted to the 500 data sets simulated from L‐L DGM in CDPN data setting with the sample sizes of 2000. Estimates are given with 2.5th and 97.5th percentiles.
**Table D.48** 95% CrI coverages for prevalence, sensitivities, and specificities across converged L‐L models with all interaction terms within both diseased and disease‐free states with three different priors (Hyplerlasso, Elastic Net, Regularized Horseshoe) fitted to the 500 data sets simulated from L‐L DGM in CDPN data setting with the sample sizes of 2000. Coverages are presented as percentages along with 95% Monte Carlo confidence intervals.
**Table D.49** Mean absolute biases of posterior median estimates for prevalence, sensitivities, and specificities with mean residual deviances, DIC values, CrI widths providing minimum distances, corresponding correct inclusion rates (CIR) and false inclusion rates (FIR) of the interaction terms, as well as 95% CrI distances, CIRs and FIRs across converged L‐L models with all interaction terms within both diseased and disease‐free states with three different priors (Hyplerlasso, Elastic Net, Regularized Horseshoe) fitted to the 500 data sets simulated from L‐L DGM in CDPN data setting with the sample sizes of 5000. Estimates are given with 2.5th and 97.5th percentiles.
**Table D.50** 95% CrI coverages for prevalence, sensitivities, and specificities across converged L‐L models with all interaction terms within both diseased and disease‐free states with three different priors (Hyplerlasso, Elastic Net, Regularized Horseshoe) fitted to the 500 data sets simulated from L‐L DGM in CDPN data setting with the sample sizes of 5000. Coverages are presented as percentages along with 95% Monte Carlo confidence intervals.
**Table E.1** 95% CrI coverages for prevalence, sensitivities, and specificities across converged CInd and L‐L models with correct pairwise interactions fitted to the 1,250 data sets simulated from each DGM and L‐L models with regularized horseshoe priors fitted to the 500 data sets simulated from the L‐L DGMs in HIV and VL data settings (sample size = 2,000). Coverages are presented as percentages.
**Table E.2** 95% CrI coverages for prevalence, sensitivities, and specificities across converged CInd and L‐L models with correct pairwise interactions fitted to the 1,250 data sets simulated from each DGM and L‐L models with regularized horseshoe priors fitted to the 500 data sets simulated from the L‐L DGMs in CPTB and CDPN data settings (sample size = 2,000). Coverages are presented as percentages.
**Figure F.1** Absolute biases: means and 2.5th and 97.5th percentiles across models fitted to 1,250 (CInd and L‐L with correct pairwise interactions) or 500 (L‐L with regularized horseshoe (RH) priors) data sets simulated based on the HIV data set. Sample size = 2000.
**Table G.1** Absolute biases: means, along with 2.5th and 97.5th percentiles, across models fitted to 1,250 (CInd and L‐L with correct pairwise interactions) or 500 (L‐L with regularized horseshoe (RH) priors) data sets simulated from L‐L DGMs based on four different data sets with different sample sizes (n). Shading: Blue shading denotes cells whose 95% empirical intervals are entirely positive (indicating overestimation), while orange shading denotes cells whose 95% empirical intervals are entirely negative (indicating underestimation).

Supporting information: 1.

## Data Availability

This study uses simulated data. The simulation scenarios were informed by parameter values reported in previously published diagnostic accuracy studies, which are cited in the manuscript. For reproducibility, all code used to generate the simulated data and to fit the different model types is provided in the Supporting Information.
