# Supplementary material for: Latent Class Log‐Linear Models for Estimating Diagnostic Test Accuracy Without a Gold Standard: A Simulation Study
Source: Stat Med. 2026 Jul 6;45(15-17):e70660. doi: 10.1002/sim.70660 (PMC13334516; doi:10.1002/sim.70660)
Supplement: Supplementary file 1 — Appendix A–G Supporting information. Table B.1 Parameter values for the sensitivities, specificities, slope parameters, and prevalence to simulate data from the latent trait model based on the HIV data. Table B.2 Parameter values for the sensitivities, specificities, covariances, and prevalence to simulate data from the fixed effect model based on the HIV data. Table B.3 Parameter values for the sensitivities, specificities, main effects, interaction, and prevalence to simulate data from the log‐linear model based on the HIV data. Table B.4 Parameter values for the sensitivities, specificities, slope parameters, and prevalence to simulate data from the latent trait model based on the VL data. Table B.5 Parameter values for the sensitivities, specificities, covariances, and prevalence to simulate data from the fixed effect model based on the VL data. Table B.6 Parameter values for the sensitivities, specificities, main effects, interaction, and prevalence to simulate data from the log‐linear model based on the VL data. Table B.7 Parameter values for the sensitivities, specificities, slope parameters, and prevalence to simulate data from the latent trait model based on the CPTB data. Table B.8 Parameter values for the sensitivities, specificities, dependency terms, and prevalence to simulate data from the fixed effect model based on the CPTB data. Table B.9 Parameter values for the sensitivities, specificities, main effects, interaction, and prevalence to simulate data from the log‐linear model based on the CPTB data. Table B.10 Parameter values for the sensitivities, specificities, slope parameters, and prevalence to simulate data from the latent trait model based on the CDPN scenario. Table B.11 Parameter values for the sensitivities, specificities, covariances, and prevalence to simulate data from the fixed effect model based on the CDPN scenario. Table B.12 Parameter values for the sensitivities, specificities, main effects, interaction, and prevalence to simulate [file SIM-45-0-s001.pdf]

Appendix A

The shrinkage priors and hyperparameter specifications used in models that included all pairwise interaction terms (λ_{jl}) within the relevant disease states were as follows:

Hyperlasso (HL): **[1]**

$$\lambda_{jl}|\tau_{jl} \sim \text{Double-Exponential}\left(0, \sqrt{\tau_{jl}}\right)$$

$$\tau_{jl}|\theta^2 \sim \text{Gamma}\left(0.5, \frac{1}{\theta^2}\right)$$

$$\theta \sim \text{half-Cauchy}(0,1)$$

Regularized Horseshoe: **[2]**

$$\lambda_{jl}|\tau_{jl}, \theta \sim \text{Normal}\left(0, \tau_{jl}^2\theta\right), \qquad \tau_{jl}^2 = \frac{c^2\delta_{jl}^2}{c^2 + \theta^2\delta_{jl}^2}$$

$$\theta \sim \text{half-Cauchy}(0,1)$$

$$\delta_{jl}^2 \sim \text{half-Cauchy}(0,1)$$

$$c^2 \sim \text{inverse-Gamma}(2,8)$$

Elastic Net: **[3]**

$$\lambda_{jl}|\tau_{jl}, \theta_2 \sim \text{Normal}\left(0, \frac{\tau_{jl} - 1}{\tau_{jl}\theta_2}\right)$$

$$\tau_{jl}|\theta_1, \theta_2 \sim \text{Gamma}\left(\frac{1}{2}, \frac{8\theta_2}{\theta_1}\right)T(1,) \text{ (truncated)}$$

$$\theta_1 \sim \text{half-Cauchy}(0,1)$$

$$\theta_2 \sim \text{half-Cauchy}(0,1).$$

Detailed information on the properties of these shrinkage priors can be found in the study by van Erp et al. [4].

Appendix B

Data Generating Mechanisms and Fitted Log-Linear (L-L) Models

HIV Data

The original HIV data [5] consists of the results of four diagnostic tests – three different types of radioimmunoassay (ag121, p24, and gp120) and one enzyme-linked immunosorbent assay – applied to 428 subjects. This data set has been used in various studies, proposing different conditional dependence latent class models.

Latent Trait Model

Qu et al. [6] fitted a latent trait model (Eq. 5 in the main text) assuming dependence between the p24 and gp120 radioimmunoassays (Test 2 and Test 3) within the HIV positive subjects. Qu et al. [6] assumed a shared slope parameter for Test 2 and Test 3 within the diseased state ($\beta_{21} = \beta_{31}$). To simulate data from this model, the values of the intercepts and this slope parameter are needed. However, only estimates of the sensitivities and specificities were provided by Qu et al. [6]. In a latent trait model the mean sensitivity and specificity estimates of Test i across subjects are given by [6]

$$Se_j = \Phi\left(\frac{\alpha_{j1}}{\sqrt{1+\beta_{j1}^2}}\right)$$

$$Sp_j = \Phi\left(\frac{-\alpha_{j0}}{\sqrt{1+\beta_{j0}^2}}\right). \qquad \textit{Eq. B. 1}$$

Although the reported sensitivities and specificities could be used to infer intercept parameters for the conditionally independent tests, the values of the slope parameter and prevalence are still needed. Therefore, we first fitted the same latent trait model – i.e., the model incorporating $\beta_{21} = \beta_{31}$ parameters and assuming $\beta_{j0} = \beta_{11} = \beta_{41} = 0$ for $j = 1,2,3,4$ – to the original data set to obtain estimates of the slope parameters and prevalence (π), and then we used parameter estimates obtained from this fitted model to simulate data. As our main interest in this study is the accuracy estimates, we

fixed the accuracy estimates and slope parameter and then used intercepts that were calculated based on Eq. B. 1. The final parameter estimates that we used to simulate data similar to the HIV data from a latent trait model are presented in Table B. 1.

Table B. 1 Parameter values for the sensitivities, specificities, slope parameters, and prevalence to simulate data from the latent trait model based on the HIV data

Parameter	Test 1	Test 2	Test 3	Test 4
$\boldsymbol{Se_j}$	0.997	0.569	0.908	0.996
$\boldsymbol{Sp_j}$	0.970	0.962	0.997	0.918
$\boldsymbol{\beta_{j1}}$	0	1.050	1.050	0
$\boldsymbol{\beta_{j0}}$	0	0	0	0
$\boldsymbol{\pi}$	0.542			

The L-L model that we fitted to data simulated from this model was the model incorporating an interaction term for the pair Test 2 x Test 3 within the diseased state ($\lambda_{23|d=1}$) assuming conditional independence within the disease-free state and between the other pairs within the diseased state.

Fixed Effect Model

Wang et al. [7] fitted a fixed effect model defined by Jones et al. [8] (Parameterisation 1 / Eq. 6 in the main text) to the HIV data set, assuming conditional dependence only between Test 2 and Test 3, but within both disease states. The estimates for all necessary parameters to simulate data were provided by Wang et al. [7] and are shown in Table B. 2. These parameter estimates were adopted without any modification.

Table B. 2 Parameter values for the sensitivities, specificities, covariances, and prevalence to simulate data from the fixed effect model based on the HIV data

Parameter	Test 1	Test 2	Test 3	Test 4
$\boldsymbol{Se_j}$	0.997	0.571	0.908	0.996
$\boldsymbol{Sp_j}$	0.972	0.961	0.995	0.925
$\boldsymbol{covse_{23}}$	0.032			
$\boldsymbol{covsp_{23}}$	0.002			
$\boldsymbol{\pi}$	0.542			

The L-L model that we fitted to data simulated from this fixed-effect model included a pairwise interaction term for the Test 2 x Test 3 pair within each disease state ($\lambda_{23|d=0}$ and $\lambda_{23|d=1}$) to more closely resemble the fixed-effect model from which data were simulated.

Log-Linear Model

Sepulveda et al. [9] proposed a L-L model (Eq. 4 in the main text) incorporating a pairwise interaction for the same pair (Test 2 x Test 3), but only within the diseased state ($\lambda_{23|d=1}$) for the HIV data set. They reported estimates of all sensitivities, specificities and prevalence, but estimates of the main effects and the interaction between Test 2 and Test 3 within the diseased state are also needed to simulate data from this model. Therefore, we employed a similar strategy to that described above for the latent trait data-generating mechanism: we fitted a L-L model including only the $\lambda_{23|d=1}$ interaction to the original HIV data set and used the parameter estimates for the main effects and the interaction obtained (Table B. 3) to simulate data.

Table B. 3 Parameter values for the sensitivities, specificities, main effects, interaction, and prevalence to simulate data from the log-linear model based on the HIV data

Parameter	Test 1	Test 2	Test 3	Test 4
Se_j	0.997	0.573	0.910	0.996
Sp_j	0.969	0.962	0.996	0.918
$\lambda_{j\|d=1}$	5.742	-0.981	1.711	5.623
$\lambda_{j\|d=0}$	-3.456	-3.227	-5.641	-2.417
$\lambda_{23\|d=1}$	1.398			
π	0.541			

The L-L model that we fitted to data simulated from this model was the same as the model simulated from (which is also the same that we fitted to the data sets simulated from the latent trait DGM).

Visceral Leishmaniasis (VL) Data

The original VL data [10] comprises the results of four diagnostic tests (Test 1:DAT, Test 2: rk39, Test 3: KAtex, Test 4: Parasitology) for 291 subjects with VL symptoms. Menten et al. [11] fitted latent trait and fixed effect models with different correlation structures to the data set. They considered their correlation structure #5 to be the most plausible based on expert opinion: this assumes conditional independence within the disease-free subjects, but correlation between Test 1 and Test 2 and between Test 3 and Test 4 within the diseased group [11].

Latent Trait Model

Instead of a probit link (Eq. 5 in the main text), Menten et al. [11] used a logit link to fit the latent trait model and provided parameter estimates for the prevalence, sensitivities, specificities, and the slope

parameters. However, we could not derive the intercepts for the logit link model from this information because the equations for the mean sensitivities and specificities (Eq. B. 1) do not hold for the logit link. Instead, we simulated data from a latent trait model employing a probit link (Eq. 5 in the main text) with the same parameter values provided by Menten et al. [11] (Table B. 4) to calculate intercepts using the equations in Eq. B. 1.

Table B. 4 Parameter values for the sensitivities, specificities, slope parameters, and prevalence to simulate data from the latent trait model based on the VL data

Parameter	Test 1	Test 2	Test 3	Test 4
Se_j	0.849	0.782	0.693	0.712
Sp_j	0.977	0.918	0.981	0.985
β_{j1}	0.440	0.440	4.360	4.360
β_{j0}	0	0	0	0
π	0.372			

Menten et al. [11] assumed shared slope parameters for Test 1 and Test 2 ($\beta_{11} = \beta_{21}$), and another shared slope parameter for Test 3 and Test 4 ($\beta_{31} = \beta_{41}$), while assuming independence within the disease free state ($\beta_{j0} = 0$ for $j = 1,2,3,4$). Note that this model does not only imply correlations between Tests 1 and 2 and between Tests 3 and 4 within the diseased state: conditional dependence between all pairs is additionally implied. The L-L model that we fitted to data simulated from this model therefore incorporated interaction terms for all six pairs within the diseased state.

Polychoric correlations between jth test and lth test in a latent trait model can be calculated using the following formula [12]:

$$\rho_{jld_i} = \frac{\beta_{jd_i}\beta_{ld_i}}{\sqrt{1+\beta_{jd_i}^2}\sqrt{1+\beta_{ld_i}^2}}\ , d_i = 0,1\ ; j,l = 1,2,\dots,J; j < l. \qquad \textit{Eq. B. 2}$$

Hence, this latent trait model (Table B. 4) implies the same polychoric correlations for the pairs Test 1 x Test 3, Test 1 x Test 4, Test 2 x Test 3, and Test 2 x Test 4 within the diseased state (i.e., $\rho_{131} = \rho_{141} = \rho_{231} = \rho_{241} = 0.393$).

Although interaction terms in a log-linear model represent log odds ratios for test pairs, and shared polychoric correlations might not necessarily imply shared log odds ratios, we assumed shared

interaction terms for the pairs with shared polychoric correlations while specifying the L-L model to be fitted to each simulated data set. This was considered a pragmatic approach since assuming shared interaction terms for pairs with similar correlations reduces the number of parameters to be estimated, potentially avoiding identifiability issues and improving convergence. Hence, we fitted an L-L model (Eq. 4 in the main text) incorporating all pairwise interactions within the diseased state and assuming $\lambda_{13|d=1} = \lambda_{14|d=1} = \lambda_{23|d=1} = \lambda_{24|d=1}$ to data sets simulated from the latent trait data-generating mechanism based on the VL data.

Fixed Effect Model

Menten et al. [11] also fitted a (different type of) fixed effect model to the VL data (Parameterisation 2 / Eq. 7 in the main text). They included covariances between Test 1 and Test 2 ($covse_{12}$) and between Test 3 and Test 4 ($covse_{34}$) within the diseased state. They assumed conditional independence for the other pairs within the diseased state and for all pairs within the disease-free state. Estimates for all necessary parameters to simulate data from this model were provided by Menten et al. [11]. However, we found the reported parameter estimates to yield negative probabilities of some combinations of test results within the diseased group, potentially indicating that necessary constraints on the covariance terms had not been enforced. Hence, we fitted the same model to the original VL data set, enforcing the constraints given by Dendukuri and Joseph [14], and obtained the following parameter estimates (Table B. 5), which we used to simulate data from this model.

Table B. 5 Parameter values for the sensitivities, specificities, covariances, and prevalence to simulate data from the fixed effect model based on the VL data

Parameter	Test 1	Test 2	Test 3	Test 4
$\boldsymbol{Se_j}$	0.857	0.779	0.729	0.749
$\boldsymbol{Sp_j}$	0.982	0.918	0.981	0.985
$\boldsymbol{covse_{12}}$	-0.009			
$\boldsymbol{covse_{34}}$	0.125			
$\boldsymbol{\pi}$	0.370			

The L-L model (Eq. 4 in the main text) that we fitted to data simulated from this model incorporated pairwise interactions between Tests 1 and 2 ($\lambda_{12|d=1}$), as well as between Tests 3 and 4 ($\lambda_{34|d=1}$) within the diseased state.

Log-Linear Model

Since we did not encounter a study fitting L-L models to the VL data set in the literature, we fitted an L-L model including pairwise interactions between Tests 1 and 2 ($\lambda_{12|d=1}$), as well as between Tests 3 and 4 ($\lambda_{34|d=1}$) within the diseased state – i.e., the same model fitted to the data sets simulated from the fixed-effect DGM – to the original VL data, obtaining the parameter settings provided in Table B. 6, which we simulated from.

Table B. 6 Parameter values for the sensitivities, specificities, main effects, interaction, and prevalence to simulate data from the log-linear model based on the VL data

Parameter	Test 1	Test 2	Test 3	Test 4
Se_j	0.857	0.779	0.730	0.750
Sp_j	0.982	0.918	0.981	0.985
$\lambda_{j\|d=1}$	2.140	1.637	-1.176	-0.883
$\lambda_{j\|d=0}$	-3.997	-2.412	-3.943	-4.182
$\lambda_{12\|d=1}$	-0.435			
$\lambda_{34\|d=1}$	3.316			
π	0.370			

The L-L model that we fitted to data simulated from this model was the correct model, i.e. the same as that simulated from (also the same model fitted to data simulated from the fixed-effect DGM).

Childhood Pulmonary Tuberculosis (CPTB) Data

The original CPTB data set [15, 16] contains results of 5 diagnostic tests for CPTB (3 microbiological tests, tuberculin skin test, and chest radiography) on 749 children who were suspected to be infected.

Latent Trait Model

Wang et al. [17] fitted a latent trait model to this data set, in their study where they explored the performance of a type of fixed effect model (see next section). Based on expert opinion, they assumed conditional dependence for the three microbiological tests and skin test among the diseased subjects and that radiography was conditionally independent of the other four tests. The random effect component of the latent trait model was considered to represent the unmeasured bacillary burden of the subjects. In the latent trait model fitted by Wang et al. [17], shared slope parameters for three microbiological tests were assumed, and an additional quadratic function of the random effect was incorporated for the skin test. In this study, we chose to simulate from a slightly simplified model that

incorporated the shared slope parameters assumption but excluded the quadratic term for the skin test, as the dependency implied by this quadratic random effect could not be easily translated into log odds ratios in the L-L models to be fitted. Thus, we fitted a latent trait model (Eq. 5 in the main text) incorporating slope parameters for the microbiological tests and the skin test within the diseased state ($\beta_{11}, \beta_{21}, \beta_{31}, \beta_{41}$) and assuming shared slope parameters across microbiological tests ($\beta_{11} = \beta_{21} = \beta_{31}$) to the original CPTB data. We obtained the following parameter estimates (Table B. 7), which we used to simulate data.

Table B. 7 Parameter values for the sensitivities, specificities, slope parameters, and prevalence to simulate data from the latent trait model based on the CPTB data

Parameter	Test 1	Test 2	Test 3	Test 4	Test 5
$\boldsymbol{Se_j}$	0.564	0.461	0.201	0.686	0.646
$\boldsymbol{Sp_j}$	0.999	0.989	0.997	0.678	0.797
$\boldsymbol{\beta_{j1}}$	2.804	2.804	2.804	0.023	0
$\boldsymbol{\beta_{j0}}$	0	0	0	0	0
$\boldsymbol{\pi}$	0.292				

Shared slope parameters for Test 1, Test 2, and Test 3 within the diseased state implies shared polychoric correlations (Eq. B. 2) for the pairs Test 1 x Test 2, Test 1 x Test 3, and Test 2 x Test 3 ($\rho_{121} = \rho_{131} = \rho_{231} = 0.887$). Additionally, these slope parameters imply identical polychoric correlations for all test pairs including the microbiological tests and skin test ($\rho_{141} = \rho_{241} = \rho_{341} = 0.021$). Therefore, following the same strategy as described above for the VL setting, we made the pragmatic decision to assume shared corresponding pairwise interaction terms in the L-L model. Hence, we fitted an L-L model (Eq. 4 in the main text) that included all pairwise interactions – excluding those involving Test 5 – within the diseased state, and assumed $\lambda_{12|1} = \lambda_{13|1} = \lambda_{23|1}$ and $\lambda_{14|1} = \lambda_{24|1} = \lambda_{34|1}$.

Fixed Effect Model

Wang et al. [17] defined a different fixed effect model (Parameterisation 3 / Eq. 8 in the main text) and applied this model to the CPTB data set. Differently from the other fixed effect models, they suggested adding higher order dependency terms – measuring the difference between joint probabilities under conditional dependence and independence assumptions – rather than pairwise covariance terms. To reflect expert opinion, Tests 1–4 were assumed to be correlated within the diseased state, while Test 5

was considered independent of the others. Estimates for all necessary parameters – given by Wang et al. [17] – are provided in Table B. 8 and were used to simulate data from this model.

Table B. 8 Parameter values for the sensitivities, specificities, dependency terms, and prevalence to simulate data from the fixed effect model based on the CPTB data

Parameter	Test 1	Test 2	Test 3	Test 4	Test 5
Se_j	0.689	0.572	0.267	0.707	0.647
Sp_j	0.997	0.988	1.000	0.659	0.760
$\delta_{1111\|1}$	-0.005				
$\delta_{0111\|1}$	-0.027				
$\delta_{1011\|1}$	-0.049				
$\delta_{1101\|1}$	0.039				
$\delta_{1110\|1}$	0.127				
$\delta_{0011\|1}$	-0.016				
$\delta_{0101\|1}$	-0.057				
$\delta_{0110\|1}$	-0.009				
$\delta_{1001\|1}$	0.000				
$\delta_{1010\|1}$	-0.017				
$\delta_{1100\|1}$	-0.052				
$\delta_{0001\|1}$	0.115				
$\delta_{0010\|1}$	-0.006				
$\delta_{0100\|1}$	-0.018				
$\delta_{1000\|1}$	-0.043				
$\delta_{0000\|1}$	0.008				
π	0.227				

The L-L model that we fitted to data simulated from this model included the same pairwise interaction terms as that fitted to the data sets simulated from the latent trait model. However, we did not assume any shared interactions in this case since such an assumption was not made by Wang et al. [17] when fitting the fixed effect model.

Log-Linear Model

We did not encounter a study that applied L-L models to the CPTB data in literature. We therefore fitted an L-L model to the CPTB data set and simulated data based on the parameter estimates from this. We fitted the L-L model described in the fixed effect model selection above, i.e. including pairwise interactions for all pairs including Test 1, Test 2, Test 3, and Test 4 within the diseased state, but not assuming shared interactions for any pairs. The parameter values used to simulate from the L-L model for the CPTB data are presented in Table B. 9.

Table B. 9 Parameter values for the sensitivities, specificities, main effects, interaction, and prevalence to simulate data from the log-linear model based on the CPTB data

Parameter	Test 1	Test 2	Test 3	Test 4	Test 5
Se_j	0.689	0.572	0.274	0.765	0.648
Sp_j	0.997	0.988	1.000	0.659	0.760
$\lambda_{j\|d=1}$	0.440	-1.113	-0.655	3.350	0.610
$\lambda_{j\|d=0}$	-5.850	-4.410	-8.394	-0.660	-1.154
$\lambda_{12\|d=1}$	2.193				
$\lambda_{13\|d=1}$	0.232				
$\lambda_{14\|d=1}$	-0.848				
$\lambda_{23\|d=1}$	1.865				
$\lambda_{24\|d=1}$	-0.598				
$\lambda_{34\|d=1}$	-2.656				
π	0.227				

The L-L model fitted to data simulated from this model was the correct model (i.e. with no assumption of shared interaction terms).

Keddie's Conditional Dependence in Positives and Negatives (CDPN) Data

Latent Trait Model

Keddie et al. [18] adopted latent trait models (Eq. 5 in the main text) to simulate data sets corresponding to the results of five diagnostic tests, reflecting characteristics of four serological tests (Test 1, Test 2, Test 3 and Test 4) and one culture test (Test 5) used in diagnosis of Melioidosis. They simulated data under four different assumptions: i) conditional independence, ii) conditional dependence in disease positive individuals, iii) conditional dependence in disease negative individuals, iv) conditional dependence in both disease positive and negative individuals. Since studies assuming conditional dependence within disease-free subjects are scarce in the literature, and the other three conditional dependence scenarios explored in our study assumed conditional dependence only within diseased subjects, we adopted the (iv) conditional dependence in disease positive and negative subjects (CDPN) scenario to simulate data. This is referred to as the "CDPN data set" in our manuscript, although is not strictly based on a specific data set. Keddie et al. [18] assumed shared sensitivities for all tests, and shared specificities for the serological tests (Tests 1–4). Shared slope parameters were assumed across the serological tests in both states ($\beta_{jd_i} = 1$ for $j = 1,2,3,4; d_i = 0,1$), while Test 5 was assumed to be independent of the serological tests. We used the same model [18] with the same parameter values (Table B. 10) to simulate data.

Table B. 10 Parameter values for the sensitivities, specificities, slope parameters, and prevalence to simulate data from the latent trait model based on the CDPN scenario

Parameter	Test 1	Test 2	Test 3	Test 4	Test 5
$\boldsymbol{Se_j}$	0.650	0.650	0.650	0.650	0.650
$\boldsymbol{Sp_j}$	0.900	0.900	0.900	0.900	0.990
$\boldsymbol{\beta_{j1}}$	1.000	1.000	1.000	1.000	0
$\boldsymbol{\beta_{j0}}$	1.000	1.000	1.000	1.000	0
$\boldsymbol{\pi}$	0.500				

Since all slope parameters were set to 1 (following Keddie et al. [18]), all polychoric correlations (Eq. B. 2) between tests 1-4 within each disease state were identical and equal to 0.5. Therefore, following the reasoning described in previous sections of this appendix, the L-L model (Eq. 4 in the main text) that we fitted to data simulated from this model included a single shared interaction for all pairs including Tests 1-4 within both disease states ($\lambda_{12|d} = \lambda_{13|d} = \lambda_{14|d} = \lambda_{23|d} = \lambda_{24|d} = \lambda_{34|d}$ for $d = 0{,}1$).

Fixed Effect Model

Keddie et al. [18] only described latent trait models for the CDPN dependency structure. Therefore, we fabricated a fixed effect model with the same sensitivities, specificities and prevalence (Table B. 11), and with covariance terms that produced similar odds ratios for the test pairs to those generated by the latent trait model. We used a fixed-effect model with the same parameterisation as in the HIV setting (Parameterisation 1 / Eq. 6 in the main text). This type of parameterisation was proposed by Jones et al. [8], and their study can be referred to for the general form of the model. We initially assumed a single shared covariance term for all pairs involving Tests 1–4 within each disease state, resulting in a shared Pearson correlation across these pairs and across both states. However, unlike polychoric correlations (Eq. B. 2), Pearson correlations depend on the sensitivities in the diseased group and specificities in the disease-free group. Since the assumed values of sensitivities (0.650) and specificities (0.900) were very different from each other [18], this approach produced substantially different log odds ratios from those obtained from the latent trait model. Therefore, we assumed shared covariance terms within each disease state but different across different states (one for the diseased group: $covse_{12} = covse_{13} = covse_{14} = covse_{23} = covse_{24} = covse_{34} = covse$, and one for the disease-free group: $covsp_{12} = covsp_{13} = covsp_{14} = covsp_{23} = covsp_{24} = covsp_{34} = covsp$), to fabricate a scenario more similar

to the original latent trait model [18]. The parameter values used to simulate data from this model are provided in Table B. 11.

Table B. 11 Parameter values for the sensitivities, specificities, covariances, and prevalence to simulate data from the fixed effect model based on the CDPN scenario

Parameter	Test 1	Test 2	Test 3	Test 4	Test 5
Se_j	0.650	0.650	0.650	0.650	0.650
Sp_j	0.900	0.900	0.900	0.900	0.990
$covse$	0.075				
$covsp$	0.015				
π	0.500				

The L-L model that we fitted to data simulated from this model assumed one shared interaction parameter in the diseased group ($\lambda_{12|d=1} = \lambda_{13|d=1} = \lambda_{14|d=1} = \lambda_{23|d=1} = \lambda_{24|d=1} = \lambda_{34|d=1}$), and one shared interaction parameter in the disease-free group ($\lambda_{12|d=0} = \lambda_{13|d=0} = \lambda_{14|d=0} = \lambda_{23|d=0} = \lambda_{24|d=0} = \lambda_{34|d=0}$).

Log-Linear Model

The L-L model that we simulated from included all pairwise interactions for the correlated tests (Tests 1-4) within both disease states. Since we found that the original (latent trait) data generating mechanism for the CDPN data implied very similar odds ratios across tests pairs and also across disease states, we simulated from a L-L model with a single shared interaction term.

The value of this interaction parameter was set to produce similar odds ratios for correlated pairs as those obtained from the latent trait model. Prevalence was set to 0.5, as with the other data generating mechanisms for this data set, and main effect parameters were set to produce the same sensitivities and specificities as in Table B. 10. The full set of parameter values assumed is presented in Table B. 12.

Table B. 12 Parameter values for the sensitivities, specificities, main effects, interaction, and prevalence to simulate data from the log-linear model based on the CPTB data

Parameter	Test 1	Test 2	Test 3	Test 4	Test 5
Se_j	0.650	0.650	0.650	0.650	0.650
Sp_j	0.900	0.900	0.900	0.900	0.990
$\lambda_{j\mid d=1}$	-1.035	-1.035	-1.035	-1.035	0.620
$\lambda_{j\mid d=0}$	-2.595	-2.595	-2.595	-2.595	-4.600
$\lambda_{jl\mid d=1}$, $j = 1,2,3$; $l = i, \dots, 4$	0.900				
$\lambda_{jl\mid d=0}$, $j = 1,2,3$; $l = i, \dots, 4$	0.900				
π	0.500				

The log-linear model that we fitted to data simulated from this model was the correct true model (i.e., the model assuming shared interactions across test pairs and across both disease states; $\lambda_{12|d} = \lambda_{13|d} = \lambda_{14|d} = \lambda_{23|d} = \lambda_{24|d} = \lambda_{34|d}$ for $d = 0,1$).

Appendix C

To determine the number of simulations (n_{sim}) for each of the 36 scenarios, 100 data sets were initially simulated from the 12 data generating mechanisms (i.e., four data settings: i) HIV, ii) VL, iii) CPTB, iv) CDPN combined with three types of models: i) latent trait, ii) fixed effect, iii) log-linear), with sample sizes of 500. We then fitted L-L models incorporating the 'correct' pairwise interaction terms for each data generating mechanism (as specified in Appendix B) to these data sets. The empirical variances of posterior median prevalence, sensitivity and specificity across these 100 data sets were calculated. The highest empirical variance across these data sets was 0.0076, for the sensitivity of Test 3 (Se_3) for the latent trait model in the CPTB setting. Using the Monte Carlo Standard Error (MCSE) formula for bias given by Morris et al. [19], the number of required (n_{req}) simulated data sets for an MCSE of at most 0.0025 can be calculated from

$$0.0025 \leq \sqrt{\frac{0.0076}{n_{req}}},$$

i.e., $n_{req} \geq 1216$. This implies that we require at least 1216 simulated data sets to obtain a maximum MCSE of 0.0025. See Figure C. 1 for the required number of simulations for different MCSEs. We considered an MCSE of 0.0025 as acceptable since sensitivity and specificity estimates are typically reported as percentages, rounded to two decimal places, and an MCSE error of 0.0025 would not result in substantial differences in reported parameter estimates across simulated data sets. We therefore set n_{sim} to 1250.

Note that this calculation excludes the fitted L-L models with shrinkage priors, as conducting more than 500 simulations was not feasible, even using the University of Bristol's High Performance Computing systems.

Figure C. 1 Required number of simulations (n_{req}) for each maximum value of Monte Carlo Standard Error (MCSE) of biases, based on the maximum empirical variance estimate from the initial 100 simulation runs.

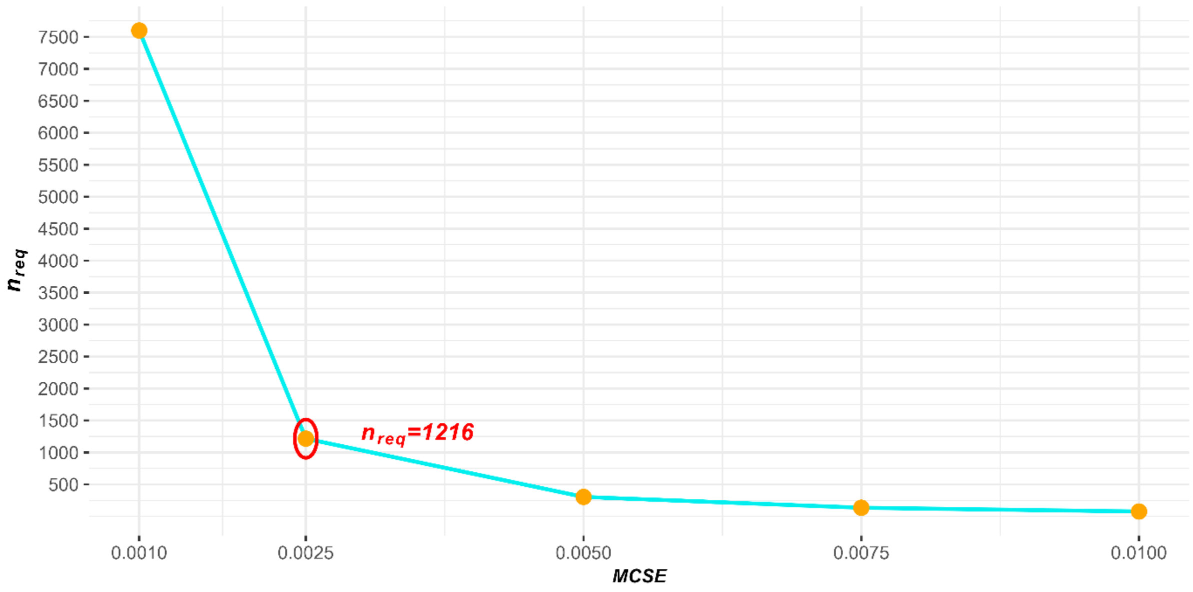

Appendix D

Table D. 1 Number of converged CInd and L-L models with known correlation structure fitted to the 1,250 data sets simulated from three different DGMs (LT, FE, and L-L) in four different data settings (HIV, VL, CPTB, and CDPN) for different sample sizes. The percentages of converged models in each scenario are also presented in brackets.

		HIV		VL		CPTB		CDPN	
**DGM**	**Sample Size**	**CInd**	**L-L**	**CInd**	**L-L**	**CInd**	**L-L**	**CInd**	**L-L**
LT	500	1250 (100.0)	1250 (100.0)	1250 (100.0)	1226 (98.1)	1250 (100.0)	1230 (98.4)	1250 (100.0)	1248 (99.8)
	2000	1250 (100.0)	1250 (100.0)	1250 (100.0)	1126 (90.1)	1250 (100.0)	1131 (90.5)	1250 (100.0)	1248 (99.8)
	5000	1250 (100.0)	1250 (100.0)	1250 (100.0)	1126 (90.1)	1250 (100.0)	1157 (92.6)	1250 (100.0)	1239 (99.1)
FE	500	1250 (100.0)	1250 (100.0)	1250 (100.0)	1250 (100.0)	1250 (100.0)	1250 (100.0)	1250 (100.0)	1244 (99.5)
	2000	1250 (100.0)	1250 (100.0)	1250 (100.0)	1250 (100.0)	1250 (100.0)	1214 (97.1)	1250 (100.0)	1131 (90.5)
	5000	1250 (100.0)	1250 (100.0)	1250 (100.0)	1250 (100.0)	1250 (100.0)	1193 (95.4)	1250 (100.0)	1199 (95.9)
L-L	500	1250 (100.0)	1250 (100.0)	1250 (100.0)	1250 (100.0)	1250 (100.0)	1250 (100.0)	1250 (100.0)	1250 (100.0)
	2000	1250 (100.0)	1250 (100.0)	1250 (100.0)	1250 (100.0)	1250 (100.0)	1227 (98.2)	1250 (100.0)	1233 (98.6)
	5000	1250 (100.0)	1250 (100.0)	1250 (100.0)	1250 (100.0)	1250 (100.0)	1228 (98.2)	1250 (100.0)	1212 (97.0)

Table D. 2 Number of converged L-L models with three different shrinkage priors (HL,EN,RH) fitted to the 500 data sets simulated from L-L DGMs in four different data settings (HIV, VL, CPTB, and CDPN) for different sample sizes. The percentages of converged models in each scenario are also presented in brackets.

	HIV			VL			CPTB			CDPN		
**Sample Size**	**HL**	**EN**	**RH**	**HL**	**EN**	**RH**	**HL**	**EN**	**RH**	**HL**	**EN**	**RH**
500	499 (99.8)	494 (98.8)	497 (99.4)	500 (100.0)	499 (99.8)	500 (100.0)	499 (99.8)	498 (99.6)	500 (100.0)	496 (99.2)	444 (88.8)	492 (98.4)
2000	500 (100.0)	499 (99.8)	498 (99.6)	500 (100.0)	499 (99.8)	500 (100.0)	500 (100.0)	498 (99.6)	500 (100.0)	446 (89.2)	417 (83.4)	439 (87.8)
5000	479 (95.8)	495 (99.0)	487 (97.4)	500 (100.0)	496 (99.2)	498 (99.6)	500 (100.0)	500 (100.0)	500 (100.0)	296 (59.2)	217 (43.4)	264 (52.8)

Table D. 3 Mean absolute biases of posterior median estimates for prevalence, sensitivities, and specificities with mean residual deviances and DIC values across converged CInd and L-L models with known correlation structures fitted to the 1,250 data sets simulated from LT DGM in HIV data setting. Estimates are given with 2.5th and 97.5th percentiles.

	Sample Size = 500		Sample Size = 2000		Sample Size = 5000	
**Parameter**	**CInd**	**L-L**	**CInd**	**L-L**	**CInd**	**L-L**
π	-0.001 (-0.046,0.039)	0.000 (-0.045,0.040)	-0.001 (-0.024,0.020)	0.000 (-0.023,0.021)	-0.001 (-0.015,0.012)	0.000 (-0.013,0.013)
Se_1	-0.003 (-0.011,0.000)	-0.003 (-0.012,0.000)	-0.001 (-0.005,0.002)	-0.001 (-0.005,0.002)	0.000 (-0.003,0.002)	0.000 (-0.003,0.002)
Se_2	0.001 (-0.055,0.062)	0.002 (-0.055,0.063)	0.001 (-0.028,0.031)	0.000 (-0.029,0.030)	0.001 (-0.018,0.020)	0.000 (-0.019,0.019)
Se_3	0.000 (-0.037,0.034)	-0.002 (-0.038,0.031)	0.002 (-0.016,0.020)	0.000 (-0.019,0.017)	0.002 (-0.010,0.013)	0.000 (-0.012,0.011)
Se_4	-0.003 (-0.011,0.001)	-0.003 (-0.011,0.001)	0.000 (-0.005,0.003)	-0.001 (-0.005,0.003)	0.000 (-0.003,0.002)	0.000 (-0.003,0.002)
Sp_1	-0.007 (-0.034,0.017)	-0.004 (-0.029,0.018)	-0.004 (-0.017,0.009)	-0.001 (-0.014,0.010)	-0.003 (-0.011,0.005)	0.000 (-0.008,0.007)
Sp_2	-0.002 (-0.029,0.021)	-0.003 (-0.030,0.021)	0.000 (-0.014,0.012)	-0.001 (-0.014,0.011)	0.000 (-0.008,0.008)	0.000 (-0.008,0.008)
Sp_3	-0.003 (-0.013,0.000)	-0.003 (-0.013,0.000)	-0.001 (-0.005,0.002)	-0.001 (-0.005,0.002)	0.000 (-0.003,0.002)	0.000 (-0.003,0.002)
Sp_4	-0.006 (-0.049,0.031)	-0.003 (-0.045,0.032)	-0.003 (-0.022,0.015)	-0.001 (-0.020,0.016)	-0.003 (-0.015,0.009)	-0.001 (-0.012,0.011)
**ResD**	31.73 (19.04,49.50)	15.97 (13.00,21.27)	79.42 (51.80,113.22)	15.81 (11.35,22.55)	175.4 (129.13,229.87)	15.38 (11.34,23.33)
**DIC**	42.47 (29.62,60.49)	28.41 (24.36,34.26)	88.91 (61.25,123.01)	26.63 (21.98,33.29)	184.55 (138.21,238.9)	25.67 (21.64,33.71)

Table D. 4 95% CrI coverages for prevalence, sensitivities, and specificities across converged CInd and L-L models with known correlation structures fitted to the 1,250 data sets simulated from LT DGM in HIV data setting. Coverages are presented as percentages along with 95% Monte Carlo confidence intervals.

	Sample Size = 500		Sample Size = 2000		Sample Size = 5000	
**Parameter**	**CInd**	**L-L**	**CInd**	**L-L**	**CInd**	**L-L**
π	95.4 (94.2,96.5)	95.6 (94.5,96.7)	94.6 (93.4,95.9)	94.8 (93.6,96.0)	94.8 (93.6,96.0)	96.0 (94.9,97.1)
Se_1	95.2 (94.0,96.4)	95.2 (94.0,96.4)	95.4 (94.2,96.5)	95.5 (94.4,96.7)	94.5 (93.2,95.7)	94.4 (93.1,95.7)
Se_2	95.1 (93.9,96.3)	95.1 (93.9,96.3)	95.2 (94.0,96.4)	95.2 (94.0,96.4)	94.1 (92.8,95.4)	94.8 (93.6,96.0)
Se_3	95.2 (94.0,96.4)	95.0 (93.8,96.2)	94.4 (93.1,95.7)	94.5 (93.2,95.7)	93.4 (92.0,94.7)	94.1 (92.8,95.4)
Se_4	95.0 (93.8,96.2)	94.5 (93.2,95.7)	95.8 (94.7,96.9)	94.2 (92.9,95.5)	94.5 (93.2,95.7)	94.7 (93.5,96.0)
Sp_1	92.8 (91.4,94.2)	95.4 (94.2,96.5)	90.8 (89.2,92.4)	94.7 (93.5,96.0)	88.5 (86.7,90.2)	95.5 (94.4,96.7)
Sp_2	94.9 (93.7,96.1)	94.7 (93.5,96.0)	94.2 (92.9,95.5)	94.2 (92.9,95.5)	94.4 (93.1,95.7)	94.4 (93.1,95.7)
Sp_3	95.1 (93.9,96.3)	94.8 (93.6,96.0)	96.1 (95.0,97.2)	95.9 (94.8,97.0)	95.1 (93.9,96.3)	95.2 (94.0,96.4)
Sp_4	93.3 (91.9,94.7)	94.1 (92.8,95.4)	93.4 (92.1,94.8)	95.5 (94.4,96.7)	92.2 (90.7,93.7)	95.1 (93.9,96.3)

Table D. 5 Mean absolute biases of posterior median estimates for prevalence, sensitivities, and specificities with mean residual deviances and DIC values across converged CInd and L-L models with known correlation structures fitted to the 1,250 data sets simulated from FE DGM in HIV data setting. Estimates are given with 2.5th and 97.5th percentiles.

	Sample Size = 500		Sample Size = 2000		Sample Size = 5000	
**Parameter**	**CInd**	**L-L**	**CInd**	**L-L**	**CInd**	**L-L**
$\boldsymbol{\pi}$	-0.002 (-0.045,0.042)	-0.001 (-0.044,0.043)	-0.001 (-0.023,0.021)	0.000 (-0.022,0.022)	-0.001 (-0.014,0.013)	0.000 (-0.013,0.014)
$\boldsymbol{Se_1}$	-0.003 (-0.011,0.000)	-0.003 (-0.011,0.000)	-0.001 (-0.005,0.002)	-0.001 (-0.005,0.002)	0.000 (-0.003,0.002)	0.000 (-0.003,0.002)
$\boldsymbol{Se_2}$	0.003 (-0.054,0.058)	0.003 (-0.053,0.058)	0.001 (-0.028,0.031)	0.001 (-0.029,0.030)	0.001 (-0.018,0.021)	0.000 (-0.019,0.020)
$\boldsymbol{Se_3}$	0.000 (-0.034,0.035)	-0.001 (-0.036,0.034)	0.001 (-0.017,0.019)	0.000 (-0.018,0.017)	0.002 (-0.010,0.013)	0.000 (-0.012,0.011)
$\boldsymbol{Se_4}$	-0.003 (-0.012,0.001)	-0.003 (-0.012,0.001)	-0.001 (-0.005,0.003)	-0.001 (-0.005,0.003)	0.000 (-0.003,0.002)	0.000 (-0.003,0.002)
$\boldsymbol{Sp_1}$	-0.005 (-0.031,0.015)	-0.003 (-0.027,0.016)	-0.003 (-0.015,0.009)	-0.001 (-0.013,0.010)	-0.002 (-0.010,0.005)	0.000 (-0.008,0.007)
$\boldsymbol{Sp_2}$	-0.002 (-0.027,0.022)	-0.002 (-0.028,0.022)	0.000 (-0.013,0.013)	-0.001 (-0.014,0.013)	0.000 (-0.008,0.008)	0.000 (-0.008,0.007)
$\boldsymbol{Sp_3}$	-0.003 (-0.013,0.002)	-0.003 (-0.014,0.002)	-0.001 (-0.006,0.003)	-0.001 (-0.006,0.003)	0.000 (-0.003,0.003)	0.000 (-0.003,0.003)
$\boldsymbol{Sp_4}$	-0.004 (-0.043,0.027)	-0.002 (-0.040,0.029)	-0.003 (-0.021,0.015)	-0.001 (-0.018,0.016)	-0.002 (-0.014,0.009)	0.000 (-0.012,0.011)
**ResD**	30.80 (17.82,50.40)	17.02 (13.08,23.93)	76.56 (47.32,110.80)	18.24 (12.38,26.26)	166.28 (118.44,220.13)	17.67 (12.77,25.40)
**DIC**	41.38 (28.43,60.90)	29.70 (24.64,40.09)	85.95 (56.98,120.25)	31.32 (23.04,42.63)	175.40 (127.48,229.23)	31.70 (23.92,40.46)

Table D. 6 95% CrI coverages for prevalence, sensitivities, and specificities across converged CInd and L-L models with known correlation structures fitted to the 1,250 data sets simulated from FE DGM in HIV data setting. Coverages are presented as percentages along with 95% Monte Carlo confidence intervals.

	Sample Size = 500		Sample Size = 2000		Sample Size = 5000	
**Parameter**	**CInd**	**L-L**	**CInd**	**L-L**	**CInd**	**L-L**
$\boldsymbol{\pi}$	95.0 (93.8,96.2)	95.2 (94.0,96.4)	94.7 (93.5,96.0)	94.6 (93.4,95.9)	95.5 (94.4,96.7)	95.4 (94.3,96.6)
$\boldsymbol{Se_1}$	95.6 (94.5,96.7)	95.9 (94.8,97.0)	95.4 (94.2,96.5)	95.8 (94.7,96.6)	93.6 (92.2,95.0)	94.1 (92.8,95.4)
$\boldsymbol{Se_2}$	95.8 (94.7,96.9)	96.0 (94.9,97.1)	95.1 (93.9,96.3)	94.9 (93.7,96.1)	93.2 (91.8,94.6)	93.4 (92.1,94.8)
$\boldsymbol{Se_3}$	95.1 (93.9,96.3)	95.1 (93.9,96.3)	94.7 (93.5,96.0)	95.0 (93.7,96.2)	93.5 (92.2,94.9)	94.6 (93.4,95.9)
$\boldsymbol{Se_4}$	93.7 (92.3,95.0)	93.5 (92.2,94.9)	94.4 (93.1,95.7)	94.2 (92.9,95.5)	93.9 (92.6,95.2)	94.2 (92.9,95.5)
$\boldsymbol{Sp_1}$	93.5 (92.2,94.9)	94.5 (93.2,95.7)	91.6 (90.1,93.1)	94.6 (93.4,95.9)	91.8 (90.2,93.3)	95.4 (94.3,96.6)
$\boldsymbol{Sp_2}$	95.4 (94.3,96.6)	95.1 (93.9,96.3)	93.7 (92.3,95.0)	93.8 (92.4,95.1)	95.7 (94.6,96.8)	95.0 (93.8,96.2)
$\boldsymbol{Sp_3}$	96.5 (95.5,97.5)	96.1 (95.0,97.2)	95.8 (94.7,96.9)	95.1 (93.9,96.3)	94.8 (93.6,96.0)	94.4 (93.1,95.7)
$\boldsymbol{Sp_4}$	94.6 (93.5,95.8)	95.3 (94.1,96.5)	93.4 (92.1,94.8)	95.4 (94.3,96.6)	93.8 (92.5,95.2)	94.2 (92.9,95.5)

Table D. 7 Mean absolute biases of posterior median estimates for prevalence, sensitivities, and specificities with mean residual deviances and DIC values across converged CInd and L-L models with known correlation structures fitted to the 1,250 data sets simulated from L-L DGM in HIV data setting. Estimates are given with 2.5th and 97.5th percentiles.

	Sample Size = 500		Sample Size = 2000		Sample Size = 5000	
**Parameter**	**CInd**	**L-L**	**CInd**	**L-L**	**CInd**	**L-L**
π	-0.001 (-0.044,0.045)	0.000 (-0.043,0.046)	-0.001 (-0.022,0.020)	0.000 (-0.021,0.021)	-0.001 (-0.013,0.013)	0.000 (-0.012,0.013)
Se_1	-0.003 (-0.011,0.001)	-0.003 (-0.011,0.001)	-0.001 (-0.005,0.002)	-0.001 (-0.005,0.002)	0.000 (-0.003,0.002)	0.000 (-0.003,0.002)
Se_2	0.002 (-0.057,0.058)	0.002 (-0.056,0.058)	0.001 (-0.029,0.029)	0.000 (-0.029,0.029)	0.001 (-0.018,0.021)	0.000 (-0.018,0.020)
Se_3	0.000 (-0.034,0.032)	-0.002 (-0.035,0.031)	0.001 (-0.016,0.019)	-0.001 (-0.018,0.017)	0.001 (-0.010,0.012)	0.000 (-0.011,0.010)
Se_4	-0.003 (-0.011,0.001)	-0.003 (-0.011,0.001)	-0.001 (-0.005,0.002)	-0.001 (-0.005,0.002)	0.000 (-0.003,0.002)	0.000 (-0.003,0.002)
Sp_1	-0.004 (-0.031,0.018)	-0.002 (-0.027,0.019)	-0.003 (-0.015,0.009)	-0.001 (-0.013,0.011)	-0.002 (-0.010,0.005)	0.000 (-0.008,0.007)
Sp_2	-0.003 (-0.028,0.021)	-0.003 (-0.029,0.021)	0.000 (-0.013,0.011)	0.000 (-0.013,0.011)	0.000 (-0.008,0.008)	0.000 (-0.008,0.007)
Sp_3	-0.003 (-0.012,0.001)	-0.003 (-0.012,0.001)	-0.001 (-0.005,0.003)	-0.001 (-0.005,0.003)	0.000 (-0.003,0.002)	0.000 (-0.003,0.002)
Sp_4	-0.005 (-0.046,0.031)	-0.003 (-0.042,0.031)	-0.003 (-0.023,0.015)	-0.001 (-0.021,0.016)	-0.002 (-0.015,0.009)	0.000 (-0.012,0.011)
**ResD**	24.89 (14.89,40.40)	15.36 (12.74,20.31)	54.13 (31.92,79.28)	15.57 (11.10,23.20)	111.20 (73.60,151.50)	15.13 (11.17,22.39)
**DIC**	35.59 (25.35,51.34)	27.22 (23.73,32.43)	63.57 (41.17,88.62)	26.08 (21.42,33.55)	120.33 (82.77,160.65)	25.29 (21.34,32.52)

Table D. 8 95% CrI coverages for prevalence, sensitivities, and specificities across converged CInd and L-L models with known correlation structures fitted to the 1,250 data sets simulated from L-L DGM in HIV data setting. Coverages are presented as percentages along with 95% Monte Carlo confidence intervals.

	Sample Size = 500		Sample Size = 2000		Sample Size = 5000	
**Parameter**	**CInd**	**L-L**	**CInd**	**L-L**	**CInd**	**L-L**
π	94.4 (93.1,95.7)	94.3 (93.0,95.6)	96.2 (95.2,97.3)	95.8 (94.6,96.9)	96.5 (95.5,97.5)	96.3 (95.3,97.4)
Se_1	96.2 (95.2,97.3)	96.2 (95.2,97.3)	96.4 (95.4,97.4)	96.4 (95.4,97.4)	95.4 (94.3,96.6)	95.6 (94.5,96.7)
Se_2	95.4 (94.2,96.5)	95.4 (94.2,96.5)	95.5 (94.4,96.7)	95.6 (94.5,96.7)	93.7 (92.3,95.0)	94.3 (93.0,95.6)
Se_3	96.4 (95.4,97.4)	96.5 (95.5,97.5)	94.0 (92.7,95.3)	94.4 (93.1,95.7)	95.1 (93.9,96.3)	95.6 (94.5,96.7)
Se_4	94.0 (92.7,95.3)	94.0 (92.7,95.3)	95.8 (94.7,96.9)	96.2 (95.2,97.3)	94.3 (93.0,95.6)	94.0 (92.7,95.3)
Sp_1	94.0 (92.7,95.3)	95.8 (94.6,96.9)	92.8 (91.4,94.2)	95.1 (93.9,96.3)	91.7 (90.1,93.2)	95.8 (94.6,96.9)
Sp_2	95.2 (94.0,96.4)	94.7 (93.5,96.0)	95.4 (94.2,96.5)	94.9 (93.7,96.1)	95.4 (94.3,96.6)	95.3 (94.1,96.5)
Sp_3	96.7 (95.7,97.7)	96.7 (95.7,97.7)	95.4 (94.3,96.6)	95.4 (94.3,96.6)	93.2 (91.8,94.6)	93.2 (91.8,94.6)
Sp_4	94.3 (93.0,95.6)	94.5 (93.2,95.7)	93.2 (91.8,94.6)	94.6 (93.3,95.8)	93.4 (92.1,94.8)	94.4 (93.1,95.7)

Table D. 9 Mean absolute biases of posterior median estimates for prevalence, sensitivities, and specificities with mean residual deviances, DIC values, CrI widths providing minimum distances, corresponding correct inclusion rates (CIR) and false inclusion rates (FIR) of the interaction terms, as well as 95% CrI distances, CIRs and FIRs across converged L-L models with all interaction terms within the diseased state with three different priors (Hyplerlasso, Elastic Net, Regularized Horseshoe) fitted to the 500 data sets simulated from L-L DGM in HIV data setting with the sample sizes of 500. Estimates are given with 2.5th and 97.5th percentiles.

	**Sample size = 500**		
**Parameter**	**L-L with HL priors**	**L-L with EN priors**	**L-L with RH priors**
$\boldsymbol{\pi}$	0.001 (-0.045,0.048)	0.002 (-0.044,0.049)	0.002 (-0.045,0.048)
$\boldsymbol{Se_1}$	-0.001 (-0.010,0.003)	-0.002 (-0.012,0.002)	-0.001 (-0.011,0.003)
$\boldsymbol{Se_2}$	0.003 (-0.054,0.061)	0.003 (-0.054,0.060)	0.003 (-0.054,0.060)
$\boldsymbol{Se_3}$	0.000 (-0.035,0.032)	0.000 (-0.035,0.032)	-0.001 (-0.035,0.031)
$\boldsymbol{Se_4}$	-0.001 (-0.010,0.004)	-0.002 (-0.011,0.003)	-0.001 (-0.011,0.003)
$\boldsymbol{Sp_1}$	-0.002 (-0.027,0.018)	-0.002 (-0.027,0.019)	-0.002 (-0.027,0.018)
$\boldsymbol{Sp_2}$	-0.003 (-0.028,0.021)	-0.003 (-0.028,0.021)	-0.003 (-0.028,0.021)
$\boldsymbol{Sp_3}$	-0.003 (-0.013,0.001)	-0.003 (-0.012,0.001)	-0.003 (-0.012,0.001)
$\boldsymbol{Sp_4}$	-0.004 (-0.045,0.031)	-0.003 (-0.044,0.031)	-0.004 (-0.044,0.031)
**ResD**	14.37 (10.39,19.27)	14.29 (11.36,18.49)	14.60 (11.37,19.04)
**DIC**	25.74 (19.92,32.62)	25.85 (21.76,31.49)	26.23 (22.00,31.82)
**Minimum Distance**	0.225 (70% CrI)	0.216 (80% CrI)	0.218 (70% CrI)
**CIR**	0.934	0.921	0.928
**FIR**	0.215	0.201	0.205
**95% CrI Distance**	0.337	0.268	0.349
**95% CrI CIR**	0.703	0.755	0.672
**95% CrI FIR**	0.161	0.109	0.118

Table D. 10 95% CrI coverages for prevalence, sensitivities, and specificities across converged L-L models with all interaction terms within the diseased state with three different priors (Hyplerlasso, Elastic Net, Regularized Horseshoe) fitted to the 500 data sets simulated from L-L DGM in HIV data setting with the sample sizes of 500. Coverages are presented as percentages along with 95% Monte Carlo confidence intervals.

	**Sample size = 500**		
**Parameter**	**L-L with HL priors**	**L-L with EN priors**	**L-L with RH priors**
π	92.8 (90.5,95.1)	92.7 (90.4,95.0)	92.8 (90.5,95.0)
Se_1	97.6 (96.3,98.9)	96.4 (94.7,98.0)	96.8 (95.2,98.3)
Se_2	95.2 (93.3,97.1)	95.1 (93.2,97.0)	95.4 (93.5,97.2)
Se_3	96.4 (94.8,98.0)	96.6 (95.0,98.2)	96.4 (94.7,98.0)
Se_4	97.6 (96.3,98.9)	96.0 (94.2,97.7)	96.0 (94.2,97.7)
Sp_1	95.6 (93.8,97.4)	96.4 (94.0,98.0)	95.8 (94.0,97.5)
Sp_2	94.2 (92.1,96.2)	94.7 (92.8,96.7)	94.8 (92.8,96.7)
Sp_3	95.2 (93.3,97.1)	96.8 (95.2,98.3)	97.0 (95.5,98.5)
Sp_4	94.0 (91.9,96.1)	93.3 (91.1,95.5)	93.8 (91.6,95.9)

Table D. 11 Mean absolute biases of posterior median estimates for prevalence, sensitivities, and specificities with mean residual deviances, DIC values, CrI widths providing minimum distances, corresponding correct inclusion rates (CIR) and false inclusion rates (FIR) of the interaction terms, as well as 95% CrI distances, CIRs and FIRs across converged L-L models with all interaction terms within the diseased state with three different priors (Hyplerlasso, Elastic Net, Regularized Horseshoe) fitted to the 500 data sets simulated from L-L DGM in HIV data setting with the sample sizes of 2000. Estimates are given with 2.5th and 97.5th percentiles.

	**Sample size = 2000**		
**Parameter**	**L-L with HL priors**	**L-L with EN priors**	**L-L with RH priors**
$\boldsymbol{\pi}$	0.002 (-0.019,0.023)	0.002 (-0.019,0.023)	0.002 (-0.019,0.023)
$\boldsymbol{Se_1}$	-0.001 (-0.006,0.003)	-0.001 (-0.006,0.002)	-0.001 (-0.006,0.002)
$\boldsymbol{Se_2}$	0.000 (-0.030,0.029)	-0.001 (-0.031,0.029)	0.000 (-0.030,0.029)
$\boldsymbol{Se_3}$	-0.001 (-0.020,0.018)	-0.001 (-0.020,0.018)	-0.001 (-0.020,0.018)
$\boldsymbol{Se_4}$	-0.001 (-0.005,0.003)	-0.001 (-0.006,0.002)	-0.001 (-0.006,0.002)
$\boldsymbol{Sp_1}$	-0.001 (-0.012,0.011)	0.000 (-0.012,0.012)	0.000 (-0.012,0.011)
$\boldsymbol{Sp_2}$	0.000 (-0.013,0.012)	0.000 (-0.013,0.012)	0.000 (-0.013,0.012)
$\boldsymbol{Sp_3}$	-0.001 (-0.006,0.003)	-0.001 (-0.006,0.003)	-0.001 (-0.005,0.003)
$\boldsymbol{Sp_4}$	0.000 (-0.021,0.017)	0.000 (-0.020,0.017)	0.000 (-0.021,0.017)
**ResD**	15.18 (12.07,20.56)	14.79 (11.84,20.31)	15.02 (11.75,20.78)
**DIC**	26.76 (22.88,33.50)	26.16 (22.48,32.51)	26.30 (22.34,33.29)
**Minimum Distance**	0.167 (95% CrI)	0.146 (95% CrI)	0.127 (95% CrI)
**CIR**	1.000	1.000	1.000
**FIR**	0.167	0.146	0.127
**95% CrI Distance**	0.167	0.146	0.127
**95% CrI CIR**	1.000	1.000	1.000
**95% CrI FIR**	0.167	0.146	0.127

Table D. 12 95% CrI coverages for prevalence, sensitivities, and specificities across converged L-L models with all interaction terms within the diseased state with three different priors (Hyplerlasso, Elastic Net, Regularized Horseshoe) fitted to the 500 data sets simulated from L-L DGM in HIV data setting with the sample sizes of 2000. Coverages are presented as percentages along with 95% Monte Carlo confidence intervals.

	**Sample size = 2000**		
**Parameter**	**L-L with HL priors**	**L-L with EN priors**	**L-L with RH priors**
$\boldsymbol{\pi}$	95.6 (93.8,97.4)	95.8 (94.0,97.6)	95.4 (93.5,97.2)
$\boldsymbol{Se_1}$	96.2 (94.5,97.9)	95.4 (93.6,97.2)	96.0 (94.3,97.7)
$\boldsymbol{Se_2}$	94.8 (92.9,96.7)	94.8 (92.8,96.7)	94.8 (92.8,96.7)
$\boldsymbol{Se_3}$	93.4 (91.2,95.6)	92.8 (90.5,95.1)	93.0 (90.7,95.2)
$\boldsymbol{Se_4}$	93.4 (91.2,95.6)	94.4 (92.4,96.4)	94.0 (91.9,96.1)
$\boldsymbol{Sp_1}$	96.0 (94.3,97.7)	95.6 (93.8,97.4)	95.6 (93.8,97.4)
$\boldsymbol{Sp_2}$	95.0 (93.1,96.9)	95.3 (93.1,96.9)	95.0 (93.1,96.9)
$\boldsymbol{Sp_3}$	96.5 (95.0,98.2)	95.6 (93.8,97.4)	96.4 (94.7,98.0)
$\boldsymbol{Sp_4}$	94.4 (92.4,96.4)	94.2 (92.1,96.2)	94.2 (92.1,96.2)

Table D. 13 Mean absolute biases of posterior median estimates for prevalence, sensitivities, and specificities with mean residual deviances, DIC values, CrI widths providing minimum distances, corresponding correct inclusion rates (CIR) and false inclusion rates (FIR) of the interaction terms, as well as 95% CrI distances, CIRs and FIRs across converged L-L models with all interaction terms within the diseased state with three different priors (Hyplerlasso, Elastic Net, Regularized Horseshoe) fitted to the 500 data sets simulated from L-L DGM in HIV data setting with the sample sizes of 5000. Estimates are given with 2.5th and 97.5th percentiles.

	**Sample size = 5000**		
**Parameter**	**L-L with HL priors**	**L-L with EN priors**	**L-L with RH priors**
$\boldsymbol{\pi}$	0.002 (-0.011,0.015)	0.002 (-0.011,0.015)	0.001 (-0.011,0.015)
$\boldsymbol{Se_1}$	-0.001 (-0.004,0.002)	-0.001 (-0.004,0.001)	-0.001 (-0.004,0.002)
$\boldsymbol{Se_2}$	0.000 (-0.019,0.019)	0.000 (-0.019,0.019)	0.000 (-0.019,0.019)
$\boldsymbol{Se_3}$	-0.001 (-0.012,0.010)	-0.001 (-0.013,0.009)	-0.001 (-0.012,0.010)
$\boldsymbol{Se_4}$	-0.001 (-0.004,0.002)	-0.001 (-0.004,0.001)	-0.001 (-0.004,0.002)
$\boldsymbol{Sp_1}$	0.000 (-0.007,0.008)	0.001 (-0.007,0.008)	0.000 (-0.007,0.008)
$\boldsymbol{Sp_2}$	0.000 (-0.007,0.008)	0.000 (-0.007,0.008)	0.000 (-0.007,0.008)
$\boldsymbol{Sp_3}$	0.000 (-0.003,0.002)	0.000 (-0.003,0.002)	0.000 (-0.003,0.002)
$\boldsymbol{Sp_4}$	0.000 (-0.012,0.012)	0.000 (-0.012,0.012)	0.000 (-0.012,0.012)
**ResD**	14.99 (12.36,20.18)	14.63 (12.35,19.76)	14.76 (12.13,19.91)
**DIC**	26.93 (23.40,33.59)	26.33 (23.41,32.28)	26.39 (23.05,32.98)
**Minimum Distance**	0.141 (95% CrI)	0.118 (95% CrI)	0.097 (95% CrI)
**CIR**	1.000	1.000	1.000
**FIR**	0.141	0.118	0.097
**95% CrI Distance**	0.141	0.118	0.097
**95% CrI CIR**	1.000	1.000	1.000
**95% CrI FIR**	0.141	0.118	0.097

Table D. 14 95% CrI coverages for prevalence, sensitivities, and specificities across converged L-L models with all interaction terms within the diseased state with three different priors (Hyplerlasso, Elastic Net, Regularized Horseshoe) fitted to the 500 data sets simulated from L-L DGM in HIV data setting with the sample sizes of 5000. Coverages are presented as percentages along with 95% Monte Carlo confidence intervals.

	**Sample size = 5000**		
**Parameter**	**L-L with HL priors**	**L-L with EN priors**	**L-L with RH priors**
$\boldsymbol{\pi}$	95.0 (93.0,96.9)	95.6 (93.7,97.4)	95.3 (93.4,97.2)
$\boldsymbol{Se_1}$	94.2 (92.1,96.3)	94.1 (92.1,96.2)	94.3 (92.2,96.3)
$\boldsymbol{Se_2}$	94.4 (92.3,96.4)	94.1 (92.1,96.2)	93.8 (91.7,96.0)
$\boldsymbol{Se_3}$	95.4 (93.5,97.3)	95.4 (93.5,97.2)	95.7 (93.9,97.5)
$\boldsymbol{Se_4}$	93.7 (91.6,95.9)	93.1 (90.9,95.4)	93.6 (91.5,95.8)
$\boldsymbol{Sp_1}$	95.8 (94.0,97.6)	95.4 (93.5,97.2)	95.7 (93.9,97.5)
$\boldsymbol{Sp_2}$	96.0 (94.3,97.8)	95.6 (93.7,97.4)	95.9 (94.1,97.7)
$\boldsymbol{Sp_3}$	94.6 (92.5,96.6)	93.7 (91.6,95.9)	93.8 (91.7,96.0)
$\boldsymbol{Sp_4}$	94.4 (92.3,96.4)	94.9 (93.0,96.9)	94.9 (92.9,96.8)

Table D. 15 Mean absolute biases of posterior median estimates for prevalence, sensitivities, and specificities with mean residual deviances and DIC values across converged CInd and L-L models with known correlation structures fitted to the 1,250 data sets simulated from LT DGM in VL data setting. Estimates are given with 2.5th and 97.5th percentiles.

	Sample Size = 500		Sample Size = 2000		Sample Size = 5000	
**Parameter**	**CInd**	**L-L**	**CInd**	**L-L**	**CInd**	**L-L**
$\boldsymbol{\pi}$	-0.095 (-0.137,-0.051)	-0.044 (-0.096,0.003)	-0.097 (-0.117,-0.076)	-0.031 (-0.062,0.003)	-0.098 (-0.110,-0.084)	-0.016 (-0.046,0.016)
$\boldsymbol{Se_1}$	0.051 (-0.006,0.101)	0.043 (-0.016,0.093)	0.054 (0.027,0.080)	0.034 (-0.012,0.068)	0.055 (0.039,0.071)	0.018 (-0.026,0.052)
$\boldsymbol{Se_2}$	0.063 (0.001,0.118)	0.053 (-0.007,0.110)	0.067 (0.034,0.097)	0.041 (-0.005,0.081)	0.067 (0.046,0.086)	0.019 (-0.026,0.061)
$\boldsymbol{Se_3}$	0.229 (0.162,0.281)	0.099 (0.011,0.196)	0.234 (0.206,0.260)	0.063 (0.000,0.119)	0.236 (0.217,0.253)	0.031 (-0.026,0.089)
$\boldsymbol{Se_4}$	0.231 (0.163,0.272)	0.099 (0.011,0.197)	0.239 (0.214,0.261)	0.065 (0.001,0.119)	0.240 (0.225,0.254)	0.031 (-0.028,0.089)
$\boldsymbol{Sp_1}$	-0.090 (-0.128,-0.054)	-0.035 (-0.075,-0.005)	-0.090 (-0.108,-0.073)	-0.022 (-0.041,-0.001)	-0.090 (-0.102,-0.079)	-0.010 (-0.031,0.008)
$\boldsymbol{Sp_2}$	-0.068 (-0.110,-0.030)	-0.020 (-0.063,0.015)	-0.069 (-0.087,-0.049)	-0.012 (-0.031,0.008)	-0.069 (-0.081,-0.057)	-0.007 (-0.022,0.009)
$\boldsymbol{Sp_3}$	-0.003 (-0.020,0.011)	-0.002 (-0.019,0.012)	-0.002 (-0.010,0.006)	-0.001 (-0.010,0.007)	-0.001 (-0.006,0.004)	0.000 (-0.006,0.005)
$\boldsymbol{Sp_4}$	-0.004 (-0.021,0.010)	-0.003 (-0.019,0.010)	-0.003 (-0.011,0.005)	-0.001 (-0.010,0.006)	-0.003 (-0.008,0.002)	-0.001 (-0.006,0.004)
**ResD**	53.18 (28.54,85.26)	20.62 (14.74,29.62)	163.62 (114.70,216.91)	17.97 (13.74,26.01)	384.78 (309.87,465.91)	17.52 (13.44,24.99)
**DIC**	62.36 (37.60,94.40)	38.55 (29.44,56.33)	172.65 (123.73,226.01)	33.01 (27.19,42.13)	393.79 (318.91,474.85)	32.05 (26.73,40.23)

Table D. 16 95% CrI coverages for prevalence, sensitivities, and specificities across converged CInd and L-L models with known correlation structures fitted to the 1,250 data sets simulated from LT DGM in VL data setting. Coverages are presented as percentages along with 95% Monte Carlo confidence intervals.

	Sample Size = 500		Sample Size = 2000		Sample Size = 5000	
**Parameter**	**CInd**	**L-L**	**CInd**	**L-L**	**CInd**	**L-L**
$\boldsymbol{\pi}$	0.9 (0.4,1.4)	73.3 (70.9,75.8)	0.0 (0.0,0.0)	83.6 (81.4,85.7)	0.0 (0.0,0.0)	93.9 (92.5,95.3)
$\boldsymbol{Se_1}$	58.9 (56.2,61.6)	83.2 (81.1,85.3)	4.4 (3.3,5.5)	84.4 (82.2,86.5)	0.0 (0.0,0.0)	93.9 (92.5,95.3)
$\boldsymbol{Se_2}$	54.9 (52.1,57.6)	83.0 (80.8,85.1)	2.8 (1.9,3.7)	85.5 (83.5,87.6)	0.0 (0.0,0.0)	94.6 (93.3,95.9)
$\boldsymbol{Se_3}$	0.2 (0.0,0.4)	61.3 (58.5,64.0)	0.0 (0.0,0.0)	81.0 (78.7,83.3)	0.0 (0.0,0.0)	93.3 (91.9,94.8)
$\boldsymbol{Se_4}$	0.2 (0.0,0.4)	59.5 (56.8,62.3)	0.0 (0.0,0.0)	81.3 (79.1,83.6)	0.0 (0.0,0.0)	94.0 (92.7,95.4)
$\boldsymbol{Sp_1}$	0.2 (0.0,0.4)	59.1 (56.3,61.8)	0.0 (0.0,0.0)	80.3 (78.0,82.6)	0.0 (0.0,0.0)	94.5 (93.2,95.8)
$\boldsymbol{Sp_2}$	4.7 (3.5,5.9)	82.2 (80.1,84.4)	0.0 (0.0,0.0)	83.2 (81.0,85.4)	0.0 (0.0,0.0)	90.9 (89.2,92.5)
$\boldsymbol{Sp_3}$	93.2 (91.8,94.6)	94.5 (93.2,95.7)	92.9 (91.5,94.3)	92.9 (91.4,94.4)	91.9 (90.4,93.4)	94.4 (93.1,95.7)
$\boldsymbol{Sp_4}$	91.6 (90.1,93.1)	95.0 (93.8,96.2)	87.9 (86.1,89.7)	94.5 (93.2,95.8)	78.6 (76.3,80.8)	94.8 (93.5,96.1)

Table D. 17 Mean absolute biases of posterior median estimates for prevalence, sensitivities, and specificities with mean residual deviances and DIC values across converged CInd and L-L models with known correlation structures fitted to the 1,250 data sets simulated from FE DGM in VL data setting. Estimates are given with 2.5th and 97.5th percentiles.

	Sample Size = 500		Sample Size = 2000		Sample Size = 5000	
**Parameter**	**CInd**	**L-L**	**CInd**	**L-L**	**CInd**	**L-L**
π	-0.047 (-0.094,0.008)	-0.006 (-0.050,0.040)	-0.047 (-0.077,-0.017)	-0.001 (-0.025,0.021)	-0.048 (-0.068,-0.026)	0.000 (-0.016,0.015)
Se_1	0.001 (-0.056,0.054)	-0.002 (-0.060,0.052)	0.002 (-0.029,0.030)	-0.001 (-0.033,0.028)	0.002 (-0.016,0.020)	-0.001 (-0.019,0.018)
Se_2	0.008 (-0.058,0.075)	0.004 (-0.062,0.069)	0.009 (-0.026,0.042)	0.000 (-0.035,0.033)	0.010 (-0.012,0.031)	0.000 (-0.021,0.022)
Se_3	0.109 (-0.003,0.201)	0.017 (-0.062,0.090)	0.109 (0.042,0.170)	0.004 (-0.034,0.040)	0.112 (0.056,0.155)	0.002 (-0.022,0.027)
Se_4	0.111 (-0.003,0.202)	0.016 (-0.062,0.094)	0.112 (0.045,0.173)	0.004 (-0.033,0.042)	0.155 (0.062,0.158)	0.002 (-0.022,0.027)
Sp_1	-0.058 (-0.107,-0.011)	-0.010 (-0.039,0.010)	-0.057 (-0.087,-0.025)	-0.002 (-0.016,0.010)	-0.058 (-0.080,-0.035)	-0.001 (-0.010,0.008)
Sp_2	-0.045 (-0.098,0.006)	-0.006 (-0.041,0.028)	-0.044 (-0.076,-0.010)	-0.001 (-0.018,0.016)	-0.045 (-0.068,-0.020)	-0.001 (-0.012,0.009)
Sp_3	0.000 (-0.017,0.014)	-0.001 (-0.018,0.013)	0.002 (-0.007,0.009)	0.000 (-0.009,0.007)	0.002 (-0.004,0.007)	0.000 (-0.006,0.005)
Sp_4	-0.001 (-0.017,0.011)	-0.001 (-0.017,0.011)	0.001 (-0.006,0.008)	0.000 (-0.008,0.007)	0.002 (-0.003,0.006)	0.000 (-0.005,0.005)
**ResD**	64.65 (35.51,96.26)	17.92 (13.10,25.45)	215.70 (156.10,281.76)	15.87 (12.18,23.05)	516.08 (421.89,609.69)	15.40 (11.81,22.47)
**DIC**	73.67 (44.60,105.73)	31.97 (25.48,40.83)	224.67 (165.08,290.38)	28.22 (24.31,35.46)	525.04 (430.89,618.47)	27.01 (23.36,34.19)

Table D. 18 95% CrI coverages for prevalence, sensitivities, and specificities across converged CInd and L-L models with known correlation structures fitted to the 1,250 data sets simulated from FE DGM in VL data setting. Coverages are presented as percentages along with 95% Monte Carlo confidence intervals.

	Sample Size = 500		Sample Size = 2000		Sample Size = 5000	
**Parameter**	**CInd**	**L-L**	**CInd**	**L-L**	**CInd**	**L-L**
π	50.2 (47.5,53.0)	94.9 (93.7,96.1)	8.8 (7.2,10.4)	95.4 (94.3,96.6)	0.0 (0.0,0.0)	94.2 (92.9,95.5)
Se_1	96.2 (95.1,97.2)	96.0 (94.9,97.1)	94.1 (92.8,95.4)	94.1 (92.8,95.4)	95.0 (93.7,96.2)	95.3 (94.1,96.5)
Se_2	93.3 (91.9,94.7)	94.8 (93.6,96.0)	90.0 (88.3,91.7)	94.3 (93.0,95.6)	85.0 (83.1,87.0)	94.9 (93.7,96.1)
Se_3	32.8 (30.2,35.4)	92.6 (91.1,94.0)	2.0 (1.2,2.8)	95.4 (94.3,96.6)	0.0 (0.0,0.0)	94.2 (92.9,95.5)
Se_4	32.9 (30.3,35.5)	91.4 (89.9,93.0)	1.5 (0.8,2.2)	95.3 (94.1,96.5)	0.0 (0.0,0.0)	95.0 (93.8,96.2)
Sp_1	12.7 (10.9,14.6)	91.1 (89.5,92.7)	0.2 (0.0,0.5)	95.0 (93.7,96.2)	0.0 (0.0,0.0)	94.2 (92.9,95.5)
Sp_2	42.3 (39.6,45.1)	93.8 (92.4,95.1)	10.3 (8.6,12.0)	94.9 (93.7,96.1)	0.4 (0.1,0.7)	95.3 (94.1,96.5)
Sp_3	96.1 (95.0,97.2)	95.7 (94.6,96.8)	92.5 (91.0,93.9)	95.0 (93.8,96.2)	87.0 (85.2,88.9)	94.2 (92.9,95.5)
Sp_4	96.5 (95.5,97.5)	96.2 (95.1,97.2)	93.4 (92.1,94.8)	95.0 (93.8,96.2)	88.3 (86.5,90.1)	94.8 (93.6,96.0)

Table D. 19 Mean absolute biases of posterior median estimates for prevalence, sensitivities, and specificities with mean residual deviances and DIC values across converged CInd and L-L models with known correlation structures fitted to the 1,250 data sets simulated from L-L DGM in VL data setting. Estimates are given with 2.5th and 97.5th percentiles.

	Sample Size = 500		Sample Size = 2000		Sample Size = 5000	
**Parameter**	**CInd**	**L-L**	**CInd**	**L-L**	**CInd**	**L-L**
π	-0.046 (-0.094,0.007)	-0.006 (-0.050,0.040)	-0.046 (-0.076,-0.016)	-0.002 (-0.025,0.023)	-0.046 (-0.067,-0.025)	0.000 (-0.014,0.014)
Se_1	0.001 (-0.056,0.055)	-0.002 (-0.061,0.053)	0.003 (-0.025,0.030)	0.000 (-0.030,0.028)	0.002 (-0.016,0.020)	-0.001 (-0.020,0.018)
Se_2	0.009 (-0.056,0.074)	0.003 (-0.063,0.067)	0.010 (-0.023,0.043)	0.001 (-0.032,0.033)	0.010 (-0.012,0.032)	0.000 (-0.022,0.022)
Se_3	0.106 (-0.004,0.199)	0.017 (-0.058,0.089)	0.106 (0.041,0.164)	0.005 (-0.033,0.043)	0.107 (0.059,0.150)	0.002 (-0.022,0.025)
Se_4	0.108 (-0.003,0.201)	0.016 (-0.060,0.090)	0.109 (0.045,0.168)	0.005 (-0.032,0.045)	0.110 (0.061,0.153)	0.002 (-0.023,0.025)
Sp_1	-0.057 (-0.104,-0.011)	-0.010 (-0.037,0.010)	-0.055 (-0.084,-0.026)	-0.003 (-0.017,0.010)	-0.056 (-0.079,-0.033)	-0.001 (-0.010,0.008)
Sp_2	-0.044 (-0.097,0.006)	-0.006 (-0.043,0.027)	-0.042 (-0.074,-0.010)	-0.002 (-0.018,0.016)	-0.043 (-0.066,-0.019)	-0.001 (-0.012,0.010)
Sp_3	0.000 (-0.017,0.014)	-0.001 (-0.018,0.013)	0.002 (-0.007,0.009)	0.000 (-0.009,0.008)	0.002 (-0.003,0.007)	0.000 (-0.005,0.005)
Sp_4	-0.001 (-0.017,0.011)	-0.002 (-0.017,0.011)	0.001 (-0.006,0.008)	0.000 (-0.008,0.007)	0.002 (-0.003,0.006)	0.000 (-0.005,0.004)
**ResD**	63.55 (35.10,95.38)	17.89 (13.04,25.50)	209.42 (150.55,274.34)	15.81 (12.16,22.58)	507.91 (411.15,609.57)	15.44 (11.81,22.89)
**DIC**	72.57 (44.09,104.13)	31.81 (25.46,40.57)	218.39 (159.43,283.10)	28.09 (24.24,34.98)	516.88 (420.11,618.72)	27.02 (23.36,34.52)

Table D. 20 95% CrI coverages for prevalence, sensitivities, and specificities across converged CInd and L-L models with known correlation structures fitted to the 1,250 data sets simulated from L-L DGM in VL data setting. Coverages are presented as percentages along with 95% Monte Carlo confidence intervals.

	Sample Size = 500		Sample Size = 2000		Sample Size = 5000	
**Parameter**	**CInd**	**L-L**	**CInd**	**L-L**	**CInd**	**L-L**
π	50.9 (48.1,53.7)	95.0 (93.8,96.2)	8.7 (7.2,10.3)	94.6 (93.4,95.9)	0.2 (-0.1,0.4)	96.6 (95.5,97.6)
Se_1	95.3 (94.1,96.5)	95.4 (94.2,96.5)	94.9 (93.7,96.1)	95.5 (94.4,96.7)	94.3 (93.0,95.6)	94.4 (93.1,95.7)
Se_2	93.6 (92.2,95.0)	95.5 (94.4,96.7)	90.3 (88.7,92.0)	95.6 (94.5,96.7)	83.3 (81.2,85.3)	94.7 (93.5,96.0)
Se_3	33.8 (31.2,36.5)	93.4 (92.0,94.7)	2.3 (1.5,3.2)	93.8 (92.4,95.1)	0.0 (0.0,0.0)	95.4 (94.2,96.5)
Se_4	33.0 (30.4,35.6)	93.1 (91.7,94.5)	1.8 (1.1,2.6)	93.4 (92.1,94.8)	0.0 (0.0,0.0)	95.1 (93.9,96.3)
Sp_1	13.5 (11.6,15.4)	91.0 (89.5,92.6)	0.0 (0.0,0.0)	94.0 (92.7,95.3)	0.0 (0.0,0.0)	94.7 (93.5,96.0)
Sp_2	44.7 (42.0,47.5)	93.2 (91.8,94.6)	11.8 (10.0,13.5)	94.5 (93.2,95.7)	0.2 (-0.1,0.4)	95.0 (93.7,96.2)
Sp_3	96.1 (95.0,97.2)	96.1 (95.0,97.2)	92.0 (90.5,93.5)	94.2 (92.9,95.5)	87.5 (85.7,89.4)	95.9 (94.8,97.0)
Sp_4	96.2 (95.1,97.2)	95.8 (94.7,96.9)	93.4 (92.1,94.8)	94.9 (93.7,96.1)	89.5 (87.8,91.2)	94.6 (93.4,95.9)

Table D. 21 Mean absolute biases of posterior median estimates for prevalence, sensitivities, and specificities with mean residual deviances, DIC values, CrI widths providing minimum distances, corresponding correct inclusion rates (CIR) and false inclusion rates (FIR) of the interaction terms, as well as 95% CrI distances, CIRs and FIRs across converged L-L models with all interaction terms within the diseased state with three different priors (Hyplerlasso, Elastic Net, Regularized Horseshoe) fitted to the 500 data sets simulated from L-L DGM in VL data setting with the sample sizes of 500. Estimates are given with 2.5th and 97.5th percentiles.

	**Sample size = 500**		
**Parameter**	**L-L with HL priors**	**L-L with EN priors**	**L-L with RH priors**
$\boldsymbol{\pi}$	-0.003 (-0.049,0.046)	-0.005 (-0.051,0.045)	-0.003 (-0.049,0.046)
$\boldsymbol{Se_1}$	-0.006 (-0.059,0.049)	-0.006 (-0.062,0.047)	-0.006 (-0.059,0.049)
$\boldsymbol{Se_2}$	0.006 (-0.053,0.069)	0.009 (-0.051,0.071)	0.006 (-0.054,0.069)
$\boldsymbol{Se_3}$	0.012 (-0.059,0.089)	0.015 (-0.058,0.091)	0.012 (-0.058,0.089)
$\boldsymbol{Se_4}$	0.012 (-0.060,0.084)	0.014 (-0.058,0.086)	0.012 (-0.060,0.084)
$\boldsymbol{Sp_1}$	-0.011 (-0.039,0.007)	-0.013 (-0.042,0.006)	-0.011 (-0.039,0.007)
$\boldsymbol{Sp_2}$	-0.003 (-0.038,0.032)	-0.003 (-0.039,0.033)	-0.003 (-0.038,0.032)
$\boldsymbol{Sp_3}$	-0.001 (-0.019,0.013)	-0.001 (-0.019,0.013)	-0.001 (-0.019,0.013)
$\boldsymbol{Sp_4}$	-0.003 (-0.018,0.010)	-0.002 (-0.018,0.010)	-0.002 (-0.018,0.010)
**ResD**	15.86 (12.32,21.98)	16.35 (13.50,21.70)	15.91 (12.33,21.99)
**DIC**	28.86 (23.99,37.25)	31.24 (26.77,39.13)	28.96 (24.11,37.43)
**Minimum Distance**	0.442 (50% CrI)	0.440 (60% CrI)	0.441 (50% CrI)
**CIR**	0.578	0.600	0.580
**FIR**	0.131	0.184	0.135
**95% CrI Distance**	0.499	0.498	0.499
**95% CrI CIR**	0.501	0.502	0.501
**95% CrI FIR**	0.001	0.004	0.001

Table D. 22 95% CrI coverages for prevalence, sensitivities, and specificities across converged L-L models with all interaction terms within the diseased state with three different priors (Hyplerlasso, Elastic Net, Regularized Horseshoe) fitted to the 500 data sets simulated from L-L DGM in VL data setting with the sample sizes of 500. Coverages are presented as percentages along with 95% Monte Carlo confidence intervals.

	**Sample size = 500**		
**Parameter**	**L-L with HL priors**	**L-L with EN priors**	**L-L with RH priors**
$\boldsymbol{\pi}$	96.4 (94.8,98.0)	96.4 (94.8,98.0)	96.4 (94.8,98.0)
$\boldsymbol{Se_1}$	98.4 (97.3,99.5)	98.6 (97.6,99.6)	98.4 (97.3,99.5)
$\boldsymbol{Se_2}$	95.6 (93.8,97.4)	95.6 (93.8,97.4)	95.6 (93.8,97.4)
$\boldsymbol{Se_3}$	97.0 (95.5,98.5)	97.2 (95.7,98.6)	96.8 (95.3,98.3)
$\boldsymbol{Se_4}$	96.6 (95.0,98.2)	96.6 (95.0,98.2)	96.4 (94.8,98.0)
$\boldsymbol{Sp_1}$	97.6 (96.3,98.9)	96.0 (94.3,97.7)	97.2 (95.8,98.6)
$\boldsymbol{Sp_2}$	96.4 (94.8,98.0)	96.8 (95.2,98.3)	96.4 (94.8,98.0)
$\boldsymbol{Sp_3}$	96.0 (94.3,97.7)	96.2 (94.5,97.9)	96.0 (94.3,97.7)
$\boldsymbol{Sp_4}$	95.6 (93.8,97.4)	95.4 (93.6,97.2)	95.8 (94.0,97.6)

Table D. 23 Mean absolute biases of posterior median estimates for prevalence, sensitivities, and specificities with mean residual deviances, DIC values, CrI widths providing minimum distances, corresponding correct inclusion rates (CIR) and false inclusion rates (FIR) of the interaction terms, as well as 95% CrI distances, CIRs and FIRs across converged L-L models with all interaction terms within the diseased state with three different priors (Hyplerlasso, Elastic Net, Regularized Horseshoe) fitted to the 500 data sets simulated from L-L DGM in VL data setting with the sample sizes of 2000. Estimates are given with 2.5th and 97.5th percentiles.

	**Sample size = 2000**		
**Parameter**	**L-L with HL priors**	**L-L with EN priors**	**L-L with RH priors**
$\boldsymbol{\pi}$	-0.001 (-0.025,0.026)	-0.002 (-0.027,0.026)	-0.001 (-0.025,0.026)
$\boldsymbol{Se_1}$	-0.002 (-0.041,0.028)	-0.002 (-0.044,0.031)	-0.002 (-0.040,0.028)
$\boldsymbol{Se_2}$	0.001 (-0.032,0.034)	0.004 (-0.031,0.039)	0.001 (-0.032,0.035)
$\boldsymbol{Se_3}$	0.004 (-0.036,0.044)	0.007 (-0.035,0.048)	0.005 (-0.036,0.045)
$\boldsymbol{Se_4}$	0.004 (-0.033,0.044)	0.007 (-0.033,0.047)	0.004 (-0.033,0.044)
$\boldsymbol{Sp_1}$	-0.004 (-0.019,0.009)	-0.005 (-0.021,0.008)	-0.004 (-0.019,0.009)
$\boldsymbol{Sp_2}$	-0.001 (-0.019,0.018)	-0.001 (-0.020,0.020)	-0.001 (-0.019,0.018)
$\boldsymbol{Sp_3}$	0.000 (-0.009,0.008)	0.000 (-0.009,0.008)	0.000 (-0.009,0.008)
$\boldsymbol{Sp_4}$	0.000 (-0.008,0.007)	0.000 (-0.008,0.007)	0.000 (-0.008,0.007)
**ResD**	14.69 (12.66,17.78)	14.76 (13.75,16.92)	14.69 (12.65,17.76)
**DIC**	28.02 (24.34,33.97)	28.90 (26.93,32.47)	28.01 (24.35,34.01)
**Minimum Distance**	0.333 (60% CrI)	0.334 (70% CrI)	0.335 (60% CrI)
**CIR**	0.693	0.724	0.692
**FIR**	0.129	0.188	0.131
**95% CrI Distance**	0.478	0.441	0.478
**95% CrI CIR**	0.522	0.559	0.522
**95% CrI FIR**	0.006	0.017	0.005

Table D. 24 95% CrI coverages for prevalence, sensitivities, and specificities across converged L-L models with all interaction terms within the diseased state with three different priors (Hyplerlasso, Elastic Net, Regularized Horseshoe) fitted to the 500 data sets simulated from L-L DGM in VL data setting with the sample sizes of 2000. Coverages are presented as percentages along with 95% Monte Carlo confidence intervals.

	**Sample size = 2000**		
**Parameter**	**L-L with HL priors**	**L-L with EN priors**	**L-L with RH priors**
$\boldsymbol{\pi}$	97.4 (96.0,98.8)	97.4 (96.0,98.8)	97.2 (95.8,98.6)
$\boldsymbol{Se_1}$	96.4 (94.8,98.0)	96.4 (94.8,98.0)	96.6 (95.0,98.2)
$\boldsymbol{Se_2}$	98.2 (97.0,99.4)	97.0 (95.5,98.5)	98.2 (97.0,99.4)
$\boldsymbol{Se_3}$	97.2 (95.8,98.6)	97.0 (95.5,98.5)	97.2 (95.8,98.6)
$\boldsymbol{Se_4}$	98.4 (97.3,99.5)	97.6 (96.3,98.9)	98.4 (97.3,99.5)
$\boldsymbol{Sp_1}$	98.4 (97.3,99.5)	97.0 (95.5,98.5)	98.4 (97.3,99.5)
$\boldsymbol{Sp_2}$	97.4 (96.0,98.8)	98.0 (96.8,99.2)	97.4 (96.0,98.8)
$\boldsymbol{Sp_3}$	94.8 (92.9,96.7)	95.2 (93.3,97.1)	94.6 (92.6,96.6)
$\boldsymbol{Sp_4}$	96.0 (94.3,97.7)	95.2 (93.3,97.1)	96.0 (94.3,97.7)

Table D. 25 Mean absolute biases of posterior median estimates for prevalence, sensitivities, and specificities with mean residual deviances, DIC values, CrI widths providing minimum distances, corresponding correct inclusion rates (CIR) and false inclusion rates (FIR) of the interaction terms, as well as 95% CrI distances, CIRs and FIRs across converged L-L models with all interaction terms within the diseased state with three different priors (Hyplerlasso, Elastic Net, Regularized Horseshoe) fitted to the 500 data sets simulated from L-L DGM in VL data setting with the sample sizes of 5000. Estimates are given with 2.5th and 97.5th percentiles.

	**Sample size = 5000**		
**Parameter**	**L-L with HL priors**	**L-L with EN priors**	**L-L with RH priors**
$\boldsymbol{\pi}$	0.001 (-0.016,0.017)	0.000 (-0.019,0.019)	0.001 (-0.016,0.017)
$\boldsymbol{Se_1}$	-0.003 (-0.027,0.018)	-0.002 (-0.031,0.021)	-0.002 (-0.027,0.018)
$\boldsymbol{Se_2}$	0.001 (-0.024,0.024)	0.002 (-0.023,0.026)	0.001 (-0.024,0.024)
$\boldsymbol{Se_3}$	0.001 (-0.028,0.029)	0.003 (-0.029,0.035)	0.001 (-0.028,0.029)
$\boldsymbol{Se_4}$	0.001 (-0.028,0.026)	0.003 (-0.032,0.031)	0.001 (-0.028,0.026)
$\boldsymbol{Sp_1}$	-0.002 (-0.014,0.009)	-0.002 (-0.016,0.008)	-0.002 (-0.014,0.009)
$\boldsymbol{Sp_2}$	0.000 (-0.013,0.013)	0.000 (-0.014,0.015)	0.000 (-0.013,0.013)
$\boldsymbol{Sp_3}$	0.000 (-0.005,0.005)	0.000 (-0.005,0.005)	0.000 (-0.005,0.005)
$\boldsymbol{Sp_4}$	0.000 (-0.005,0.004)	0.000 (-0.005,0.004)	0.000 (-0.005,0.004)
**ResD**	14.59 (13.12,16.34)	14.59 (14.09,15.59)	14.60 (13.05,16.31)
**DIC**	28.38 (25.26,32.34)	28.81 (27.64,30.70)	28.39 (25.09,32.35)
**Minimum Distance**	0.202 (70% CrI)	0.204 (80% CrI)	0.203 (70% CrI)
**CIR**	0.837	0.855	0.835
**FIR**	0.120	0.143	0.118
**95% CrI Distance**	0.365	0.279	0.369
**95% CrI CIR**	0.635	0.722	0.632
**95% CrI FIR**	0.008	0.025	0.008

Table D. 26 95% CrI coverages for prevalence, sensitivities, and specificities across converged L-L models with all interaction terms within the diseased state with three different priors (Hyplerlasso, Elastic Net, Regularized Horseshoe) fitted to the 500 data sets simulated from L-L DGM in VL data setting with the sample sizes of 5000. Coverages are presented as percentages along with 95% Monte Carlo confidence intervals.

	**Sample size = 5000**		
**Parameter**	**L-L with HL priors**	**L-L with EN priors**	**L-L with RH priors**
$\boldsymbol{\pi}$	98.6 (97.6,99.6)	97.8 (96.5,99.1)	98.6 (97.6,99.6)
$\boldsymbol{Se_1}$	97.6 (96.3,98.9)	97.8 (96.5,99.1)	97.6 (96.2,98.9)
$\boldsymbol{Se_2}$	97.8 (96.5,99.1)	97.4 (96.0,98.8)	97.8 (96.5,99.1)
$\boldsymbol{Se_3}$	98.0 (96.8,99.2)	97.2 (95.7,98.6)	97.8 (96.5,99.1)
$\boldsymbol{Se_4}$	98.8 (97.8,99.8)	98.0 (96.7,99.2)	98.8 (97.8,99.8)
$\boldsymbol{Sp_1}$	98.4 (97.3,99.5)	97.0 (95.5,98.5)	98.2 (97.0,99.4)
$\boldsymbol{Sp_2}$	96.8 (95.3,98.3)	95.8 (94.0,97.5)	96.8 (95.2,98.3)
$\boldsymbol{Sp_3}$	95.4 (93.6,97.2)	96.0 (94.2,97.7)	95.6 (93.8,97.4)
$\boldsymbol{Sp_4}$	95.8 (94.0,97.6)	95.8 (94.0,97.5)	95.6 (93.8,97.4)

Table D. 27 Mean absolute biases of posterior median estimates for prevalence, sensitivities, and specificities with mean residual deviances and DIC values across converged CInd and L-L models with known correlation structures fitted to the 1,250 data sets simulated from LT DGM in CPTB data setting. Estimates are given with 2.5^th^ and 97.5^th^ percentiles.

	Sample Size = 500		Sample Size = 2000		Sample Size = 5000	
**Parameter**	**CInd**	**L-L**	**CInd**	**L-L**	**CInd**	**L-L**
π	-0.139 (-0.175,-0.103)	-0.113 (-0.162,-0.037)	-0.141 (-0.162,-0.121)	-0.049 (-0.113,0.023)	-0.141 (-0.155,-0.128)	-0.020 (-0.059,0.031)
Se_1	0.392 (0.326,0.421)	0.315 (0.069,0.408)	0.408 (0.368,0.428)	0.111 (-0.051,0.317)	0.412 (0.387,0.430)	0.036 (-0.057,0.133)
Se_2	0.386 (0.259,0.498)	0.294 (0.063,0.454)	0.384 (0.308,0.469)	0.101 (-0.029,0.265)	0.381 (0.332,0.432)	0.034 (-0.041,0.111)
Se_3	0.190 (0.069,0.347)	0.142 (0.012,0.288)	0.190 (0.119,0.268)	0.047 (-0.023,0.127)	0.189 (0.139,0.240)	0.016 (-0.023,0.057)
Se_4	0.018 (-0.087,0.120)	0.024 (-0.079,0.125)	0.023 (-0.030,0.074)	0.023 (-0.036,0.079)	0.024 (-0.009,0.058)	0.014 (-0.034,0.058)
Se_5	0.019 (-0.095,0.117)	0.016 (-0.090,0.115)	0.023 (-0.034,0.078)	0.011 (-0.042,0.062)	0.024 (-0.012,0.060)	0.006 (-0.029,0.041)
Sp_1	-0.024 (-0.048,-0.006)	-0.015 (-0.036,-0.004)	-0.021 (-0.038,-0.008)	-0.005 (-0.013,-0.001)	-0.021 (-0.030,-0.011)	-0.003 (-0.008,0.000)
Sp_2	-0.007 (-0.022,0.006)	-0.004 (-0.019,0.007)	-0.007 (-0.014,0.001)	-0.001 (-0.009,0.007)	-0.007 (-0.012,-0.002)	0.000 (-0.006,0.005)
Sp_3	-0.001 (-0.007,0.001)	-0.001 (-0.007,0.001)	0.000 (-0.003,0.002)	0.000 (-0.003,0.002)	0.000 (-0.001,0.002)	0.000 (-0.002,0.002)
Sp_4	-0.056 (-0.107,-0.010)	-0.043 (-0.099,0.018)	-0.056 (-0.081,-0.032)	-0.015 (-0.054,0.024)	-0.056 (-0.072,-0.041)	0.000 (-0.027,0.019)
Sp_5	-0.071 (-0.118,-0.026)	-0.057 (-0.106,-0.005)	-0.070 (-0.094,-0.047)	-0.025 (-0.063,0.018)	-0.070 (-0.084,-0.055)	-0.010 (-0.035,0.020)
**ResD**	43.84 (29.61,62.06)	32.43 (23.64,43.72)	98.85 (70.81,131.93)	33.67 (25.40,45.44)	207.01 (162.02,255.34)	33.72 (24.21,46.13)
**DIC**	55.15 (41.09,73.18)	49.67 (38.08,65.97)	100.82 (81.65,142.79)	53.10 (43.63,66.31)	217.95 (172.90,266.45)	49.39 (39.49,62.02)

Table D. 28 95% CrI coverages for prevalence, sensitivities, and specificities across converged CInd and L-L models with known correlation structures fitted to the 1,250 data sets simulated from LT DGM in CPTB data setting. Coverages are presented as percentages along with 95% Monte Carlo confidence intervals.

	Sample Size = 500		Sample Size = 2000		Sample Size = 5000	
**Parameter**	**CInd**	**L-L**	**CInd**	**L-L**	**CInd**	**L-L**
π	0.0 (0.0,0.0)	23.3 (20.9,25.6)	0.0 (0.0,0.0)	70.4 (67.7,73.0)	0.0 (0.0,0.0)	88.1 (86.2,89.9)
Se_1	0.0 (0.0,0.0)	25.1 (22.7,27.5)	0.0 (0.0,0.0)	73.9 (71.4,76.5)	0.0 (0.0,0.0)	89.7 (88.0,91.5)
Se_2	0.0 (0.0,0.0)	23.5 (21.1,25.9)	0.0 (0.0,0.0)	69.9 (67.3,72.6)	0.0 (0.0,0.0)	88.7 (86.9,90.5)
Se_3	6.5 (5.1,7.8)	39.3 (36.5,42.0)	0.0 (0.0,0.0)	74.7 (72.2,77.2)	0.0 (0.0,0.0)	89.3 (87.5,91.1)
Se_4	94.4 (93.1,95.7)	94.8 (93.6,96.0)	86.7 (84.8,88.6)	90.2 (88.5,91.9)	72.9 (70.4,75.3)	91.4 (89.7,93.0)
Se_5	94.5 (93.2,95.7)	94.9 (93.6,96.1)	86.9 (85.0,88.8)	92.4 (90.9,93.9)	73.8 (71.3,76.2)	93.9 (92.6,95.3)
Sp_1	19.1 (16.9,21.3)	50.7 (47.9,53.5)	2.6 (1.8,3.5)	80.9 (78.6,83.2)	0.0 (0.0,0.0)	88.8 (86.9,90.6)
Sp_2	87.6 (85.8,89.4)	93.1 (91.7,94.5)	51.4 (48.7,54.2)	94.3 (92.9,95.6)	17.0 (15.0,19.1)	94.6 (93.2,95.9)
Sp_3	97.4 (96.6,98.3)	97.5 (96.6,98.4)	97.5 (96.7,98.4)	96.9 (95.9,97.9)	92.9 (91.5,94.3)	95.2 (94.0,96.5)
Sp_4	34.6 (32.0,37.3)	64.3 (61.6,67.0)	0.3 (0.0,0.6)	83.6 (81.5,85.8)	0.0 (0.0,0.0)	93.4 (92.0,94.9)
Sp_5	9.1 (7.5,10.7)	42.9 (40.2,45.7)	0.0 (0.0,0.0)	75.6 (73.1,78.1)	0.0 (0.0,0.0)	88.8 (86.9,90.6)

Table D. 29 Mean absolute biases of posterior median estimates for prevalence, sensitivities, and specificities with mean residual deviances and DIC values across converged CInd and L-L models with known correlation structures fitted to the 1,250 data sets simulated from FE DGM in CPTB data setting. Estimates are given with 2.5th and 97.5th percentiles.

	Sample Size = 500		Sample Size = 2000		Sample Size = 5000	
**Parameter**	**CInd**	**L-L**	**CInd**	**L-L**	**CInd**	**L-L**
π	-0.065 (-0.100,-0.029)	-0.053 (-0.093,-0.010)	-0.059 (-0.079,-0.040)	-0.037 (-0.066,0.002)	-0.057 (-0.070,-0.045)	-0.025 (-0.049,0.000)
Se_1	0.217 (0.123,0.286)	0.196 (0.056,0.284)	0.217 (0.167,0.265)	0.130 (-0.004,0.236)	0.215 (0.182,0.245)	0.079 (-0.002,0.172)
Se_2	0.205 (0.082,0.345)	0.154 (0.028,0.278)	0.177 (0.115,0.249)	0.092 (-0.016,0.176)	0.169 (0.127,0.216)	0.049 (-0.019,0.121)
Se_3	0.102 (-0.009,0.232)	0.075 (-0.026,0.187)	0.087 (0.033,0.152)	0.046 (-0.011,0.108)	0.081 (0.045,0.123)	0.029 (-0.013,0.072)
Se_4	-0.039 (-0.152,0.069)	-0.016 (-0.125,0.085)	-0.029 (-0.086,0.022)	0.014 (-0.046,0.069)	-0.027 (-0.060,0.005)	0.033 (-0.007,0.067)
Se_5	0.016 (-0.093,0.124)	0.010 (-0.097,0.118)	0.012 (-0.041,0.065)	0.008 (-0.044,0.058)	0.010 (-0.024,0.044)	0.007 (-0.026,0.039)
Sp_1	-0.013 (-0.036,-0.001)	-0.007 (-0.020,0.000)	-0.006 (-0.017,0.001)	-0.003 (-0.010,0.001)	-0.003 (-0.012,0.001)	-0.003 (-0.008,0.001)
Sp_2	-0.006 (-0.022,0.006)	-0.007 (-0.022,0.006)	-0.005 (-0.013,0.004)	-0.006 (-0.014,0.003)	-0.004 (-0.009,0.001)	-0.006 (-0.012,-0.001)
Sp_3	-0.004 (-0.010,-0.002)	-0.004 (-0.010,-0.002)	-0.002 (-0.005,-0.001)	-0.002 (-0.005,-0.001)	-0.001 (-0.003,0.000)	-0.001 (-0.003,0.000)
Sp_4	-0.037 (-0.083,0.009)	-0.027 (-0.074,0.023)	-0.033 (-0.058,-0.008)	-0.014 (-0.043,0.023)	-0.032 (-0.047,-0.017)	-0.004 (-0.025,0.018)
Sp_5	-0.028 (-0.072,0.016)	-0.023 (-0.067,0.020)	-0.025 (-0.047,-0.005)	-0.015 (-0.041,0.011)	-0.025 (-0.039,-0.011)	-0.010 (-0.028,0.007)
**ResD**	71.17 (47.07,98.95)	35.68 (26.62,48.53)	194.60 (148.48,245.02)	39.96 (28.82,56.06)	433.23 (356.96,517.38)	44.24 (29.39,63.09)
**DIC**	82.64 (58.44,110.54)	54.57 (43.68,68.50)	205.77 (159.65,257.15)	60.44 (47.17,78.70)	444.17 (367.87,528.32)	64.24 (48.96,83.85)

Table D. 30 95% CrI coverages for prevalence, sensitivities, and specificities across converged CInd and L-L models with known correlation structures fitted to the 1,250 data sets simulated from FE DGM in CPTB data setting. Coverages are presented as percentages along with 95% Monte Carlo confidence intervals.

	Sample Size = 500		Sample Size = 2000		Sample Size = 5000	
**Parameter**	**CInd**	**L-L**	**CInd**	**L-L**	**CInd**	**L-L**
π	11.7 (9.9,13.5)	47.2 (44.4,50.0)	0.0 (0.0,0.0)	49.3 (46.5,52.2)	0.0 (0.0,0.0)	50.1 (47.3,53.0)
Se_1	2.8 (1.9,3.7)	40.0 (37.3,42.7)	0.0 (0.0,0.0)	50.4 (47.6,53.2)	0.0 (0.0,0.0)	53.5 (50.6,56.3)
Se_2	8.6 (7.0,10.1)	44.5 (41.7,47.2)	0.0 (0.0,0.0)	60.5 (57.8,63.3)	0.0 (0.0,0.0)	72.5 (70.0,75.0)
Se_3	55.5 (52.8,58.3)	75.4 (73.0,77.7)	10.7 (9.0,12.4)	70.9 (68.4,73.5)	0.5 (0.1,0.9)	73.3 (70.8,75.9)
Se_4	87.4 (85.6,89.3)	94.4 (93.1,95.7)	77.8 (75.5,80.1)	92.3 (90.8,93.8)	63.4 (60.7,66.0)	57.5 (54.7,60.3)
Se_5	93.9 (92.6,95.2)	94.4 (93.1,95.7)	92.8 (91.4,94.2)	94.8 (93.6,96.1)	91.5 (90.0,93.1)	94.6 (93.4,95.9)
Sp_1	80.6 (78.4,82.8)	91.4 (89.9,93.0)	86.3 (84.4,88.2)	89.7 (88.0,91.4)	85.2 (83.2,87.2)	76.6 (74.2,79.0)
Sp_2	90.0 (88.3,91.7)	88.9 (87.1,90.6)	80.2 (77.9,82.4)	69.6 (67.0,72.2)	66.3 (63.7,68.9)	31.3 (28.6,33.9)
Sp_3	77.8 (75.5,80.1)	77.4 (75.0,79.7)	80.2 (77.9,82.4)	85.2 (83.2,87.2)	79.3 (77.0,81.5)	91.9 (91.4,94.3)
Sp_4	66.7 (64.1,69.3)	82.3 (80.2,84.4)	22.6 (20.2,24.9)	86.1 (84.1,88.0)	0.8 (0.3,1.3)	92.9 (91.4,94.3)
Sp_5	75.9 (73.5,78.3)	84.7 (82.7,86.7)	36.3 (33.7,39.0)	79.7 (77.5,82.0)	5.2 (4.0,6.4)	77.1 (74.7,79.5)

Table D. 31 Mean absolute biases of posterior median estimates for prevalence, sensitivities, and specificities with mean residual deviances and DIC values across converged CInd and L-L models with known correlation structures fitted to the 1,250 data sets simulated from L-L DGM in CPTB data setting. Estimates are given with 2.5th and 97.5th percentiles.

	Sample Size = 500		Sample Size = 2000		Sample Size = 5000	
**Parameter**	**CInd**	**L-L**	**CInd**	**L-L**	**CInd**	**L-L**
π	-0.061 (-0.099,-0.021)	-0.045 (-0.088,0.007)	-0.054 (-0.073,-0.034)	-0.021 (-0.054,0.020)	-0.051 (-0.064,-0.039)	-0.008 (-0.032,0.018)
Se_1	0.198 (0.116,0.276)	0.161 (-0.007,0.270)	0.195 (0.145,0.243)	0.072 (-0.054,0.188)	0.193 (0.162,0.222)	0.026 (-0.051,0.106)
Se_2	0.203 (0.081,0.333)	0.141 (0.001,0.267)	0.180 (0.121,0.245)	0.066 (-0.044,0.168)	0.171 (0.136,0.210)	0.025 (-0.040,0.092)
Se_3	0.096 (-0.012,0.238)	0.062 (-0.037,0.174)	0.081 (0.028,0.141)	0.027 (-0.035,0.094)	0.075 (0.039,0.114)	0.008 (-0.030,0.047)
Se_4	-0.085 (-0.203,0.019)	-0.059 (-0.161,0.039)	-0.077 (-0.132,-0.025)	-0.026 (-0.081,0.029)	-0.074 (-0.107,-0.043)	-0.009 (-0.043,0.025)
Se_5	0.011 (-0.096,0.113)	0.007 (-0.099,0.106)	0.007 (-0.048,0.060)	0.005 (-0.048,0.055)	0.003 (-0.030,0.037)	0.003 (-0.027,0.035)
Sp_1	-0.011 (-0.033,0.001)	-0.005 (-0.017,0.000)	-0.004 (-0.014,0.001)	-0.001 (-0.007,0.002)	-0.001 (-0.008,0.002)	-0.001 (-0.004,0.002)
Sp_2	-0.001 (-0.014,0.008)	-0.002 (-0.015,0.009)	0.001 (-0.007,0.008)	0.000 (-0.007,0.007)	0.001 (-0.003,0.006)	0.000 (-0.004,0.004)
Sp_3	-0.004 (-0.009,-0.001)	-0.004 (-0.009,-0.001)	-0.002 (-0.004,0.000)	-0.001 (-0.004,0.000)	-0.001 (-0.003,0.000)	-0.001 (-0.002,0.000)
Sp_4	-0.047 (-0.098,0.000)	-0.034 (-0.084,0.020)	-0.043 (-0.067,-0.020)	-0.016 (-0.042,0.020)	-0.042 (-0.058,-0.026)	-0.007 (-0.030,0.017)
Sp_5	-0.027 (-0.072,0.018)	-0.020 (-0.065,0.028)	-0.025 (-0.047,-0.004)	-0.009 (-0.035,0.020)	-0.025 (-0.039,-0.012)	-0.004 (-0.021,0.013)
**ResD**	71.56 (57.78,98.95)	36.18 (27.22,47.76)	191.52 (146.89,242.45)	35.39 (25.23,48.23)	429.44 (354.14,504.43)	33.26 (24.08,45.92)
**DIC**	83.22 (59.58,111.15)	55.99 (45.14,70.24)	202.89 (158.27,254.22)	55.72 (43.95,70.21)	440.57 (365.33,515.56)	52.10 (42.51,65.00)

Table D. 32 95% CrI coverages for prevalence, sensitivities, and specificities across converged CInd and L-L models with known correlation structures fitted to the 1,250 data sets simulated from L-L DGM in CPTB data setting. Coverages are presented as percentages along with 95% Monte Carlo confidence intervals.

	Sample Size = 500		Sample Size = 2000		Sample Size = 5000	
**Parameter**	**CInd**	**L-L**	**CInd**	**L-L**	**CInd**	**L-L**
π	15.1 (13.1,17.1)	63.9 (61.3,66.6)	0.0 (0.0,0.0)	79.4 (77.1,81.6)	0.0 (0.0,0.0)	90.6 (89.0,92.3)
Se_1	2.9 (2.3,3.8)	58.1 (55.3,60.8)	0.0 (0.0,0.0)	77.8 (75.5,80.2)	0.0 (0.0,0.0)	90.1 (88.5,91.8)
Se_2	8.2 (6.7,9.8)	58.6 (55.8,61.3)	0.0 (0.0,0.0)	76.9 (74.6,79.3)	0.0 (0.0,0.0)	88.8 (87.1,90.6)
Se_3	59.2 (56.5,61.9)	82.0 (79.9,84.1)	14.6 (12.7,16.6)	86.3 (84.4,88.2)	0.7 (0.3,1.2)	93.5 (92.1,94.9)
Se_4	61.3 (58.6,64.0)	79.9 (77.7,82.1)	12.6 (10.8,14.5)	84.6 (82.6,86.6)	0.2 (0.0,0.4)	90.5 (88.8,92.1)
Se_5	95.0 (93.7,96.2)	95.3 (94.1,96.5)	94.8 (93.6,96.0)	94.9 (93.6,96.1)	95.4 (94.3,96.6)	94.9 (93.6,96.1)
Sp_1	84.6 (82.6,86.6)	96.1 (95.0,97.2)	93.5 (92.2,94.9)	96.4 (95.4,97.5)	96.3 (95.3,97.4)	96.2 (95.1,97.2)
Sp_2	97.7 (96.8,98.5)	97.8 (97.0,98.6)	94.4 (93.1,95.7)	96.3 (95.2,97.3)	92.0 (90.5,93.5)	95.2 (94.0,96.4)
Sp_3	62.0 (59.3,64.7)	58.6 (55.9,61.4)	71.7 (69.2,74.2)	82.5 (80.4,84.6)	69.1 (66.6,71.7)	89.2 (87.4,90.9)
Sp_4	51.4 (48.7,54.2)	76.9 (74.5,79.2)	6.1 (4.8,7.4)	83.6 (81.5,85.7)	0.0 (0.0,0.0)	90.6 (89.0,92.3)
Sp_5	74.8 (72.4,77.2)	86.9 (85.0,88.8)	36.8 (34.1,39.5)	88.8 (87.0,90.5)	5.0 (3.8,6.2)	92.8 (91.3,94.2)

Table D. 33 Mean absolute biases of posterior median estimates for prevalence, sensitivities, and specificities with mean residual deviances, DIC values, CrI widths providing minimum distances, corresponding correct inclusion rates (CIR) and false inclusion rates (FIR) of the interaction terms, as well as 95% CrI distances, CIRs and FIRs across converged L-L models with all interaction terms within the diseased state with three different priors (Hyplerlasso, Elastic Net, Regularized Horseshoe) fitted to the 500 data sets simulated from L-L DGM in CPTB data setting with the sample sizes of 500. Estimates are given with 2.5th and 97.5th percentiles.

	**Sample size = 500**		
**Parameter**	**L-L with HL priors**	**L-L with EN priors**	**L-L with RH priors**
π	-0.048 (-0.088,0.003)	-0.047 (-0.087,0.005)	-0.047 (-0.088,0.002)
Se_1	0.172 (0.010,0.273)	0.166 (-0.005,0.274)	0.170 (0.008,0.271)
Se_2	0.150 (0.004,0.268)	0.146 (-0.009,0.267)	0.149 (0.006,0.267)
Se_3	0.065 (-0.042,0.174)	0.064 (-0.042,0.173)	0.065 (-0.040,0.172)
Se_4	-0.057 (-0.150,0.033)	-0.056 (-0.148,0.033)	-0.057 (-0.149,0.033)
Se_5	0.013 (-0.093,0.118)	0.014 (-0.090,0.117)	0.013 (-0.093,0.117)
Sp_1	-0.006 (-0.018,0.000)	-0.006 (-0.018,0.000)	-0.006 (-0.018,0.000)
Sp_2	-0.002 (-0.015,0.008)	-0.002 (-0.015,0.008)	-0.002 (-0.015,0.008)
Sp_3	-0.004 (-0.009,-0.001)	-0.004 (-0.009,-0.001)	-0.004 (-0.009,-0.001)
Sp_4	-0.035 (-0.082,0.018)	-0.034 (-0.083,0.021)	-0.035 (-0.083,0.018)
Sp_5	-0.021 (-0.066,0.030)	-0.020 (-0.066,0.033)	-0.021 (-0.066,0.031)
**ResD**	34.80 (27.22,44.85)	35.33 (28.02,45.01)	34.69 (26.98,44.75)
**DIC**	57.32 (46.60,74.05)	60.32 (48.86,78.16)	56.63 (46.17,69.84)
**Minimum Distance**	0.397 (40% CrI)	0.479 (50% CrI)	0.477 (40% CrI)
**CIR**	0.644	0.660	0.671
**FIR**	0.308	0.337	0.346
**95% CrI Distance**	0.768	0.748	0.756
**95% CrI CIR**	0.232	0.252	0.244
**95% CrI FIR**	0.004	0.010	0.004

Table D. 34 95% CrI coverages for prevalence, sensitivities, and specificities across converged L-L models with all interaction terms within the diseased state with three different priors (Hyplerlasso, Elastic Net, Regularized Horseshoe) fitted to the 500 data sets simulated from L-L DGM in CPTB data setting with the sample sizes of 500. Coverages are presented as percentages along with 95% Monte Carlo confidence intervals.

	**Sample size = 500**		
**Parameter**	**L-L with HL priors**	**L-L with EN priors**	**L-L with RH priors**
$\boldsymbol{\pi}$	56.9 (52.6,61.3)	59.4 (55.1,63.8)	58.0 (53.7,62.3)
$\boldsymbol{Se_1}$	51.3 (46.9,55.7)	56.0 (51.7,60.4)	52.0 (47.6,56.4)
$\boldsymbol{Se_2}$	52.9 (48.5,57.3)	54.8 (50.4,59.2)	53.2 (48.8,57.6)
$\boldsymbol{Se_3}$	81.0 (77.5,84.4)	81.3 (77.9,84.7)	81.0 (77.6,84.4)
$\boldsymbol{Se_4}$	83.0 (79.7,86.3)	83.1 (79.8,86.4)	82.8 (79.5,86.1)
$\boldsymbol{Se_5}$	94.8 (92.8,96.7)	94.8 (92.8,96.7)	95.0 (93.1,96.9)
$\boldsymbol{Sp_1}$	95.4 (93.6,97.2)	95.4 (93.5,97.2)	96.0 (94.3,97.7)
$\boldsymbol{Sp_2}$	98.2 (97.0,99.4)	98.4 (97.3,99.5)	98.2 (97.0,99.4)
$\boldsymbol{Sp_3}$	53.7 (49.3,58.1)	55.6 (51.3,60.0)	54.4 (50.0,58.8)
$\boldsymbol{Sp_4}$	75.8 (72.0,79.5)	76.9 (73.2,80.6)	75.8 (72.0,79.6)
$\boldsymbol{Sp_5}$	84.6 (81.4,87.7)	85.3 (82.2,88.4)	85.2 (82.1,88.3)

Table D. 35 Mean absolute biases of posterior median estimates for prevalence, sensitivities, and specificities with mean residual deviances, DIC values, CrI widths providing minimum distances, corresponding correct inclusion rates (CIR) and false inclusion rates (FIR) of the interaction terms, as well as 95% CrI distances, CIRs and FIRs across converged L-L models with all interaction terms within the diseased state with three different priors (Hyplerlasso, Elastic Net, Regularized Horseshoe) fitted to the 500 data sets simulated from L-L DGM in CPTB data setting with the sample sizes of 2000. Estimates are given with 2.5th and 97.5th percentiles.

	**Sample size = 2000**		
**Parameter**	**L-L with HL priors**	**L-L with EN priors**	**L-L with RH priors**
$\boldsymbol{\pi}$	-0.023 (-0.056,0.016)	-0.023 (-0.054,0.014)	-0.023 (-0.056,0.015)
$\boldsymbol{Se_1}$	0.078 (-0.046,0.205)	0.075 (-0.041,0.195)	0.078 (-0.042,0.201)
$\boldsymbol{Se_2}$	0.069 (-0.041,0.176)	0.068 (-0.036,0.171)	0.069 (-0.039,0.174)
$\boldsymbol{Se_3}$	0.028 (-0.033,0.093)	0.028 (-0.031,0.091)	0.028 (-0.033,0.092)
$\boldsymbol{Se_4}$	-0.026 (-0.085,0.028)	-0.024 (-0.082,0.029)	-0.026 (-0.085,0.028)
$\boldsymbol{Se_5}$	0.010 (-0.048,0.057)	0.011 (-0.048,0.062)	0.010 (-0.048,0.058)
$\boldsymbol{Sp_1}$	-0.001 (-0.007,0.002)	-0.002 (-0.007,0.001)	-0.001 (-0.006,0.001)
$\boldsymbol{Sp_2}$	0.000 (-0.008,0.007)	0.000 (-0.007,0.007)	0.000 (-0.008,0.007)
$\boldsymbol{Sp_3}$	-0.002 (-0.004,0.000)	-0.002 (-0.004,0.000)	-0.002 (-0.004,0.000)
$\boldsymbol{Sp_4}$	-0.017 (-0.049,0.019)	-0.017 (-0.048,0.020)	-0.017 (-0.049,0.019)
$\boldsymbol{Sp_5}$	-0.008 (-0.035,0.022)	-0.008 (-0.034,0.021)	-0.008 (-0.035,0.021)
**ResD**	34.85 (26.42,46.32)	34.68 (26.75,46.00)	34.76 (26.40,46.51)
**DIC**	58.24 (48.15,71.17)	57.99 (48.49,70.35)	57.97 (48.00,70.87)
**Minimum Distance**	0.350 (60% CrI)	0.342 (80% CrI)	0.351 (70% CrI)
**CIR**	0.762	0.711	0.706
**FIR**	0.256	0.184	0.192
**95% CrI Distance**	0.539	0.462	0.526
**95% CrI CIR**	0.462	0.539	0.474
**95% CrI FIR**	0.018	0.042	0.020

Table D. 36 95% CrI coverages for prevalence, sensitivities, and specificities across converged L-L models with all interaction terms within the diseased state with three different priors (Hyplerlasso, Elastic Net, Regularized Horseshoe) fitted to the 500 data sets simulated from L-L DGM in CPTB data setting with the sample sizes of 2000. Coverages are presented as percentages along with 95% Monte Carlo confidence intervals.

	**Sample size = 2000**		
**Parameter**	**L-L with HL priors**	**L-L with EN priors**	**L-L with RH priors**
$\boldsymbol{\pi}$	81.2 (77.8,84.6)	80.7 (77.3,84.2)	81.0 (77.6,84.4)
$\boldsymbol{Se_1}$	80.6 (77.1,84.1)	81.1 (77.7,84.6)	80.0 (76.5,83.5)
$\boldsymbol{Se_2}$	80.8 (77.3,84.3)	80.7 (77.3,84.2)	80.6 (77.1,84.1)
$\boldsymbol{Se_3}$	87.0 (84.1,89.9)	87.1 (84.2,90.1)	87.2 (84.3,90.1)
$\boldsymbol{Se_4}$	85.8 (82.7,88.9)	86.5 (83.5,89.5)	85.8 (82.7,88.9)
$\boldsymbol{Se_5}$	96.6 (95.0,98.2)	96.0 (94.3,97.7)	96.8 (95.3,98.3)
$\boldsymbol{Sp_1}$	98.0 (96.8,99.2)	98.0 (96.8,99.2)	98.2 (97.0,99.4)
$\boldsymbol{Sp_2}$	95.6 (93.8,97.4)	96.4 (94.7,98.0)	95.6 (93.8,97.4)
$\boldsymbol{Sp_3}$	81.8 (78.4,85.2)	79.7 (76.2,83.3)	82.0 (78.6,85.4)
$\boldsymbol{Sp_4}$	85.2 (82.1,88.3)	85.7 (82.7,88.8)	85.0 (81.9,88.1)
$\boldsymbol{Sp_5}$	88.8 (86.0,91.6)	89.4 (86.6,92.1)	89.0 (86.3,91.7)

Table D. 37 Mean absolute biases of posterior median estimates for prevalence, sensitivities, and specificities with mean residual deviances, DIC values, CrI widths providing minimum distances, corresponding correct inclusion rates (CIR) and false inclusion rates (FIR) of the interaction terms, as well as 95% CrI distances, CIRs and FIRs across converged L-L models with all interaction terms within the diseased state with three different priors (Hyplerlasso, Elastic Net, Regularized Horseshoe) fitted to the 500 data sets simulated from L-L DGM in CPTB data setting with the sample sizes of 5000. Estimates are given with 2.5th and 97.5th percentiles.

	**Sample size = 5000**		
**Parameter**	**L-L with HL priors**	**L-L with EN priors**	**L-L with RH priors**
π	-0.009 (-0.033,0.018)	-0.010 (-0.034,0.017)	-0.009 (-0.033,0.017)
Se_1	0.029 (-0.043,0.113)	0.033 (-0.040,0.115)	0.030 (-0.042,0.115)
Se_2	0.026 (-0.038,0.096)	0.030 (-0.035,0.097)	0.027 (-0.037,0.097)
Se_3	0.009 (-0.030,0.049)	0.011 (-0.028,0.050)	0.010 (-0.030,0.049)
Se_4	-0.010 (-0.044,0.024)	-0.010 (-0.044,0.025)	-0.010 (-0.044,0.024)
Se_5	0.006 (-0.026,0.042)	0.008 (-0.026,0.045)	0.007 (-0.026,0.042)
Sp_1	-0.001 (-0.004,0.002)	-0.001 (-0.004,0.002)	-0.001 (-0.004,0.002)
Sp_2	0.000 (-0.005,0.004)	0.000 (-0.005,0.004)	0.000 (-0.005,0.004)
Sp_3	-0.001 (-0.002,0.000)	-0.001 (-0.002,0.000)	-0.001 (-0.002,0.000)
Sp_4	-0.007 (-0.032,0.018)	-0.008 (-0.032,0.018)	-0.008 (-0.032,0.018)
Sp_5	-0.003 (-0.020,0.013)	-0.004 (-0.020,0.013)	-0.003 (-0.020,0.013)
**ResD**	32.22 (24.65,41.33)	32.48 (25.12,41.54)	32.23 (24.71,41.26)
**DIC**	53.68 (45.60,63.24)	54.34 (46.35,63.45)	53.68 (45.76,63.06)
**Minimum Distance**	0.307 (80% CrI)	0.301 (90% CrI)	0.310 (80% CrI)
**CIR**	0.816	0.796	0.823
**FIR**	0.246	0.221	0.255
**95% CrI Distance**	0.350	0.307	0.345
**95% CrI CIR**	0.671	0.741	0.677
**95% CrI FIR**	0.118	0.164	0.122

Table D. 38 95% CrI coverages for prevalence, sensitivities, and specificities across converged L-L models with all interaction terms within the diseased state with three different priors (Hyplerlasso, Elastic Net, Regularized Horseshoe) fitted to the 500 data sets simulated from L-L DGM in CPTB data setting with the sample sizes of 5000. Coverages are presented as percentages along with 95% Monte Carlo confidence intervals.

	**Sample size = 5000**		
**Parameter**	**L-L with HL priors**	**L-L with EN priors**	**L-L with RH priors**
π	92.4 (90.1,94.7)	91.4 (88.9,93.9)	92.2 (89.8,94.6)
Se_1	91.0 (88.5,93.5)	90.0 (87.4,92.6)	90.4 (87.8,93.0)
Se_2	91.0 (88.5,93.5)	89.8 (87.1,92.5)	90.8 (88.3,93.3)
Se_3	93.6 (91.5,95.7)	93.6 (91.5,95.7)	93.4 (91.2,95.6)
Se_4	90.8 (88.3,93.3)	91.6 (89.2,94.0)	90.6 (88.0,93.2)
Se_5	97.2 (95.8,98.6)	96.2 (94.5,97.9)	97.2 (95.8,98.6)
Sp_1	97.4 (96.0,98.8)	96.2 (94.5,97.9)	97.4 (96.0,98.8)
Sp_2	95.4 (93.6,97.2)	95.0 (93.1,96.9)	95.4 (93.6,97.2)
Sp_3	91.8 (89.4,94.2)	87.8 (84.9,90.7)	91.4 (88.9,93.9)
Sp_4	90.4 (87.8,93.0)	89.4 (86.7,92.1)	90.2 (87.6,92.8)
Sp_5	93.4 (91.2,95.6)	92.6 (90.3,94.9)	93.4 (91.2,95.6)

Table D. 39 Mean absolute biases of posterior median estimates for prevalence, sensitivities, and specificities with mean residual deviances and DIC values across converged CInd and L-L models with known correlation structures fitted to the 1,250 data sets simulated from LT DGM in CDPN data setting. Estimates are given with 2.5th and 97.5th percentiles.

	Sample Size = 500		Sample Size = 2000		Sample Size = 5000	
**Parameter**	**CInd**	**L-L**	**CInd**	**L-L**	**CInd**	**L-L**
π	-0.070 (-0.126,-0.011)	-0.024 (-0.135,0.055)	-0.067 (-0.096,-0.039)	-0.019 (-0.092,0.040)	-0.067 (-0.084,-0.048)	-0.021 (-0.069,0.018)
Se_1	0.117 (0.044,0.186)	0.043 (-0.037,0.128)	0.116 (0.079,0.152)	0.016 (-0.026,0.061)	0.116 (0.093,0.139)	0.006 (-0.021,0.036)
Se_2	0.117 (0.044,0.188)	0.043 (-0.033,0.130)	0.116 (0.083,0.152)	0.017 (-0.025,0.064)	0.116 (0.094,0.138)	0.007 (-0.018,0.038)
Se_3	0.117 (0.046,0.188)	0.043 (-0.031,0.129)	0.116 (0.081,0.153)	0.017 (-0.025,0.065)	0.116 (0.093,0.139)	0.006 (-0.018,0.039)
Se_4	0.116 (0.045,0.189)	0.042 (-0.035,0.127)	0.116 (0.078,0.153)	0.016 (-0.026,0.059)	0.116 (0.093,0.139)	0.006 (-0.020,0.037)
Se_5	-0.035 (-0.104,0.032)	-0.018 (-0.102,0.096)	-0.036 (-0.073,0.001)	0.004 (-0.066,0.100)	-0.036 (-0.058,-0.015)	0.018 (-0.029,0.083)
Sp_1	0.021 (-0.021,0.059)	0.014 (-0.070,0.069)	0.024 (0.004,0.044)	-0.002 (-0.069,0.048)	0.024 (0.011,0.037)	-0.015 (-0.055,0.019)
Sp_2	0.021 (-0.022,0.060)	0.015 (-0.075,0.072)	0.024 (0.003,0.044)	-0.001 (-0.069,0.048)	0.024 (0.012,0.036)	-0.015 (-0.052,0.019)
Sp_3	0.021 (-0.020,0.060)	0.015 (-0.067,0.070)	0.023 (0.003,0.044)	-0.002 (-0.068,0.045)	0.024 (0.011,0.037)	-0.015 (-0.055,0.020)
Sp_4	0.021 (-0.021,0.058)	0.015 (-0.071,0.070)	0.024 (0.004,0.044)	-0.001 (-0.068,0.049)	0.024 (0.011,0.037)	-0.015 (-0.054,0.020)
Sp_5	-0.106 (-0.153,-0.061)	-0.048 (-0.095,-0.011)	-0.103 (-0.128,-0.081)	-0.020 (-0.049,-0.002)	-0.103 (-0.118,-0.089)	-0.009 (-0.033,0.003)
**ResD**	55.61 (35.84,80.38)	34.08 (22.61,48.69)	127.74 (90.57,170.11)	35.46 (23.70,50.69)	217.19 (209.10,331.91)	38.50 (25.70,56.65)
**DIC**	66.52 (46.73,91.30)	47.86 (35.58,63.36)	138.70 (101.60,181.02)	49.81 (37.79,65.40)	282.17 (220.02,343.05)	52.30 (39.36,70.75)

Table D. 40 95% CrI coverages for prevalence, sensitivities, and specificities across converged CInd and L-L models with known correlation structures fitted to the 1,250 data sets simulated from LT DGM in CDPN data setting. Coverages are presented as percentages along with 95% Monte Carlo confidence intervals.

	Sample Size = 500		Sample Size = 2000		Sample Size = 5000	
**Parameter**	**CInd**	**L-L**	**CInd**	**L-L**	**CInd**	**L-L**
π	31.1 (28.6,33.7)	95.2 (94.1,96.4)	0.2 (0.0,0.5)	96.2 (95.1,97.3)	0.0 (0.0,0.0)	97.6 (96.7,98.4)
Se_1	9.8 (8.2,11.5)	88.6 (86.9,90.4)	0.0 (0.0,0.0)	92.3 (90.8,93.9)	0.0 (0.0,0.0)	96.1 (94.9,97.2)
Se_2	8.1 (6.6,9.6)	89.4 (87.7,91.2)	0.0 (0.0,0.0)	93.5 (92.0,94.9)	0.0 (0.0,0.0)	96.5 (95.4,97.6)
Se_3	9.5 (7.9,11.1)	89.4 (87.7,91.2)	0.0 (0.0,0.0)	91.2 (89.5,92.8)	0.0 (0.0,0.0)	95.1 (93.8,96.4)
Se_4	13.0 (11.1,14.8)	88.6 (86.8,90.3)	0.0 (0.0,0.0)	93.1 (91.7,94.6)	0.0 (0.0,0.0)	95.8 (94.6,97.0)
Se_5	82.2 (80.0,84.3)	98.0 (97.2,98.8)	47.1 (44.4,49.9)	99.5 (99.1,99.9)	10.1 (8.4,11.7)	99.0 (98.5,99.6)
Sp_1	81.8 (79.7,84.0)	97.0 (96.1,98.0)	38.6 (35.9,41.3)	99.6 (99.2,99.9)	5.4 (4.2,6.7)	99.3 (98.8,99.8)
Sp_2	81.6 (79.5,83.7)	97.2 (96.3,98.1)	37.2 (34.5,39.9)	99.3 (98.8,99.8)	4.6 (3.4,5.7)	99.6 (99.2,99.9)
Sp_3	83.8 (81.7,85.8)	97.7 (96.9,98.6)	39.8 (37.1,42.6)	99.4 (99.0,99.8)	5.4 (4.2,6.7)	99.1 (98.6,99.7)
Sp_4	81.6 (79.5,83.7)	97.5 (96.6,98.4)	37.8 (35.1,40.4)	99.4 (99.0,99.8)	5.0 (3.8,6.2)	98.9 (98.2,99.5)
Sp_5	0.0 (0.0,0.0)	74.8 (72.4,77.2)	0.0 (0.0,0.0)	96.0 (94.9,97.1)	0.0 (0.0,0.0)	97.5 (96.6,98.4)

Table D. 41 Mean absolute biases of posterior median estimates for prevalence, sensitivities, and specificities with mean residual deviances and DIC values across converged CInd and L-L models with known correlation structures fitted to the 1,250 data sets simulated from FE DGM in CDPN data setting. Estimates are given with 2.5th and 97.5th percentiles.

	Sample Size = 500		Sample Size = 2000		Sample Size = 5000	
**Parameter**	**CInd**	**L-L**	**CInd**	**L-L**	**CInd**	**L-L**
π	-0.118 (-0.176,-0.049)	-0.021 (-0.121,0.060)	-0.120 (-0.151,-0.085)	-0.027 (-0.090,0.026)	-0.121 (-0.142,-0.100)	-0.064 (-0.135,-0.003)
Se_1	0.152 (0.064,0.230)	0.020 (-0.055,0.100)	0.156 (0.112,0.196)	-0.002 (-0.039,0.035)	0.158 (0.131,0.184)	-0.006 (-0.029,0.017)
Se_2	0.154 (0.066,0.232)	0.021 (-0.057,0.104)	0.156 (0.110,0.194)	-0.003 (-0.042,0.034)	0.158 (0.132,0.184)	-0.006 (-0.029,0.016)
Se_3	0.152 (0.061,0.230)	0.021 (-0.058,0.110)	0.157 (0.111,0.198)	-0.001 (-0.037,0.033)	0.158 (0.130,0.184)	-0.006 (-0.028,0.017)
Se_4	0.154 (0.067,0.229)	0.021 (-0.055,0.108)	0.156 (0.114,0.198)	-0.002 (-0.037,0.036)	0.158 (0.130,0.184)	-0.006 (-0.029,0.017)
Se_5	-0.015 (-0.094,0.059)	0.002 (-0.094,0.122)	-0.016 (-0.051,0.020)	0.039 (-0.032,0.133)	-0.015 (-0.039,0.009)	0.107 (0.013,0.245)
Sp_1	-0.011 (-0.059,0.034)	-0.004 (-0.082,0.058)	-0.011 (-0.034,0.015)	-0.029 (-0.084,0.018)	-0.011 (-0.026,0.003)	-0.065 (-0.123,-0.011)
Sp_2	-0.012 (-0.062,0.039)	-0.004 (-0.084,0.058)	-0.010 (-0.034,0.013)	-0.028 (-0.087,0.020)	-0.011 (-0.026,0.004)	-0.065 (-0.122,-0.011)
Sp_3	-0.011 (-0.058,0.035)	-0.004 (-0.082,0.056)	-0.011 (-0.035,0.013)	-0.029 (-0.086,0.020)	-0.011 (-0.027,0.004)	-0.065 (-0.122,-0.010)
Sp_4	-0.011 (-0.061,0.038)	-0.004 (-0.081,0.062)	-0.011 (-0.035,0.013)	-0.029 (-0.084,0.018)	-0.011 (-0.026,0.003)	-0.065 (-0.121,-0.009)
Sp_5	-0.133 (-0.185,-0.078)	-0.025 (-0.073,-0.003)	-0.133 (-0.159,-0.106)	0.003 (-0.001,0.005)	-0.134 (-0.151,-0.116)	0.007 (0.006,0.008)
**ResD**	78.72 (52.76,110.84)	45.23 (29.33,66.89)	219.58 (168.86,272.31)	71.64 (46.29,98.87)	503.37 (420.95,584.08)	128.26 (91.47,175.28)
**DIC**	89.58 (93.74,121.63)	60.88 (44.09,84.28)	230.55 (179.87,283.29)	86.66 (61.63,114.12)	514.36 (431.94,595.14)	143.30 (106.75,189.98)

Table D. 42 95% CrI coverages for prevalence, sensitivities, and specificities across converged CInd and L-L models with known correlation structures fitted to the 1,250 data sets simulated from FE DGM in CDPN data setting. Coverages are presented as percentages along with 95% Monte Carlo confidence intervals.

	Sample Size = 500		Sample Size = 2000		Sample Size = 5000	
**Parameter**	**CInd**	**L-L**	**CInd**	**L-L**	**CInd**	**L-L**
π	5.8 (4.5,7.1)	95.9 (94.8,97.0)	0.0 (0.0,0.0)	96.4 (95.3,97.5)	0.0 (0.0,0.0)	84.1 (82.0,86.1)
Se_1	4.5 (3.3,5.6)	95.3 (94.2,96.5)	0.0 (0.0,0.0)	95.8 (94.7,97.0)	0.0 (0.0,0.0)	92.1 (90.5,93.6)
Se_2	4.4 (3.3,5.5)	94.7 (93.4,95.9)	0.0 (0.0,0.0)	94.6 (93.3,95.9)	0.0 (0.0,0.0)	92.2 (90.7,93.8)
Se_3	5.0 (3.8,6.3)	94.9 (93.7,96.2)	0.0 (0.0,0.0)	97.0 (96.0,98.0)	0.0 (0.0,0.0)	93.4 (92.0,94.8)
Se_4	4.4 (3.3,5.5)	94.7 (93.4,95.9)	0.0 (0.0,0.0)	96.0 (94.9,97.2)	0.0 (0.0,0.0)	93.0 (91.5,94.4)
Se_5	92.3 (90.8,93.8)	97.7 (96.8,98.5)	86.2 (84.3,88.1)	95.4 (94.2,96.6)	74.9 (72.5,77.3)	73.9 (71.4,76.4)
Sp_1	89.3 (87.6,91.0)	97.1 (96.2,98.0)	79.0 (76.7,81.2)	93.9 (92.5,95.3)	61.3 (58.6,64.0)	67.8 (65.2,70.5)
Sp_2	88.2 (86.4,90.0)	96.8 (95.8,97.8)	81.7 (79.5,83.8)	95.0 (93.7,96.2)	63.0 (60.4,65.7)	68.5 (65.8,71.1)
Sp_3	89.8 (88.1,91.4)	98.0 (97.2,98.8)	79.1 (76.9,81.4)	94.8 (93.5,96.1)	60.9 (58.2,63.6)	69.4 (66.8,72.0)
Sp_4	88.6 (86.8,90.3)	97.2 (96.3,98.1)	80.9 (78.7,83.1)	94.1 (92.7,95.5)	64.2 (61.5,66.8)	70.1 (67.6,72.7)
Sp_5	0.0 (0.0,0.0)	98.5 (97.8,99.2)	0.0 (0.0,0.0)	100.0 (100.0,100.0)	0.0 (0.0,0.0)	98.3 (97.6,99.1)

Table D. 43 Mean absolute biases of posterior median estimates for prevalence, sensitivities, and specificities with mean residual deviances and DIC values across converged CInd and L-L models with known correlation structures fitted to the 1,250 data sets simulated from L-L DGM in CDPN data setting. Estimates are given with 2.5th and 97.5th percentiles.

	Sample Size = 500		Sample Size = 2000		Sample Size = 5000	
**Parameter**	**CInd**	**L-L**	**CInd**	**L-L**	**CInd**	**L-L**
π	-0.088 (-0.142,-0.031)	-0.048 (-0.120,0.024)	-0.086 (-0.114,-0.057)	-0.022 (-0.062,0.017)	-0.086 (-0.103,-0.068)	-0.012 (-0.041,0.014)
Se_1	0.124 (0.055,0.194)	0.060 (-0.019,0.141)	0.124 (0.089,0.159)	0.028 (-0.014,0.076)	0.125 (0.102,0.148)	0.017 (-0.013,0.053)
Se_2	0.125 (0.054,0.196)	0.061 (-0.022,0.151)	0.124 (0.088,0.159)	0.029 (-0.016,0.076)	0.124 (0.103,0.147)	0.017 (-0.012,0.051)
Se_3	0.123 (0.054,0.195)	0.059 (-0.018,0.146)	0.123 (0.089,0.157)	0.028 (-0.013,0.075)	0.125 (0.102,0.147)	0.017 (-0.012,0.052)
Se_4	0.123 (0.053,0.190)	0.059 (-0.021,0.139)	0.124 (0.090,0.159)	0.028 (-0.013,0.074)	0.124 (0.101,0.147)	0.017 (-0.012,0.052)
Se_5	-0.015 (-0.084,0.055)	0.000 (-0.080,0.086)	-0.017 (-0.050,0.021)	-0.003 (-0.044,0.043)	-0.017 (-0.039,0.005)	-0.002 (-0.029,0.024)
Sp_1	0.004 (-0.042,0.044)	-0.002 (-0.059,0.050)	0.006 (-0.015,0.026)	0.002 (-0.029,0.028)	0.007 (-0.007,0.020)	0.002 (-0.017,0.019)
Sp_2	0.005 (-0.037,0.047)	-0.001 (-0.053,0.048)	0.007 (-0.015,0.026)	0.002 (-0.024,0.029)	0.006 (-0.007,0.020)	0.002 (-0.017,0.020)
Sp_3	0.004 (-0.037,0.043)	-0.002 (-0.057,0.048)	0.006 (-0.016,0.026)	0.002 (-0.027,0.028)	0.007 (-0.006,0.019)	0.001 (-0.018,0.018)
Sp_4	0.004 (-0.036,0.045)	-0.002 (-0.052,0.047)	0.006 (-0.014,0.027)	0.002 (-0.030,0.029)	0.007 (-0.007,0.019)	0.002 (-0.017,0.019)
Sp_5	-0.107 (-0.152,-0.063)	-0.056 (-0.109,-0.015)	-0.106 (-0.128,-0.082)	-0.029 (-0.064,-0.005)	-0.106 (-0.120,-0.092)	-0.017 (-0.045,0.000)
**ResD**	47.28 (29.62,69.61)	32.77 (21.85,48.31)	94.21 (64.07,129.88)	32.18 (21.44,45.83)	187.92 (138.92,245.85)	31.51 (21.61,45.27)
**DIC**	58.17 (40.46,80.53)	45.36 (33.86,60.66)	105.17 (75.03,140.86)	44.67 (33.82,58.28)	198.91 (149.87,256.85)	43.80 (33.98,57.62)

Table D. 44 95% CrI coverages for prevalence, sensitivities, and specificities across converged CInd and L-L models with known correlation structures fitted to the 1,250 data sets simulated from L-L DGM in CDPN data setting. Coverages are presented as percentages along with 95% Monte Carlo confidence intervals.

	Sample Size = 500		Sample Size = 2000		Sample Size = 5000	
**Parameter**	**CInd**	**L-L**	**CInd**	**L-L**	**CInd**	**L-L**
π	11.4 (9.7,13.2)	80.2 (78.0,82.4)	0.0 (0.0,0.0)	87.3 (85.5,89.2)	0.0 (0.0,0.0)	89.0 (87.3,90.8)
Se_1	8.7 (7.2,10.3)	78.9 (76.6,81.1)	0.0 (0.0,0.0)	85.9 (83.9,87.8)	0.0 (0.0,0.0)	88.8 (87.0,90.6)
Se_2	7.7 (6.2,9.2)	77.1 (74.8,79.4)	0.0 (0.0,0.0)	85.0 (83.0,87.0)	0.0 (0.0,0.0)	89.4 (87.6,91.1)
Se_3	9.1 (7.5,10.7)	79.9 (77.7,82.1)	0.0 (0.0,0.0)	85.5 (83.5,87.4)	0.0 (0.0,0.0)	89.4 (87.6,91.1)
Se_4	7.6 (6.1,9.1)	80.0 (77.8,92.2)	0.0 (0.0,0.0)	86.4 (84.5,88.3)	0.0 (0.0,0.0)	89.6 (87.9,91.3)
Se_5	92.5 (91.0,93.9)	95.8 (94.6,96.9)	86.0 (84.1,87.9)	94.2 (92.9,95.5)	69.8 (67.2,72.3)	95.2 (94.0,96.4)
Sp_1	93.0 (91.5,94.4)	94.2 (92.9,95.5)	90.1 (88.4,91.7)	94.6 (93.3,95.8)	82.2 (80.1,84.4)	95.4 (94.2,96.6)
Sp_2	93.7 (92.3,95.0)	95.5 (94.4,96.7)	89.4 (87.7,91.1)	95.4 (94.2,96.5)	83.7 (81.6,85.7)	95.6 (94.5,96.8)
Sp_3	94.4 (93.1,95.7)	95.6 (94.5,96.7)	90.8 (89.2,92.4)	94.9 (93.7,96.1)	83.3 (81.2,85.3)	96.4 (95.3,97.4)
Sp_4	94.5 (93.2,95.7)	96.2 (95.1,97.2)	89.5 (87.8,91.2)	94.2 (92.9,95.5)	79.1 (76.9,81.4)	95.0 (93.8,96.3)
Sp_5	0.0 (0.0,0.0)	61.7 (59.0,64.4)	0.0 (0.0,0.0)	87.0 (85.1,88.9)	0.0 (0.0,0.0)	91.4 (89.8,93.0)

Table D. 45 Mean absolute biases of posterior median estimates for prevalence, sensitivities, and specificities with mean residual deviances, DIC values, CrI widths providing minimum distances, corresponding correct inclusion rates (CIR) and false inclusion rates (FIR) of the interaction terms, as well as 95% CrI distances, CIRs and FIRs across converged L-L models with all interaction terms within both diseased and disease-free states with three different priors (Hyplerlasso, Elastic Net, Regularized Horseshoe) fitted to the 500 data sets simulated from L-L DGM in CDPN data setting with the sample sizes of 500. Estimates are given with 2.5th and 97.5th percentiles.

	**Sample size = 500**		
**Parameter**	**L-L with HL priors**	**L-L with EN priors**	**L-L with RH priors**
$\boldsymbol{\pi}$	-0.076 (-0.137,-0.012)	-0.075 (-0.136,-0.011)	-0.072 (-0.134,-0.005)
$\boldsymbol{Se_1}$	0.111 (0.043,0.187)	0.111 (0.041,0.186)	0.105 (0.037,0.181)
$\boldsymbol{Se_2}$	0.108 (0.035,0.182)	0.109 (0.033,0.186)	0.103 (0.031,0.180)
$\boldsymbol{Se_3}$	0.108 (0.034,0.180)	0.107 (0.035,0.180)	0.102 (0.029,0.173)
$\boldsymbol{Se_4}$	0.108 (0.037,0.182)	0.108 (0.037,0.182)	0.102 (0.031,0.179)
$\boldsymbol{Se_5}$	-0.021 (-0.092,0.044)	-0.021 (-0.090,0.046)	-0.024 (-0.096,0.042)
$\boldsymbol{Sp_1}$	0.006 (-0.043,0.049)	0.005 (-0.046,0.050)	0.005 (-0.048,0.050)
$\boldsymbol{Sp_2}$	0.006 (-0.036,0.045)	0.005 (-0.036,0.046)	0.005 (-0.038,0.046)
$\boldsymbol{Sp_3}$	0.004 (-0.041,0.048)	0.004 (-0.043,0.046)	0.003 (-0.043,0.046)
$\boldsymbol{Sp_4}$	0.005 (-0.037,0.046)	0.004 (-0.039,0.047)	0.004 (-0.040,0.046)
$\boldsymbol{Sp_5}$	-0.102 (-0.157,-0.056)	-0.102 (-0.161,-0.051)	-0.101 (-0.161,-0.053)
**ResD**	34.35 (27.04,43.19)	34.24 (27.67,42.62)	31.67 (25.51,39.43)
**DIC**	60.06 (42.56,77.57)	61.84 (41.99,78.30)	54.89 (43.34,68.52)
**Minimum Distance**	0.672 (30% CrI)	0.683 (30% CrI)	0.656 (40% CrI)
**CIR**	0.417	0.444	0.439
**FIR**	0.334	0.396	0.338
**95% CrI Distance**	0.996	0.995	0.992
**95% CrI CIR**	0.004	0.005	0.008
**95% CrI FIR**	0.001	0.001	0.002

Table D. 46 95% CrI coverages for prevalence, sensitivities, and specificities across converged L-L models with all interaction terms within both diseased and disease-free states with three different priors (Hyplerlasso, Elastic Net, Regularized Horseshoe) fitted to the 500 data sets simulated from L-L DGM in CDPN data setting with the sample sizes of 500. Coverages are presented as percentages along with 95% Monte Carlo confidence intervals.

	**Sample size = 500**		
**Parameter**	**L-L with HL priors**	**L-L with EN priors**	**L-L with RH priors**
$\boldsymbol{\pi}$	49.0 (44.6,53.4)	52.7 (48.1,57.3)	64.0 (59.8,68.3)
$\boldsymbol{Se_1}$	33.3 (29.1,37.4)	34.5 (30.0,38.9)	45.1 (40.7,49.5)
$\boldsymbol{Se_2}$	33.9 (29.7,38.0)	36.0 (31.6,40.5)	47.8 (43.4,52.2)
$\boldsymbol{Se_3}$	35.9 (31.7,40.1)	40.3 (35.8,44.9)	49.0 (44.6,53.4)
$\boldsymbol{Se_4}$	34.7 (30.5,38.9)	36.5 (32.0,41.0)	47.0 (42.5,51.4)
$\boldsymbol{Se_5}$	94.0 (91.9,96.0)	94.4 (92.2,96.5)	94.5 (92.5,96.5)
$\boldsymbol{Sp_1}$	96.6 (95.0,98.2)	95.7 (93.8,97.6)	98.4 (97.3,99.5)
$\boldsymbol{Sp_2}$	98.0 (96.7,99.2)	98.0 (96.7,99.3)	98.0 (96.7,99.2)
$\boldsymbol{Sp_3}$	97.2 (95.7,98.6)	97.1 (95.5,98.6)	98.2 (97.0,99.4)
$\boldsymbol{Sp_4}$	97.6 (96.2,98.9)	96.8 (95.2,98.5)	98.2 (97.0,99.4)
$\boldsymbol{Sp_5}$	1.8 (0.6,3.0)	3.4 (1.7,5.1)	3.5 (1.8,5.1)

Table D. 47 Mean absolute biases of posterior median estimates for prevalence, sensitivities, and specificities with mean residual deviances, DIC values, CrI widths providing minimum distances, corresponding correct inclusion rates (CIR) and false inclusion rates (FIR) of the interaction terms, as well as 95% CrI distances, CIRs and FIRs across converged L-L models with all interaction terms within both diseased and disease-free states with three different priors (Hyplerlasso, Elastic Net, Regularized Horseshoe) fitted to the 500 data sets simulated from L-L DGM in CDPN data setting with the sample sizes of 2000. Estimates are given with 2.5th and 97.5th percentiles.

	**Sample size = 2000**		
**Parameter**	**L-L with HL priors**	**L-L with EN priors**	**L-L with RH priors**
$\boldsymbol{\pi}$	-0.044 (-0.088,0.004)	-0.045 (-0.088,0.005)	-0.043 (-0.088,0.007)
$\boldsymbol{Se_1}$	0.072 (0.015,0.128)	0.071 (0.011,0.125)	0.068 (0.009,0.123)
$\boldsymbol{Se_2}$	0.073 (0.013,0.125)	0.072 (0.013,0.125)	0.069 (0.010,0.121)
$\boldsymbol{Se_3}$	0.072 (0.011,0.124)	0.071 (0.011,0.122)	0.068 (0.014,0.119)
$\boldsymbol{Se_4}$	0.072 (0.018,0.129)	0.071 (0.016,0.127)	0.068 (0.011,0.125)
$\boldsymbol{Se_5}$	-0.036 (-0.077,0.005)	-0.036 (-0.083,0.007)	-0.039 (-0.081,0.005)
$\boldsymbol{Sp_1}$	0.015 (-0.015,0.039)	0.013 (-0.016,0.040)	0.013 (-0.016,0.040)
$\boldsymbol{Sp_2}$	0.014 (-0.016,0.038)	0.013 (-0.016,0.039)	0.013 (-0.016,0.038)
$\boldsymbol{Sp_3}$	0.015 (-0.014,0.041)	0.013 (-0.017,0.039)	0.013 (-0.016,0.041)
$\boldsymbol{Sp_4}$	0.014 (-0.017,0.039)	0.012 (-0.019,0.041)	0.013 (-0.018,0.040)
$\boldsymbol{Sp_5}$	-0.080 (-0.113,-0.046)	-0.081 (-0.117,-0.045)	-0.080 (-0.114,-0.044)
**ResD**	33.59 (28.73,40.20)	32.22 (27.70,39.03)	31.43 (26.73,38.53)
**DIC**	64.86 (56.91,74.52)	61.35 (54.64,70.99)	58.53 (51.85,69.37)
**Minimum Distance**	0.537 (60% CrI)	0.539 (60% CrI)	0.531 (60% CrI)
**CIR**	0.492	0.523	0.526
**FIR**	0.173	0.250	0.239
**95% CrI Distance**	0.870	0.853	0.868
**95% CrI CIR**	0.130	0.147	0.132
**95% CrI FIR**	0.006	0.013	0.011

Table D. 48 95% CrI coverages for prevalence, sensitivities, and specificities across converged L-L models with all interaction terms within both diseased and disease-free states with three different priors (Hyplerlasso, Elastic Net, Regularized Horseshoe) fitted to the 500 data sets simulated from L-L DGM in CDPN data setting with the sample sizes of 2000. Coverages are presented as percentages along with 95% Monte Carlo confidence intervals.

	**Sample size = 2000**		
**Parameter**	**L-L with HL priors**	**L-L with EN priors**	**L-L with RH priors**
$\boldsymbol{\pi}$	84.3 (80.9,87.7)	86.8 (83.6,90.1)	91.1 (88.5,93.8)
$\boldsymbol{Se_1}$	61.4 (56.9,66.0)	66.7 (62.1,71.2)	73.1 (69.0,77.3)
$\boldsymbol{Se_2}$	59.2 (54.6,63.8)	67.1 (62.6,71.7)	72.2 (68.0,76.4)
$\boldsymbol{Se_3}$	62.3 (57.8,66.8)	69.3 (64.9,73.7)	74.3 (70.2,78.3)
$\boldsymbol{Se_4}$	59.2 (54.6,63.8)	65.7 (61.2,70.3)	72.4 (68.3,76.6)
$\boldsymbol{Se_5}$	72.9 (68.7,77.0)	79.1 (75.2,83.0)	78.4 (74.5,82.2)
$\boldsymbol{Sp_1}$	98.2 (97.0,99.4)	99.3 (98.5,100.0)	99.3 (98.5,100.0)
$\boldsymbol{Sp_2}$	98.2 (97.0,99.4)	98.6 (97.4,99.7)	99.5 (98.9,100.0)
$\boldsymbol{Sp_3}$	98.9 (97.9,99.9)	99.3 (98.5,100.0)	99.8 (99.3,100.0)
$\boldsymbol{Sp_4}$	98.2 (97.0,99.4)	97.8 (96.4,99.2)	99.5 (98.9,100.0)
$\boldsymbol{Sp_5}$	14.6 (11.3,17.8)	14.6 (11.2,18.0)	18.2 (14.6,21.8)

Table D. 49 Mean absolute biases of posterior median estimates for prevalence, sensitivities, and specificities with mean residual deviances, DIC values, CrI widths providing minimum distances, corresponding correct inclusion rates (CIR) and false inclusion rates (FIR) of the interaction terms, as well as 95% CrI distances, CIRs and FIRs across converged L-L models with all interaction terms within both diseased and disease-free states with three different priors (Hyplerlasso, Elastic Net, Regularized Horseshoe) fitted to the 500 data sets simulated from L-L DGM in CDPN data setting with the sample sizes of 5000. Estimates are given with 2.5th and 97.5th percentiles.

	**Sample size = 5000**		
**Parameter**	**L-L with HL priors**	**L-L with EN priors**	**L-L with RH priors**
π	-0.004 (-0.052,0.042)	-0.014 (-0.073,0.039)	-0.008 (-0.052,0.037)
Se_1	0.028 (-0.018,0.076)	0.036 (-0.010,0.084)	0.030 (-0.018,0.074)
Se_2	0.028 (-0.022,0.079)	0.036 (-0.011,0.085)	0.030 (-0.018,0.075)
Se_3	0.028 (-0.016,0.074)	0.035 (-0.009,0.087)	0.030 (-0.018,0.079)
Se_4	0.028 (-0.013,0.075)	0.035 (-0.013,0.099)	0.030 (-0.014,0.079)
Se_5	-0.046 (-0.079,-0.019)	-0.047 (-0.083,-0.017)	-0.048 (-0.080,-0.021)
Sp_1	0.022 (-0.002,0.044)	0.019 (-0.017,0.044)	0.021 (-0.004,0.042)
Sp_2	0.022 (-0.008,0.042)	0.019 (-0.012,0.041)	0.020 (-0.011,0.043)
Sp_3	0.022 (-0.002,0.042)	0.018 (-0.012,0.041)	0.020 (-0.003,0.041)
Sp_4	0.023 (-0.010,0.043)	0.020 (-0.014,0.042)	0.021 (-0.010,0.045)
Sp_5	-0.049 (-0.089,-0.016)	-0.059 (-0.104,-0.021)	-0.054 (-0.094,-0.020)
**ResD**	31.76 (27.75,37.86)	31.01 (27.17,36.83)	30.49 (26.84,35.35)
**DIC**	59.89 (53.23,68.20)	58.30 (52.87,67.88)	57.01 (51.88,64.79)
**Minimum Distance**	0.489 (60% CrI)	0.494 (70% CrI)	0.489 (70% CrI)
**CIR**	0.570	0.556	0.554
**FIR**	0.232	0.215	0.202
**95% CrI Distance**	0.630	0.680	0.684
**95% CrI CIR**	0.370	0.321	0.316
**95% CrI FIR**	0.018	0.028	0.027

Table D. 50 95% CrI coverages for prevalence, sensitivities, and specificities across converged L-L models with all interaction terms within both diseased and disease-free states with three different priors (Hyplerlasso, Elastic Net, Regularized Horseshoe) fitted to the 500 data sets simulated from L-L DGM in CDPN data setting with the sample sizes of 5000. Coverages are presented as percentages along with 95% Monte Carlo confidence intervals.

	**Sample size = 5000**		
**Parameter**	**L-L with HL priors**	**L-L with EN priors**	**L-L with RH priors**
$\boldsymbol{\pi}$	99.7 (99.0,100.0)	99.5 (98.6,100.0)	99.6 (98.9,100.0)
$\boldsymbol{Se_1}$	95.9 (93.7,98.2)	94.9 (92.0,97.8)	97.7 (95.9,99.5)
$\boldsymbol{Se_2}$	94.3 (91.6,96.9)	92.6 (89.1,96.1)	95.5 (92.9,98.0)
$\boldsymbol{Se_3}$	96.6 (94.6,98.7)	94.9 (92.0,97.8)	95.8 (93.4,98.2)
$\boldsymbol{Se_4}$	94.9 (92.4,97.4)	94.0 (90.9,97.2)	97.3 (95.4,99.3)
$\boldsymbol{Se_5}$	38.2 (32.6,43.7)	56.7 (50.1,63.3)	51.1 (45.1,57.2)
$\boldsymbol{Sp_1}$	96.3 (94.1,98.4)	98.6 (97.1,100.0)	98.9 (97.6,100.0)
$\boldsymbol{Sp_2}$	98.0 (96.4,99.6)	98.6 (97.1,100.0)	98.5 (97.0,100.0)
$\boldsymbol{Sp_3}$	97.3 (95.4,99.1)	99.5 (98.6,100.0)	99.6 (98.9,100.0)
$\boldsymbol{Sp_4}$	98.3 (96.8,99.8)	100.0 (100.0,100.0)	99.6 (98.9,100.0)
$\boldsymbol{Sp_5}$	60.5 (54.9,66.0)	47.0 (40.4,53.6)	57.2 (51.2,63.2)

Appendix E

Coverage

Table E. 1 and Table E. 2 show 95% CrI coverage of parameter estimates obtained from the CInd and L-L models fitted to datasets simulated from different DGMs, with a sample size of 2,000. L-L models with pairwise interactions generally achieved 95% CrI coverage rates closer to the nominal level than those from the CInd model, regardless of whether vague or shrinkage priors were used.

For data simulated based on the HIV data set, coverage exceeded 90% and was often close to 95% across all combinations of DGM and fitted model, for all parameters (Table E. 1 and Table E. 2). For data simulated based on the VL data set, coverage of 95% CrIs from CInd models was very low for prevalence, sensitivities of Tests 3 and 4, and specificities of Tests 1 and 2 across DGMs, particularly for the latent trait DGM, where coverage was 0.0% for several parameters (Table E. 1). Similarly, when data were simulated based on the CPTB or CDPN data sets, coverage from the CInd model for the prevalence and several accuracy parameters was either 0.0% or very low (Table E. 1).

For the VL data, coverage of 95% CrIs from L-L models with 'correct' pairwise interactions was close to 95% (range 93.4% to 95.6%) when the DGM was L-L or fixed-effect, but somewhat lower when the DGM was latent trait (although these increased to above 90% for all parameters when the sample size was increased to 5,000; Table D.16 in Appendix D). When the DGM was L-L and all interactions within the diseased state were fitted using regularised horseshoe priors, coverage increased to between 94.6% and 98.4% (Table E. 1).

Across simulations based on the CPTB data set, L-L models with correctly specified interactions produced coverage ranging from 69.9% to 96.9% for the latent trait DGM, 49.3% to 94.8% for the fixed-effect DGM, and 76.9% to 96.4% for the L-L DGM. Coverage was lowest for prevalence and the sensitivities of Tests 1 and 2 across all DGMs (Table E. 2). Increasing the sample size did not necessarily

lead to an increase in the coverages for all parameters (Table D.30 in Appendix D). L-L models with correct interactions and those including all interactions with regularised horseshoe priors showed similar coverage for each parameter (Table E. 2). Additionally, increasing the sample size to 5,000 raised the minimum coverage provided by the true L-L model to 88.8% when the DGM was also L-L (Table D.32 in Appendix D).

When the data was simulated based on the CDPN data set, coverage of 95% CrIs from L-L models was greater than 90% for all parameters when the DGM was latent trait or fixed-effect (Table E. 2). When the DGM was L-L, fitting the correct L-L model produced slightly lower coverages for the sensitivities of all tests and the specificity of the independent Test 5. This coverage was only increased by around 4% when the sample size was 5,000 (Table D.44 in Appendix D). Additionally, the use of regularised horseshoe priors decreased coverages for these parameters (Table E. 2).

Table E. 1 95% CrI coverages for prevalence, sensitivities, and specificities across converged CInd and L-L models with correct pairwise interactions fitted to the 1,250 data sets simulated from each DGM and L-L models with regularized horseshoe priors fitted to the 500 data sets simulated from the L-L DGMs in HIV and VL data settings (sample size=2,000). Coverages are presented as percentages.

	HIV Data							VL Data						
	Latent Trait		Fixed-Effect		L-L			Latent Trait		Fixed-Effect		L-L		
**Parameter**	**CInd**	**L-L**	**CInd**	**L-L**	**CInd**	**L-L**	**RH***	**CInd**	**L-L**	**CInd**	**L-L**	**CInd**	**L-L**	**RH***
π	94.6	94.8	94.7	94.6	96.2	95.8	95.4	0.0	83.6	8.8	95.4	8.7	94.6	97.2
Se_1	95.4	95.5	95.4	95.8	96.4	96.4	96.0	4.4	84.4	94.1	94.1	94.9	95.5	96.6
Se_2	95.2	95.2	95.1	94.9	95.5	95.6	94.8	2.8	85.5	90.0	94.3	90.3	95.6	98.2
Se_3	94.4	94.5	94.7	95.0	94.0	94.4	93.0	0.0	81.0	2.0	95.4	2.3	93.8	97.2
Se_4	95.8	94.2	94.4	94.2	95.8	96.2	94.0	0.0	81.3	1.5	95.3	1.8	93.4	98.4
Sp_1	90.8	94.7	91.6	94.6	92.8	95.1	95.6	0.0	80.3	0.2	95.0	0.0	94.0	98.4
Sp_2	94.2	94.2	93.7	93.8	95.4	94.9	95.0	0.0	83.2	10.3	94.9	11.8	94.5	97.4
Sp_3	96.1	95.9	95.8	95.1	95.4	95.4	96.4	92.9	92.9	92.5	95.0	92.0	94.2	94.6
Sp_4	93.4	95.5	93.4	95.4	93.2	94.6	94.2	87.9	94.5	93.4	95.0	93.4	94.9	96.0

* *L-L model with regularized horseshoe (RH) priors*

Table E. 2 95% CrI coverages for prevalence, sensitivities, and specificities across converged CInd and L-L models with correct pairwise interactions fitted to the 1,250 data sets simulated from each DGM and L-L models with regularized horseshoe priors fitted to the 500 data sets simulated from the L-L DGMs in CPTB and CDPN data settings (sample size=2,000). Coverages are presented as percentages.

	CPTB Data							CDPN Data						
	Latent Trait		Fixed-Effect		L-L			Latent Trait		Fixed-Effect		L-L		
**Parameter**	**CInd**	**L-L**	**CInd**	**L-L**	**CInd**	**L-L**	**RH***	**CInd**	**L-L**	**CInd**	**L-L**	**CInd**	**L-L**	**RH***
$\boldsymbol{\pi}$	0.0	70.4	0.0	49.3	0.0	79.4	81.0	0.2	96.2	0.0	96.4	0.0	87.3	91.1
$\boldsymbol{Se_1}$	0.0	73.9	0.0	50.4	0.0	77.8	80.0	0.0	92.3	0.0	95.8	0.0	85.9	73.1
$\boldsymbol{Se_2}$	0.0	69.9	0.0	60.5	0.0	76.9	80.6	0.0	93.5	0.0	94.6	0.0	85.0	72.2
$\boldsymbol{Se_3}$	0.0	74.7	10.7	70.9	14.6	86.3	87.2	0.0	91.2	0.0	97.0	0.0	85.5	74.3
$\boldsymbol{Se_4}$	86.7	90.2	77.8	92.3	12.6	84.6	85.8	0.0	93.1	0.0	96.0	0.0	86.4	72.4
$\boldsymbol{Se_5}$	86.9	92.4	92.8	94.8	94.8	94.9	96.8	47.1	99.5	86.2	95.4	86.0	94.2	78.4
$\boldsymbol{Sp_1}$	2.6	80.9	86.3	89.7	93.5	96.4	98.2	38.6	99.6	79.0	93.9	90.1	94.6	99.3
$\boldsymbol{Sp_2}$	51.4	94.3	80.2	69.6	94.4	96.3	95.6	37.2	99.3	81.7	95.0	89.4	95.4	99.5
$\boldsymbol{Sp_3}$	97.5	96.9	80.2	85.2	71.7	82.5	82.0	39.8	99.4	79.1	94.8	90.8	94.9	99.8
$\boldsymbol{Sp_4}$	0.3	83.6	22.6	86.1	6.1	83.6	85.0	37.8	99.4	80.9	94.1	89.5	94.2	99.5
$\boldsymbol{Sp_5}$	0.0	75.6	36.3	79.7	36.8	88.8	89.0	0.0	96.0	0.0	100.0	0.0	87.0	18.2

* *L-L model with regularized horseshoe (RH) priors*

Appendix F

1.1.1. Simulations based on the HIV data set

The fitted CInd models and L-L models incorporating relevant pairwise interactions yielded unbiased estimates for the HIV data set, regardless of the DGM and whether CDep between Tests 2 and 3 was present in one (latent trait and L-L DGMs) or both (fixed-effect DGM) disease states (Figure F. 1). For the L-L DGM, the L-L models incorporating all pairwise interactions with regularised horseshoe priors also produced unbiased estimates. Additionally, the width of 95% empirical intervals for bias was similar across models fitted for each DGM (Figure F. 1).

Figure F. 1 Absolute biases: means and 2.5th and 97.5th percentiles across models fitted to 1,250 (CInd and L-L with correct pairwise interactions) or 500 (L-L with regularised horseshoe (RH) priors) data sets simulated based on the HIV data set. Sample size = 2000.

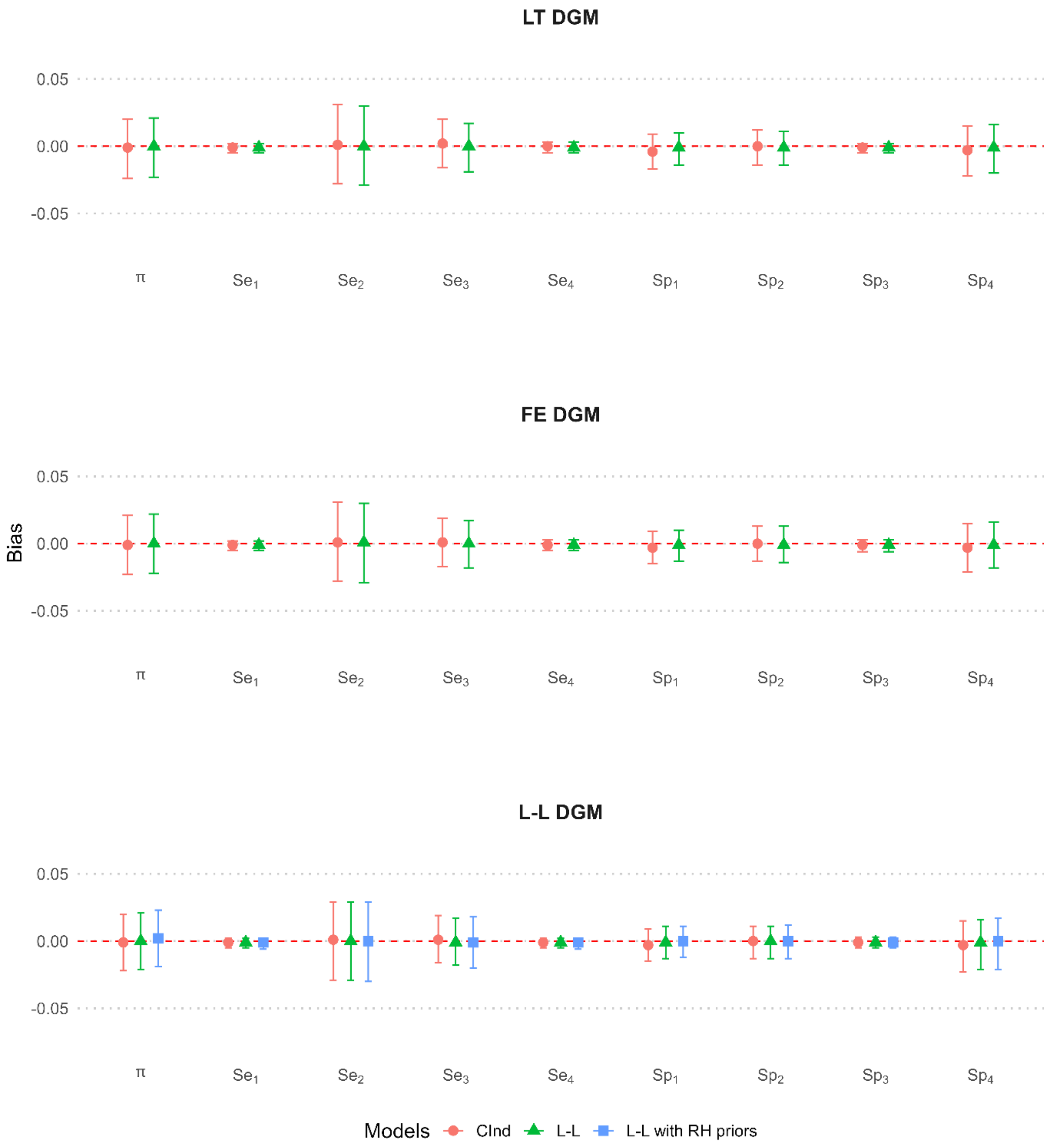

Appendix G

Table G. 1 Absolute biases: means, along with 2.5th and 97.5th percentiles, across models fitted to 1,250 (CInd and L-L with correct pairwise interactions) or 500 (L-L with regularised horseshoe (RH) priors) data sets simulated from L-L DGMs based on four different data sets with different sample sizes (n). Shading: Blue shading denotes cells whose 95% empirical intervals are entirely positive (indicating overestimation), while orange shading denotes cells whose 95% empirical intervals are entirely negative (indicating underestimation).

		HIV Data			VL Data			CPTB Data			CDPN Data		
n	Parameter	CInd	L-L	RH*	CInd	L-L	RH*	CInd	L-L	RH*	CInd	L-L	RH*
500	π	-0.1 (-4.4,4.5)	0.0 (-4.3,4.6)	0.2 (-4.5,4.8)	-4.6 (-9.4,0.7)	-0.6 (-5.0,4.0)	-0.3 (-4.9,4.6)	-6.1 (-9.9,-2.1)	-4.5 (-8.8,0.7)	-4.7 (-8.8,0.2)	-8.8 (-14.2,-3.1)	-4.8 (-12.0,2.4)	-7.2 (-13.4,-0.5)
	Se_1	-0.3 (-1.1,0.1)	-0.3 (-1.1,0.1)	-0.1 (-1.1,0.3)	0.1 (-5.6,5.5)	-0.2 (-6.1,5.3)	-0.6 (-5.9,4.9)	19.8 (11.6,27.6)	16.1 (-0.7,27.0)	17.0 (0.8,27.1)	12.4 (5.5,19.4)	6.0 (-1.9,14.1)	10.5 (3.7,18.1)
	Se_2	0.2 (-5.7,5.8)	0.2 (-5.6,5.8)	0.3 (-5.4,6.0)	0.9 (-5.6,7.4)	0.3 (-6.3,6.7)	0.6 (-5.4,6.9)	20.3 (8.1,33.3)	14.1 (0.1,26.7)	14.9 (0.6,26.7)	12.5 (5.4,19.6)	6.1 (-2.2,15.1)	10.3 (3.1,18.0)
	Se_3	0.0 (-3.4,3.2)	-0.2 (-3.5,3.1)	-0.1 (-3.5,3.1)	10.6 (-0.4,19.9)	1.7 (-5.8,8.9)	1.2 (-5.8,8.9)	9.6 (-1.2,23.8)	6.2 (-3.7,7.4)	6.5 (-4.0,17.2)	12.3 (5.4,19.5)	5.9 (-1.8,14.6)	10.2 (2.9,17.3)
	Se_4	-0.3 (-1.1,0.1)	-0.3 (-1.1,0.1)	-0.1 (-1.1,0.3)	10.8 (-0.3,20.1)	1.6 (-6.0,9.0)	1.2 (-6.0,8.4)	-8.5 (-20.3,1.9)	-5.9 (-16.1,3.9)	-5.7 (-14.9,3.3)	12.3 (5.3,19.0)	5.9 (-2.1,13.9)	10.2 (3.1,17.9)
	Se_5	-	-	-	-	-	-	1.1 (-9.6,11.3)	0.7 (-9.9,10.6)	1.3 (-9.3,11.7)	-0.15 (-8.4,5.5)	0.0 (-8.0,8.6)	-2.4 (-9.6,4.2)
	Sp_1	-0.4 (-3.1,1.8)	-0.2 (-2.7,1.9)	-0.2 (-2.7,1.8)	-5.7 (-10.4,-1.1)	-1.0 (-3.7,1.0)	-1.1 (-3.9,0.7)	-1.1 (-3.3,0.1)	-0.5 (-1.7,0.0)	-0.6 (-1.8,0.0)	0.4 (-4.2,4.4)	-0.2 (-5.9,5.0)	0.5 (-4.8,5.0)
	Sp_2	-0.3 (-2.8,2.1)	-0.3 (-2.9,2.1)	-0.3 (-2.8,2.1)	-4.4 (-9.7,0.6)	-0.6 (-4.3,2.7)	-0.3 (-3.8,3.2)	-0.1 (-1.4,0.8)	-0.2 (-1.5,0.9)	-0.2 (-1.5,0.8)	0.5 (-3.7,4.7)	-0.1 (-5.3,4.8)	0.5 (-3.8,4.6)
	Sp_3	-0.3 (-1.2,0.1)	-0.3 (-1.2,0.1)	-0.3 (-1.2,0.1)	0.0 (-1.7,1.4)	-0.1 (-1.8,1.3)	-0.1 (-1.9,1.3)	-0.4 (-0.9,-0.1)	-0.4 (-0.9,-0.1)	-0.4 (-0.9,-0.1)	0.4 (-3.7,4.3)	-0.2 (-5.7,4.8)	0.3 (-4.3,4.6)
	Sp_4	-0.5 (-4.6,3.1)	-0.3 (-4.2,3.1)	-0.4 (-4.4,3.1)	-0.1 (-1.7,1.1)	-0.2 (-1.7,1.1)	-0.2 (-1.8,1.0)	-4.7 (-9.8,0.0)	-3.4 (-8.4,2.0)	-3.5 (-8.3,1.8)	0.4 (-3.6,4.5)	-0.2 (-5.2,4.7)	0.4 (-4.0,4.6)
	Sp_5	-	-	-	-	-	-	-2.7 (-7.2,1.8)	-2.0 (-6.5,2.8)	-2.1 (-6.6,3.1)	-10.7 (-15.2,-6.3)	-5.6 (-10.9,-1.5)	-10.1 (-16.1,-5.3)
2000	π	-0.1 (-2.2,2.0)	0.0 (-2.1,2.1)	0.2 (-1.9,2.3)	-4.6 (-7.6,-1.6)	-0.2 (-2.5,2.3)	-0.1 (-2.5,2.6)	-5.4 (-7.3,-3.4)	-2.1 (-5.4,2.0)	-2.3 (-5.6,1.5)	-8.6 (-11.4,-5.7)	-2.2 (-6.2,1.7)	-4.3 (-8.8,0.7)
	Se_1	-0.1 (-0.5,0.2)	-0.1 (-0.5,0.2)	-0.1 (-0.6,0.2)	0.3 (-2.5,3.0)	0.0 (-3.0,2.8)	-0.2 (-4.0,2.8)	19.5 (14.5,24.3)	7.2 (-5.4,18.8)	7.8 (-4.2,20.1)	12.4 (8.9,15.9)	2.8 (-1.4,7.6)	6.8 (0.9,12.3)
	Se_2	0.1 (-2.9,2.9)	0.0 (-2.9,2.9)	0.0 (-3.0,2.9)	1.0 (-2.3,4.3)	0.1 (-3.2,3.3)	0.1 (-3.2,3.5)	18.0 (12.1,24.5)	6.6 (-4.4,16.8)	6.9 (-3.9,17.4)	12.4 (8.8,15.9)	2.9 (-1.6,7.6)	6.9 (1.0,12.1)

	Se_3	0.1 (-1.6,1.9)	-0.1 (-1.8,1.7)	-0.1 (-2.0,1.8)	10.6 (4.1,16.4)	0.5 (-3.3,4.3)	0.5 (-3.6,4.5)	8.1 (2.8,14.1)	2.7 (-3.5,9.4)	2.8 (-3.3,9.2)	12.3 (8.9,15.7)	2.8 (-1.3,7.5)	6.8 (1.4,11.9)
	Se_4	-0.1 (-0.5,0.2)	-0.1 (-0.5,0.2)	-0.1 (-0.6,0.2)	10.9 (4.5,16.8)	0.5 (-3.2,4.5)	0.4 (-3.3,4.4)	-7.7 (-13.2,-2.5)	-2.6 (-8.1,2.9)	-2.6 (-8.5,2.8)	12.4 (9.0,15.9)	2.8 (-1.3,7.4)	6.8 (1.1,12.5)
	Se_5	-	-		-	-	-	0.7 (-4.8,6.0)	0.5 (-4.8,5.5)	1.0 (-4.8,5.8)	-1.7 (-5.0,2.1)	-0.3 (-4.4,4.3)	-3.9 (-8.1,0.5)
	Sp_1	-0.3 (-1.5,0.9)	-0.1 (-1.3,1.1)	0.0 (-1.2,1.1)	-5.5 (-8.4,-2.6)	-0.3 (-1.7,1.0)	-0.4 (-1.9,0.9)	-0.4 (-1.4,0.1)	-0.1 (-0.7,0.2)	-0.1 (-0.6,0.1)	0.6 (-1.5,2.6)	0.2 (-2.9,2.8)	1.3 (-1.6,4.0)
	Sp_2	0.0 (-1.3,1.1)	0.0 (-1.3,1.1)	0.0 (-1.3,1.2)	-4.2 (-7.4,-1.0)	-0.2 (-1.8,1.6)	-0.1 (-1.9,1.8)	0.1 (-0.7,0.8)	0.0 (-0.7,0.7)	0.0 (-0.8,0.7)	0.7 (-1.5,2.6)	0.2 (-2.4,2.9)	1.3 (-1.6,3.8)
	Sp_3	-0.1 (-0.5,0.3)	-0.1 (-0.5,0.3)	-0.1 (-0.5,0.3)	0.2 (-0.7,0.9)	0.0 (-0.9,0.8)	0.0 (-0.9,0.8)	-0.2 (-0.4,0.0)	-0.1 (-0.4,0.0)	-0.2 (-0.4,0.0)	0.6 (-1.6,2.6)	0.2 (-2.7,2.8)	1.3 (-1.6,4.1)
	Sp_4	-0.3 (-2.3,1.5)	-0.1 (-2.1,1.6)	0.0 (-2.1,1.7)	0.1 (-0.6,0.8)	0.0 (-0.8,0.7)	0.0 (-0.8,0.7)	-4.3 (-6.7,-2.0)	-1.6 (-4.2,2.0)	-1.7 (-4.9,1.9)	0.6 (-1.4,2.7)	0.2 (-3.0,2.9)	1.3 (-1.8,4.0)
	Sp_5	-	-	-	-	-	-	-2.5 (-4.7,-0.4)	-0.9 (-3.5,2.0)	-0.8 (-3.5,2.1)	-10.6 (-12.8,-8.2)	-2.9 (-6.4,-0.5)	-8.0 (-11.4,-4.4)
	π	-0.1 (-1.3,1.3)	0.0 (-1.2,1.3)	0.1 (-1.1,1.5)	-4.6 (-6.7,-2.5)	0.0 (-1.4,1.4)	0.1 (-1.6,1.7)	-5.1 (-6.4,-3.9)	-0.8 (-3.2,1.8)	-0.9 (-3.3,1.7)	-8.6 (-10.3,-6.8)	-1.2 (-4.1,1.4)	-0.8 (-5.2,3.7)
	Se_1	0.0 (-0.3,0.2)	0.0 (-0.3,0.2)	-0.1 (-0.4,0.2)	0.2 (-1.6,2.0)	-0.1 (-2.0,1.8)	-0.2 (-2.7,1.8)	19.3 (16.2,22.2)	2.6 (-5.1,10.6)	3.0 (-4.2,11.5)	12.5 (10.2,14.8)	1.7 (-1.3,5.3)	3.0 (-1.8,7.4)
	Se_2	0.1 (-1.8,2.1)	0.0 (-1.8,2.0)	0.0 (-1.9,1.9)	1.0 (-1.2,3.2)	0.0 (-2.2,2.2)	0.1 (-2.4,2.4)	17.1 (13.6,21.0)	2.5 (-4.0,9.2)	2.7 (-3.7,9.7)	12.4 (10.3,14.7)	1.7 (-1.2,5.1)	3.0 (-1.8,7.5)
	Se_3	0.1 (-1.0,1.2)	0.0 (-1.1,1.0)	-0.1 (-1.2,1.0)	10.7 (5.9,15.0)	0.2 (-2.2,2.5)	0.1 (-2.8,2.9)	7.5 (3.9,11.4)	0.8 (-3.0,4.7)	1.0 (-3.0,4.9)	12.5 (10.2,14.7)	1.7 (-1.2,5.2)	3.0 (-1.8,7.9)
	Se_4	0.0 (-0.3,0.2)	0.0 (-0.3,0.2)	-0.1 (-0.4,0.2)	11.0 (6.1,15.3)	0.2 (-2.3,2.5)	0.1 (-2.8,2.6)	-7.4 (10.7,-4.3)	-0.9 (-4.3,2.5)	-1.0 (-4.4,2.4)	12.4 (10.1,14.7)	1.7 (-1.2,5.2)	3.0 (-1.4,7.9)
**5000**	Se_5	-	-	-	-	-	-	0.3 (-3.0,3.7)	0.3 (-2.7,3.5)	0.7 (-2.6,4.2)	-1.7 (-3.9,0.5)	-0.2 (-2.9,2.4)	-4.8 (-8.0,-2.1)
	Sp_1	-0.2 (-1.0,0.5)	0.0 (-0.8,0.7)	0.0 (-0.7,0.8)	-5.6 (-7.9,-3.3)	-0.1 (-1.0,0.8)	-0.2 (-1.4,0.9)	-0.1 (-0.8,0.2)	-0.1 (-0.4,0.2)	-0.1 (-0.4,0.2)	0.7 (-0.7,2.0)	0.2 (-1.7,1.9)	2.1 (-0.4,4.2)
	Sp_2	0.0 (-0.8,0.8)	0.0 (-0.8,0.7)	0.0 (-0.7,0.8)	-4.3 (-6.6,-1.9)	-0.1 (-1.2,1.0)	0.0 (-1.3,1.3)	0.1 (-0.3,0.6)	0.0 (-0.4,0.4)	0.0 (-0.5,0.4)	0.6 (-0.7,2.0)	0.2 (-1.7,2.0)	2.0 (-1.1,4.3)
	Sp_3	0.0 (-0.3,0.2)	0.0 (-0.3,0.2)	0.0 (-0.3,0.2)	0.2 (-0.3,0.7)	0.0 (-0.5,0.5)	0.0 (-0.5,0.5)	-0.1 (-0.3,0.0)	-0.1 (-0.2,0.0)	-0.1 (-0.2,0.0)	0.7 (-0.6,1.9)	0.1 (-1.8,1.8)	2.0 (-0.3,4.1)
	Sp_4	-0.2 (-1.5,0.9)	0.0 (-1.2,1.1)	0.0 (-1.2,1.2)	0.2 (-0.3,0.6)	0.0 (-0.5,0.4)	0.0 (-0.5,0.4)	-4.2 (-5.8,-2.6)	-0.7 (-3.0,1.7)	-0.8 (-3.2,1.8)	0.7 (-0.7,1.9)	0.2 (-1.7,1.9)	2.1 (-1.0,4.5)
	Sp_5	-	-	-	-	-	-	-2.5 (-3.9,-1.2)	-0.4 (-2.1,1.3)	-0.3 (-2.0,1.3)	-10.6 (-12.0,-9.2)	-1.7 (-4.5,0.0)	-5.4 (-9.4,-2.0)

* *L-L model with regularized horseshoe (RH) priors*

References

1. Griffin, J.E. and P.J. Brown, *Bayesian hyper-lassos with non-convex penalization.* Australian & New Zealand Journal of Statistics, 2011. **53**(4): p. 423-442.
2. Piironen, J. and A. Vehtari, *Sparsity information and regularization in the horseshoe and other shrinkage priors.* 2017.
3. Li, Q. and N. Lin, *The Bayesian elastic net.* 2010.
4. Van Erp, S., D.L. Oberski, and J. Mulder, *Shrinkage priors for Bayesian penalized regression.* Journal of Mathematical Psychology, 2019. **89**: p. 31-50.
5. Alvord, W.G., et al., *A method for predicting individual HIV infection status in the absence of clinical information.* AIDS research and human retroviruses, 1988. **4**(4): p. 295-304.
6. Qu, Y.S., M. Tan, and M.H. Kutner, *Random effects models in latent class analysis for evaluating accuracy of diagnostic tests.* Biometrics, 1996. **52**(3): p. 797-810.
7. Wang, C., X. Lin, and K.P. Nelson, *Bayesian hierarchical latent class models for estimating diagnostic accuracy.* Statistical methods in medical research, 2020. **29**(4): p. 1112-1128.
8. Jones, G., et al., *Identifiability of models for multiple diagnostic testing in the absence of a gold standard.* Biometrics, 2010. **66**(3): p. 855-63.
9. Sepúlveda, R., J. Vicente-Villardón, and M. Galindo, *The Biplot as a diagnostic tool of local dependence in latent class models. A medical application.* Statistics in Medicine, 2008. **27**(11): p. 1855-1869.
10. Boelaert, M., et al., *Diagnostic tests for kala-azar: a multi-centre study of the freeze-dried DAT, rK39 strip test and KAtex in East Africa and the Indian subcontinent.* Transactions of the Royal Society of Tropical Medicine and Hygiene, 2008. **102**(1): p. 32-40.
11. Menten, J., M. Boelaert, and E. Lesaffre, *Bayesian latent class models with conditionally dependent diagnostic tests: A case study.* Statistics in Medicine, 2008. **27**(22): p. 4469-4488.
12. Xu, H. and B.A. Craig, *A probit latent class model with general correlation structures for evaluating accuracy of diagnostic tests.* Biometrics, 2009. **65**(4): p. 1145-1155.
13. TorranceRynard, V.L. and S.D. Walter, *Effects of dependent errors in the assessment of diagnostic test performance.* Statistics in Medicine, 1997. **16**(19): p. 2157-2175.
14. Dendukuri, N. and L. Joseph, *Bayesian approaches to modeling the conditional dependence between multiple diagnostic tests.* Biometrics, 2001. **57**(1): p. 158-167.
15. Nicol, M.P., et al., *Accuracy of the Xpert MTB/RIF test for the diagnosis of pulmonary tuberculosis in children admitted to hospital in Cape Town, South Africa: a descriptive study.* The Lancet infectious diseases, 2011. **11**(11): p. 819-824.
16. Zar, H.J., et al., *Rapid molecular diagnosis of pulmonary tuberculosis in children using nasopharyngeal specimens.* Clinical infectious diseases, 2012. **55**(8): p. 1088-1095.
17. Wang, Z., et al., *Modeling conditional dependence among multiple diagnostic tests.* Statistics in Medicine, 2017. **36**(30): p. 4843-4859.
18. Keddie, S.H., et al., *Estimating sensitivity and specificity of diagnostic tests using latent class models that account for conditional dependence between tests: a simulation study.* BMC Medical Research Methodology, 2023. **23**(1): p. 58.
19. Morris, T.P., I.R. White, and M.J. Crowther, *Using simulation studies to evaluate statistical methods.* Statistics in Medicine, 2019. **38**(11): p. 2074-2102.
