# Supplementary material for: Latent Class Log‐Linear Models for Estimating Diagnostic Test Accuracy Without a Gold Standard: A Simulation Study
Source: Stat Med. 2026 Jul 6;45(15-17):e70660. doi: 10.1002/sim.70660 (PMC13334516; doi:10.1002/sim.70660)
Supplement: Supplementary file 2 — Supporting information: 1. [file SIM-45-0-s002.pdf]

R and JAGS Scripts to Simulate Data and Fit Models for Each Data-Generating Mechanism Based on Four Real-World Data Sets

All R scripts simulating data with sample size of 2,000 (as an example) and JAGS scripts fitting L-L models with pairwise interaction terms are given below. Throughout the analyses, CInd models were fitted within the L-L framework – that is, as L-L models including only main effects. The JAGS script for a CInd model can therefore be obtained by simply removing the interaction terms from the corresponding L-L model script with interactions.

HIV Data

Latent Trait DGM

R script to simulate data and fit L-L models

```
## This R script simulates data from latent trait DGM based on the HIV data
## with the parameter settings specified in Appendix B and fits the
corresponding
## log-linear model with correct interactions.

## Clear workspace
rm(list = ls())

## Load required package
require(R2jags)

## Record start time
start <- Sys.time()

## Create results folder if it does not exist
folder <- "Chapter5-HIV_LT_LL"
if (!file.exists(folder)) dir.create(folder)

## Set seed for reproducibility
set.seed(567)

## Simulation settings
nobs <- 2000          # number of individuals per dataset
ntests <- 4           # number of diagnostic tests
nsim <- 1250          # number of simulated datasets
n.burnin <- 10000     # burn-in iterations
n.iter <- 50000       # total MCMC iterations
n.thin <- 1           # thinning interval
n.chains <- 3         # number of MCMC chains

## True values for data generation
```

```
prev <- 0.542
se <- c(0.997, 0.569, 0.908, 0.996)     # test sensitivities
sp <- c(0.970, 0.962, 0.997, 0.918)     # test specificities
b.RE1 <- c(0, 1.050, 1.050, 0)          # sensitivity random effect loading
b.RE2 <- c(0, 0, 0, 0)                  # specificity random effect loading
(fixed here)

## Compute latent thresholds for sensitivity and specificity
qnorm_adj <- function(p, b) qnorm(p) * sqrt(1 + b^2)
a1 <- mapply(qnorm_adj, se, b.RE1)
a2 <- mapply(qnorm_adj, sp, b.RE2)

## Initialise data storage
r <- d <- matrix(NA, nsim, nobs)
s <- c <- p <- y <- array(NA, dim = c(nsim, nobs, ntests))

## Generate latent data for each dataset
for (k in 1:nsim) {
  r[k, ] <- rnorm(nobs)
  d[k, ] <- rbinom(nobs, 1, prev)
  for (j in 1:ntests) {
    s[k, , j] <- pnorm(a1[j] + b.RE1[j] * r[k, ])
    c[k, , j] <- pnorm(a2[j] + b.RE2[j] * r[k, ])
    p[k, , j] <- (s[k, , j]^d[k, ]) * ((1 - c[k, , j])^(1 - d[k, ]))
    y[k, , j] <- rbinom(nobs, 1, p[k, , j])
  }
}

## Function to tabulate test patterns
freqtable <- function(data) {
  freq <- rep(0, 16)
  pats <- apply(data, 1, function(x) paste0(x, collapse = ""))
  tpats <- table(pats)
  names_vec <- apply(expand.grid(rep(list(c(0, 1)), 4)), 1, paste0,
collapse = "")
  freq[match(names(tpats), names_vec)] <- tpats
  cbind(expand.grid(c(0, 1), c(0, 1), c(0, 1), c(0, 1)), freq)
}

## Convert test results into 4-test pattern frequencies
Y <- array(NA, dim = c(nsim, 16, ntests + 1))
for (i in 1:nsim) {
  Y[i, , ] <- as.matrix(freqtable(y[i, , ]))
}

## Parameters to monitor in JAGS
mymonitoredparamslist <- c("prev", "sens", "spec", "sumrd")

## Prepare storage for posterior summaries
prevmedian <- prevlower <- prevupper <- rep(NA, nsim)
semedian <- selower <- seupper <- matrix(NA, nrow = ntests, ncol = nsim)
spmedian <- splower <- spupper <- matrix(NA, nrow = ntests, ncol = nsim)
ResD <- DIC <- rep(NA, nsim)

## Fit JAGS model to each simulated dataset
for (j in 1:nsim) {
  mydatalist <- list(
    r = Y[j, , ntests + 1],
    y = Y[j, , 1:ntests],
    nobs = nobs,
    AcceptTest = rep(1, ntests)
```

```
  )

  fit <- jags(
    data = mydatalist,
    parameters.to.save = mymonitoredparamslist,
    model.file = "hiv_lt_ll.txt",
    n.chains = n.chains,
    n.iter = n.iter,
    n.burnin = n.burnin,
    n.thin = n.thin,
    progress.bar = "text"
  )

  # Convergence check
  summ <- fit$BUGSoutput$summary
  if (any(summ[2:11, 8] > 1.1) || any(summ[2:11, 9] < 400)) {
    next
  }

  prevmedian[j] <- fit$BUGSoutput$median$prev
  prevlower[j] <- summ[2, 3]
  prevupper[j] <- summ[2, 7]
  semedian[, j] <- fit$BUGSoutput$median$sens
  selower[, j] <- summ[3:6, 3]
  seupper[, j] <- summ[3:6, 7]
  spmedian[, j] <- fit$BUGSoutput$median$spec
  splower[, j] <- summ[7:10, 3]
  spupper[, j] <- summ[7:10, 7]
  ResD[j] <- fit$BUGSoutput$mean$sumrd
  DIC[j] <- ResD[j] + fit$BUGSoutput$pD
}

## Clean up
rm(fit)

## Save workspace
save.image(file = file.path(folder, "HIV_LT_LL_2000.RData"))

## Report total run time
print(Sys.time() - start)
```

JAGS script to fit the L-L model with correct interactions

```
model{

  for (i in 1:16){

    r[i] ~ dpois(mu[i])

    mu[i] <- p[i] * nobs   # Expected frequency

    p[i] <- (1 - prev) * p1[i] + prev * p2[i]   # Mixture model

    p1[i] <- y1[i] / sum(y1[])   # Pr(pattern i | no disease)
    p2[i] <- y2[i] / sum(y2[])   # Pr(pattern i | disease)

    # Log-linear regression for disease-free population:
    log(y1[i]) <- lambda_coef[1,1] * y[i,1] + lambda_coef[1,2] * y[i,2] +
                  lambda_coef[1,3] * y[i,3] + lambda_coef[1,4] * y[i,4]
```

```
    # Log-linear regression for diseased population:
    log(y2[i]) <- lambda_coef[2,1] * y[i,1] + lambda_coef[2,2] * y[i,2] +
                  lambda_coef[2,3] * y[i,3] + lambda_coef[2,4] * y[i,4] +
                  lambda_coef[2,5] * y[i,2] * y[i,3]

    # Residual deviance contribution
    rd[i] <- 2 * ((mu[i] - r[i]) + r[i] * log(r[i] / mu[i]))
  }

  sumrd <- sum(rd[])

  # Prior for prevalence
  prev ~ dbeta(1, 1)

  for(t in 1:4){

    # Posterior summaries of test accuracy
    sens[t] <- inprod(y[1:16, t], y2[1:16]) / sum(y2[1:16])    # Sensitivity
    fpr[t]  <- inprod(y[1:16, t], y1[1:16]) / sum(y1[1:16])    # False
positive rate
    spec[t] <- 1 - fpr[t]                                       # Specificity
    youden[t] <- sens[t] + spec[t] - 1                          # Youden
index

    AcceptTest[t] ~ dbern(ifelse(youden[t] >= 0, 1, 0))        # Binary
accept/reject

    # Priors for log-linear coefficients (via inverse logit)
    q[2, t] ~ dbeta(1, 1)
    q[1, t] ~ dbeta(1, 1)

    for(d in 1:2){
      lambda_coef[d, t] <- logit(q[d, t])
    }
  }

  # Prior for interaction term
  lambda_coef[2,5] ~ dnorm(0, 1)
}
```

Fixed-Effect DGM

R script to simulate data and fit L-L models

```
## This R script simulates data from fixed-effect DGM based on the HIV data
## with the parameter settings specified in Appendix B and fits the
corresponding
## log-linear model with correct interactions.

rm(list = ls())                       # Clear environment
require(R2jags)                       # Load JAGS interface

start <- Sys.time()                  # Start timer

# Set up working folder
folder <- "Chapter5-HIV_JJHC_LL"
if (!file.exists(folder)) dir.create(folder)
```

```r
set.seed(567)                        # For reproducibility

# ----------------------
# Simulation Parameters
# ----------------------
nobs     <- 2000                     # Number of observations per dataset
ntests   <- 4                        # Number of tests
nsim     <- 1250                     # Number of simulated datasets
n.burnin <- 10000                    # Burn-in for MCMC
n.iter   <- 50000                    # Total MCMC iterations
n.thin   <- 1                        # Thinning interval
n.chains <- 3                        # Number of MCMC chains

# ----------------------
# True Values for Simulation
# ----------------------
prev <- 0.542                        # True prevalence
se   <- c(0.997, 0.571, 0.908, 0.996) # Test sensitivities
sp   <- c(0.972, 0.961, 0.995, 0.925) # Test specificities

# Pairwise covariance terms for sensitivity and specificity
covse12 <- 0; covse13 <- 0;    covse14 <- 0
covse23 <- 0.032; covse24 <- 0; covse34 <- 0
covsp12 <- 0; covsp13 <- 0;    covsp14 <- 0
covsp23 <- 0.002; covsp24 <- 0; covsp34 <- 0

# ----------------------
# Generate Probabilities for Each Pattern
# ----------------------
p1 <- rep(NA, 2^ntests)  # Probabilities from true positives
p0 <- rep(NA, 2^ntests)  # Probabilities from true negatives

# Compute probability of each of the 16 test patterns
# (0 = negative, 1 = positive) using known Se/Sp and covariances

# Probability of observing Test1- Test2- Test3- Test4- from a true
positive::
p1[1] <- prev * ((1-se[1])*(1-se[2])*(1-se[3])*(1-se[4]) +
                 covse12*(1-se[3])*(1-se[4]) +
                 covse13*(1-se[2])*(1-se[4]) +
                 covse14*(1-se[2])*(1-se[3]) +
                 covse23*(1-se[1])*(1-se[4]) +
                 covse24*(1-se[1])*(1-se[3]) +
                 covse34*(1-se[1])*(1-se[2]))

# Probability of observing Test1- Test2- Test3- Test4- from a true
negative::
p0[1] <- (1-prev) * (sp[1]*sp[2]*sp[3]*sp[4] +
                     covsp12*sp[3]*sp[4] +
                     covsp13*sp[2]*sp[4] +
                     covsp14*sp[2]*sp[3] +
                     covsp23*sp[1]*sp[4] +
                     covsp24*sp[1]*sp[3] +
                     covsp34*sp[1]*sp[2])

# Probability of observing Test1+ Test2- Test3- Test4- from a true
positive::
p1[2] <- prev * (se[1]*(1-se[2])*(1-se[3])*(1-se[4]) -
                 covse12*(1-se[3])*(1-se[4]) -
                 covse13*(1-se[2])*(1-se[4]) -
```

```
                    covse14*(1-se[2])*(1-se[3]) +
                    covse23*se[1]*(1-se[4]) +
                    covse24*se[1]*(1-se[3]) +
                    covse34*se[1]*(1-se[2]))

# Probability of observing Test1+ Test2- Test3- Test4- from a true
negative::
p0[2] <- (1-prev) * ((1-sp[1])*sp[2]*sp[3]*sp[4] -
                    covsp12*sp[3]*sp[4] -
                    covsp13*sp[2]*sp[4] -
                    covsp14*sp[2]*sp[3] +
                    covsp23*(1-sp[1])*sp[4] +
                    covsp24*(1-sp[1])*sp[3] +
                    covsp34*(1-sp[1])*sp[2])


# Probability of observing Test1- Test2+ Test3- Test4- from a true
positive::
p1[3] <- prev * ((1-se[1])*se[2]*(1-se[3])*(1-se[4]) -
                covse12*(1-se[3])*(1-se[4]) +
                covse13*se[2]*(1-se[4]) +
                covse14*se[2]*(1-se[3]) -
                covse23*(1-se[1])*(1-se[4]) -
                covse24*(1-se[1])*(1-se[3]) +
                covse34*(1-se[1])*se[2])

# Probability of observing Test1- Test2+ Test3- Test4- from a true
negative::
p0[3] <- (1-prev) * (sp[1]*(1-sp[2])*sp[3]*sp[4] -
                    covsp12*sp[3]*sp[4] +
                    covsp13*(1-sp[2])*sp[4] +
                    covsp14*(1-sp[2])*sp[3] -
                    covsp23*sp[1]*sp[4] -
                    covsp24*sp[1]*sp[3] +
                    covsp34*sp[1]*(1-sp[2]))


# Probability of observing Test1+ Test2+ Test3- Test4- from a true
positive::
p1[4] <- prev * (se[1]*se[2]*(1-se[3])*(1-se[4]) +
                covse12*(1-se[3])*(1-se[4]) -
                covse13*se[2]*(1-se[4]) -
                covse14*se[2]*(1-se[3]) -
                covse23*se[1]*(1-se[4]) -
                covse24*se[1]*(1-se[3]) +
                covse34*se[1]*se[2])

# Probability of observing Test1+ Test2+ Test3- Test4- from a true
negative::
p0[4] <- (1-prev) * ((1-sp[1])*(1-sp[2])*sp[3]*sp[4] +
                    covsp12*sp[3]*sp[4] -
                    covsp13*(1-sp[2])*sp[4] -
                    covsp14*(1-sp[2])*sp[3] -
                    covsp23*(1-sp[1])*sp[4] -
                    covsp24*(1-sp[1])*sp[3] +
                    covsp34*(1-sp[1])*(1-sp[2]))


# Probability of observing Test1+ Test2+ Test3- Test4- from a true
positive::
p1[4] <- prev * (se[1]*se[2]*(1-se[3])*(1-se[4]) +
```

```
                    covse12*(1-se[3])*(1-se[4]) -
                    covse13*se[2]*(1-se[4]) -
                    covse14*se[2]*(1-se[3]) -
                    covse23*se[1]*(1-se[4]) -
                    covse24*se[1]*(1-se[3]) +
                    covse34*se[1]*se[2])

# Probability of observing Test1+ Test2+ Test3- Test4- from a true
negative::
p0[4] <- (1-prev) * ((1-sp[1])*(1-sp[2])*sp[3]*sp[4] +
                        covsp12*sp[3]*sp[4] -
                        covsp13*(1-sp[2])*sp[4] -
                        covsp14*(1-sp[2])*sp[3] -
                        covsp23*(1-sp[1])*sp[4] -
                        covsp24*(1-sp[1])*sp[3] +
                        covsp34*(1-sp[1])*(1-sp[2]))


# Probability of observing Test1- Test2- Test3+ Test4- from a true
positive::
p1[5] <- prev * ((1-se[1])*(1-se[2])*se[3]*(1-se[4]) +
                    covse12*se[3]*(1-se[4]) -
                    covse13*(1-se[2])*(1-se[4]) +
                    covse14*(1-se[2])*se[3] -
                    covse23*(1-se[1])*(1-se[4]) +
                    covse24*(1-se[1])*se[3] -
                    covse34*(1-se[1])*(1-se[2]))

# Probability of observing Test1- Test2- Test3+ Test4- from a true
negative::
p0[5] <- (1-prev) * (sp[1]*sp[2]*(1-sp[3])*sp[4] +
                        covsp12*(1-sp[3])*sp[4] -
                        covsp13*sp[2]*sp[4] +
                        covsp14*sp[2]*(1-sp[3]) -
                        covsp23*sp[1]*sp[4] +
                        covsp24*sp[1]*(1-sp[3]) -
                        covsp34*sp[1]*sp[2])


# Probability of observing Test1+ Test2- Test3+ Test4- from a true
positive::
p1[6] <- prev * (se[1]*(1-se[2])*se[3]*(1-se[4]) -
                    covse12*se[3]*(1-se[4]) +
                    covse13*(1-se[2])*(1-se[4]) -
                    covse14*(1-se[2])*se[3] -
                    covse23*se[1]*(1-se[4]) +
                    covse24*se[1]*se[3] -
                    covse34*se[1]*(1-se[2]))

# Probability of observing Test1+ Test2- Test3+ Test4- from a true
negative::
p0[6] <- (1-prev) * ((1-sp[1])*sp[2]*(1-sp[3])*sp[4] -
                        covsp12*(1-sp[3])*sp[4] +
                        covsp13*sp[2]*sp[4] -
                        covsp14*sp[2]*(1-sp[3]) -
                        covsp23*(1-sp[1])*sp[4] +
                        covsp24*(1-sp[1])*(1-sp[3]) -
                        covsp34*(1-sp[1])*sp[2])
```

```
# Probability of observing Test1- Test2+ Test3+ Test4- from a true
positive::
p1[7] <- prev * ((1-se[1])*se[2]*se[3]*(1-se[4]) -
                 covse12*se[3]*(1-se[4]) -
                 covse13*se[2]*(1-se[4]) +
                 covse14*se[2]*se[3] +
                 covse23*(1-se[1])*(1-se[4]) -
                 covse24*(1-se[1])*se[3] -
                 covse34*(1-se[1])*se[2])

# Probability of observing Test1- Test2+ Test3+ Test4- from a true
negative::
p0[7] <- (1-prev) * (sp[1]*(1-sp[2])*(1-sp[3])*sp[4] -
                     covsp12*(1-sp[3])*sp[4] -
                     covsp13*(1-sp[2])*sp[4] +
                     covsp14*(1-sp[2])*(1-sp[3]) +
                     covsp23*sp[1]*sp[4] -
                     covsp24*sp[1]*(1-sp[3]) -
                     covsp34*sp[1]*(1-sp[2]))


# Probability of observing Test1+ Test2+ Test3+ Test4- from a true
positive::
p1[8] <- prev * (se[1]*se[2]*se[3]*(1-se[4]) +
                 covse12*se[3]*(1-se[4]) +
                 covse13*se[2]*(1-se[4]) -
                 covse14*se[2]*se[3] +
                 covse23*se[1]*(1-se[4]) -
                 covse24*se[1]*se[3] -
                 covse34*se[1]*se[2])

# Probability of observing Test1+ Test2+ Test3+ Test4- from a true
negative::
p0[8] <- (1-prev) * ((1-sp[1])*(1-sp[2])*(1-sp[3])*sp[4] +
                     covsp12*(1-sp[3])*sp[4] +
                     covsp13*(1-sp[2])*sp[4] -
                     covsp14*(1-sp[2])*(1-sp[3]) +
                     covsp23*(1-sp[1])*sp[4] -
                     covsp24*(1-sp[1])*(1-sp[3]) -
                     covsp34*(1-sp[1])*(1-sp[2]))


# Probability of observing Test1- Test2- Test3- Test4+ from a true
positive::
p1[9] <- prev * ((1-se[1])*(1-se[2])*(1-se[3])*se[4] +
                 covse12*(1-se[3])*se[4] +
                 covse13*(1-se[2])*se[4] -
                 covse14*(1-se[2])*(1-se[3]) +
                 covse23*(1-se[1])*se[4] -
                 covse24*(1-se[1])*(1-se[3]) -
                 covse34*(1-se[1])*(1-se[2]))

# Probability of observing Test1- Test2- Test3- Test4+ from a true
negative::
p0[9] <- (1-prev) * (sp[1]*sp[2]*sp[3]*(1-sp[4]) +
                     covsp12*sp[3]*(1-sp[4]) +
                     covsp13*sp[2]*(1-sp[4]) -
                     covsp14*sp[2]*sp[3] +
                     covsp23*sp[1]*(1-sp[4]) -
                     covsp24*sp[1]*sp[3] -
                     covsp34*sp[1]*sp[2])
```

```
# Probability of observing Test1+ Test2- Test3- Test4+ from a true
positive::
p1[10] <- prev * (se[1]*(1-se[2])*(1-se[3])*se[4] -
                  covse12*(1-se[3])*se[4] -
                  covse13*(1-se[2])*se[4] +
                  covse14*(1-se[2])*(1-se[3]) +
                  covse23*se[1]*se[4] -
                  covse24*se[1]*(1-se[3]) -
                  covse34*se[1]*(1-se[2]))

# Probability of observing Test1+ Test2- Test3- Test4+ from a true
negative::
p0[10] <- (1-prev) * ((1-sp[1])*sp[2]*sp[3]*(1-sp[4]) -
                      covsp12*sp[3]*(1-sp[4]) -
                      covsp13*sp[2]*(1-sp[4]) +
                      covsp14*sp[2]*sp[3] +
                      covsp23*(1-sp[1])*(1-sp[4]) -
                      covsp24*(1-sp[1])*sp[3] -
                      covsp34*(1-sp[1])*sp[2])


# Probability of observing Test1- Test2+ Test3- Test4+ from a true
positive::
p1[11] <- prev * ((1-se[1])*se[2]*(1-se[3])*se[4] -
                  covse12*(1-se[3])*se[4] +
                  covse13*se[2]*se[4] -
                  covse14*se[2]*(1-se[3]) -
                  covse23*(1-se[1])*se[4] +
                  covse24*(1-se[1])*(1-se[3]) -
                  covse34*(1-se[1])*se[2])

# Probability of observing Test1- Test2+ Test3- Test4+ from a true
negative::
p0[11] <- (1-prev) * (sp[1]*(1-sp[2])*sp[3]*(1-sp[4]) -
                      covsp12*sp[3]*(1-sp[4]) +
                      covsp13*(1-sp[2])*(1-sp[4]) -
                      covsp14*(1-sp[2])*sp[3] -
                      covsp23*sp[1]*(1-sp[4]) +
                      covsp24*sp[1]*sp[3] -
                      covsp34*sp[1]*(1-sp[2]))


# Probability of observing Test1+ Test2+ Test3- Test4+ from a true
positive::
p1[12] <- prev * (se[1]*se[2]*(1-se[3])*se[4] +
                  covse12*(1-se[3])*se[4] -
                  covse13*se[2]*se[4] +
                  covse14*se[2]*(1-se[3]) -
                  covse23*se[1]*se[4] +
                  covse24*se[1]*(1-se[3]) -
                  covse34*se[1]*se[2])

# Probability of observing Test1+ Test2+ Test3- Test4+ from a true
negative::
p0[12] <- (1-prev) * ((1-sp[1])*(1-sp[2])*sp[3]*(1-sp[4]) +
                      covsp12*sp[3]*(1-sp[4]) -
                      covsp13*(1-sp[2])*(1-sp[4]) +
                      covsp14*(1-sp[2])*sp[3] -
                      covsp23*(1-sp[1])*(1-sp[4]) +
```

```
                        covsp24*(1-sp[1])*sp[3] -
                        covsp34*(1-sp[1])*(1-sp[2]))


# Probability of observing Test1- Test2- Test3+ Test4+ from a true
positive::
p1[13] <- prev * ((1-se[1])*(1-se[2])*se[3]*se[4] +
                    covse12*se[3]*se[4] -
                    covse13*(1-se[2])*se[4] -
                    covse14*(1-se[2])*se[3] -
                    covse23*(1-se[1])*se[4] -
                    covse24*(1-se[1])*se[3] +
                    covse34*(1-se[1])*(1-se[2]))

# Probability of observing Test1- Test2- Test3+ Test4+ from a true
negative::
p0[13] <- (1-prev) * (sp[1]*sp[2]*(1-sp[3])*(1-sp[4]) +
                        covsp12*(1-sp[3])*(1-sp[4]) -
                        covsp13*sp[2]*(1-sp[4]) -
                        covsp14*sp[2]*(1-sp[3]) -
                        covsp23*sp[1]*(1-sp[4]) -
                        covsp24*sp[1]*(1-sp[3]) +
                        covsp34*sp[1]*sp[2])


# Probability of observing Test1+ Test2- Test3+ Test4+ from a true
positive::
p1[14] <- prev * (se[1]*(1-se[2])*se[3]*se[4] -
                    covse12*se[3]*se[4] +
                    covse13*(1-se[2])*se[4] +
                    covse14*(1-se[2])*se[3] -
                    covse23*se[1]*se[4] -
                    covse24*se[1]*se[3] +
                    covse34*se[1]*(1-se[2]))

# Probability of observing Test1+ Test2- Test3+ Test4+ from a true
negative::
p0[14] <- (1-prev) * ((1-sp[1])*sp[2]*(1-sp[3])*(1-sp[4]) -
                        covsp12*(1-sp[3])*(1-sp[4]) +
                        covsp13*sp[2]*(1-sp[4]) +
                        covsp14*sp[2]*(1-sp[3]) -
                        covsp23*(1-sp[1])*(1-sp[4]) -
                        covsp24*(1-sp[1])*(1-sp[3]) +
                        covsp34*(1-sp[1])*sp[2])


# Probability of observing Test1- Test2+ Test3+ Test4+ from a true
positive::
p1[15] <- prev * ((1-se[1])*se[2]*se[3]*se[4] -
                    covse12*se[3]*se[4] -
                    covse13*se[2]*se[4] -
                    covse14*se[2]*se[3] +
                    covse23*(1-se[1])*se[4] +
                    covse24*(1-se[1])*se[3] +
                    covse34*(1-se[1])*se[2])

# Probability of observing Test1- Test2+ Test3+ Test4+ from a true
negative::
p0[15] <- (1-prev) * (sp[1]*(1-sp[2])*(1-sp[3])*(1-sp[4]) -
                        covsp12*(1-sp[3])*(1-sp[4]) -
                        covsp13*(1-sp[2])*(1-sp[4]) -
```

```
                          covsp14*(1-sp[2])*(1-sp[3]) +
                          covsp23*sp[1]*(1-sp[4]) +
                          covsp24*sp[1]*(1-sp[3]) +
                          covsp34*sp[1]*(1-sp[2]))


# Probability of observing Test1+ Test2+ Test3+ Test4+ from a true
positive::
p1[16] <- prev * (se[1]*se[2]*se[3]*se[4] +
                      covse12*se[3]*se[4] +
                      covse13*se[2]*se[4] +
                      covse14*se[2]*se[3] +
                      covse23*se[1]*se[4] +
                      covse24*se[1]*se[3] +
                      covse34*se[1]*se[2])

# Probability of observing Test1+ Test2+ Test3+ Test4+ from a true
negative::
p0[16] <- (1-prev) * ((1-sp[1])*(1-sp[2])*(1-sp[3])*(1-sp[4]) +
                          covsp12*(1-sp[3])*(1-sp[4]) +
                          covsp13*(1-sp[2])*(1-sp[4]) +
                          covsp14*(1-sp[2])*(1-sp[3]) +
                          covsp23*(1-sp[1])*(1-sp[4]) +
                          covsp24*(1-sp[1])*(1-sp[3]) +
                          covsp34*(1-sp[1])*(1-sp[2]))

# ----------------------
# Simulate Frequencies from Multinomial Distribution
# ----------------------
prob <- p1 + p0
freq <- rmultinom(nsim, nobs, prob)

# Format simulated data into array Y:
# Each row corresponds to a simulated dataset
# Columns: 4 test outcomes + frequency
Y <- array(NA, dim = c(nsim, 2^ntests, ntests + 1))
for (i in 1:nsim) {
  Y[i,,1:ntests]   <- as.matrix(expand.grid(rep(list(c(0,1)), ntests)))
  Y[i,,ntests + 1] <- freq[,i]
}

# ----------------------
# Run JAGS Model on Simulated Datasets
# ----------------------

# Parameters to extract from JAGS output
mymonitoredparamslist <- c("prev", "sens", "spec", "sumrd")

# Preallocate output storage
prevmedian <- rep(NA, nsim)
prevlower  <- rep(NA, nsim)
prevupper  <- rep(NA, nsim)

semedian <- selower <- seupper <- matrix(NA, nrow = ntests, ncol = nsim)
spmedian <- splower <- spupper <- matrix(NA, nrow = ntests, ncol = nsim)

ResD <- rep(NA, nsim)
DIC  <- rep(NA, nsim)

# Loop over simulated datasets and run JAGS
for (j in 1:nsim) {
```

```
  mydatalist <- list(
    r = Y[j,,ntests+1],
    y = Y[j,,1:ntests],
    nobs = nobs,
    AcceptTest = rep(1, ntests)   # All tests used for model fit
  )

  fit <- jags(data = mydatalist,
              parameters.to.save = mymonitoredparamslist,
              model.file = "hiv_jjhc_ll.txt",
              n.chains = n.chains,
              n.iter = n.iter,
              n.burnin = n.burnin,
              n.thin = n.thin,
              progress.bar = "text")

  # Check for convergence (Rhat < 1.1 and effective sample size > 400)
  if (any(fit$BUGSoutput$summary[2:11,8] > 1.1) |
      any(fit$BUGSoutput$summary[2:11,9] < 400)) {

    # If not converged, store NA
    prevmedian[j] <- prevlower[j] <- prevupper[j] <- NA
    semedian[,j]  <- selower[,j]  <- seupper[,j] <- NA
    spmedian[,j]  <- splower[,j]  <- spupper[,j] <- NA
    ResD[j] <- DIC[j] <- NA

  } else {

    # Store median, lower, and upper quantiles
    prevmedian[j] <- fit$BUGSoutput$median$prev
    prevlower[j]  <- fit$BUGSoutput$summary[2, 3]
    prevupper[j]  <- fit$BUGSoutput$summary[2, 7]

    semedian[,j]  <- fit$BUGSoutput$median$sens
    selower[,j]   <- fit$BUGSoutput$summary[3:6, 3]
    seupper[,j]   <- fit$BUGSoutput$summary[3:6, 7]

    spmedian[,j]  <- fit$BUGSoutput$median$spec
    splower[,j]   <- fit$BUGSoutput$summary[7:10, 3]
    spupper[,j]   <- fit$BUGSoutput$summary[7:10, 7]

    ResD[j] <- fit$BUGSoutput$mean$sumrd
    DIC[j]  <- ResD[j] + fit$BUGSoutput$pD
  }
}

# Cleanup
rm(fit)

# ----------------------
# Save Workspace
# ----------------------
save.image(file = file.path(folder, "HIV_JJHC_LL_2000.RData"))

# Print total execution time
print(Sys.time() - start)
```

JAGS script to fit the L-L model with correct interactions

```
model {

  # Loop over 16 possible test outcome patterns
  for (i in 1:16) {

    r[i] ~ dpois(mu[i])       # Poisson likelihood for observed frequency
in cell i
    mu[i] <- p[i] * nobs      # Expected count = Pr(cell i) × total sample
size

    # Mixture model: weighted average of cell probabilities under negative
and positive groups
    p[i] <- (1 - prev) * p1[i] + prev * p2[i]

    # Probability of test pattern i under no disease and disease
(normalized)
    p1[i] <- y1[i] / sum(y1[])    # Pr(pattern i | no disease)
    p2[i] <- y2[i] / sum(y2[])    # Pr(pattern i | disease)

    # Log-linear model for test pattern probability in disease-free
population
    log(y1[i]) <- lambda_coef[1,1] * y[i,1] +
                  lambda_coef[1,2] * y[i,2] +
                  lambda_coef[1,3] * y[i,3] +
                  lambda_coef[1,4] * y[i,4] +
                  lambda_coef[1,5] * y[i,2] * y[i,3]   # 2-way interaction
term

    # Log-linear model for test pattern probability in diseased population
    log(y2[i]) <- lambda_coef[2,1] * y[i,1] +
                  lambda_coef[2,2] * y[i,2] +
                  lambda_coef[2,3] * y[i,3] +
                  lambda_coef[2,4] * y[i,4] +
                  lambda_coef[2,5] * y[i,2] * y[i,3]   # 2-way interaction
term

    # Compute deviance contribution for each cell
    rd[i] <- 2 * ((mu[i] - r[i]) + r[i] * log(r[i] / mu[i]))
  }

  # Total deviance across all cells
  sumrd <- sum(rd[])

  # Prior for prevalence (Beta(1,1) = uniform)
  prev ~ dbeta(1, 1)

  # Sensitivity and specificity calculations
  for (t in 1:4) {

    sens[t] <- inprod(y[1:16, t], y2[1:16]) / sum(y2[1:16])      #
Sensitivity for test t
    fpr[t]  <- inprod(y[1:16, t], y1[1:16]) / sum(y1[1:16])      # False
positive rate
    spec[t] <- 1 - fpr[t]                                        #
Specificity

    youden[t] <- sens[t] + spec[t] - 1                           # Youden's
index
```

```
    # Acceptance indicator (1 if Youden's index ≥ 0)
    AcceptTest[t] ~ dbern(ifelse(youden[t] >= 0, 1, 0))

    # Priors for q parameters (on probability scale)
    q[2, t] ~ dbeta(1, 1)       # For diseased population
    q[1, t] ~ dbeta(1, 1)       # For non-diseased population

    # Logistic transformation to get regression coefficients
    for (d in 1:2) {
      lambda_coef[d, t] <- logit(q[d, t])
    }
  }

  # Priors for interaction terms in both populations
  lambda_coef[1,5] ~ dnorm(0, 1)
  lambda_coef[2,5] ~ dnorm(0, 1)

}
```

L-L DGM

R script to simulate data and fit L-L models

```
## This R script simulates data from L-L DGM based on the HIV data
## with the parameter settings specified in Appendix B and fits the
corresponding
## log-linear model with correct interactions.

rm(list = ls())                       # Clear R environment
require(R2jags)                       # Load R2jags for JAGS interface

start <- Sys.time()                  # Start timer

# ---------------------------
# Setup
# ---------------------------
folder <- "Chapter5-HIV_LL_LL"
if (!file.exists(folder)) dir.create(folder)  # Create output folder if
needed
set.seed(567)                                    # Ensure reproducibility

# ---------------------------
# Simulation Parameters
# ---------------------------
nobs     <- 2000                     # Number of individuals per dataset
ntests   <- 4                        # Number of diagnostic tests
nsim     <- 1250                     # Number of datasets to simulate
n.burnin <- 10000                    # MCMC burn-in iterations
n.iter   <- 50000                    # Total MCMC iterations
n.thin   <- 1                        # Thinning rate
n.chains <- 3                        # Number of MCMC chains

# ---------------------------
# True Parameter Values
# ---------------------------
prev <- 0.541                        # True disease prevalence
```

```
# Log-linear coefficients: lambda_coef[d, t]
# d = 1: non-diseased group, d = 2: diseased group
lambda_coef <- matrix(NA, nrow = 2, ncol = ntests)
lambda_coef[1, ] <- c(-3.456, -3.227, -5.641, -2.417)
lambda_coef[2, ] <- c( 5.742, -0.981,  1.711,  5.623)

lambda23 <- 1.398                    # 2-way interaction between Tests 2 and
3 in diseased group

# ---------------------------
# Generate True Probabilities
# ---------------------------
pattern <- as.matrix(expand.grid(rep(list(c(0, 1)), ntests)))  # All 2^4 =
16 possible test result patterns

log_prob0 <- rep(NA, 16)             # Log-probs under non-diseased group
log_prob1 <- rep(NA, 16)             # Log-probs under diseased group
p0 <- rep(NA, 16)                    # Pattern probabilities × (1 - prev)
p1 <- rep(NA, 16)                    # Pattern probabilities × prev

for (i in 1:16) {
  log_prob0[i] <- sum(lambda_coef[1, ] * pattern[i, ])
  log_prob1[i] <- sum(lambda_coef[2, ] * pattern[i, ]) + lambda23 *
pattern[i, 2] * pattern[i, 3]
}

for (i in 1:16) {
  p0[i] <- (1 - prev) * (exp(log_prob0[i]) / sum(exp(log_prob0)))  # Weight
by (1 - prev)
  p1[i] <- prev       * (exp(log_prob1[i]) / sum(exp(log_prob1)))  # Weight
by prev
}

prob <- p0 + p1                     # Final population-level probabilities

# ---------------------------
# Calculate True SE and SP
# ---------------------------
se <- sp <- rep(NA, ntests)

for (i in 1:ntests) {
  se[i] <- sum(p1[pattern[, i] == 1]) / sum(p1)  # Sensitivity: Pr(test = 1
| disease)
  sp[i] <- sum(p0[pattern[, i] == 0]) / sum(p0)  # Specificity: Pr(test = 0
| no disease)
}

# ---------------------------
# Simulate Observed Data
# ---------------------------
freq <- rmultinom(nsim, nobs, prob)  # Simulate frequencies under
multinomial distribution

Y <- array(NA, dim = c(nsim, 16, ntests + 1))  # Store test results and
their frequencies

for (i in 1:nsim) {
  Y[i, , 1:ntests]   <- pattern
  Y[i, , ntests + 1] <- freq[, i]
}
```

```r
# --------------------------
# JAGS Setup
# --------------------------
mymonitoredparamslist <- c("prev", "sens", "spec", "sumrd")

# Preallocate output objects
prevmedian <- prevlower <- prevupper <- ResD <- DIC <- rep(NA, nsim)
semedian   <- selower   <- seupper   <- matrix(NA, nrow = ntests, ncol =
nsim)
spmedian   <- splower   <- spupper   <- matrix(NA, nrow = ntests, ncol =
nsim)

# --------------------------
# Fit JAGS Model to Each Dataset
# --------------------------
for (j in 1:nsim) {

  mydatalist <- list(
    r = Y[j, , ntests + 1],      # Frequencies
    y = Y[j, , 1:ntests],        # Test patterns
    nobs = nobs,
    AcceptTest = rep(1, ntests) # All tests considered valid
  )

  fit <- jags(
    data = mydatalist,
    parameters.to.save = mymonitoredparamslist,
    model.file = "hiv_ll_ll.txt",  # JAGS model file
    n.chains = n.chains,
    n.iter   = n.iter,
    n.burnin = n.burnin,
    n.thin   = n.thin,
    progress.bar = "text"
  )

  # Convergence check: Rhat < 1.1 and n.eff > 400 for monitored parameters
  if (any(fit$BUGSoutput$summary[2:11, 8] > 1.1) |
      any(fit$BUGSoutput$summary[2:11, 9] < 400)) {

    # If not converged, store NA
    prevmedian[j] <- prevlower[j] <- prevupper[j] <- NA
    ResD[j] <- DIC[j] <- NA
    semedian[, j] <- selower[, j] <- seupper[, j] <- NA
    spmedian[, j] <- splower[, j] <- spupper[, j] <- NA

  } else {

    # If converged, store summaries
    prevmedian[j] <- fit$BUGSoutput$median$prev
    prevlower[j]  <- fit$BUGSoutput$summary[2, 3]
    prevupper[j]  <- fit$BUGSoutput$summary[2, 7]

    semedian[, j] <- fit$BUGSoutput$median$sens
    selower[, j]  <- fit$BUGSoutput$summary[3:6, 3]
    seupper[, j]  <- fit$BUGSoutput$summary[3:6, 7]

    spmedian[, j] <- fit$BUGSoutput$median$spec
    splower[, j]  <- fit$BUGSoutput$summary[7:10, 3]
    spupper[, j]  <- fit$BUGSoutput$summary[7:10, 7]
```

```
    ResD[j] <- fit$BUGSoutput$mean$sumrd
    DIC[j]  <- ResD[j] + fit$BUGSoutput$pD
  }
}

# --------------------------
# Save Final Outputs
# --------------------------
rm(fit)
save.image(file = file.path(folder, "HIV_LL_LL_2000.RData"))

# --------------------------
# End Timer
# --------------------------
print(Sys.time() - start)
```

JAGS script to fit the L-L model with correct interactions

The same script used for the latent trait DGM can also be applied to the L-L DGM, as the fitted L-L models assumed the same dependency structure as in the latent trait DGM.

JAGS script to fit the L-L model with all interactions (in the diseased group) and hyperlasso priors

```
model {

  for (i in 1:16) {

    # Likelihood for contingency table cell counts
    r[i] ~ dpois(mu[i])
    mu[i] <- p[i] * nobs                              # Expected counts

    # Mixture model of disease status
    p[i] <- (1 - prev) * p1[i] + prev * p2[i]

    # Cell probabilities for non-diseased and diseased
    p1[i] <- y1[i] / sum(y1[])
    p2[i] <- y2[i] / sum(y2[])

    # Log-linear model for non-diseased group (main effects only)
    log(y1[i]) <- lambda_coef[1,1] * y[i,1] +
                  lambda_coef[1,2] * y[i,2] +
                  lambda_coef[1,3] * y[i,3] +
                  lambda_coef[1,4] * y[i,4]

    # Log-linear model for diseased group (main effects + interactions)
    log(y2[i]) <- lambda_coef[2,1] * y[i,1] +
                  lambda_coef[2,2] * y[i,2] +
                  lambda_coef[2,3] * y[i,3] +
                  lambda_coef[2,4] * y[i,4] +
                  lambda_int[1] * y[i,1]*y[i,2] +
                  lambda_int[2] * y[i,1]*y[i,3] +
                  lambda_int[3] * y[i,1]*y[i,4] +
                  lambda_int[4] * y[i,2]*y[i,3] +
                  lambda_int[5] * y[i,2]*y[i,4] +
```

```
                  lambda_int[6] * y[i,3]*y[i,4]

    # Deviance residual
    rd[i] <- 2 * ((mu[i] - r[i]) + r[i] * log(r[i] / mu[i]))
  }

  sumrd <- sum(rd[])

  # Uniform prior for disease prevalence
  prev ~ dbeta(1, 1)

  for (t in 1:4) {
    # Diagnostic accuracy metrics
    sens[t] <- inprod(y[1:16, t], y2[1:16]) / sum(y2[1:16])
    fpr[t]  <- inprod(y[1:16, t], y1[1:16]) / sum(y1[1:16])
    spec[t] <- 1 - fpr[t]
    youden[t] <- sens[t] + spec[t] - 1

    # Indicator of test informativeness
    AcceptTest[t] ~ dbern(ifelse(youden[t] >= 0, 1, 0))

    # Main effect priors (shared for both groups)
    for (d in 1:2) {
      q[d, t] ~ dbeta(1, 1)
      lambda_coef[d, t] <- logit(q[d, t])
    }
  }

  # Hyper-LASSO priors for interaction terms (diseased group only)
  for (j in 1:6) {
    lambda_int[j] ~ ddexp(0, inv_scale_tau[j])           # Laplace prior
with scale = sqrt(tau_j)
    inv_scale_tau[j] <- 1 / sqrt(theta[j])
    theta[j] ~ dgamma(0.5, inv_theta2)                   # Local shrinkage
component
  }

  # Global shrinkage: Half-Cauchy(0,1)
  theta_global ~ dt(0, 1, 1) T(0,)
  inv_theta2 <- 1 / (theta_global^2)
}
```

JAGS script to fit the L-L model with all interactions (in the diseased group) and elastic net priors

```
model {

  for (i in 1:16) {

    # Likelihood for observed counts in each cell
    r[i] ~ dpois(mu[i])
    mu[i] <- p[i] * nobs

    # Mixture probability of test pattern given disease status
    p[i] <- (1 - prev) * p1[i] + prev * p2[i]

    # Probabilities for disease-free and diseased individuals
    p1[i] <- y1[i] / sum(y1[])
    p2[i] <- y2[i] / sum(y2[])
```

```
    # --- Log-linear model for the non-diseased group (main effects only) -
--
    log(y1[i]) <- lambda_coef[1,1]*y[i,1] + lambda_coef[1,2]*y[i,2] +
                  lambda_coef[1,3]*y[i,3] + lambda_coef[1,4]*y[i,4]

    # --- Log-linear model for diseased group: main effects + interactions
---
    log(y2[i]) <- lambda_coef[2,1]*y[i,1] + lambda_coef[2,2]*y[i,2] +
                  lambda_coef[2,3]*y[i,3] + lambda_coef[2,4]*y[i,4] +
                  lambda_int[1]*y[i,1]*y[i,2] +
                  lambda_int[2]*y[i,1]*y[i,3] +
                  lambda_int[3]*y[i,1]*y[i,4] +
                  lambda_int[4]*y[i,2]*y[i,3] +
                  lambda_int[5]*y[i,2]*y[i,4] +
                  lambda_int[6]*y[i,3]*y[i,4]

    # Deviance residual
    rd[i] <- 2 * ((mu[i] - r[i]) + r[i] * log(r[i] / mu[i]))
  }

  # Total residual deviance
  sumrd <- sum(rd[])

  # Prior for prevalence
  prev ~ dbeta(1, 1)

  # Main effects and diagnostic accuracy measures
  for (t in 1:4) {

    sens[t] <- inprod(y[1:16, t], y2[1:16]) / sum(y2[1:16])
    fpr[t]  <- inprod(y[1:16, t], y1[1:16]) / sum(y1[1:16])
    spec[t] <- 1 - fpr[t]
    youden[t] <- sens[t] + spec[t] - 1

    AcceptTest[t] ~ dbern(ifelse(youden[t] >= 0, 1, 0))

    for (d in 1:2) {
      q[d, t] ~ dbeta(1, 1)
      lambda_coef[d, t] <- logit(q[d, t])
    }
  }

  # --- Elastic Net Priors for Interaction Coefficients Only ---

  # Global shrinkage (Half-Cauchy)
  theta_1 ~ dt(0, 1, 1) T(0,)
  theta_2 ~ dt(0, 1, 1) T(0,)

  for (j in 1:6) {
    tau_jl[j] ~ dgamma(0.5, (8 * theta_2) / theta_1) T(1,)
    inv_var_lambda[j] <- tau_jl[j] * theta_2 / (tau_jl[j] - 1 + 1e-8)
    lambda_int[j] ~ dnorm(0, inv_var_lambda[j])
  }
}
```

JAGS script to fit the L-L model with all interactions (in the diseased group) and regularized horseshoe priors

```
model {
```

```
  for (i in 1:16) {

    # Likelihood for observed counts in each test pattern
    r[i] ~ dpois(mu[i])
    mu[i] <- p[i] * nobs                          # Expected count

    # Mixture model of disease status
    p[i] <- (1 - prev) * p1[i] + prev * p2[i]

    # Pattern probabilities conditional on disease status
    p1[i] <- y1[i] / sum(y1[])                    # Pr(pattern i | no
disease)
    p2[i] <- y2[i] / sum(y2[])                    # Pr(pattern i |
disease)

    # Log-linear model for the non-diseased group (main effects only)
    log(y1[i]) <- lambda_coef[1,1]*y[i,1] + lambda_coef[1,2]*y[i,2] +
                  lambda_coef[1,3]*y[i,3] + lambda_coef[1,4]*y[i,4]

    # Log-linear model for diseased group (main + interaction effects)
    log(y2[i]) <- lambda_coef[2,1]*y[i,1] + lambda_coef[2,2]*y[i,2] +
                  lambda_coef[2,3]*y[i,3] + lambda_coef[2,4]*y[i,4] +
                  lambda_int[1]*y[i,1]*y[i,2] +
                  lambda_int[2]*y[i,1]*y[i,3] +
                  lambda_int[3]*y[i,1]*y[i,4] +
                  lambda_int[4]*y[i,2]*y[i,3] +
                  lambda_int[5]*y[i,2]*y[i,4] +
                  lambda_int[6]*y[i,3]*y[i,4]

    # Deviance residual
    rd[i] <- 2 * ((mu[i] - r[i]) + r[i] * log(r[i] / mu[i]))
  }

  sumrd <- sum(rd[])

  # Prior for prevalence
  prev ~ dbeta(1, 1)

  # Main effects and diagnostic accuracy
  for (t in 1:4) {

    sens[t] <- inprod(y[1:16, t], y2[1:16]) / sum(y2[1:16])   # Sensitivity
    fpr[t]  <- inprod(y[1:16, t], y1[1:16]) / sum(y1[1:16])   # False
positive rate
    spec[t] <- 1 - fpr[t]                                     # Specificity
    youden[t] <- sens[t] + spec[t] - 1

    AcceptTest[t] ~ dbern(ifelse(youden[t] >= 0, 1, 0))       # Acceptance
indicator

    for (d in 1:2) {
      q[d, t] ~ dbeta(1, 1)
      lambda_coef[d, t] <- logit(q[d, t])                     # Main effect
coefficients
    }
  }

  # --- Regularized Horseshoe Priors on Interaction Coefficients Only ---
  theta ~ dt(0, 1, 1) T(0,)
  c2 <- 1 / inv_c2
```

```
  inv_c2 ~ dgamma(2, 8)

  for (t in 1:6) {
    lambda_int[t] ~ dnorm(0, prec_lambda[t])
    prec_lambda[t] <- 1 / (tau2_jl[t] * theta)
    tau2_jl[t] <- (c2 * delta2_jl[t]) /
                  (c2 + theta^2 * delta2_jl[t])
    delta2_jl[t] ~ dt(0, 1, 1) T(0,)
  }

}
```

VL Data

Latent Trait DGM

R script to simulate data and fit L-L models

```
## This R script simulates data from latent trait DGM based on the VL data
## with the parameter settings specified in Appendix B and fits the
corresponding
## log-linear model with correct interactions.

## Clear workspace
rm(list = ls())

## Load required package
require(R2jags)

## Record start time
start <- Sys.time()

## Create results folder if it does not exist
folder <- "Chapter5-VL_LT_LL"
if (!file.exists(folder)) dir.create(folder)

## Set seed for reproducibility
set.seed(567)

## Simulation settings
nobs <- 2000              # number of individuals per dataset
ntests <- 4               # number of diagnostic tests
nsim <- 1250              # number of simulated datasets
n.burnin <- 10000         # burn-in iterations
n.iter <- 50000           # total MCMC iterations
n.thin <- 1               # thinning interval
n.chains <- 3             # number of MCMC chains

## True values for data generation
prev <- 0.372
se <- c(0.849, 0.782, 0.693, 0.712)      # sensitivities
sp <- c(0.977, 0.918, 0.981, 0.985)      # specificities
b.RE1 <- c(0.44, 0.44, 4.36, 4.36)       # sensitivity latent loadings
b.RE2 <- c(0, 0, 0, 0)                   # specificity latent loadings
(fixed)

## Compute latent thresholds for sensitivity and specificity
```

```
a1 <- qnorm(se) * sqrt(1 + b.RE1^2)
a2 <- qnorm(sp) * sqrt(1 + b.RE2^2)

## Initialise data storage
r <- d <- matrix(NA, nsim, nobs)
s <- c <- p <- y <- array(NA, dim = c(nsim, nobs, ntests))

## Generate latent data for each dataset
for (k in 1:nsim) {
  r[k, ] <- rnorm(nobs)
  d[k, ] <- rbinom(nobs, 1, prev)
  for (j in 1:ntests) {
    s[k, , j] <- pnorm(a1[j] + b.RE1[j] * r[k, ])
    c[k, , j] <- pnorm(a2[j] + b.RE2[j] * r[k, ])
    p[k, , j] <- (s[k, , j]^d[k, ]) * ((1 - c[k, , j])^(1 - d[k, ]))
    y[k, , j] <- rbinom(nobs, 1, p[k, , j])
  }
}

## Function to tabulate test patterns
freqtable <- function(data) {
  freq <- rep(0, 16)
  patterns <- apply(data, 1, function(x) paste0(x, collapse = ""))
  tab <- table(patterns)
  names_vec <- apply(expand.grid(rep(list(c(0, 1)), 4)), 1, paste0,
collapse = "")
  freq[match(names(tab), names_vec)] <- tab
  cbind(expand.grid(c(0,1), c(0,1), c(0,1), c(0,1)), freq)
}

## Convert test results into 4-test pattern frequencies
Y <- array(NA, dim = c(nsim, 16, ntests + 1))
for (i in 1:nsim) {
  Y[i, , ] <- as.matrix(freqtable(y[i, , ]))
}

## Parameters to monitor in JAGS
mymonitoredparamslist <- c("prev", "sens", "spec", "sumrd")

## Initialise storage for posterior summaries
prevmedian <- prevlower <- prevupper <- rep(NA, nsim)
semedian <- selower <- seupper <- matrix(NA, nrow = ntests, ncol = nsim)
spmedian <- splower <- spupper <- matrix(NA, nrow = ntests, ncol = nsim)
ResD <- DIC <- rep(NA, nsim)

## Fit JAGS model to each dataset
for (j in 1:nsim) {
  mydatalist <- list(
    r = Y[j, , ntests + 1],
    y = Y[j, , 1:ntests],
    nobs = nobs,
    AcceptTest = rep(1, ntests)
  )

  fit <- jags(
    data = mydatalist,
    parameters.to.save = mymonitoredparamslist,
    model.file = "vl_lt_ll.txt",
    n.chains = n.chains,
    n.iter = n.iter,
    n.burnin = n.burnin,
```

```
    n.thin = n.thin,
    progress.bar = "text"
  )

  # Convergence check
  summ <- fit$BUGSoutput$summary
  if (any(summ[2:11, 8] > 1.1) || any(summ[2:11, 9] < 400)) next

  prevmedian[j] <- fit$BUGSoutput$median$prev
  prevlower[j] <- summ[2, 3]
  prevupper[j] <- summ[2, 7]
  semedian[, j] <- fit$BUGSoutput$median$sens
  selower[, j] <- summ[3:6, 3]
  seupper[, j] <- summ[3:6, 7]
  spmedian[, j] <- fit$BUGSoutput$median$spec
  splower[, j] <- summ[7:10, 3]
  spupper[, j] <- summ[7:10, 7]
  ResD[j] <- fit$BUGSoutput$mean$sumrd
  DIC[j] <- ResD[j] + fit$BUGSoutput$pD
}

## Clean up
rm(fit)

## Save workspace
save.image(file = file.path(folder, "VL_LT_LL_2000.RData"))

## Report total runtime
print(Sys.time() - start)
```

JAGS script to fit the L-L model with correct interactions

```
model {

  for (i in 1:16) {

    r[i] ~ dpois(mu[i])                         # Poisson likelihood for
observed counts
    mu[i] <- p[i] * nobs                        # Expected count for
pattern i

    p[i] <- (1 - prev) * p1[i] + prev * p2[i]   # Mixture of non-diseased
and diseased probabilities

    p1[i] <- y1[i] / sum(y1[])                  # Pr(pattern i | non-
diseased)
    p2[i] <- y2[i] / sum(y2[])                  # Pr(pattern i | diseased)

    # Log-linear model for non-diseased group: only main effects
    log(y1[i]) <- lambda_coef[1,1]*y[i,1] + lambda_coef[1,2]*y[i,2]
                + lambda_coef[1,3]*y[i,3] + lambda_coef[1,4]*y[i,4]

    # Log-linear model for diseased group: main + interaction effects
    log(y2[i]) <- lambda_coef[2,1]*y[i,1] + lambda_coef[2,2]*y[i,2]
                + lambda_coef[2,3]*y[i,3] + lambda_coef[2,4]*y[i,4]
                + lambda_coef[2,5]*y[i,1]*y[i,2]
                + lambda_coef[2,6]*y[i,1]*y[i,3]
                + lambda_coef[2,6]*y[i,1]*y[i,4]
                + lambda_coef[2,6]*y[i,2]*y[i,3]
                + lambda_coef[2,6]*y[i,2]*y[i,4]
```

```
                 + lambda_coef[2,7]*y[i,3]*y[i,4]

      # Residual deviance contribution
      rd[i] <- 2 * ((mu[i] - r[i]) + r[i] * log(r[i] / mu[i]))
    }

    # Total residual deviance
    sumrd <- sum(rd[])

    # Prior for prevalence
    prev ~ dbeta(1, 1)

    for (t in 1:4) {
      # Sensitivity and specificity calculations
      sens[t] <- inprod(y[1:16, t], y2[1:16]) / sum(y2[1:16])
      fpr[t] <- inprod(y[1:16, t], y1[1:16]) / sum(y1[1:16])
      spec[t] <- 1 - fpr[t]
      youden[t] <- sens[t] + spec[t] - 1

      # Test acceptance indicator
      AcceptTest[t] ~ dbern(ifelse(youden[t] >= 0, 1, 0))

      # Priors on main effect logits
      q[2, t] ~ dbeta(1, 1)
      q[1, t] ~ dbeta(1, 1)

      for (d in 1:2) {
        lambda_coef[d, t] <- logit(q[d, t])
      }
    }

    # Priors for interaction terms (in diseased group only)
    lambda_coef[2,5] ~ dnorm(0, 1)
    lambda_coef[2,6] ~ dnorm(0, 1)
    lambda_coef[2,7] ~ dnorm(0, 1)

}
```

Fixed-Effect DGM

R script to simulate data and fit L-L models

```
## This R script simulates data from fixed-effect DGM based on the VL data
## with the parameter settings specified in Appendix B and fits the
corresponding
## log-linear model with correct interactions.

## Clear workspace
rm(list = ls())

## Load required package
require(R2jags)

## Record start time
start <- Sys.time()

## Create results folder if it does not exist
folder <- "Chapter5-VL_MENTEN_LL"
```

```r
if (!file.exists(folder)) dir.create(folder)

## Set seed for reproducibility
set.seed(567)

## Simulation settings
nobs <- 2000          # number of individuals per dataset
ntests <- 4           # number of diagnostic tests
nsim <- 1250          # number of simulated datasets
n.burnin <- 10000     # burn-in iterations
n.iter <- 50000       # total MCMC iterations
n.thin <- 1           # thinning interval
n.chains <- 3         # number of MCMC chains

## True values for data generation
prev <- 0.370
se <- c(0.857, 0.779, 0.729, 0.749)    # test sensitivities
sp <- c(0.982, 0.918, 0.981, 0.985)    # test specificities

## Covariance terms for conditional dependencies among test results
covse12 <- -0.009
covse34 <-  0.125

## Generate all 4-test patterns (rows = 16 combinations, columns = 4 tests)
pattern <- as.matrix(expand.grid(rep(list(c(0,1)), ntests)))

## Initialise probabilities
p1 <- p0 <- rep(NA, 2^ntests)

## Compute probabilities under latent class model with specified
covariances
for (i in 1:2^ntests) {
  # Diseased group with dependencies
  term12 <- se[1]^pattern[i,1] * (1 - se[1])^(1 - pattern[i,1]) *
            se[2]^pattern[i,2] * (1 - se[2])^(1 - pattern[i,2]) +
            (-1)^(pattern[i,1] - pattern[i,2]) * covse12

  term34 <- se[3]^pattern[i,3] * (1 - se[3])^(1 - pattern[i,3]) *
            se[4]^pattern[i,4] * (1 - se[4])^(1 - pattern[i,4]) +
            (-1)^(pattern[i,3] - pattern[i,4]) * covse34

  p1[i] <- prev * term12 * term34

  # Non-diseased group (assumed independent)
  p0[i] <- (1 - prev) *
           prod(sp^(1 - pattern[i,]) * (1 - sp)^pattern[i,])
}

## Total probability for each test pattern
prob <- p0 + p1

## Simulate counts from multinomial for each dataset
freq <- rmultinom(nsim, nobs, prob)

## Build array for storing test results and pattern frequencies
Y <- array(NA, dim = c(nsim, 2^ntests, ntests + 1))
for (i in 1:nsim) {
  Y[i,,1:ntests] <- pattern
  Y[i,,ntests+1] <- freq[,i]
}
```

```r
## Parameters to monitor from JAGS
mymonitoredparamslist <- c("prev", "sens", "spec", "sumrd")

## Initialise storage for posterior summaries
prevmedian <- prevlower <- prevupper <- rep(NA, nsim)
semedian <- selower <- seupper <- matrix(NA, nrow = ntests, ncol = nsim)
spmedian <- splower <- spupper <- matrix(NA, nrow = ntests, ncol = nsim)
ResD <- DIC <- rep(NA, nsim)

## Fit model to each simulated dataset
for (j in 1:nsim) {
  mydatalist <- list(
    r = Y[j,,ntests+1],
    y = Y[j,,1:ntests],
    nobs = nobs,
    AcceptTest = rep(1, ntests)
  )

  fit <- jags(
    data = mydatalist,
    parameters.to.save = mymonitoredparamslist,
    model.file = "vl_menten_ll.txt",
    n.chains = n.chains,
    n.iter = n.iter,
    n.burnin = n.burnin,
    n.thin = n.thin,
    progress.bar = "text"
  )

  # Convergence check
  summ <- fit$BUGSoutput$summary
  if (any(summ[2:11, 8] > 1.1) || any(summ[2:11, 9] < 400)) {
    next  # skip dataset if not converged
  }

  # Extract posterior summaries
  prevmedian[j] <- fit$BUGSoutput$median$prev
  prevlower[j]  <- summ[2, 3]
  prevupper[j]  <- summ[2, 7]
  semedian[,j]  <- fit$BUGSoutput$median$sens
  selower[,j]   <- summ[3:6, 3]
  seupper[,j]   <- summ[3:6, 7]
  spmedian[,j]  <- fit$BUGSoutput$median$spec
  splower[,j]   <- summ[7:10, 3]
  spupper[,j]   <- summ[7:10, 7]
  ResD[j]       <- fit$BUGSoutput$mean$sumrd
  DIC[j]        <- ResD[j] + fit$BUGSoutput$pD
}

## Clean up
rm(fit)

## Save output to file
save.image(file = file.path(folder, "VL_MENTEN_LL_2000.RData"))

## Report total run time
print(Sys.time() - start)
```

Jags script to fit the L-L model with correct interactions

```
model {

  for (i in 1:16) {

    r[i] ~ dpois(mu[i])                       # Poisson likelihood for
contingency cell i
    mu[i] <- p[i] * nobs                     # Expected cell count

    p[i] <- (1 - prev) * p1[i] + prev * p2[i]   # Mixture model

    p1[i] <- y1[i] / sum(y1[])               # Pr(pattern i | not diseased)
    p2[i] <- y2[i] / sum(y2[])               # Pr(pattern i | diseased)

    # Log-linear model for the non-diseased group
    log(y1[i]) <- lambda_coef[1,1]*y[i,1] + lambda_coef[1,2]*y[i,2] +
                  lambda_coef[1,3]*y[i,3] + lambda_coef[1,4]*y[i,4]

    # Log-linear model for the diseased group (with interactions)
    log(y2[i]) <- lambda_coef[2,1]*y[i,1] + lambda_coef[2,2]*y[i,2] +
                  lambda_coef[2,3]*y[i,3] + lambda_coef[2,4]*y[i,4] +
                  lambda_coef[2,5]*y[i,1]*y[i,2] +
                  lambda_coef[2,6]*y[i,3]*y[i,4]

    # Residual deviance for model fit assessment
    rd[i] <- 2 * ((mu[i] - r[i]) + r[i] * log(r[i] / mu[i]))
  }

  sumrd <- sum(rd[])                         # Total residual deviance

  # Prior for prevalence
  prev ~ dbeta(1, 1)

  for (t in 1:4) {

    # Sensitivity and specificity for each test
    sens[t] <- inprod(y[1:16, t], y2[1:16]) / sum(y2[1:16])
    fpr[t]  <- inprod(y[1:16, t], y1[1:16]) / sum(y1[1:16])
    spec[t] <- 1 - fpr[t]

    youden[t] <- sens[t] + spec[t] - 1

    # Test validity check using Youden index
    AcceptTest[t] ~ dbern(ifelse(youden[t] >= 0, 1, 0))

    # Priors for test coefficients (logit-scale probabilities)
    q[2, t] ~ dbeta(1, 1)
    q[1, t] ~ dbeta(1, 1)

    for (d in 1:2) {
      lambda_coef[d, t] <- logit(q[d, t])
    }
  }

  # Priors for interaction terms (in diseased group only)
  lambda_coef[2,5] ~ dnorm(0, 1)
  lambda_coef[2,6] ~ dnorm(0, 1)
}
```

L-L DGM

R script to simulate data and fit L-L models

```
## This R script simulates data from L-L DGM based on the VL data
## with the parameter settings specified in Appendix B and fits the
corresponding
## log-linear model with correct interactions.


## Clear workspace
rm(list = ls())

## Load required package
require(R2jags)

## Record start time
start <- Sys.time()

## Create results folder if it does not exist
folder <- "Chapter5-VL_LL_LL"
if (!file.exists(folder)) dir.create(folder)

## Set seed for reproducibility
set.seed(567)

## Simulation settings
nobs <- 2000          # number of individuals per dataset
ntests <- 4           # number of diagnostic tests
nsim <- 1250          # number of simulated datasets
n.burnin <- 10000     # burn-in iterations
n.iter <- 50000       # total MCMC iterations
n.thin <- 1           # thinning interval
n.chains <- 3         # number of MCMC chains

## True values for data generation
prev <- 0.370
mu <- matrix(NA, nrow = 2, ncol = ntests)  # logistic coefficients
lambda_coef <- c(-0.435, 3.316)           # interaction terms (log-linear)

mu[1, ] <- c(-3.997, -2.412, -3.943, -4.182)  # No disease (specificity
logits)
mu[2, ] <- c( 2.140,  1.637, -1.176, -0.883)  # Disease (sensitivity
logits)

## Pattern matrix for all 16 binary response patterns
pattern <- as.matrix(expand.grid(rep(list(c(0, 1)), ntests)))

## Compute log-probabilities for each pattern
log_prob0 <- log_prob1 <- p0 <- p1 <- rep(NA, 2^ntests)

for (i in 1:16) {
  log_prob0[i] <- sum(mu[1, ] * pattern[i, ])
  log_prob1[i] <- sum(mu[2, ] * pattern[i, ]) +
                  lambda_coef[1] * pattern[i,1] * pattern[i,2] +
                  lambda_coef[2] * pattern[i,3] * pattern[i,4]
}
```

```
## Normalize to get probabilities
p0 <- (1 - prev) * exp(log_prob0) / sum(exp(log_prob0))
p1 <- prev * exp(log_prob1) / sum(exp(log_prob1))
prob <- p0 + p1

## Compute true sensitivity and specificity
se <- sp <- rep(NA, ntests)
for (i in 1:ntests) {
  se[i] <- sum(p1[pattern[, i] == 1]) / sum(p1)
  sp[i] <- sum(p0[pattern[, i] == 0]) / sum(p0)
}

## Simulate frequencies under multinomial
freq <- rmultinom(nsim, nobs, prob)

## Store test patterns and frequencies
Y <- array(NA, dim = c(nsim, 16, ntests + 1))
for (i in 1:nsim) {
  Y[i, , 1:ntests] <- pattern
  Y[i, , ntests + 1] <- freq[, i]
}

## Parameters to monitor in JAGS
mymonitoredparamslist <- c("prev", "sens", "spec", "sumrd")

## Initialise storage
prevmedian <- prevlower <- prevupper <- rep(NA, nsim)
semedian <- selower <- seupper <- matrix(NA, nrow = ntests, ncol = nsim)
spmedian <- splower <- spupper <- matrix(NA, nrow = ntests, ncol = nsim)
ResD <- DIC <- rep(NA, nsim)

## Fit JAGS model to each dataset
for (j in 1:nsim) {
  mydatalist <- list(
    r = Y[j, , ntests + 1],
    y = Y[j, , 1:ntests],
    nobs = nobs,
    AcceptTest = rep(1, ntests)
  )

  fit <- jags(
    data = mydatalist,
    parameters.to.save = mymonitoredparamslist,
    model.file = "vl_ll_ll.txt",
    n.chains = n.chains,
    n.iter = n.iter,
    n.burnin = n.burnin,
    n.thin = n.thin,
    progress.bar = "text"
  )

  # Check convergence using Rhat and effective sample size
  summ <- fit$BUGSoutput$summary
  if (any(summ[2:11, 8] > 1.1) || any(summ[2:11, 9] < 400)) next

  prevmedian[j] <- fit$BUGSoutput$median$prev
  prevlower[j]  <- summ[2, 3]
  prevupper[j]  <- summ[2, 7]
  semedian[, j] <- fit$BUGSoutput$median$sens
  selower[, j]  <- summ[3:6, 3]
  seupper[, j]  <- summ[3:6, 7]
```

```
  spmedian[, j] <- fit$BUGSoutput$median$spec
  splower[, j]  <- summ[7:10, 3]
  spupper[, j]  <- summ[7:10, 7]
  ResD[j] <- fit$BUGSoutput$mean$sumrd
  DIC[j]  <- ResD[j] + fit$BUGSoutput$pD
}

## Clean workspace
rm(fit)

## Save workspace
save.image(file = file.path(folder, "VL_LL_LL_2000.RData"))

## Report runtime
print(Sys.time() - start)
```

JAGS script to fit the L-L model with correct interactions

As the L-L DGM for the VL data assumed dependencies for the same test pairs as the fixed-effect DGM for the same data, the same L-L model was fitted to the data sets simulated from the L-L DGMs.

JAGS script to fit the L-L model with all interactions (in the diseased group) and hyperlasso priors

As the L-L model with all interactions (in the diseased group) and hyperlasso priors is identical to the one fitted for the HIV data, the same JAGS script was also used to fit the model for the VL data.

JAGS script to fit the L-L model with all interactions (in the diseased group) and elastic net priors

As the L-L model with all interactions (in the diseased group) and elastic net priors is identical to the one fitted for the HIV data, the same JAGS script was also used to fit the model for the VL data.

JAGS script to fit the L-L model with all interactions (in the diseased group) and regularized horseshoe priors

As the L-L model with all interactions (in the diseased group) and regularized horseshoe priors is identical to the one fitted for the HIV data, the same JAGS script was also used to fit the model for the VL data.

CPTB Data

Latent Trait DGM

R script to simulate data and fit L-L models

```
## This R script simulates data from latent trait DGM based on the CPTB
data
## with the parameter settings specified in Appendix B and fits the
corresponding
## log-linear model with correct interactions.

# Clear workspace
rm(list = ls())

# Load required package
require(R2jags)

# Start timing the simulation
start <- Sys.time()

# Set results folder
folder <- "Chapter5-CPTB_LT_LL"
if (!file.exists(folder)) dir.create(folder)

# Set seed for reproducibility
set.seed(567)

# Simulation settings
nobs <- 2000            # Number of individuals per simulated dataset
ntests <- 5             # Number of diagnostic tests
nsim <- 1250            # Number of simulated datasets
n.burnin <- 10000       # Burn-in iterations for MCMC
n.iter <- 50000         # Total MCMC iterations
n.thin <- 1             # Thinning interval
n.chains <- 3           # Number of MCMC chains

# True parameter values
prev <- 0.292
se <- c(0.564, 0.461, 0.201, 0.686, 0.646)    # Sensitivities
sp <- c(0.999, 0.989, 0.997, 0.678, 0.797)    # Specificities
b.RE1 <- c(2.804, 2.804, 2.804, 0.023, 0.0)   # Random effect loadings (se)
b.RE2 <- c(0, 0, 0, 0, 0)                     # Random effect loadings (sp)

# Compute latent thresholds from inverse probit
a1 <- qnorm(se) * sqrt(1 + b.RE1^2)
a2 <- qnorm(sp) * sqrt(1 + b.RE2^2)

# Storage arrays for simulated data
r <- d <- matrix(NA, nsim, nobs)
s <- c <- p <- y <- array(NA, dim = c(nsim, nobs, ntests))

# Generate data from latent trait model
for (k in 1:nsim) {
  for (i in 1:nobs) {
    r[k, i] <- rnorm(1)                             # Latent trait
    d[k, i] <- rbinom(1, 1, prev)                   # Disease status
```

```
    for (j in 1:ntests) {
      s[k, i, j] <- pnorm(a1[j] + b.RE1[j] * r[k, i])         # Se
      c[k, i, j] <- pnorm(a2[j] + b.RE2[j] * r[k, i])         # Sp
      p[k, i, j] <- (s[k, i, j]^d[k, i]) * ((1 - c[k, i, j])^(1 - d[k, i]))
      y[k, i, j] <- rbinom(1, 1, p[k, i, j])       # Simulated test outcome
    }
  }
}

# Function to convert binary test patterns to frequency counts
freqtable <- function(data) {
  freq <- rep(0, 32)
  pats <- apply(data, 1, function(x) paste0(x, collapse = ""))
  pattern_names <- apply(expand.grid(rep(list(c(0, 1)), 5)), 1, paste0,
collapse = "")
  tpats <- table(factor(pats, levels = pattern_names))
  freq[] <- as.numeric(tpats)
  cbind(expand.grid(rep(list(c(0, 1)), 5)), freq)
}

# Aggregate frequencies for each simulated dataset
Y <- array(NA, dim = c(nsim, 2^ntests, ntests + 1))
for (i in 1:nsim) {
  Y[i, , ] <- as.matrix(freqtable(y[i, , ]))
}

# Parameters to monitor from JAGS output
mymonitoredparamslist <- c("prev", "sens", "spec", "sumrd")

# Create storage objects for posterior summaries
prevmedian <- prevlower <- prevupper <- rep(NA, nsim)
semedian <- selower <- seupper <- matrix(NA, nrow = ntests, ncol = nsim)
spmedian <- splower <- spupper <- matrix(NA, nrow = ntests, ncol = nsim)
ResD <- DIC <- rep(NA, nsim)

# Fit the model to each dataset using JAGS
for (j in 1:nsim) {
  mydatalist <- list(
    r = Y[j, , ntests + 1],
    y = Y[j, , 1:ntests],
    nobs = nobs,
    AcceptTest = rep(1, ntests)
  )

  fit <- jags(
    data = mydatalist,
    parameters.to.save = mymonitoredparamslist,
    model.file = "cptb_lt_ll.txt",  # JAGS model file
    n.chains = n.chains,
    n.iter = n.iter,
    n.burnin = n.burnin,
    n.thin = n.thin,
    progress.bar = "text"
  )

  # Check for convergence based on Rhat and effective sample size
  summ <- fit$BUGSoutput$summary
  if (any(summ[2:11 + ntests, 8] > 1.1) || any(summ[2:11 + ntests, 9] <
400)) {
    next
  }
```

```
  # Extract posterior summaries
  prevmedian[j] <- fit$BUGSoutput$median$prev
  prevlower[j] <- summ[2, 3]
  prevupper[j] <- summ[2, 7]
  semedian[, j] <- fit$BUGSoutput$median$sens
  selower[, j] <- summ[3:(2 + ntests), 3]
  seupper[, j] <- summ[3:(2 + ntests), 7]
  spmedian[, j] <- fit$BUGSoutput$median$spec
  splower[, j] <- summ[(3 + ntests):(2 + 2 * ntests), 3]
  spupper[, j] <- summ[(3 + ntests):(2 + 2 * ntests), 7]
  ResD[j] <- fit$BUGSoutput$mean$sumrd
  DIC[j] <- ResD[j] + fit$BUGSoutput$pD
}

# Clean workspace
rm(fit)

# Save all results
save.image(file = file.path(folder, "CPTB_LT_LL_2000.RData"))

# Report total execution time
print(Sys.time() - start)
```

JAGS script to fit the L-L model with correct interactions

```
model {

  for (i in 1:32) {

    r[i] ~ dpois(mu[i])                        # Poisson likelihood for
observed count

    mu[i] <- p[i] * nobs                       # Expected count

    p[i] <- (1 - prev) * p1[i] + prev * p2[i]  # Mixture probability

    p1[i] <- y1[i] / sum(y1[])                 # P(pattern i | non-diseased)
    p2[i] <- y2[i] / sum(y2[])                 # P(pattern i | diseased)

    # Log-linear model for non-diseased class (main effects only)
    log(y1[i]) <-
        lambda_coef[1,1]*y[i,1] +
        lambda_coef[1,2]*y[i,2] +
        lambda_coef[1,3]*y[i,3] +
        lambda_coef[1,4]*y[i,4] +
        lambda_coef[1,5]*y[i,5]

    # Log-linear model for diseased class (main effects + selected
interactions)
    log(y2[i]) <-
        lambda_coef[2,1]*y[i,1] +
        lambda_coef[2,2]*y[i,2] +
        lambda_coef[2,3]*y[i,3] +
        lambda_coef[2,4]*y[i,4] +
        lambda_coef[2,5]*y[i,5] +
        lambda_coef[2,6]*y[i,1]*y[i,2] +
        lambda_coef[2,6]*y[i,1]*y[i,3] +
        lambda_coef[2,7]*y[i,1]*y[i,4] +
        lambda_coef[2,6]*y[i,2]*y[i,3] +
```

```
        lambda_coef[2,7]*y[i,2]*y[i,4] +
        lambda_coef[2,7]*y[i,3]*y[i,4]

    # Deviance contribution
    rd[i] <- 2 * ((mu[i] - r[i]) + r[i] * log(r[i] / mu[i]))
  }

  sumrd <- sum(rd[])                          # Total residual deviance

  prev ~ dbeta(1, 1)                          # Uniform prior for prevalence

  for (t in 1:5) {
    sens[t] <- inprod(y[1:32, t], y2[1:32]) / sum(y2[1:32])  # Sensitivity
    fpr[t]  <- inprod(y[1:32, t], y1[1:32]) / sum(y1[1:32])  # False
positive rate
    spec[t] <- 1 - fpr[t]                                    # Specificity

    youden[t] <- sens[t] + spec[t] - 1

    AcceptTest[t] ~ dbern(ifelse(youden[t] >= 0, 1, 0))      # Filter bad
tests

    q[1,t] ~ dbeta(1,1)
    q[2,t] ~ dbeta(1,1)

    for (d in 1:2) {
      lambda_coef[d,t] <- logit(q[d,t])                     # Main effects
    }
  }

  # Priors for interaction terms in diseased class
  lambda_coef[2,6] ~ dnorm(0, 1)    # Shared across selected interactions
  lambda_coef[2,7] ~ dnorm(0, 1)    # Shared across other selected
interactions
}
```

Fixed-Effect DGM

R script to simulate data and fit L-L models

```
## This R script simulates data from fixed-effect DGM based on the CPTB
data
## with the parameter settings specified in Appendix B and fits the
corresponding
## log-linear model with correct interactions.


rm(list = ls())  # Clear the workspace

require(R2jags)  # Load the JAGS interface for R

start <- Sys.time()  # Start timing

# Create a folder to save output
folder <- "Chapter5-CPTB_WANG_LL"
if(!file.exists(folder)) dir.create(folder)

set.seed(567)  # Set seed for reproducibility
```

```
# Simulation settings
nobs <- 2000          # Number of observations per dataset
ntests <- 5           # Number of diagnostic tests
nsim <- 1250          # Number of datasets to simulate
n.burnin <- 10000     # Number of burn-in iterations
n.iter <- 50000       # Total iterations per chain
n.thin <- 1           # Thinning
n.chains <- 3         # Number of MCMC chains

# True parameters
prev <- 0.227
se <- c(0.689, 0.572, 0.267, 0.707, 0.647)
sp <- c(0.997, 0.988, 1.000, 0.659, 0.760)

# Dependence terms for the diseased population
covS0000=0.008; covS1000=-0.043; covS0100=-0.018; covS1100=-0.052
covS0010=-0.006; covS1010=-0.017; covS0110=-0.009; covS1110=0.127
covS0001=0.115; covS1001=0; covS0101=-0.057; covS1101=0.039
covS0011=-0.016; covS1011=-0.049; covS0111=-0.027; covS1111=-0.005

# Allocate probability vector for each of the 32 response patterns
prob <- rep(NA, 2^ntests)

# Probability of observing test pattern 1:
prob[1] = prev*((1-se[1])*(1-se[2])*(1-se[3])*(1-se[4])+covS0000)*(1-
se[5])+(1-prev)*sp[1]*sp[2]*sp[3]*sp[4]*sp[5]

# Probability of observing test pattern 2:
prob[2] = prev*(se[1]*(1-se[2])*(1-se[3])*(1-se[4])+covS1000)*(1-se[5])+(1-
prev)*(1-sp[1])*sp[2]*sp[3]*sp[4]*sp[5]

# Probability of observing test pattern 3:
prob[3] = prev*((1-se[1])*se[2]*(1-se[3])*(1-se[4])+covS0100)*(1-se[5])+(1-
prev)*sp[1]*(1-sp[2])*sp[3]*sp[4]*sp[5]

# Probability of observing test pattern 4:
prob[4] = prev*(se[1]*se[2]*(1-se[3])*(1-se[4])+covS1100)*(1-se[5])+(1-
prev)*(1-sp[1])*(1-sp[2])*sp[3]*sp[4]*sp[5]

# Probability of observing test pattern 5:
prob[5] = prev*((1-se[1])*(1-se[2])*se[3]*(1-se[4])+covS0010)*(1-se[5])+(1-
prev)*sp[1]*sp[2]*(1-sp[3])*sp[4]*sp[5]

# Probability of observing test pattern 6:
prob[6] = prev*(se[1]*(1-se[2])*se[3]*(1-se[4])+covS1010)*(1-se[5])+(1-
prev)*(1-sp[1])*sp[2]*(1-sp[3])*sp[4]*sp[5]

# Probability of observing test pattern 7:
prob[7] = prev*((1-se[1])*se[2]*se[3]*(1-se[4])+covS0110)*(1-se[5])+(1-
prev)*sp[1]*(1-sp[2])*(1-sp[3])*sp[4]*sp[5]

# Probability of observing test pattern 8:
prob[8] = prev*(se[1]*se[2]*se[3]*(1-se[4])+covS1110)*(1-se[5])+(1-
prev)*(1-sp[1])*(1-sp[2])*(1-sp[3])*sp[4]*sp[5]

# Probability of observing test pattern 9:
prob[9] = prev*((1-se[1])*(1-se[2])*(1-se[3])*se[4]+covS0001)*(1-se[5])+(1-
prev)*sp[1]*sp[2]*sp[3]*(1-sp[4])*sp[5]

# Probability of observing test pattern 10:
```

```
prob[10] = prev*(se[1]*(1-se[2])*(1-se[3])*se[4]+covS1001)*(1-se[5])+(1-
prev)*(1-sp[1])*sp[2]*sp[3]*(1-sp[4])*sp[5]

# Probability of observing test pattern 11:
prob[11] = prev*((1-se[1])*se[2]*(1-se[3])*se[4]+covS0101)*(1-se[5])+(1-
prev)*sp[1]*(1-sp[2])*sp[3]*(1-sp[4])*sp[5]

# Probability of observing test pattern 12:
prob[12] = prev*(se[1]*se[2]*(1-se[3])*se[4]+covS1101)*(1-se[5])+(1-
prev)*(1-sp[1])*(1-sp[2])*sp[3]*(1-sp[4])*sp[5]

# Probability of observing test pattern 13:
prob[13] = prev*((1-se[1])*(1-se[2])*se[3]*se[4]+covS0011)*(1-se[5])+(1-
prev)*sp[1]*sp[2]*(1-sp[3])*(1-sp[4])*sp[5]

# Probability of observing test pattern 14:
prob[14] = prev*(se[1]*(1-se[2])*se[3]*se[4]+covS1011)*(1-se[5])+(1-
prev)*(1-sp[1])*sp[2]*(1-sp[3])*(1-sp[4])*sp[5]

# Probability of observing test pattern 15:
prob[15] = prev*((1-se[1])*se[2]*se[3]*se[4]+covS0111)*(1-se[5])+(1-
prev)*sp[1]*(1-sp[2])*(1-sp[3])*(1-sp[4])*sp[5]

# Probability of observing test pattern 16:
prob[16] = prev*(se[1]*se[2]*se[3]*se[4]+covS1111)*(1-se[5])+(1-prev)*(1-
sp[1])*(1-sp[2])*(1-sp[3])*(1-sp[4])*sp[5]

# Probability of observing test pattern 17:
prob[17] = prev*((1-se[1])*(1-se[2])*(1-se[3])*(1-
se[4])+covS0000)*se[5]+(1-prev)*sp[1]*sp[2]*sp[3]*sp[4]*(1-sp[5])

# Probability of observing test pattern 18:
prob[18] = prev*(se[1]*(1-se[2])*(1-se[3])*(1-se[4])+covS1000)*se[5]+(1-
prev)*(1-sp[1])*sp[2]*sp[3]*sp[4]*(1-sp[5])

# Probability of observing test pattern 19:
prob[19] = prev*((1-se[1])*se[2]*(1-se[3])*(1-se[4])+covS0100)*se[5]+(1-
prev)*sp[1]*(1-sp[2])*sp[3]*sp[4]*(1-sp[5])

# Probability of observing test pattern 20:
prob[20] = prev*(se[1]*se[2]*(1-se[3])*(1-se[4])+covS1100)*se[5]+(1-
prev)*(1-sp[1])*(1-sp[2])*sp[3]*sp[4]*(1-sp[5])

# Probability of observing test pattern 21:
prob[21] = prev*((1-se[1])*(1-se[2])*se[3]*(1-se[4])+covS0010)*se[5]+(1-
prev)*sp[1]*sp[2]*(1-sp[3])*sp[4]*(1-sp[5])

# Probability of observing test pattern 22:
prob[22] = prev*(se[1]*(1-se[2])*se[3]*(1-se[4])+covS1010)*se[5]+(1-
prev)*(1-sp[1])*sp[2]*(1-sp[3])*sp[4]*(1-sp[5])

# Probability of observing test pattern 23:
prob[23] = prev*((1-se[1])*se[2]*se[3]*(1-se[4])+covS0110)*se[5]+(1-
prev)*sp[1]*(1-sp[2])*(1-sp[3])*sp[4]*(1-sp[5])

# Probability of observing test pattern 24:
prob[24] = prev*(se[1]*se[2]*se[3]*(1-se[4])+covS1110)*se[5]+(1-prev)*(1-
sp[1])*(1-sp[2])*(1-sp[3])*sp[4]*(1-sp[5])

# Probability of observing test pattern 25:
```

```
prob[25] = prev*((1-se[1])*(1-se[2])*(1-se[3])*se[4]+covS0001)*se[5]+(1-
prev)*sp[1]*sp[2]*sp[3]*(1-sp[4])*(1-sp[5])

# Probability of observing test pattern 26:
prob[26] = prev*(se[1]*(1-se[2])*(1-se[3])*se[4]+covS1001)*se[5]+(1-
prev)*(1-sp[1])*sp[2]*sp[3]*(1-sp[4])*(1-sp[5])

# Probability of observing test pattern 27:
prob[27] = prev*((1-se[1])*se[2]*(1-se[3])*se[4]+covS0101)*se[5]+(1-
prev)*sp[1]*(1-sp[2])*sp[3]*(1-sp[4])*(1-sp[5])

# Probability of observing test pattern 28:
prob[28] = prev*(se[1]*se[2]*(1-se[3])*se[4]+covS1101)*se[5]+(1-prev)*(1-
sp[1])*(1-sp[2])*sp[3]*(1-sp[4])*(1-sp[5])

# Probability of observing test pattern 29:
prob[29] = prev*((1-se[1])*(1-se[2])*se[3]*se[4]+covS0011)*se[5]+(1-
prev)*sp[1]*sp[2]*(1-sp[3])*(1-sp[4])*(1-sp[5])

# Probability of observing test pattern 30:
prob[30] = prev*(se[1]*(1-se[2])*se[3]*se[4]+covS1011)*se[5]+(1-prev)*(1-
sp[1])*sp[2]*(1-sp[3])*(1-sp[4])*(1-sp[5])

# Probability of observing test pattern 31:
prob[31] = prev*((1-se[1])*se[2]*se[3]*se[4]+covS0111)*se[5]+(1-
prev)*sp[1]*(1-sp[2])*(1-sp[3])*(1-sp[4])*(1-sp[5])

# Probability of observing test pattern 32:
prob[32] = prev*(se[1]*se[2]*se[3]*se[4]+covS1111)*se[5]+(1-prev)*(1-
sp[1])*(1-sp[2])*(1-sp[3])*(1-sp[4])*(1-sp[5])

# Simulate counts from multinomial using these probabilities
freq <- rmultinom(nsim, nobs, prob)

# Create Y array to hold each simulated dataset
Y <- array(NA, dim = c(nsim, 2^ntests, ntests + 1))
for(i in 1:nsim){
  Y[i,,1:ntests] <- as.matrix(expand.grid(rep(list(c(0,1)), ntests)))
  Y[i,,ntests+1] <- freq[,i]
}

# Parameters to monitor from JAGS
mymonitoredparamslist <- c("prev", "sens", "spec", "sumrd")

# Initialise storage matrices for results
prevmedian <- rep(NA, nsim)
prevlower  <- rep(NA, nsim)
prevupper  <- rep(NA, nsim)
semedian   <- matrix(NA, nrow=ntests, ncol=nsim)
selower    <- matrix(NA, nrow=ntests, ncol=nsim)
seupper    <- matrix(NA, nrow=ntests, ncol=nsim)
spmedian   <- matrix(NA, nrow=ntests, ncol=nsim)
splower    <- matrix(NA, nrow=ntests, ncol=nsim)
spupper    <- matrix(NA, nrow=ntests, ncol=nsim)
ResD       <- rep(NA, nsim)
DIC        <- rep(NA, nsim)

# Fit JAGS model for each simulated dataset
for(j in 1:nsim){
  mydatalist <- list(r = Y[j,,ntests+1], y = Y[j,,1:ntests], nobs = nobs,
AcceptTest = rep(1, ntests))
```

```
  fit <- jags(data = mydatalist,
              parameters.to.save = mymonitoredparamslist,
              model.file = "cptb_wang_ll.txt",
              n.chains = n.chains,
              n.iter = n.iter,
              n.burnin = n.burnin,
              n.thin = n.thin,
              progress.bar = "text")

  # Check convergence
  if(any(fit$BUGSoutput$summary[2:13,8] > 1.1) |
any(fit$BUGSoutput$summary[2:13,9] < 400)){
    prevmedian[j] <- prevlower[j] <- prevupper[j] <- ResD[j] <- DIC[j] <-
NA
    semedian[,j] <- selower[,j] <- seupper[,j] <- spmedian[,j] <-
splower[,j] <- spupper[,j] <- NA
  } else {
    prevmedian[j] <- fit$BUGSoutput$median$prev
    prevlower[j]  <- fit$BUGSoutput$summary["prev",3]
    prevupper[j]  <- fit$BUGSoutput$summary["prev",7]
    semedian[,j]  <- fit$BUGSoutput$median$sens
    selower[,j]   <- fit$BUGSoutput$summary[3:7,3]
    seupper[,j]   <- fit$BUGSoutput$summary[3:7,7]
    spmedian[,j]  <- fit$BUGSoutput$median$spec
    splower[,j]   <- fit$BUGSoutput$summary[8:12,3]
    spupper[,j]   <- fit$BUGSoutput$summary[8:12,7]
    ResD[j]       <- fit$BUGSoutput$mean$sumrd
    DIC[j]        <- ResD[j] + fit$BUGSoutput$pD
  }
}


# Clean workspace
rm(fit)

# Save all results
save.image(file = file.path(folder, "CPTB_LT_LL_2000.RData"))

# Report total execution time
print(Sys.time() - start)
```

JAGS script to fit the L-L model with correct interactions

```
model {

  for (i in 1:32) {

    r[i] ~ dpois(mu[i])                    # Poisson likelihood for
observed count

    mu[i] <- p[i] * nobs                   # Expected count

    p[i] <- (1 - prev) * p1[i] + prev * p2[i]  # Mixture probability

    p1[i] <- y1[i] / sum(y1[])             # P(pattern i | non-diseased)
    p2[i] <- y2[i] / sum(y2[])             # P(pattern i | diseased)

    # Log-linear model for non-diseased class (main effects only)
    log(y1[i]) <-
```

```
        lambda_coef[1,1]*y[i,1] +
        lambda_coef[1,2]*y[i,2] +
        lambda_coef[1,3]*y[i,3] +
        lambda_coef[1,4]*y[i,4] +
        lambda_coef[1,5]*y[i,5]

    # Log-linear model for diseased class (main effects + selected
interactions)
    log(y2[i]) <-
        lambda_coef[2,1]*y[i,1] +
        lambda_coef[2,2]*y[i,2] +
        lambda_coef[2,3]*y[i,3] +
        lambda_coef[2,4]*y[i,4] +
        lambda_coef[2,5]*y[i,5] +
        lambda_coef[2,6]*y[i,1]*y[i,2] +
        lambda_coef[2,7]*y[i,1]*y[i,3] +
        lambda_coef[2,8]*y[i,1]*y[i,4] +
        lambda_coef[2,9]*y[i,2]*y[i,3] +
        lambda_coef[2,10]*y[i,2]*y[i,4] +
        lambda_coef[2,11]*y[i,3]*y[i,4]

    # Deviance contribution
    rd[i] <- 2 * ((mu[i] - r[i]) + r[i] * log(r[i] / mu[i]))
  }

  sumrd <- sum(rd[])                          # Total residual deviance

  prev ~ dbeta(1, 1)                          # Uniform prior for prevalence

  for (t in 1:5) {
    sens[t] <- inprod(y[1:32, t], y2[1:32]) / sum(y2[1:32])  # Sensitivity
    fpr[t]  <- inprod(y[1:32, t], y1[1:32]) / sum(y1[1:32])  # False
positive rate
    spec[t] <- 1 - fpr[t]                                    # Specificity

    youden[t] <- sens[t] + spec[t] - 1

    AcceptTest[t] ~ dbern(ifelse(youden[t] >= 0, 1, 0))     # Filter bad
tests

    q[1,t] ~ dbeta(1,1)
    q[2,t] ~ dbeta(1,1)

    for (d in 1:2) {
      lambda_coef[d,t] <- logit(q[d,t])                     # Main effects
    }
  }

  # Priors for interaction terms in diseased class
  lambda_coef[2,6] ~ dnorm(0, 1)
  lambda_coef[2,7] ~ dnorm(0, 1)
  lambda_coef[2,8] ~ dnorm(0, 1)
  lambda_coef[2,9] ~ dnorm(0, 1)
  lambda_coef[2,10] ~ dnorm(0, 1)
  lambda_coef[2,11] ~ dnorm(0, 1)
}
```

L-L DGM

R script to simulate data and fit L-L models

```
## This R script simulates data from L-L DGM based on the CPTB data
## with the parameter settings specified in Appendix B and fits the
corresponding
## log-linear model with correct interactions.


rm(list = ls())  # Clear workspace

require(R2jags)  # Load JAGS interface

start <- Sys.time()  # Track start time

# Set output folder
folder <- "Chapter5-CPTB_LL_LL"
if (!file.exists(folder)) dir.create(folder)

set.seed(567)  # Reproducibility

# Simulation and model settings
nobs <- 2000          # Observations per dataset
ntests <- 5           # Number of diagnostic tests
nsim <- 1250          # Number of simulated datasets
n.burnin <- 10000     # Burn-in iterations
n.iter <- 50000       # Total iterations
n.thin <- 1           # Thinning
n.chains <- 3         # MCMC chains

# Initialise vectors
log_prob1 <- rep(NA, 2^ntests)
log_prob0 <- rep(NA, 2^ntests)
p1 <- rep(NA, 2^ntests)
p0 <- rep(NA, 2^ntests)
se <- rep(NA, ntests)
sp <- rep(NA, ntests)

# Latent logistic coefficients
mu <- matrix(NA, nrow = 2, ncol = ntests)
prev <- 0.227

# Interaction coefficients for diseased individuals
lambda_coef12 <- 2.193
lambda_coef13 <- 0.232
lambda_coef14 <- -0.848
lambda_coef23 <- 1.865
lambda_coef24 <- -0.598
lambda_coef34 <- -2.656

# Main effects for test outcomes under no disease (row 1) and disease (row
2)
mu[1, ] <- c(-5.850, -4.410, -8.394, -0.660, -1.154)
mu[2, ] <- c(0.440, -1.113, -0.655, 3.350, 0.610)

# Generate all 32 binary patterns (rows of contingency table)
pattern <- as.matrix(expand.grid(rep(list(c(0, 1)), ntests)))
```

```r
# Calculate log-probabilities for each pattern
for (i in 1:2^ntests) {
  log_prob0[i] <- sum(mu[1, ] * pattern[i, ])
  log_prob1[i] <- sum(mu[2, ] * pattern[i, ]) +
    lambda_coef12 * pattern[i, 1] * pattern[i, 2] +
    lambda_coef13 * pattern[i, 1] * pattern[i, 3] +
    lambda_coef14 * pattern[i, 1] * pattern[i, 4] +
    lambda_coef23 * pattern[i, 2] * pattern[i, 3] +
    lambda_coef24 * pattern[i, 2] * pattern[i, 4] +
    lambda_coef34 * pattern[i, 3] * pattern[i, 4]
}

# Convert log-probabilities to joint probabilities
for (i in 1:2^ntests) {
  p0[i] <- (1 - prev) * (exp(log_prob0[i]) / sum(exp(log_prob0)))
  p1[i] <- prev * (exp(log_prob1[i]) / sum(exp(log_prob1)))
}
prob <- p0 + p1

# True sensitivity and specificity (for reference)
for (i in 1:ntests) {
  se[i] <- sum(p1[pattern[, i] == 1]) / sum(p1)
  sp[i] <- sum(p0[pattern[, i] == 0]) / sum(p0)
}

# Simulate frequency tables
freq <- rmultinom(nsim, nobs, prob)

# Store datasets: 2^ntests patterns, ntests columns, +1 for frequencies
Y <- array(NA, dim = c(nsim, 2^ntests, ntests + 1))
for (i in 1:nsim) {
  Y[i, , 1:ntests] <- pattern
  Y[i, , ntests + 1] <- freq[, i]
}

# Parameters to monitor from JAGS output
mymonitoredparamslist <- c("prev", "sens", "spec", "sumrd")

# Initialise output storage
prevmedian <- rep(NA, nsim)
prevlower <- rep(NA, nsim)
prevupper <- rep(NA, nsim)
semedian <- matrix(NA, nrow = ntests, ncol = nsim)
selower <- matrix(NA, nrow = ntests, ncol = nsim)
seupper <- matrix(NA, nrow = ntests, ncol = nsim)
spmedian <- matrix(NA, nrow = ntests, ncol = nsim)
splower <- matrix(NA, nrow = ntests, ncol = nsim)
spupper <- matrix(NA, nrow = ntests, ncol = nsim)
ResD <- rep(NA, nsim)
DIC <- rep(NA, nsim)

# Fit model to each simulated dataset
for (j in 1:nsim) {
  mydatalist <- list(
    r = Y[j, , ntests + 1],
    y = Y[j, , 1:ntests],
    nobs = nobs,
    AcceptTest = rep(1, ntests)
  )
```

```
  fit <- jags(
    data = mydatalist,
    parameters.to.save = mymonitoredparamslist,
    model.file = "cptb_ll_ll.txt",
    n.chains = n.chains,
    n.iter = n.iter,
    n.burnin = n.burnin,
    n.thin = n.thin,
    progress.bar = "text"
  )

  # Convergence check
  if (any(fit$BUGSoutput$summary[2:13, 8] > 1.1) |
any(fit$BUGSoutput$summary[2:13, 9] < 400)) {
    prevmedian[j] <- prevlower[j] <- prevupper[j] <- ResD[j] <- DIC[j] <-
NA
    semedian[, j] <- selower[, j] <- seupper[, j] <- NA
    spmedian[, j] <- splower[, j] <- spupper[, j] <- NA
  } else {
    prevmedian[j] <- fit$BUGSoutput$median$prev
    prevlower[j] <- fit$BUGSoutput$summary[2, 3]
    prevupper[j] <- fit$BUGSoutput$summary[2, 7]
    semedian[, j] <- fit$BUGSoutput$median$sens
    selower[, j] <- fit$BUGSoutput$summary[3:7, 3]
    seupper[, j] <- fit$BUGSoutput$summary[3:7, 7]
    spmedian[, j] <- fit$BUGSoutput$median$spec
    splower[, j] <- fit$BUGSoutput$summary[8:12, 3]
    spupper[, j] <- fit$BUGSoutput$summary[8:12, 7]
    ResD[j] <- fit$BUGSoutput$mean$sumrd
    DIC[j] <- ResD[j] + fit$BUGSoutput$pD
  }
}

rm(fit)  # Clean up

# Save full workspace
save.image(file = file.path(folder, "CPTB_LL_LL_2000.RData"))

# Report runtime
print(Sys.time() - start)
# Save everything
save.image(file = file.path(folder, "CPTB_WANG_LL_2000.RData"))
```

JAGS script to fit the L-L model with correct interactions

The fitted L-L model was the same as that used for the fixed-effect DGM for the CPTB data, as both models assumed dependence for the same test pairs without sharing interactions across tests.

JAGS script to fit the L-L model with all interactions (in the diseased group) and hyperlasso priors

```
model {

  for (i in 1:32) {

    # Likelihood for observed counts
    r[i] ~ dpois(mu[i])
```

```
    mu[i] <- p[i] * nobs

    # Mixture model for disease status
    p[i] <- (1 - prev) * p1[i] + prev * p2[i]

    # Probabilities for non-diseased and diseased groups
    p1[i] <- y1[i] / sum(y1[])
    p2[i] <- y2[i] / sum(y2[])

    # Log-linear model for non-diseased group
    log(y1[i]) <- lambda_coef[1,1]*y[i,1] + lambda_coef[1,2]*y[i,2] +
                  lambda_coef[1,3]*y[i,3] + lambda_coef[1,4]*y[i,4] +
                  lambda_coef[1,5]*y[i,5]

    # Log-linear model for diseased group
    log(y2[i]) <- lambda_coef[2,1]*y[i,1] + lambda_coef[2,2]*y[i,2] +
                  lambda_coef[2,3]*y[i,3] + lambda_coef[2,4]*y[i,4] +
                  lambda_coef[2,5]*y[i,5] +
                  lambda_int[1]*y[i,1]*y[i,2] +
                  lambda_int[2]*y[i,1]*y[i,3] +
                  lambda_int[3]*y[i,1]*y[i,4] +
                  lambda_int[4]*y[i,1]*y[i,5] +
                  lambda_int[5]*y[i,2]*y[i,3] +
                  lambda_int[6]*y[i,2]*y[i,4] +
                  lambda_int[7]*y[i,2]*y[i,5] +
                  lambda_int[8]*y[i,3]*y[i,4] +
                  lambda_int[9]*y[i,3]*y[i,5] +
                  lambda_int[10]*y[i,4]*y[i,5]

    # Deviance residual
    rd[i] <- 2 * ((mu[i] - r[i]) + r[i] * log(r[i] / mu[i]))
  }

  sumrd <- sum(rd[])

  # Prior for disease prevalence
  prev ~ dbeta(1, 1)

  # Diagnostic accuracy parameters
  for (t in 1:5) {
    sens[t] <- inprod(y[1:32, t], y2[1:32]) / sum(y2[1:32])
    fpr[t]  <- inprod(y[1:32, t], y1[1:32]) / sum(y1[1:32])
    spec[t] <- 1 - fpr[t]
    youden[t] <- sens[t] + spec[t] - 1

    AcceptTest[t] ~ dbern(ifelse(youden[t] >= 0, 1, 0))

    for (d in 1:2) {
      q[d, t] ~ dbeta(1, 1)
      lambda_coef[d, t] <- logit(q[d, t])   # Main effects via logit(q)
    }
  }

  # Hyper-LASSO priors for interaction terms
  for (t in 1:10) {
    lambda_int[t] ~ ddexp(0, prec_lambda[t])          # Double-exponential
prior
    prec_lambda[t] <- 1 / sqrt(2 * tau_jl[t])         # Precision = 1 /
sqrt(2 * tau)
    tau_jl[t] ~ dgamma(0.5, inv_theta2)               # Local shrinkage
scale
```

```
  }

  # Global shrinkage parameter
  theta ~ dt(0, 1, 1) T(0,)                          # Half-Cauchy(0,1)
  inv_theta2 <- 1 / (theta^2)

}
```

JAGS script to fit the L-L model with all interactions (in the diseased group) and elastic net priors

```
model {

  for (i in 1:32) {

    # Likelihood for observed counts
    r[i] ~ dpois(mu[i])
    mu[i] <- p[i] * nobs

    # Mixture of non-diseased and diseased probabilities
    p[i] <- (1 - prev) * p1[i] + prev * p2[i]

    # Probability of pattern i given no disease / disease
    p1[i] <- y1[i] / sum(y1[])
    p2[i] <- y2[i] / sum(y2[])

    # Log-linear model for non-diseased population
    log(y1[i]) <- lambda_coef[1,1]*y[i,1] + lambda_coef[1,2]*y[i,2] +
                  lambda_coef[1,3]*y[i,3] + lambda_coef[1,4]*y[i,4] +
                  lambda_coef[1,5]*y[i,5]

    # Log-linear model for diseased population
    log(y2[i]) <- lambda_coef[2,1]*y[i,1] + lambda_coef[2,2]*y[i,2] +
                  lambda_coef[2,3]*y[i,3] + lambda_coef[2,4]*y[i,4] +
                  lambda_coef[2,5]*y[i,5] +
                  lambda_int[1]*y[i,1]*y[i,2] +
                  lambda_int[2]*y[i,1]*y[i,3] +
                  lambda_int[3]*y[i,1]*y[i,4] +
                  lambda_int[4]*y[i,1]*y[i,5] +
                  lambda_int[5]*y[i,2]*y[i,3] +
                  lambda_int[6]*y[i,2]*y[i,4] +
                  lambda_int[7]*y[i,2]*y[i,5] +
                  lambda_int[8]*y[i,3]*y[i,4] +
                  lambda_int[9]*y[i,3]*y[i,5] +
                  lambda_int[10]*y[i,4]*y[i,5]

    # Deviance residual
    rd[i] <- 2 * ((mu[i] - r[i]) + r[i] * log(r[i] / mu[i]))
  }

  sumrd <- sum(rd[])

  # Prior for disease prevalence
  prev ~ dbeta(1, 1)

  # Main effects for diagnostic tests
  for (t in 1:5) {
    sens[t] <- inprod(y[1:32, t], y2[1:32]) / sum(y2[1:32])
    fpr[t]  <- inprod(y[1:32, t], y1[1:32]) / sum(y1[1:32])
    spec[t] <- 1 - fpr[t]
    youden[t] <- sens[t] + spec[t] - 1
```

```
    AcceptTest[t] ~ dbern(ifelse(youden[t] >= 0, 1, 0))

    for (d in 1:2) {
      q[d, t] ~ dbeta(1, 1)
      lambda_coef[d, t] <- logit(q[d, t])
    }
  }

  # Global elastic net parameters
  lambda1 ~ dt(0, 1, 1) T(0,)
  lambda2 ~ dt(0, 1, 1) T(0,)

  upper <- 8 * lambda2 / (lambda1^2 + 1e-8)

  # Shrinkage priors for interaction terms
  for (t in 1:10) {
    tau_jl[t] ~ dgamma(0.5, upper) T(1,)
    inv_var_lambda[t] <- lambda2 * tau_jl[t] / (tau_jl[t] - 1 + 1e-8)
    lambda_int[t] ~ dnorm(0, inv_var_lambda[t])
  }

}
```

JAGS script to fit the L-L model with all interactions (in the diseased group) and regularized horseshoe priors

```
model {

  for (i in 1:32) {

    # Poisson likelihood for counts in the contingency table
    r[i] ~ dpois(mu[i])
    mu[i] <- p[i] * nobs

    # Mixture model for disease status
    p[i] <- (1 - prev) * p1[i] + prev * p2[i]

    # Probabilities conditional on disease status
    p1[i] <- y1[i] / sum(y1[])
    p2[i] <- y2[i] / sum(y2[])

    # Log-linear model for non-diseased population
    log(y1[i]) <- lambda_coef[1,1]*y[i,1] + lambda_coef[1,2]*y[i,2] +
                  lambda_coef[1,3]*y[i,3] + lambda_coef[1,4]*y[i,4] +
                  lambda_coef[1,5]*y[i,5]

    # Log-linear model for diseased population
    log(y2[i]) <- lambda_coef[2,1]*y[i,1] + lambda_coef[2,2]*y[i,2] +
                  lambda_coef[2,3]*y[i,3] + lambda_coef[2,4]*y[i,4] +
                  lambda_coef[2,5]*y[i,5] +
                  lambda_int[1]*y[i,1]*y[i,2] +
                  lambda_int[2]*y[i,1]*y[i,3] +
                  lambda_int[3]*y[i,1]*y[i,4] +
                  lambda_int[4]*y[i,1]*y[i,5] +
                  lambda_int[5]*y[i,2]*y[i,3] +
                  lambda_int[6]*y[i,2]*y[i,4] +
                  lambda_int[7]*y[i,2]*y[i,5] +
                  lambda_int[8]*y[i,3]*y[i,4] +
```

```
                 lambda_int[9]*y[i,3]*y[i,5] +
                 lambda_int[10]*y[i,4]*y[i,5]

    # Deviance residuals
    rd[i] <- 2 * ((mu[i] - r[i]) + r[i] * log(r[i] / mu[i]))
  }

  sumrd <- sum(rd[])

  # Prior for disease prevalence
  prev ~ dbeta(1, 1)

  # Main effects: logit-transformed from beta priors
  for (t in 1:5) {
    sens[t] <- inprod(y[1:32, t], y2[1:32]) / sum(y2[1:32])
    fpr[t]  <- inprod(y[1:32, t], y1[1:32]) / sum(y1[1:32])
    spec[t] <- 1 - fpr[t]
    youden[t] <- sens[t] + spec[t] - 1
    AcceptTest[t] ~ dbern(ifelse(youden[t] >= 0, 1, 0))

    for (d in 1:2) {
      q[d, t] ~ dbeta(1, 1)
      lambda_coef[d, t] <- logit(q[d, t])
    }
  }

  # Regularized horseshoe priors for interaction terms
  for (t in 1:10) {
    lambda_int[t] ~ dnorm(0, prec_lambda[t])
    prec_lambda[t] <- 1 / (lambda * sigma2[t])
    sigma2[t] <- c2 * pow(delta[t], 2) / (c2 + pow(lambda, 2) *
pow(delta[t], 2))
    delta[t] ~ dt(0, 1, 1) T(0,)    # Half-Cauchy
  }

  lambda ~ dt(0, 1, 1) T(0,)        # Global shrinkage
  c2 <- 1 / invc2
  invc2 ~ dgamma(2, 8)              # Prior for inverse scale squared
}
```

CDPN Data

Latent Trait DGM

R script to simulate data and fit L-L models

```
## This R script simulates data from latent trait DGM based on the CDPN
data
## with the parameter settings specified in Appendix B and fits the
corresponding
## log-linear model with correct interactions.


# Clear the workspace
rm(list = ls())

# Load required package
```

```r
require(R2jags)

# Start timing the script
start <- Sys.time()

# Create output folder if it doesn't exist
folder <- "Chapter5-KEDDIE_LT_LL"
if(!file.exists(folder)) dir.create(folder)

# Set seed for reproducibility
set.seed(567)

# Simulation settings
nobs <- 2000                # Number of observations per dataset
ntests <- 5                 # Number of tests
nsim <- 1250                # Number of simulated datasets
n.burnin <- 10000           # Number of burn-in iterations
n.iter <- 50000             # Total number of MCMC iterations
n.thin <- 1                 # Thinning rate
n.chains <- 3               # Number of MCMC chains

# True parameter values
prev <- 0.5                 # True prevalence
se <- rep(0.65, 5)          # Sensitivity of each test
sp <- c(0.90, 0.90, 0.90, 0.90, 0.99)  # Specificity

b.RE1 <- c(1,1,1,1,0)       # Random effect scaling for diseased
b.RE2 <- c(1,1,1,1,0)       # Random effect scaling for non-diseased

# Calculate thresholds based on sensitivities and specificities
a1 <- qnorm(se) * sqrt(1 + b.RE1^2)
a2 <- qnorm(sp) * sqrt(1 + b.RE2^2)

# Initialise storage arrays
r <- d <- matrix(NA, nsim, nobs)
s <- c <- p <- y <- array(NA, dim = c(nsim, nobs, ntests))

# Simulate datasets
for(k in 1:nsim){
  for(i in 1:nobs){
    r[k, i] <- rnorm(1)                    # Latent variable
    d[k, i] <- rbinom(1, 1, prev)          # True disease status
    for(j in 1:ntests){
      s[k, i, j] <- pnorm(a1[j] + b.RE1[j] * r[k, i])
      c[k, i, j] <- pnorm(a2[j] + b.RE2[j] * r[k, i])
      p[k, i, j] <- s[k, i, j]^d[k, i] * (1 - c[k, i, j])^(1 - d[k, i])
      y[k, i, j] <- rbinom(1, 1, p[k, i, j]) # Observed test result
    }
  }
}

# Function to tabulate 5-test pattern frequencies (32 combinations)
freqtable <- function(data){
  freq <- rep(0, 32)
  pats <- apply(data, 1, function(x) paste(x, collapse = ""))
  tpats <- table(pats)
  pattern_strings <- apply(expand.grid(rep(list(c(0,1)), 5)), 1, paste,
collapse = "")
  for (i in seq_along(pattern_strings)) {
    freq[i] <- as.numeric(tpats[pattern_strings[i]])
  }
```

```r
  patterns <- cbind(expand.grid(rep(list(c(0,1)), 5)), freq)
  return(patterns)
}

# Create frequency table array for all simulations
Y <- array(NA, dim = c(nsim, 32, ntests + 1))
for(i in 1:nsim){
  Y[i,,] <- as.matrix(freqtable(y[i,,]))
}

# Parameters to monitor from JAGS
mymonitoredparamslist <- c("prev", "sens", "spec", "sumrd")

# Initialise result storage
prevmedian <- prevlower <- prevupper <- ResD <- DIC <- rep(NA, nsim)
semedian <- selower <- seupper <- spmedian <- splower <- spupper <-
matrix(NA, nrow = ntests, ncol = nsim)

# Run JAGS for each simulated dataset
for(j in 1:nsim){
  mydatalist <- list(
    r = Y[j, , ntests + 1],
    y = Y[j, , 1:ntests],
    nobs = nobs,
    AcceptTest = rep(1, ntests)
  )

  fit <- jags(
    data = mydatalist,
    parameters.to.save = mymonitoredparamslist,
    model.file = "keddie_lt_ll.txt",
    n.chains = n.chains,
    n.iter = n.iter,
    n.burnin = n.burnin,
    n.thin = n.thin,
    progress.bar = "text"
  )

  # Check convergence and extract results
  if(any(fit$BUGSoutput$summary[2:13,8] > 1.1) |
any(fit$BUGSoutput$summary[2:13,9] < 400)){
    prevmedian[j] <- prevlower[j] <- prevupper[j] <- ResD[j] <- DIC[j] <-
NA
    semedian[,j] <- selower[,j] <- seupper[,j] <- spmedian[,j] <-
splower[,j] <- spupper[,j] <- NA
  } else {
    prevmedian[j] <- fit$BUGSoutput$median$prev
    prevlower[j]  <- fit$BUGSoutput$summary[2, 3]
    prevupper[j]  <- fit$BUGSoutput$summary[2, 7]
    semedian[,j]  <- fit$BUGSoutput$median$sens
    selower[,j]   <- fit$BUGSoutput$summary[3:7, 3]
    seupper[,j]   <- fit$BUGSoutput$summary[3:7, 7]
    spmedian[,j]  <- fit$BUGSoutput$median$spec
    splower[,j]   <- fit$BUGSoutput$summary[8:12, 3]
    spupper[,j]   <- fit$BUGSoutput$summary[8:12, 7]
    ResD[j]       <- fit$BUGSoutput$mean$sumrd
    DIC[j]        <- ResD[j] + fit$BUGSoutput$pD
  }
}

# Save workspace
```

```
save.image(file = file.path(folder, "KEDDIE_LT_LL_2000.RData"))

# Print runtime
print(Sys.time() - start)
```

JAGS script to fit the L-L model with correct interactions

```
model {

  for (i in 1:32) {

    r[i] ~ dpois(mu[i])                          # Likelihood: Poisson
counts for each pattern
    mu[i] <- p[i] * nobs                         # Expected count =
probability × sample size

    p[i] <- (1 - prev) * p1[i] + prev * p2[i]    # Mixture probability

    p1[i] <- y1[i] / sum(y1[])                   # Pr(pattern i | non-
diseased)
    p2[i] <- y2[i] / sum(y2[])                   # Pr(pattern i | diseased)

    # Log-linear model for non-diseased group
    log(y1[i]) <- lambda_coef[1,1]*y[i,1] + lambda_coef[1,2]*y[i,2] +
                  lambda_coef[1,3]*y[i,3] + lambda_coef[1,4]*y[i,4] +
lambda_coef[1,5]*y[i,5] +
                  lambda_int * ( y[i,1]*y[i,2] + y[i,1]*y[i,3] +
y[i,1]*y[i,4] +
                                 y[i,2]*y[i,3] + y[i,2]*y[i,4] +
y[i,3]*y[i,4] )

    # Log-linear model for diseased group (sharing interaction effects with
non-diseased)
    log(y2[i]) <- lambda_coef[2,1]*y[i,1] + lambda_coef[2,2]*y[i,2] +
                  lambda_coef[2,3]*y[i,3] + lambda_coef[2,4]*y[i,4] +
lambda_coef[2,5]*y[i,5] +
                  lambda_int * ( y[i,1]*y[i,2] + y[i,1]*y[i,3] +
y[i,1]*y[i,4] +
                                 y[i,2]*y[i,3] + y[i,2]*y[i,4] +
y[i,3]*y[i,4] )

    rd[i] <- 2 * ((mu[i] - r[i]) + r[i] * log(r[i] / mu[i]))  # Deviance
residual
  }

  sumrd <- sum(rd[])                              # Total deviance

  # Prior for prevalence
  prev ~ dbeta(1, 1)

  # Priors for main effects and test accuracy calculations
  for (t in 1:5) {

    sens[t] <- inprod(y[1:32, t], y2[1:32]) / sum(y2[1:32])    #
Sensitivity
    fpr[t]  <- inprod(y[1:32, t], y1[1:32]) / sum(y1[1:32])    # False
positive rate
    spec[t] <- 1 - fpr[t]                                      #
Specificity
```

```
    youden[t] <- sens[t] + spec[t] - 1                             # Youden
index
    AcceptTest[t] ~ dbern(ifelse(youden[t] >= 0, 1, 0))            # Accept if
Youden ≥ 0

    for (d in 1:2) {
      q[d, t] ~ dbeta(1, 1)
      lambda_coef[d, t] <- logit(q[d, t])                          # Main
effects: logit scale
    }
  }

  # Shared interaction parameter across both groups
  lambda_int ~ dnorm(0, 1)                                         # Prior for
interaction term
}
```

Fixed-Effect DGM

R script to simulate data and fit L-L models

```
## This R script simulates data from fixed-effect DGM based on the CDPN
data
## with the parameter settings specified in Appendix B and fits the
corresponding
## log-linear model with correct interactions.


rm(list = ls())  # Clear all existing objects

require(R2jags)  # Load JAGS interface for R

start <- Sys.time()  # Track computation time

# Create results folder if not already existing
folder <- "Chapter5-KEDDIE_JJHC_LL_SC_NCS_DI"
if (!file.exists(folder)) dir.create(folder)

set.seed(567)  # Set reproducibility seed

# ---- Simulation Settings ----
nobs <- 2000              # Number of observations per dataset
ntests <- 5               # Number of tests
nsim <- 1250              # Number of simulated datasets
n.burnin <- 10000         # Burn-in iterations
n.iter <- 50000           # Total MCMC iterations
n.thin <- 1               # Thinning interval
n.chains <- 3             # Number of MCMC chains

# ---- Disease Model Settings ----
prev <- 0.5                                   # Disease prevalence
se <- c(0.65, 0.65, 0.65, 0.65, 0.65)         # Sensitivities
sp <- c(0.9, 0.9, 0.9, 0.9, 0.99)            # Specificities


covsp12 = 0.015; covsp13 = 0.015; covsp14 = 0.015
covsp15 = 0;     covsp23 = 0.015; covsp24 = 0.015
covsp25 = 0;     covsp34 = 0.015; covsp35 = 0
```

```
covsp45 = 0


covse12 = 0.075; covse13 = 0.075; covse14 = 0.075
covse15 = 0;     covse23 = 0.075; covse24 = 0.075
covse25 = 0;     covse34 = 0.075; covse35 = 0
covse45 = 0


p0 <- rep(NA, 2^ntests)
p1 <- rep(NA, 2^ntests)

# Probability of observing Test1- Test2- Test3- Test4- Test5- from a true
positive:
p1[1] <- prev * (
  (1 - se[1]) * (1 - se[2]) * (1 - se[3]) * (1 - se[4]) * (1 - se[5])
  + covse12 * (1 - se[3]) * (1 - se[4]) * (1 - se[5])
  + covse13 * (1 - se[2]) * (1 - se[4]) * (1 - se[5])
  + covse14 * (1 - se[2]) * (1 - se[3]) * (1 - se[5])
  + covse15 * (1 - se[2]) * (1 - se[3]) * (1 - se[4])
  + covse23 * (1 - se[1]) * (1 - se[4]) * (1 - se[5])
  + covse24 * (1 - se[1]) * (1 - se[3]) * (1 - se[5])
  + covse25 * (1 - se[1]) * (1 - se[3]) * (1 - se[4])
  + covse34 * (1 - se[1]) * (1 - se[2]) * (1 - se[5])
  + covse35 * (1 - se[1]) * (1 - se[2]) * (1 - se[4])
  + covse45 * (1 - se[1]) * (1 - se[2]) * (1 - se[3])
)

# Probability of observing Test1- Test2- Test3- Test4- Test5- from a true
negative:
p0[1] <- (1 - prev) * (
  sp[1] * sp[2] * sp[3] * sp[4] * sp[5]
  + covsp12 * sp[3] * sp[4] * sp[5]
  + covsp13 * sp[2] * sp[4] * sp[5]
  + covsp14 * sp[2] * sp[3] * sp[5]
  + covsp15 * sp[2] * sp[3] * sp[4]
  + covsp23 * sp[1] * sp[4] * sp[5]
  + covsp24 * sp[1] * sp[3] * sp[5]
  + covsp25 * sp[1] * sp[3] * sp[4]
  + covsp34 * sp[1] * sp[2] * sp[5]
  + covsp35 * sp[1] * sp[2] * sp[4]
  + covsp45 * sp[1] * sp[2] * sp[3]
)
# Probability of observing Test1+ Test2- Test3- Test4- Test5- from a true
positive:
p1[2] <- prev * (
  se[1] * (1 - se[2]) * (1 - se[3]) * (1 - se[4]) * (1 - se[5])
  - covse12 * (1 - se[3]) * (1 - se[4]) * (1 - se[5])
  - covse13 * (1 - se[2]) * (1 - se[4]) * (1 - se[5])
  - covse14 * (1 - se[2]) * (1 - se[3]) * (1 - se[5])
  - covse15 * (1 - se[2]) * (1 - se[3]) * (1 - se[4])
  + covse23 * se[1] * (1 - se[4]) * (1 - se[5])
  + covse24 * se[1] * (1 - se[3]) * (1 - se[5])
  + covse25 * se[1] * (1 - se[3]) * (1 - se[4])
  + covse34 * se[1] * (1 - se[2]) * (1 - se[5])
  + covse35 * se[1] * (1 - se[2]) * (1 - se[4])
  + covse45 * se[1] * (1 - se[2]) * (1 - se[3])
)

# Probability of observing Test1+ Test2- Test3- Test4- Test5- from a true
negative:
```

```
p0[2] <- (1 - prev) * (
  (1 - sp[1]) * sp[2] * sp[3] * sp[4] * sp[5]
  - covsp12 * sp[3] * sp[4] * sp[5]
  - covsp13 * sp[2] * sp[4] * sp[5]
  - covsp14 * sp[2] * sp[3] * sp[5]
  - covsp15 * sp[2] * sp[3] * sp[4]
  + covsp23 * (1 - sp[1]) * sp[4] * sp[5]
  + covsp24 * (1 - sp[1]) * sp[3] * sp[5]
  + covsp25 * (1 - sp[1]) * sp[3] * sp[4]
  + covsp34 * (1 - sp[1]) * sp[2] * sp[5]
  + covsp35 * (1 - sp[1]) * sp[2] * sp[4]
  + covsp45 * (1 - sp[1]) * sp[2] * sp[3]
)
# Probability of observing Test1- Test2+ Test3- Test4- Test5- from a true
positive:
p1[3] <- prev * (
  (1 - se[1]) * se[2] * (1 - se[3]) * (1 - se[4]) * (1 - se[5])
  - covse12 * (1 - se[3]) * (1 - se[4]) * (1 - se[5])
  + covse13 * se[2] * (1 - se[4]) * (1 - se[5])
  + covse14 * se[2] * (1 - se[3]) * (1 - se[5])
  + covse15 * se[2] * (1 - se[3]) * (1 - se[4])
  - covse23 * (1 - se[1]) * (1 - se[4]) * (1 - se[5])
  - covse24 * (1 - se[1]) * (1 - se[3]) * (1 - se[5])
  - covse25 * (1 - se[1]) * (1 - se[3]) * (1 - se[4])
  + covse34 * (1 - se[1]) * se[2] * (1 - se[5])
  + covse35 * (1 - se[1]) * se[2] * (1 - se[4])
  + covse45 * (1 - se[1]) * se[2] * (1 - se[3])
)

# Probability of observing Test1- Test2+ Test3- Test4- Test5- from a true
negative:
p0[3] <- (1 - prev) * (
  sp[1] * (1 - sp[2]) * sp[3] * sp[4] * sp[5]
  - covsp12 * sp[3] * sp[4] * sp[5]
  + covsp13 * (1 - sp[2]) * sp[4] * sp[5]
  + covsp14 * (1 - sp[2]) * sp[3] * sp[5]
  + covsp15 * (1 - sp[2]) * sp[3] * sp[4]
  - covsp23 * sp[1] * sp[4] * sp[5]
  - covsp24 * sp[1] * sp[3] * sp[5]
  - covsp25 * sp[1] * sp[3] * sp[4]
  + covsp34 * sp[1] * (1 - sp[2]) * sp[5]
  + covsp35 * sp[1] * (1 - sp[2]) * sp[4]
  + covsp45 * sp[1] * (1 - sp[2]) * sp[3]
)
# Probability of observing Test1+ Test2+ Test3- Test4- Test5- from a true
positive:
p1[4] <- prev * (
  se[1] * se[2] * (1 - se[3]) * (1 - se[4]) * (1 - se[5])
  + covse12 * (1 - se[3]) * (1 - se[4]) * (1 - se[5])
  - covse13 * se[2] * (1 - se[4]) * (1 - se[5])
  - covse14 * se[2] * (1 - se[3]) * (1 - se[5])
  - covse15 * se[2] * (1 - se[3]) * (1 - se[4])
  - covse23 * se[1] * (1 - se[4]) * (1 - se[5])
  - covse24 * se[1] * (1 - se[3]) * (1 - se[5])
  - covse25 * se[1] * (1 - se[3]) * (1 - se[4])
  + covse34 * se[1] * se[2] * (1 - se[5])
  + covse35 * se[1] * se[2] * (1 - se[4])
  + covse45 * se[1] * se[2] * (1 - se[3])
)
```

```
# Probability of observing Test1+ Test2+ Test3- Test4- Test5- from a true
negative:
p0[4] <- (1 - prev) * (
   (1 - sp[1]) * (1 - sp[2]) * sp[3] * sp[4] * sp[5]
   + covsp12 * sp[3] * sp[4] * sp[5]
   - covsp13 * (1 - sp[2]) * sp[4] * sp[5]
   - covsp14 * (1 - sp[2]) * sp[3] * sp[5]
   - covsp15 * (1 - sp[2]) * sp[3] * sp[4]
   - covsp23 * (1 - sp[1]) * sp[4] * sp[5]
   - covsp24 * (1 - sp[1]) * sp[3] * sp[5]
   - covsp25 * (1 - sp[1]) * sp[3] * sp[4]
   + covsp34 * (1 - sp[1]) * (1 - sp[2]) * sp[5]
   + covsp35 * (1 - sp[1]) * (1 - sp[2]) * sp[4]
   + covsp45 * (1 - sp[1]) * (1 - sp[2]) * sp[3]
)
# Probability of observing Test1- Test2- Test3+ Test4- Test5- from a true
positive:
p1[5] <- prev * (
   (1 - se[1]) * (1 - se[2]) * se[3] * (1 - se[4]) * (1 - se[5])
   + covse12 * se[3] * (1 - se[4]) * (1 - se[5])
   - covse13 * (1 - se[2]) * (1 - se[4]) * (1 - se[5])
   + covse14 * (1 - se[2]) * se[3] * (1 - se[5])
   + covse15 * (1 - se[2]) * se[3] * (1 - se[4])
   - covse23 * (1 - se[1]) * (1 - se[4]) * (1 - se[5])
   + covse24 * (1 - se[1]) * se[3] * (1 - se[5])
   + covse25 * (1 - se[1]) * se[3] * (1 - se[4])
   - covse34 * (1 - se[1]) * (1 - se[2]) * (1 - se[5])
   - covse35 * (1 - se[1]) * (1 - se[2]) * (1 - se[4])
   + covse45 * (1 - se[1]) * (1 - se[2]) * se[3]
)

# Probability of observing Test1- Test2- Test3+ Test4- Test5- from a true
negative:
p0[5] <- (1 - prev) * (
   sp[1] * sp[2] * (1 - sp[3]) * sp[4] * sp[5]
   + covsp12 * (1 - sp[3]) * sp[4] * sp[5]
   - covsp13 * sp[2] * sp[4] * sp[5]
   + covsp14 * sp[2] * (1 - sp[3]) * sp[5]
   + covsp15 * sp[2] * (1 - sp[3]) * sp[4]
   - covsp23 * sp[1] * sp[4] * sp[5]
   + covsp24 * sp[1] * (1 - sp[3]) * sp[5]
   + covsp25 * sp[1] * (1 - sp[3]) * sp[4]
   - covsp34 * sp[1] * sp[2] * sp[5]
   - covsp35 * sp[1] * sp[2] * sp[4]
   + covsp45 * sp[1] * sp[2] * (1 - sp[3])
)
# Probability of observing Test1+ Test2- Test3+ Test4- Test5- from a true
positive:
p1[6] <- prev * (
   se[1] * (1 - se[2]) * se[3] * (1 - se[4]) * (1 - se[5])
   - covse12 * se[3] * (1 - se[4]) * (1 - se[5])
   + covse13 * (1 - se[2]) * (1 - se[4]) * (1 - se[5])
   - covse14 * (1 - se[2]) * se[3] * (1 - se[5])
   - covse15 * (1 - se[2]) * se[3] * (1 - se[4])
   - covse23 * se[1] * (1 - se[4]) * (1 - se[5])
   + covse24 * se[1] * se[3] * (1 - se[5])
   + covse25 * se[1] * se[3] * (1 - se[4])
   - covse34 * se[1] * (1 - se[2]) * (1 - se[5])
   - covse35 * se[1] * (1 - se[2]) * (1 - se[4])
   + covse45 * se[1] * (1 - se[2]) * se[3]
)
```

```
# Probability of observing Test1+ Test2- Test3+ Test4- Test5- from a true
negative:
p0[6] <- (1 - prev) * (
  (1 - sp[1]) * sp[2] * (1 - sp[3]) * sp[4] * sp[5]
  - covsp12 * (1 - sp[3]) * sp[4] * sp[5]
  + covsp13 * sp[2] * sp[4] * sp[5]
  - covsp14 * sp[2] * (1 - sp[3]) * sp[5]
  - covsp15 * sp[2] * (1 - sp[3]) * sp[4]
  - covsp23 * (1 - sp[1]) * sp[4] * sp[5]
  + covsp24 * (1 - sp[1]) * (1 - sp[3]) * sp[5]
  + covsp25 * (1 - sp[1]) * (1 - sp[3]) * sp[4]
  - covsp34 * (1 - sp[1]) * sp[2] * sp[5]
  - covsp35 * (1 - sp[1]) * sp[2] * sp[4]
  + covsp45 * (1 - sp[1]) * sp[2] * (1 - sp[3])
)
# Probability of observing Test1- Test2+ Test3+ Test4- Test5- from a true
positive:
p1[7] <- prev * (
  (1 - se[1]) * se[2] * se[3] * (1 - se[4]) * (1 - se[5])
  - covse12 * se[3] * (1 - se[4]) * (1 - se[5])
  - covse13 * se[2] * (1 - se[4]) * (1 - se[5])
  + covse14 * se[2] * se[3] * (1 - se[5])
  + covse15 * se[2] * se[3] * (1 - se[4])
  + covse23 * (1 - se[1]) * (1 - se[4]) * (1 - se[5])
  - covse24 * (1 - se[1]) * se[3] * (1 - se[5])
  - covse25 * (1 - se[1]) * se[3] * (1 - se[4])
  - covse34 * (1 - se[1]) * se[2] * (1 - se[5])
  - covse35 * (1 - se[1]) * se[2] * (1 - se[4])
  + covse45 * (1 - se[1]) * se[2] * se[3]
)

# Probability of observing Test1- Test2+ Test3+ Test4- Test5- from a true
negative:
p0[7] <- (1 - prev) * (
  sp[1] * (1 - sp[2]) * (1 - sp[3]) * sp[4] * sp[5]
  - covsp12 * (1 - sp[3]) * sp[4] * sp[5]
  - covsp13 * (1 - sp[2]) * sp[4] * sp[5]
  + covsp14 * (1 - sp[2]) * (1 - sp[3]) * sp[5]
  + covsp15 * (1 - sp[2]) * (1 - sp[3]) * sp[4]
  + covsp23 * sp[1] * sp[4] * sp[5]
  - covsp24 * sp[1] * (1 - sp[3]) * sp[5]
  - covsp25 * sp[1] * (1 - sp[3]) * sp[4]
  - covsp34 * sp[1] * (1 - sp[2]) * sp[5]
  - covsp35 * sp[1] * (1 - sp[2]) * sp[4]
  + covsp45 * sp[1] * (1 - sp[2]) * (1 - sp[3])
)
# Probability of observing Test1+ Test2+ Test3+ Test4- Test5- from a true
positive:
p1[8] <- prev * (
  se[1] * se[2] * se[3] * (1 - se[4]) * (1 - se[5])
  + covse12 * se[3] * (1 - se[4]) * (1 - se[5])
  + covse13 * se[2] * (1 - se[4]) * (1 - se[5])
  - covse14 * se[2] * se[3] * (1 - se[5])
  - covse15 * se[2] * se[3] * (1 - se[4])
  + covse23 * se[1] * (1 - se[4]) * (1 - se[5])
  - covse24 * se[1] * se[3] * (1 - se[5])
  - covse25 * se[1] * se[3] * (1 - se[4])
  - covse34 * se[1] * se[2] * (1 - se[5])
  - covse35 * se[1] * se[2] * (1 - se[4])
  + covse45 * se[1] * se[2] * se[3]
```

```
)

# Probability of observing Test1+ Test2+ Test3+ Test4- Test5- from a true
negative:
p0[8] <- (1 - prev) * (
  (1 - sp[1]) * (1 - sp[2]) * (1 - sp[3]) * sp[4] * sp[5]
  + covsp12 * (1 - sp[3]) * sp[4] * sp[5]
  + covsp13 * (1 - sp[2]) * sp[4] * sp[5]
  - covsp14 * (1 - sp[2]) * (1 - sp[3]) * sp[5]
  - covsp15 * (1 - sp[2]) * (1 - sp[3]) * sp[4]
  + covsp23 * (1 - sp[1]) * sp[4] * sp[5]
  - covsp24 * (1 - sp[1]) * (1 - sp[3]) * sp[5]
  - covsp25 * (1 - sp[1]) * (1 - sp[3]) * sp[4]
  - covsp34 * (1 - sp[1]) * (1 - sp[2]) * sp[5]
  - covsp35 * (1 - sp[1]) * (1 - sp[2]) * sp[4]
  + covsp45 * (1 - sp[1]) * (1 - sp[2]) * (1 - sp[3])
)
# Probability of observing Test1- Test2- Test3- Test4+ Test5- from a true
positive:
p1[9] <- prev * (
  (1 - se[1]) * (1 - se[2]) * (1 - se[3]) * se[4] * (1 - se[5])
  + covse12 * (1 - se[3]) * se[4] * (1 - se[5])
  + covse13 * (1 - se[2]) * se[4] * (1 - se[5])
  - covse14 * (1 - se[2]) * (1 - se[3]) * (1 - se[5])
  + covse15 * (1 - se[2]) * (1 - se[3]) * se[4]
  + covse23 * (1 - se[1]) * se[4] * (1 - se[5])
  - covse24 * (1 - se[1]) * (1 - se[3]) * (1 - se[5])
  + covse25 * (1 - se[1]) * (1 - se[3]) * se[4]
  - covse34 * (1 - se[1]) * (1 - se[2]) * (1 - se[5])
  + covse35 * (1 - se[1]) * (1 - se[2]) * se[4]
  - covse45 * (1 - se[1]) * (1 - se[2]) * (1 - se[3])
)

# Probability of observing Test1- Test2- Test3- Test4+ Test5- from a true
negative:
p0[9] <- (1 - prev) * (
  sp[1] * sp[2] * sp[3] * (1 - sp[4]) * sp[5]
  + covsp12 * sp[3] * (1 - sp[4]) * sp[5]
  + covsp13 * sp[2] * (1 - sp[4]) * sp[5]
  - covsp14 * sp[2] * sp[3] * sp[5]
  + covsp15 * sp[2] * sp[3] * (1 - sp[4])
  + covsp23 * sp[1] * (1 - sp[4]) * sp[5]
  - covsp24 * sp[1] * sp[3] * sp[5]
  + covsp25 * sp[1] * sp[3] * (1 - sp[4])
  - covsp34 * sp[1] * sp[2] * sp[5]
  + covsp35 * sp[1] * sp[2] * (1 - sp[4])
  - covsp45 * sp[1] * sp[2] * sp[3]
)
# Probability of observing Test1+ Test2- Test3- Test4+ Test5- from a true
positive:
p1[10] <- prev * (
  se[1] * (1 - se[2]) * (1 - se[3]) * se[4] * (1 - se[5])
  - covse12 * (1 - se[3]) * se[4] * (1 - se[5])
  - covse13 * (1 - se[2]) * se[4] * (1 - se[5])
  + covse14 * (1 - se[2]) * (1 - se[3]) * (1 - se[5])
  - covse15 * (1 - se[2]) * (1 - se[3]) * se[4]
  + covse23 * se[1] * se[4] * (1 - se[5])
  - covse24 * se[1] * (1 - se[3]) * (1 - se[5])
  + covse25 * se[1] * (1 - se[3]) * se[4]
  - covse34 * se[1] * (1 - se[2]) * (1 - se[5])
  + covse35 * se[1] * (1 - se[2]) * se[4]
```

```
  - covse45 * se[1] * (1 - se[2]) * (1 - se[3])
)

# Probability of observing Test1+ Test2- Test3- Test4+ Test5- from a true
negative:
p0[10] <- (1 - prev) * (
  (1 - sp[1]) * sp[2] * sp[3] * (1 - sp[4]) * sp[5]
  - covsp12 * sp[3] * (1 - sp[4]) * sp[5]
  - covsp13 * sp[2] * (1 - sp[4]) * sp[5]
  + covsp14 * sp[2] * sp[3] * sp[5]
  - covsp15 * sp[2] * sp[3] * (1 - sp[4])
  + covsp23 * (1 - sp[1]) * (1 - sp[4]) * sp[5]
  - covsp24 * (1 - sp[1]) * sp[3] * sp[5]
  + covsp25 * (1 - sp[1]) * sp[3] * (1 - sp[4])
  - covsp34 * (1 - sp[1]) * sp[2] * sp[5]
  + covsp35 * (1 - sp[1]) * sp[2] * (1 - sp[4])
  - covsp45 * (1 - sp[1]) * sp[2] * sp[3]
)
# Probability of observing Test1- Test2+ Test3- Test4+ Test5- from a true
positive:
p1[11] <- prev * (
  (1 - se[1]) * se[2] * (1 - se[3]) * se[4] * (1 - se[5])
  - covse12 * (1 - se[3]) * se[4] * (1 - se[5])
  + covse13 * se[2] * se[4] * (1 - se[5])
  - covse14 * se[2] * (1 - se[3]) * (1 - se[5])
  + covse15 * se[2] * (1 - se[3]) * se[4]
  - covse23 * (1 - se[1]) * se[4] * (1 - se[5])
  + covse24 * (1 - se[1]) * (1 - se[3]) * (1 - se[5])
  - covse25 * (1 - se[1]) * (1 - se[3]) * se[4]
  - covse34 * (1 - se[1]) * se[2] * (1 - se[5])
  + covse35 * (1 - se[1]) * se[2] * se[4]
  - covse45 * (1 - se[1]) * se[2] * (1 - se[3])
)

# Probability of observing Test1- Test2+ Test3- Test4+ Test5- from a true
negative:
p0[11] <- (1 - prev) * (
  sp[1] * (1 - sp[2]) * sp[3] * (1 - sp[4]) * sp[5]
  - covsp12 * sp[3] * (1 - sp[4]) * sp[5]
  + covsp13 * (1 - sp[2]) * (1 - sp[4]) * sp[5]
  - covsp14 * (1 - sp[2]) * sp[3] * sp[5]
  + covsp15 * (1 - sp[2]) * sp[3] * (1 - sp[4])
  - covsp23 * sp[1] * (1 - sp[4]) * sp[5]
  + covsp24 * sp[1] * sp[3] * sp[5]
  - covsp25 * sp[1] * sp[3] * (1 - sp[4])
  - covsp34 * sp[1] * (1 - sp[2]) * sp[5]
  + covsp35 * sp[1] * (1 - sp[2]) * (1 - sp[4])
  - covsp45 * sp[1] * (1 - sp[2]) * sp[3]
)
# Probability of observing Test1+ Test2+ Test3- Test4+ Test5- from a true
positive:
p1[12] <- prev * (
  se[1] * se[2] * (1 - se[3]) * se[4] * (1 - se[5])
  + covse12 * (1 - se[3]) * se[4] * (1 - se[5])
  - covse13 * se[2] * se[4] * (1 - se[5])
  + covse14 * se[2] * (1 - se[3]) * (1 - se[5])
  - covse15 * se[2] * (1 - se[3]) * se[4]
  - covse23 * se[1] * se[4] * (1 - se[5])
  + covse24 * se[1] * (1 - se[3]) * (1 - se[5])
  - covse25 * se[1] * (1 - se[3]) * se[4]
  - covse34 * se[1] * se[2] * (1 - se[5])
```

```
  + covse35 * se[1] * se[2] * se[4]
  - covse45 * se[1] * se[2] * (1 - se[3])
)

# Probability of observing Test1+ Test2+ Test3- Test4+ Test5- from a true
negative:
p0[12] <- (1 - prev) * (
   (1 - sp[1]) * (1 - sp[2]) * sp[3] * (1 - sp[4]) * sp[5]
  + covsp12 * sp[3] * (1 - sp[4]) * sp[5]
  - covsp13 * (1 - sp[2]) * (1 - sp[4]) * sp[5]
  + covsp14 * (1 - sp[2]) * sp[3] * sp[5]
  - covsp15 * (1 - sp[2]) * sp[3] * (1 - sp[4])
  - covsp23 * (1 - sp[1]) * (1 - sp[4]) * sp[5]
  + covsp24 * (1 - sp[1]) * sp[3] * sp[5]
  - covsp25 * (1 - sp[1]) * sp[3] * (1 - sp[4])
  - covsp34 * (1 - sp[1]) * (1 - sp[2]) * sp[5]
  + covsp35 * (1 - sp[1]) * (1 - sp[2]) * (1 - sp[4])
  - covsp45 * (1 - sp[1]) * (1 - sp[2]) * sp[3]
)
# Probability of observing Test1- Test2- Test3+ Test4+ Test5- from a true
positive:
p1[13] <- prev * (
   (1 - se[1]) * (1 - se[2]) * se[3] * se[4] * (1 - se[5])
  + covse12 * se[3] * se[4] * (1 - se[5])
  - covse13 * (1 - se[2]) * se[4] * (1 - se[5])
  - covse14 * (1 - se[2]) * se[3] * (1 - se[5])
  + covse15 * (1 - se[2]) * se[3] * se[4]
  - covse23 * (1 - se[1]) * se[4] * (1 - se[5])
  - covse24 * (1 - se[1]) * se[3] * (1 - se[5])
  + covse25 * (1 - se[1]) * se[3] * se[4]
  + covse34 * (1 - se[1]) * (1 - se[2]) * (1 - se[5])
  - covse35 * (1 - se[1]) * (1 - se[2]) * se[4]
  - covse45 * (1 - se[1]) * (1 - se[2]) * se[3]
)

# Probability of observing Test1- Test2- Test3+ Test4+ Test5- from a true
negative:
p0[13] <- (1 - prev) * (
  sp[1] * sp[2] * (1 - sp[3]) * (1 - sp[4]) * sp[5]
  + covsp12 * (1 - sp[3]) * (1 - sp[4]) * sp[5]
  - covsp13 * sp[2] * (1 - sp[4]) * sp[5]
  - covsp14 * sp[2] * (1 - sp[3]) * sp[5]
  + covsp15 * sp[2] * (1 - sp[3]) * (1 - sp[4])
  - covsp23 * sp[1] * (1 - sp[4]) * sp[5]
  - covsp24 * sp[1] * (1 - sp[3]) * sp[5]
  + covsp25 * sp[1] * (1 - sp[3]) * (1 - sp[4])
  + covsp34 * sp[1] * sp[2] * sp[5]
  - covsp35 * sp[1] * sp[2] * (1 - sp[4])
  - covsp45 * sp[1] * sp[2] * (1 - sp[3])
)
# Probability of observing Test1+ Test2- Test3+ Test4+ Test5- from a true
positive:
p1[14] <- prev * (
  se[1] * (1 - se[2]) * se[3] * se[4] * (1 - se[5])
  - covse12 * se[3] * se[4] * (1 - se[5])
  + covse13 * (1 - se[2]) * se[4] * (1 - se[5])
  + covse14 * (1 - se[2]) * se[3] * (1 - se[5])
  - covse15 * (1 - se[2]) * se[3] * se[4]
  - covse23 * se[1] * se[4] * (1 - se[5])
  - covse24 * se[1] * se[3] * (1 - se[5])
  + covse25 * se[1] * se[3] * se[4]
```

```
  + covse34 * se[1] * (1 - se[2]) * (1 - se[5])
  - covse35 * se[1] * (1 - se[2]) * se[4]
  - covse45 * se[1] * (1 - se[2]) * se[3]
)

# Probability of observing Test1+ Test2- Test3+ Test4+ Test5- from a true
negative:
p0[14] <- (1 - prev) * (
  (1 - sp[1]) * sp[2] * (1 - sp[3]) * (1 - sp[4]) * sp[5]
  - covsp12 * (1 - sp[3]) * (1 - sp[4]) * sp[5]
  + covsp13 * sp[2] * (1 - sp[4]) * sp[5]
  + covsp14 * sp[2] * (1 - sp[3]) * sp[5]
  - covsp15 * sp[2] * (1 - sp[3]) * (1 - sp[4])
  - covsp23 * (1 - sp[1]) * (1 - sp[4]) * sp[5]
  - covsp24 * (1 - sp[1]) * (1 - sp[3]) * sp[5]
  + covsp25 * (1 - sp[1]) * (1 - sp[3]) * (1 - sp[4])
  + covsp34 * (1 - sp[1]) * sp[2] * sp[5]
  - covsp35 * (1 - sp[1]) * sp[2] * (1 - sp[4])
  - covsp45 * (1 - sp[1]) * sp[2] * (1 - sp[3])
)
# Probability of observing Test1- Test2+ Test3+ Test4+ Test5- from a true
positive:
p1[15] <- prev * (
  (1 - se[1]) * se[2] * se[3] * se[4] * (1 - se[5])
  - covse12 * se[3] * se[4] * (1 - se[5])
  - covse13 * se[2] * se[4] * (1 - se[5])
  - covse14 * se[2] * se[3] * (1 - se[5])
  + covse15 * se[2] * se[3] * se[4]
  + covse23 * (1 - se[1]) * se[4] * (1 - se[5])
  + covse24 * (1 - se[1]) * se[3] * (1 - se[5])
  - covse25 * (1 - se[1]) * se[3] * se[4]
  + covse34 * (1 - se[1]) * se[2] * (1 - se[5])
  - covse35 * (1 - se[1]) * se[2] * se[4]
  - covse45 * (1 - se[1]) * se[2] * se[3]
)

# Probability of observing Test1- Test2+ Test3+ Test4+ Test5- from a true
negative:
p0[15] <- (1 - prev) * (
  sp[1] * (1 - sp[2]) * (1 - sp[3]) * (1 - sp[4]) * sp[5]
  - covsp12 * (1 - sp[3]) * (1 - sp[4]) * sp[5]
  - covsp13 * (1 - sp[2]) * (1 - sp[4]) * sp[5]
  - covsp14 * (1 - sp[2]) * (1 - sp[3]) * sp[5]
  + covsp15 * (1 - sp[2]) * (1 - sp[3]) * (1 - sp[4])
  + covsp23 * sp[1] * (1 - sp[4]) * sp[5]
  + covsp24 * sp[1] * (1 - sp[3]) * sp[5]
  - covsp25 * sp[1] * (1 - sp[3]) * (1 - sp[4])
  + covsp34 * sp[1] * (1 - sp[2]) * sp[5]
  - covsp35 * sp[1] * (1 - sp[2]) * (1 - sp[4])
  - covsp45 * sp[1] * (1 - sp[2]) * (1 - sp[3])
)
# Probability of observing Test1+ Test2+ Test3+ Test4+ Test5- from a true
positive:
p1[16] <- prev * (
  se[1] * se[2] * se[3] * se[4] * (1 - se[5])
  + covse12 * se[3] * se[4] * (1 - se[5])
  + covse13 * se[2] * se[4] * (1 - se[5])
  + covse14 * se[2] * se[3] * (1 - se[5])
  - covse15 * se[2] * se[3] * se[4]
  + covse23 * se[1] * se[4] * (1 - se[5])
  + covse24 * se[1] * se[3] * (1 - se[5])
```

```
  - covse25 * se[1] * se[3] * se[4]
  + covse34 * se[1] * se[2] * (1 - se[5])
  - covse35 * se[1] * se[2] * se[4]
  - covse45 * se[1] * se[2] * se[3]
)

# Probability of observing Test1+ Test2+ Test3+ Test4+ Test5- from a true
negative:
p0[16] <- (1 - prev) * (
  (1 - sp[1]) * (1 - sp[2]) * (1 - sp[3]) * (1 - sp[4]) * sp[5]
  + covsp12 * (1 - sp[3]) * (1 - sp[4]) * sp[5]
  + covsp13 * (1 - sp[2]) * (1 - sp[4]) * sp[5]
  + covsp14 * (1 - sp[2]) * (1 - sp[3]) * sp[5]
  - covsp15 * (1 - sp[2]) * (1 - sp[3]) * (1 - sp[4])
  + covsp23 * (1 - sp[1]) * (1 - sp[4]) * sp[5]
  + covsp24 * (1 - sp[1]) * (1 - sp[3]) * sp[5]
  - covsp25 * (1 - sp[1]) * (1 - sp[3]) * (1 - sp[4])
  + covsp34 * (1 - sp[1]) * (1 - sp[2]) * sp[5]
  - covsp35 * (1 - sp[1]) * (1 - sp[2]) * (1 - sp[4])
  - covsp45 * (1 - sp[1]) * (1 - sp[2]) * (1 - sp[3])
)
# Probability of observing Test1- Test2- Test3- Test4- Test5+ from a true
positive:
p1[17] <- prev * (
  (1 - se[1]) * (1 - se[2]) * (1 - se[3]) * (1 - se[4]) * se[5]
  + covse12 * (1 - se[3]) * (1 - se[4]) * se[5]
  + covse13 * (1 - se[2]) * (1 - se[4]) * se[5]
  + covse14 * (1 - se[2]) * (1 - se[3]) * se[5]
  - covse15 * (1 - se[2]) * (1 - se[3]) * (1 - se[4])
  + covse23 * (1 - se[1]) * (1 - se[4]) * se[5]
  + covse24 * (1 - se[1]) * (1 - se[3]) * se[5]
  - covse25 * (1 - se[1]) * (1 - se[3]) * (1 - se[4])
  + covse34 * (1 - se[1]) * (1 - se[2]) * se[5]
  - covse35 * (1 - se[1]) * (1 - se[2]) * (1 - se[4])
  - covse45 * (1 - se[1]) * (1 - se[2]) * (1 - se[3])
)

# Probability of observing Test1- Test2- Test3- Test4- Test5+ from a true
negative:
p0[17] <- (1 - prev) * (
  sp[1] * sp[2] * sp[3] * sp[4] * (1 - sp[5])
  + covsp12 * sp[3] * sp[4] * (1 - sp[5])
  + covsp13 * sp[2] * sp[4] * (1 - sp[5])
  + covsp14 * sp[2] * sp[3] * (1 - sp[5])
  - covsp15 * sp[2] * sp[3] * sp[4]
  + covsp23 * sp[1] * sp[4] * (1 - sp[5])
  + covsp24 * sp[1] * sp[3] * (1 - sp[5])
  - covsp25 * sp[1] * sp[3] * sp[4]
  + covsp34 * sp[1] * sp[2] * (1 - sp[5])
  - covsp35 * sp[1] * sp[2] * sp[4]
  - covsp45 * sp[1] * sp[2] * sp[3]
)
# Probability of observing Test1+ Test2- Test3- Test4- Test5+ from a true
positive:
p1[18] <- prev * (
  se[1] * (1 - se[2]) * (1 - se[3]) * (1 - se[4]) * se[5]
  - covse12 * (1 - se[3]) * (1 - se[4]) * se[5]
  - covse13 * (1 - se[2]) * (1 - se[4]) * se[5]
  - covse14 * (1 - se[2]) * (1 - se[3]) * se[5]
  + covse15 * (1 - se[2]) * (1 - se[3]) * (1 - se[4])
  + covse23 * se[1] * (1 - se[4]) * se[5]
```

```
  + covse24 * se[1] * (1 - se[3]) * se[5]
  - covse25 * se[1] * (1 - se[3]) * (1 - se[4])
  + covse34 * se[1] * (1 - se[2]) * se[5]
  - covse35 * se[1] * (1 - se[2]) * (1 - se[4])
  - covse45 * se[1] * (1 - se[2]) * (1 - se[3])
)

# Probability of observing Test1+ Test2- Test3- Test4- Test5+ from a true negative:
p0[18] <- (1 - prev) * (
  (1 - sp[1]) * sp[2] * sp[3] * sp[4] * (1 - sp[5])
  - covsp12 * sp[3] * sp[4] * (1 - sp[5])
  - covsp13 * sp[2] * sp[4] * (1 - sp[5])
  - covsp14 * sp[2] * sp[3] * (1 - sp[5])
  + covsp15 * sp[2] * sp[3] * sp[4]
  + covsp23 * (1 - sp[1]) * sp[4] * (1 - sp[5])
  + covsp24 * (1 - sp[1]) * sp[3] * (1 - sp[5])
  - covsp25 * (1 - sp[1]) * sp[3] * sp[4]
  + covsp34 * (1 - sp[1]) * sp[2] * (1 - sp[5])
  - covsp35 * (1 - sp[1]) * sp[2] * sp[4]
  - covsp45 * (1 - sp[1]) * sp[2] * sp[3]
)
# Probability of observing Test1- Test2+ Test3- Test4- Test5+ from a true positive:
p1[19] <- prev * (
  (1 - se[1]) * se[2] * (1 - se[3]) * (1 - se[4]) * se[5]
  - covse12 * (1 - se[3]) * (1 - se[4]) * se[5]
  + covse13 * se[2] * (1 - se[4]) * se[5]
  + covse14 * se[2] * (1 - se[3]) * se[5]
  - covse15 * se[2] * (1 - se[3]) * (1 - se[4])
  - covse23 * (1 - se[1]) * (1 - se[4]) * se[5]
  - covse24 * (1 - se[1]) * (1 - se[3]) * se[5]
  + covse25 * (1 - se[1]) * (1 - se[3]) * (1 - se[4])
  + covse34 * (1 - se[1]) * se[2] * se[5]
  - covse35 * (1 - se[1]) * se[2] * (1 - se[4])
  - covse45 * (1 - se[1]) * se[2] * (1 - se[3])
)

# Probability of observing Test1- Test2+ Test3- Test4- Test5+ from a true negative:
p0[19] <- (1 - prev) * (
  sp[1] * (1 - sp[2]) * sp[3] * sp[4] * (1 - sp[5])
  - covsp12 * sp[3] * sp[4] * (1 - sp[5])
  + covsp13 * (1 - sp[2]) * sp[4] * (1 - sp[5])
  + covsp14 * (1 - sp[2]) * sp[3] * (1 - sp[5])
  - covsp15 * (1 - sp[2]) * sp[3] * sp[4]
  - covsp23 * sp[1] * sp[4] * (1 - sp[5])
  - covsp24 * sp[1] * sp[3] * (1 - sp[5])
  + covsp25 * sp[1] * sp[3] * sp[4]
  + covsp34 * sp[1] * (1 - sp[2]) * (1 - sp[5])
  - covsp35 * sp[1] * (1 - sp[2]) * sp[4]
  - covsp45 * sp[1] * (1 - sp[2]) * sp[3]
)
# Probability of observing Test1+ Test2+ Test3- Test4- Test5+ from a true positive:
p1[20] <- prev * (
  se[1] * se[2] * (1 - se[3]) * (1 - se[4]) * se[5]
  + covse12 * (1 - se[3]) * (1 - se[4]) * se[5]
  - covse13 * se[2] * (1 - se[4]) * se[5]
  - covse14 * se[2] * (1 - se[3]) * se[5]
  + covse15 * se[2] * (1 - se[3]) * (1 - se[4])
```

```
  - covse23 * se[1] * (1 - se[4]) * se[5]
  - covse24 * se[1] * (1 - se[3]) * se[5]
  + covse25 * se[1] * (1 - se[3]) * (1 - se[4])
  + covse34 * se[1] * se[2] * se[5]
  - covse35 * se[1] * se[2] * (1 - se[4])
  - covse45 * se[1] * se[2] * (1 - se[3])
)

# Probability of observing Test1+ Test2+ Test3- Test4- Test5+ from a true
negative:
p0[20] <- (1 - prev) * (
  (1 - sp[1]) * (1 - sp[2]) * sp[3] * sp[4] * (1 - sp[5])
  + covsp12 * sp[3] * sp[4] * (1 - sp[5])
  - covsp13 * (1 - sp[2]) * sp[4] * (1 - sp[5])
  - covsp14 * (1 - sp[2]) * sp[3] * (1 - sp[5])
  + covsp15 * (1 - sp[2]) * sp[3] * sp[4]
  - covsp23 * (1 - sp[1]) * sp[4] * (1 - sp[5])
  - covsp24 * (1 - sp[1]) * sp[3] * (1 - sp[5])
  + covsp25 * (1 - sp[1]) * sp[3] * sp[4]
  + covsp34 * (1 - sp[1]) * (1 - sp[2]) * (1 - sp[5])
  - covsp35 * (1 - sp[1]) * (1 - sp[2]) * sp[4]
  - covsp45 * (1 - sp[1]) * (1 - sp[2]) * sp[3]
)
# Probability of observing Test1- Test2- Test3+ Test4- Test5+ from a true
positive:
p1[21] <- prev * (
  (1 - se[1]) * (1 - se[2]) * se[3] * (1 - se[4]) * se[5]
  + covse12 * se[3] * (1 - se[4]) * se[5]
  - covse13 * (1 - se[2]) * (1 - se[4]) * se[5]
  + covse14 * (1 - se[2]) * se[3] * se[5]
  - covse15 * (1 - se[2]) * se[3] * (1 - se[4])
  - covse23 * (1 - se[1]) * (1 - se[4]) * se[5]
  + covse24 * (1 - se[1]) * se[3] * se[5]
  - covse25 * (1 - se[1]) * se[3] * (1 - se[4])
  - covse34 * (1 - se[1]) * (1 - se[2]) * se[5]
  + covse35 * (1 - se[1]) * (1 - se[2]) * (1 - se[4])
  - covse45 * (1 - se[1]) * (1 - se[2]) * se[3]
)

# Probability of observing Test1- Test2- Test3+ Test4- Test5+ from a true
negative:
p0[21] <- (1 - prev) * (
  sp[1] * sp[2] * (1 - sp[3]) * sp[4] * (1 - sp[5])
  + covsp12 * (1 - sp[3]) * sp[4] * (1 - sp[5])
  - covsp13 * sp[2] * sp[4] * (1 - sp[5])
  + covsp14 * sp[2] * (1 - sp[3]) * (1 - sp[5])
  - covsp15 * sp[2] * (1 - sp[3]) * sp[4]
  - covsp23 * sp[1] * sp[4] * (1 - sp[5])
  + covsp24 * sp[1] * (1 - sp[3]) * (1 - sp[5])
  - covsp25 * sp[1] * (1 - sp[3]) * sp[4]
  - covsp34 * sp[1] * sp[2] * (1 - sp[5])
  + covsp35 * sp[1] * sp[2] * sp[4]
  - covsp45 * sp[1] * sp[2] * (1 - sp[3])
)
# Probability of observing Test1+ Test2- Test3+ Test4- Test5+ from a true
positive:
p1[22] <- prev * (
  se[1] * (1 - se[2]) * se[3] * (1 - se[4]) * se[5]
  - covse12 * se[3] * (1 - se[4]) * se[5]
  + covse13 * (1 - se[2]) * (1 - se[4]) * se[5]
  - covse14 * (1 - se[2]) * se[3] * se[5]
```

```
  + covse15 * (1 - se[2]) * se[3] * (1 - se[4])
  - covse23 * se[1] * (1 - se[4]) * se[5]
  + covse24 * se[1] * se[3] * se[5]
  - covse25 * se[1] * se[3] * (1 - se[4])
  - covse34 * se[1] * (1 - se[2]) * se[5]
  + covse35 * se[1] * (1 - se[2]) * (1 - se[4])
  - covse45 * se[1] * (1 - se[2]) * se[3]
)

# Probability of observing Test1+ Test2- Test3+ Test4- Test5+ from a true
negative:
p0[22] <- (1 - prev) * (
  (1 - sp[1]) * sp[2] * (1 - sp[3]) * sp[4] * (1 - sp[5])
  - covsp12 * (1 - sp[3]) * sp[4] * (1 - sp[5])
  + covsp13 * sp[2] * sp[4] * (1 - sp[5])
  - covsp14 * sp[2] * (1 - sp[3]) * (1 - sp[5])
  + covsp15 * sp[2] * (1 - sp[3]) * sp[4]
  - covsp23 * (1 - sp[1]) * sp[4] * (1 - sp[5])
  + covsp24 * (1 - sp[1]) * (1 - sp[3]) * (1 - sp[5])
  - covsp25 * (1 - sp[1]) * (1 - sp[3]) * sp[4]
  - covsp34 * (1 - sp[1]) * sp[2] * (1 - sp[5])
  + covsp35 * (1 - sp[1]) * sp[2] * sp[4]
  - covsp45 * (1 - sp[1]) * sp[2] * (1 - sp[3])
)
# Probability of observing Test1- Test2+ Test3+ Test4- Test5+ from a true
positive:
p1[23] <- prev * (
  (1 - se[1]) * se[2] * se[3] * (1 - se[4]) * se[5]
  - covse12 * se[3] * (1 - se[4]) * se[5]
  - covse13 * se[2] * (1 - se[4]) * se[5]
  + covse14 * se[2] * se[3] * se[5]
  - covse15 * se[2] * se[3] * (1 - se[4])
  + covse23 * (1 - se[1]) * (1 - se[4]) * se[5]
  - covse24 * (1 - se[1]) * se[3] * se[5]
  + covse25 * (1 - se[1]) * se[3] * (1 - se[4])
  - covse34 * (1 - se[1]) * se[2] * se[5]
  + covse35 * (1 - se[1]) * se[2] * (1 - se[4])
  - covse45 * (1 - se[1]) * se[2] * se[3]
)

# Probability of observing Test1- Test2+ Test3+ Test4- Test5+ from a true
negative:
p0[23] <- (1 - prev) * (
  sp[1] * (1 - sp[2]) * (1 - sp[3]) * sp[4] * (1 - sp[5])
  - covsp12 * (1 - sp[3]) * sp[4] * (1 - sp[5])
  - covsp13 * (1 - sp[2]) * sp[4] * (1 - sp[5])
  + covsp14 * (1 - sp[2]) * (1 - sp[3]) * (1 - sp[5])
  - covsp15 * (1 - sp[2]) * (1 - sp[3]) * sp[4]
  + covsp23 * sp[1] * sp[4] * (1 - sp[5])
  - covsp24 * sp[1] * (1 - sp[3]) * (1 - sp[5])
  + covsp25 * sp[1] * (1 - sp[3]) * sp[4]
  - covsp34 * sp[1] * (1 - sp[2]) * (1 - sp[5])
  + covsp35 * sp[1] * (1 - sp[2]) * sp[4]
  - covsp45 * sp[1] * (1 - sp[2]) * (1 - sp[3])
)
# Probability of observing Test1+ Test2+ Test3+ Test4- Test5+ from a true
positive:
p1[24] <- prev * (
  se[1] * se[2] * se[3] * (1 - se[4]) * se[5]
  + covse12 * se[3] * (1 - se[4]) * se[5]
  + covse13 * se[2] * (1 - se[4]) * se[5]
```

```
  - covse14 * se[2] * se[3] * se[5]
  + covse15 * se[2] * se[3] * (1 - se[4])
  + covse23 * se[1] * (1 - se[4]) * se[5]
  - covse24 * se[1] * se[3] * se[5]
  + covse25 * se[1] * se[3] * (1 - se[4])
  - covse34 * se[1] * se[2] * se[5]
  + covse35 * se[1] * se[2] * (1 - se[4])
  - covse45 * se[1] * se[2] * se[3]
)

# Probability of observing Test1+ Test2+ Test3+ Test4- Test5+ from a true
negative:
p0[24] <- (1 - prev) * (
  (1 - sp[1]) * (1 - sp[2]) * (1 - sp[3]) * sp[4] * (1 - sp[5])
  + covsp12 * (1 - sp[3]) * sp[4] * (1 - sp[5])
  + covsp13 * (1 - sp[2]) * sp[4] * (1 - sp[5])
  - covsp14 * (1 - sp[2]) * (1 - sp[3]) * (1 - sp[5])
  + covsp15 * (1 - sp[2]) * (1 - sp[3]) * sp[4]
  + covsp23 * (1 - sp[1]) * sp[4] * (1 - sp[5])
  - covsp24 * (1 - sp[1]) * (1 - sp[3]) * (1 - sp[5])
  + covsp25 * (1 - sp[1]) * (1 - sp[3]) * sp[4]
  - covsp34 * (1 - sp[1]) * (1 - sp[2]) * (1 - sp[5])
  + covsp35 * (1 - sp[1]) * (1 - sp[2]) * sp[4]
  - covsp45 * (1 - sp[1]) * (1 - sp[2]) * (1 - sp[3])
)
# Probability of observing Test1- Test2- Test3- Test4+ Test5+ from a true
positive:
p1[25] <- prev * (
  (1 - se[1]) * (1 - se[2]) * (1 - se[3]) * se[4] * se[5]
  + covse12 * (1 - se[3]) * se[4] * se[5]
  + covse13 * (1 - se[2]) * se[4] * se[5]
  - covse14 * (1 - se[2]) * (1 - se[3]) * se[5]
  - covse15 * (1 - se[2]) * (1 - se[3]) * se[4]
  + covse23 * (1 - se[1]) * se[4] * se[5]
  - covse24 * (1 - se[1]) * (1 - se[3]) * se[5]
  - covse25 * (1 - se[1]) * (1 - se[3]) * se[4]
  - covse34 * (1 - se[1]) * (1 - se[2]) * se[5]
  - covse35 * (1 - se[1]) * (1 - se[2]) * se[4]
  + covse45 * (1 - se[1]) * (1 - se[2]) * (1 - se[3])
)

# Probability of observing Test1- Test2- Test3- Test4+ Test5+ from a true
negative:
p0[25] <- (1 - prev) * (
  sp[1] * sp[2] * sp[3] * (1 - sp[4]) * (1 - sp[5])
  + covsp12 * sp[3] * (1 - sp[4]) * (1 - sp[5])
  + covsp13 * sp[2] * (1 - sp[4]) * (1 - sp[5])
  - covsp14 * sp[2] * sp[3] * (1 - sp[5])
  - covsp15 * sp[2] * sp[3] * (1 - sp[4])
  + covsp23 * sp[1] * (1 - sp[4]) * (1 - sp[5])
  - covsp24 * sp[1] * sp[3] * (1 - sp[5])
  - covsp25 * sp[1] * sp[3] * (1 - sp[4])
  - covsp34 * sp[1] * sp[2] * (1 - sp[5])
  - covsp35 * sp[1] * sp[2] * (1 - sp[4])
  + covsp45 * sp[1] * sp[2] * sp[3]
)
# Probability of observing Test1+ Test2- Test3- Test4+ Test5+ from a true
positive:
p1[26] <- prev * (
  se[1] * (1 - se[2]) * (1 - se[3]) * se[4] * se[5]
  - covse12 * (1 - se[3]) * se[4] * se[5]
```

```
  - covse13 * (1 - se[2]) * se[4] * se[5]
  + covse14 * (1 - se[2]) * (1 - se[3]) * se[5]
  + covse15 * (1 - se[2]) * (1 - se[3]) * se[4]
  + covse23 * se[1] * se[4] * se[5]
  - covse24 * se[1] * (1 - se[3]) * se[5]
  - covse25 * se[1] * (1 - se[3]) * se[4]
  - covse34 * se[1] * (1 - se[2]) * se[5]
  - covse35 * se[1] * (1 - se[2]) * se[4]
  + covse45 * se[1] * (1 - se[2]) * (1 - se[3])
)

# Probability of observing Test1+ Test2- Test3- Test4+ Test5+ from a true
negative:
p0[26] <- (1 - prev) * (
  (1 - sp[1]) * sp[2] * sp[3] * (1 - sp[4]) * (1 - sp[5])
  - covsp12 * sp[3] * (1 - sp[4]) * (1 - sp[5])
  - covsp13 * sp[2] * (1 - sp[4]) * (1 - sp[5])
  + covsp14 * sp[2] * sp[3] * (1 - sp[5])
  + covsp15 * sp[2] * sp[3] * (1 - sp[4])
  + covsp23 * (1 - sp[1]) * (1 - sp[4]) * (1 - sp[5])
  - covsp24 * (1 - sp[1]) * sp[3] * (1 - sp[5])
  - covsp25 * (1 - sp[1]) * sp[3] * (1 - sp[4])
  - covsp34 * (1 - sp[1]) * sp[2] * (1 - sp[5])
  - covsp35 * (1 - sp[1]) * sp[2] * (1 - sp[4])
  + covsp45 * (1 - sp[1]) * sp[2] * sp[3]
)
# Probability of observing Test1- Test2+ Test3- Test4+ Test5+ from a true
positive:
p1[27] <- prev * (
  (1 - se[1]) * se[2] * (1 - se[3]) * se[4] * se[5]
  - covse12 * (1 - se[3]) * se[4] * se[5]
  + covse13 * se[2] * se[4] * se[5]
  - covse14 * se[2] * (1 - se[3]) * se[5]
  - covse15 * se[2] * (1 - se[3]) * se[4]
  - covse23 * (1 - se[1]) * se[4] * se[5]
  + covse24 * (1 - se[1]) * (1 - se[3]) * se[5]
  + covse25 * (1 - se[1]) * (1 - se[3]) * se[4]
  - covse34 * (1 - se[1]) * se[2] * se[5]
  - covse35 * (1 - se[1]) * se[2] * se[4]
  + covse45 * (1 - se[1]) * se[2] * (1 - se[3])
)

# Probability of observing Test1- Test2+ Test3- Test4+ Test5+ from a true
negative:
p0[27] <- (1 - prev) * (
  sp[1] * (1 - sp[2]) * sp[3] * (1 - sp[4]) * (1 - sp[5])
  - covsp12 * sp[3] * (1 - sp[4]) * (1 - sp[5])
  + covsp13 * (1 - sp[2]) * (1 - sp[4]) * (1 - sp[5])
  - covsp14 * (1 - sp[2]) * sp[3] * (1 - sp[5])
  - covsp15 * (1 - sp[2]) * sp[3] * (1 - sp[4])
  - covsp23 * sp[1] * (1 - sp[4]) * (1 - sp[5])
  + covsp24 * sp[1] * sp[3] * (1 - sp[5])
  + covsp25 * sp[1] * sp[3] * (1 - sp[4])
  - covsp34 * sp[1] * (1 - sp[2]) * (1 - sp[5])
  - covsp35 * sp[1] * (1 - sp[2]) * (1 - sp[4])
  + covsp45 * sp[1] * (1 - sp[2]) * sp[3]
)
# Probability of observing Test1+ Test2+ Test3- Test4+ Test5+ from a true
positive:
p1[28] <- prev * (
  se[1] * se[2] * (1 - se[3]) * se[4] * se[5]
```

```
  + covse12 * (1 - se[3]) * se[4] * se[5]
  - covse13 * se[2] * se[4] * se[5]
  + covse14 * se[2] * (1 - se[3]) * se[5]
  + covse15 * se[2] * (1 - se[3]) * se[4]
  - covse23 * se[1] * se[4] * se[5]
  + covse24 * se[1] * (1 - se[3]) * se[5]
  + covse25 * se[1] * (1 - se[3]) * se[4]
  - covse34 * se[1] * se[2] * se[5]
  - covse35 * se[1] * se[2] * se[4]
  + covse45 * se[1] * se[2] * (1 - se[3])
)

# Probability of observing Test1+ Test2+ Test3- Test4+ Test5+ from a true
negative:
p0[28] <- (1 - prev) * (
  (1 - sp[1]) * (1 - sp[2]) * sp[3] * (1 - sp[4]) * (1 - sp[5])
  + covsp12 * sp[3] * (1 - sp[4]) * (1 - sp[5])
  - covsp13 * (1 - sp[2]) * (1 - sp[4]) * (1 - sp[5])
  + covsp14 * (1 - sp[2]) * sp[3] * (1 - sp[5])
  + covsp15 * (1 - sp[2]) * sp[3] * (1 - sp[4])
  - covsp23 * (1 - sp[1]) * (1 - sp[4]) * (1 - sp[5])
  + covsp24 * (1 - sp[1]) * sp[3] * (1 - sp[5])
  + covsp25 * (1 - sp[1]) * sp[3] * (1 - sp[4])
  - covsp34 * (1 - sp[1]) * (1 - sp[2]) * (1 - sp[5])
  - covsp35 * (1 - sp[1]) * (1 - sp[2]) * (1 - sp[4])
  + covsp45 * (1 - sp[1]) * (1 - sp[2]) * sp[3]
)
# Probability of observing Test1- Test2- Test3+ Test4+ Test5+ from a true
positive:
p1[29] <- prev * (
  (1 - se[1]) * (1 - se[2]) * se[3] * se[4] * se[5]
  + covse12 * se[3] * se[4] * se[5]
  - covse13 * (1 - se[2]) * se[4] * se[5]
  - covse14 * (1 - se[2]) * se[3] * se[5]
  - covse15 * (1 - se[2]) * se[3] * se[4]
  - covse23 * (1 - se[1]) * se[4] * se[5]
  - covse24 * (1 - se[1]) * se[3] * se[5]
  - covse25 * (1 - se[1]) * se[3] * se[4]
  + covse34 * (1 - se[1]) * (1 - se[2]) * se[5]
  + covse35 * (1 - se[1]) * (1 - se[2]) * se[4]
  + covse45 * (1 - se[1]) * (1 - se[2]) * se[3]
)

# Probability of observing Test1- Test2- Test3+ Test4+ Test5+ from a true
negative:
p0[29] <- (1 - prev) * (
  sp[1] * sp[2] * (1 - sp[3]) * (1 - sp[4]) * (1 - sp[5])
  + covsp12 * (1 - sp[3]) * (1 - sp[4]) * (1 - sp[5])
  - covsp13 * sp[2] * (1 - sp[4]) * (1 - sp[5])
  - covsp14 * sp[2] * (1 - sp[3]) * (1 - sp[5])
  - covsp15 * sp[2] * (1 - sp[3]) * (1 - sp[4])
  - covsp23 * sp[1] * (1 - sp[4]) * (1 - sp[5])
  - covsp24 * sp[1] * (1 - sp[3]) * (1 - sp[5])
  - covsp25 * sp[1] * (1 - sp[3]) * (1 - sp[4])
  + covsp34 * sp[1] * sp[2] * (1 - sp[5])
  + covsp35 * sp[1] * sp[2] * (1 - sp[4])
  + covsp45 * sp[1] * sp[2] * (1 - sp[3])
)
# Probability of observing Test1+ Test2- Test3+ Test4+ Test5+ from a true
positive:
p1[30] <- prev * (
```

```
  se[1] * (1 - se[2]) * se[3] * se[4] * se[5]
  - covse12 * se[3] * se[4] * se[5]
  + covse13 * (1 - se[2]) * se[4] * se[5]
  + covse14 * (1 - se[2]) * se[3] * se[5]
  + covse15 * (1 - se[2]) * se[3] * se[4]
  - covse23 * se[1] * se[4] * se[5]
  - covse24 * se[1] * se[3] * se[5]
  - covse25 * se[1] * se[3] * se[4]
  + covse34 * se[1] * (1 - se[2]) * se[5]
  + covse35 * se[1] * (1 - se[2]) * se[4]
  + covse45 * se[1] * (1 - se[2]) * se[3]
)

# Probability of observing Test1+ Test2- Test3+ Test4+ Test5+ from a true
negative:
p0[30] <- (1 - prev) * (
  (1 - sp[1]) * sp[2] * (1 - sp[3]) * (1 - sp[4]) * (1 - sp[5])
  - covsp12 * (1 - sp[3]) * (1 - sp[4]) * (1 - sp[5])
  + covsp13 * sp[2] * (1 - sp[4]) * (1 - sp[5])
  + covsp14 * sp[2] * (1 - sp[3]) * (1 - sp[5])
  + covsp15 * sp[2] * (1 - sp[3]) * (1 - sp[4])
  - covsp23 * (1 - sp[1]) * (1 - sp[4]) * (1 - sp[5])
  - covsp24 * (1 - sp[1]) * (1 - sp[3]) * (1 - sp[5])
  - covsp25 * (1 - sp[1]) * (1 - sp[3]) * (1 - sp[4])
  + covsp34 * (1 - sp[1]) * sp[2] * (1 - sp[5])
  + covsp35 * (1 - sp[1]) * sp[2] * (1 - sp[4])
  + covsp45 * (1 - sp[1]) * sp[2] * (1 - sp[3])
)
# Probability of observing Test1- Test2+ Test3+ Test4+ Test5+ from a true
positive:
p1[31] <- prev * (
  (1 - se[1]) * se[2] * se[3] * se[4] * se[5]
  - covse12 * se[3] * se[4] * se[5]
  - covse13 * se[2] * se[4] * se[5]
  - covse14 * se[2] * se[3] * se[5]
  - covse15 * se[2] * se[3] * se[4]
  + covse23 * (1 - se[1]) * se[4] * se[5]
  + covse24 * (1 - se[1]) * se[3] * se[5]
  + covse25 * (1 - se[1]) * se[3] * se[4]
  + covse34 * (1 - se[1]) * se[2] * se[5]
  + covse35 * (1 - se[1]) * se[2] * se[4]
  + covse45 * (1 - se[1]) * se[2] * se[3]
)

# Probability of observing Test1- Test2+ Test3+ Test4+ Test5+ from a true
negative:
p0[31] <- (1 - prev) * (
  sp[1] * (1 - sp[2]) * (1 - sp[3]) * (1 - sp[4]) * (1 - sp[5])
  - covsp12 * (1 - sp[3]) * (1 - sp[4]) * (1 - sp[5])
  - covsp13 * (1 - sp[2]) * (1 - sp[4]) * (1 - sp[5])
  - covsp14 * (1 - sp[2]) * (1 - sp[3]) * (1 - sp[5])
  - covsp15 * (1 - sp[2]) * (1 - sp[3]) * (1 - sp[4])
  + covsp23 * sp[1] * (1 - sp[4]) * (1 - sp[5])
  + covsp24 * sp[1] * (1 - sp[3]) * (1 - sp[5])
  + covsp25 * sp[1] * (1 - sp[3]) * (1 - sp[4])
  + covsp34 * sp[1] * (1 - sp[2]) * (1 - sp[5])
  + covsp35 * sp[1] * (1 - sp[2]) * (1 - sp[4])
  + covsp45 * sp[1] * (1 - sp[2]) * (1 - sp[3])
)
# Probability of observing Test1+ Test2+ Test3+ Test4+ Test5+ from a true
positive:
```

```
p1[32] <- prev * (
  se[1] * se[2] * se[3] * se[4] * se[5]
  + covse12 * se[3] * se[4] * se[5]
  + covse13 * se[2] * se[4] * se[5]
  + covse14 * se[2] * se[3] * se[5]
  + covse15 * se[2] * se[3] * se[4]
  + covse23 * se[1] * se[4] * se[5]
  + covse24 * se[1] * se[3] * se[5]
  + covse25 * se[1] * se[3] * se[4]
  + covse34 * se[1] * se[2] * se[5]
  + covse35 * se[1] * se[2] * se[4]
  + covse45 * se[1] * se[2] * se[3]
)

# Probability of observing Test1+ Test2+ Test3+ Test4+ Test5+ from a true
negative:
p0[32] <- (1 - prev) * (
  (1 - sp[1]) * (1 - sp[2]) * (1 - sp[3]) * (1 - sp[4]) * (1 - sp[5])
  + covsp12 * (1 - sp[3]) * (1 - sp[4]) * (1 - sp[5])
  + covsp13 * (1 - sp[2]) * (1 - sp[4]) * (1 - sp[5])
  + covsp14 * (1 - sp[2]) * (1 - sp[3]) * (1 - sp[5])
  + covsp15 * (1 - sp[2]) * (1 - sp[3]) * (1 - sp[4])
  + covsp23 * (1 - sp[1]) * (1 - sp[4]) * (1 - sp[5])
  + covsp24 * (1 - sp[1]) * (1 - sp[3]) * (1 - sp[5])
  + covsp25 * (1 - sp[1]) * (1 - sp[3]) * (1 - sp[4])
  + covsp34 * (1 - sp[1]) * (1 - sp[2]) * (1 - sp[5])
  + covsp35 * (1 - sp[1]) * (1 - sp[2]) * (1 - sp[4])
  + covsp45 * (1 - sp[1]) * (1 - sp[2]) * (1 - sp[3])
)

prob <- p0 + p1

# ---- Simulate Frequencies from Multinomial ----
freq <- rmultinom(nsim, nobs, prob)  # Simulated contingency counts

# ---- Construct Full Data Array ----
Y <- array(NA, dim = c(nsim, 2^ntests, ntests + 1))
for (i in 1:nsim) {
  Y[i,, 1:ntests] <- as.matrix(expand.grid(rep(list(c(0, 1)), ntests)))  #
Pattern matrix
  Y[i,, ntests + 1] <- freq[, i]                                          #
Frequencies
}

# ---- Parameters to Monitor from JAGS ----
mymonitoredparamslist <- c("prev", "sens", "spec", "sumrd")

# ---- Storage for Posterior Summaries ----
prevmedian <- rep(NA, nsim)
prevlower  <- rep(NA, nsim)
prevupper  <- rep(NA, nsim)

semedian <- matrix(NA, nrow = ntests, ncol = nsim)
selower  <- matrix(NA, nrow = ntests, ncol = nsim)
seupper  <- matrix(NA, nrow = ntests, ncol = nsim)

spmedian <- matrix(NA, nrow = ntests, ncol = nsim)
splower  <- matrix(NA, nrow = ntests, ncol = nsim)
spupper  <- matrix(NA, nrow = ntests, ncol = nsim)

ResD <- rep(NA, nsim)  # Residual Deviance
```

```
DIC  <- rep(NA, nsim)  # Deviance Information Criterion

# ---- Run Simulation Loop ----
for (j in 1:nsim) {
  mydatalist <- list(r = Y[j,, ntests + 1],
                     y = Y[j,, 1:ntests],
                     nobs = nobs,
                     AcceptTest = rep(1, ntests))

  fit <- jags(data = mydatalist,
              parameters.to.save = mymonitoredparamslist,
              model.file = "keddie_jjhc_ll_ncs_di.txt",
              n.chains = n.chains,
              n.iter = n.iter,
              n.burnin = n.burnin,
              n.thin = n.thin,
              progress.bar = "text")

  # Convergence check: Rhat > 1.1 or effective size < 400
  if (any(fit$BUGSoutput$summary[2:13, 8] > 1.1) |
any(fit$BUGSoutput$summary[2:13, 9] < 400)) {
    prevmedian[j] <- prevlower[j] <- prevupper[j] <- ResD[j] <- DIC[j] <-
NA
    semedian[, j] <- selower[, j] <- seupper[, j] <- NA
    spmedian[, j] <- splower[, j] <- spupper[, j] <- NA
  } else {
    prevmedian[j] <- fit$BUGSoutput$median$prev
    prevlower[j]  <- fit$BUGSoutput$summary[2, 3]
    prevupper[j]  <- fit$BUGSoutput$summary[2, 7]

    semedian[, j] <- fit$BUGSoutput$median$sens
    selower[, j]  <- fit$BUGSoutput$summary[3:7, 3]
    seupper[, j]  <- fit$BUGSoutput$summary[3:7, 7]

    spmedian[, j] <- fit$BUGSoutput$median$spec
    splower[, j]  <- fit$BUGSoutput$summary[8:12, 3]
    spupper[, j]  <- fit$BUGSoutput$summary[8:12, 7]

    ResD[j] <- fit$BUGSoutput$mean$sumrd
    DIC[j]  <- ResD[j] + fit$BUGSoutput$pD
  }
}

rm(fit)  # Free memory

# ---- Save Results ----
save.image(file = file.path(folder, "KEDDIE_JJHC_LL_2000.RData"))

# ---- Execution Time ----
print(Sys.time() - start)
```

JAGS script to fit the L-L model with correct interactions

```
model {

  for (i in 1:32) {

    r[i] ~ dpois(mu[i])                        # Poisson likelihood for
observed counts
    mu[i] <- p[i] * nobs                       # Expected counts
```

```
    p[i] <- (1 - prev) * p1[i] + prev * p2[i]  # Mixture probability

    p1[i] <- y1[i] / sum(y1[])                 # Pr(pattern i | no disease)
    p2[i] <- y2[i] / sum(y2[])                 # Pr(pattern i | disease)

    # Log-linear model for the non-diseased group
    log(y1[i]) <- lambda_coef[1,1]*y[i,1] + lambda_coef[1,2]*y[i,2] +
                  lambda_coef[1,3]*y[i,3] + lambda_coef[1,4]*y[i,4] +
                  lambda_coef[1,5]*y[i,5] +
                  lambda_coef[1,6]*(y[i,1]*y[i,2] + y[i,1]*y[i,3] +
y[i,1]*y[i,4] +
                                    y[i,2]*y[i,3] + y[i,2]*y[i,4] +
y[i,3]*y[i,4])

    # Log-linear model for the diseased group
    log(y2[i]) <- lambda_coef[2,1]*y[i,1] + lambda_coef[2,2]*y[i,2] +
                  lambda_coef[2,3]*y[i,3] + lambda_coef[2,4]*y[i,4] +
                  lambda_coef[2,5]*y[i,5] +
                  lambda_coef[2,6]*(y[i,1]*y[i,2] + y[i,1]*y[i,3] +
y[i,1]*y[i,4] +
                                    y[i,2]*y[i,3] + y[i,2]*y[i,4] +
y[i,3]*y[i,4])

    # Residual deviance
    rd[i] <- 2 * ((mu[i] - r[i]) + r[i] * log(r[i] / mu[i]))
  }

  sumrd <- sum(rd[])  # Total residual deviance

  # Prior for prevalence
  prev ~ dbeta(1, 1)

  for (t in 1:5) {
    # Sensitivity and specificity calculations
    sens[t] <- inprod(y[1:32, t], y2[1:32]) / sum(y2[1:32])
    fpr[t]  <- inprod(y[1:32, t], y1[1:32]) / sum(y1[1:32])
    spec[t] <- 1 - fpr[t]
    youden[t] <- sens[t] + spec[t] - 1

    AcceptTest[t] ~ dbern(ifelse(youden[t] >= 0, 1, 0))  # Test acceptance
indicator

    for (d in 1:2) {
      q[d, t] ~ dbeta(1, 1)                         # Prior on marginal
probabilities
      lambda_coef[d, t] <- logit(q[d, t])           # Main effects
    }
  }

  # Priors for shared interaction term
  lambda_coef[1,6] ~ dnorm(0, 1)
  lambda_coef[2,6] ~ dnorm(0, 1)

}
```

L-L DGM

R script to simulate data and fit L-L models

```
## This R script simulates data from L-L DGM based on the CDPN data
## with the parameter settings specified in Appendix B and fits the
corresponding
## log-linear model with correct interactions.

rm(list = ls())
require(R2jags)

start <- Sys.time()

# Create output folder if it doesn't exist
folder <- "Chapter5-KEDDIE_LL_LL"
if (!file.exists(folder)) dir.create(folder)

set.seed(567)

# Simulation settings
nobs <- 2000              # Number of observations per dataset
ntests <- 5               # Number of tests
nsim <- 1250              # Number of simulated datasets
n.burnin <- 10000         # MCMC burn-in
n.iter <- 50000           # Total MCMC iterations
n.thin <- 1               # Thinning rate
n.chains <- 3             # Number of chains

# Initialise objects
log_prob1 <- rep(NA, 2^ntests)
log_prob0 <- rep(NA, 2^ntests)
p1 <- rep(NA, 2^ntests)
p0 <- rep(NA, 2^ntests)
se <- rep(NA, ntests)
sp <- rep(NA, ntests)

mu <- matrix(NA, nrow = 2, ncol = ntests)  # Main effect coefficients

# Prevalence and pairwise dependency terms
prev <- 0.5
lambda_coef <- list()
lambda_coef[[1]] <- rep(0.9, 6)   # For non-diseased
lambda_coef[[2]] <- rep(0.9, 6)   # For diseased

# Main effects: [non-diseased, diseased]
mu[1, ] <- c(-2.595, -2.595, -2.595, -2.595, -4.600)
mu[2, ] <- c(-1.035, -1.035, -1.035, -1.035,  0.620)

# Generate full pattern matrix (32 rows for 5 binary tests)
pattern <- as.matrix(expand.grid(c(0,1), c(0,1), c(0,1), c(0,1), c(0,1)))

# Compute log-probabilities for all response patterns
for (i in 1:2^ntests) {
  log_prob0[i] <- mu[1,1]*pattern[i,1] + mu[1,2]*pattern[i,2] +
mu[1,3]*pattern[i,3] +
                  mu[1,4]*pattern[i,4] + mu[1,5]*pattern[i,5] +
                  lambda_coef[[1]][1]*pattern[i,1]*pattern[i,2] +
```

```
                     lambda_coef[[1]][2]*pattern[i,1]*pattern[i,3] +
                     lambda_coef[[1]][3]*pattern[i,1]*pattern[i,4] +
                     lambda_coef[[1]][4]*pattern[i,2]*pattern[i,3] +
                     lambda_coef[[1]][5]*pattern[i,2]*pattern[i,4] +
                     lambda_coef[[1]][6]*pattern[i,3]*pattern[i,4]

  log_prob1[i] <- mu[2,1]*pattern[i,1] + mu[2,2]*pattern[i,2] +
mu[2,3]*pattern[i,3] +
                     mu[2,4]*pattern[i,4] + mu[2,5]*pattern[i,5] +
                     lambda_coef[[2]][1]*pattern[i,1]*pattern[i,2] +
                     lambda_coef[[2]][2]*pattern[i,1]*pattern[i,3] +
                     lambda_coef[[2]][3]*pattern[i,1]*pattern[i,4] +
                     lambda_coef[[2]][4]*pattern[i,2]*pattern[i,3] +
                     lambda_coef[[2]][5]*pattern[i,2]*pattern[i,4] +
                     lambda_coef[[2]][6]*pattern[i,3]*pattern[i,4]
}

# Convert log-probabilities to probabilities and normalise
for (i in 1:2^ntests) {
  p0[i] <- (1 - prev) * (exp(log_prob0[i]) / sum(exp(log_prob0)))
  p1[i] <- prev * (exp(log_prob1[i]) / sum(exp(log_prob1)))
}
prob <- p0 + p1

# Compute true test characteristics
for (i in 1:ntests) {
  se[i] <- sum(p1[pattern[, i] == 1]) / sum(p1)
  sp[i] <- sum(p0[pattern[, i] == 0]) / sum(p0)
}

# Generate multinomial counts from simulated probabilities
freq <- rmultinom(nsim, nobs, prob)

# Store full simulated data
Y <- array(NA, dim = c(nsim, 2^ntests, ntests + 1))
for (i in 1:nsim) {
  Y[i, , 1:ntests] <- pattern
  Y[i, , ntests + 1] <- freq[, i]
}

# Define parameters to monitor from JAGS model
mymonitoredparamslist <- c("prev", "sens", "spec", "sumrd")

# Initialise result storage
prevmedian <- rep(NA, nsim)
prevlower <- rep(NA, nsim)
prevupper <- rep(NA, nsim)
semedian <- matrix(NA, nrow = ntests, ncol = nsim)
selower  <- matrix(NA, nrow = ntests, ncol = nsim)
seupper  <- matrix(NA, nrow = ntests, ncol = nsim)
spmedian <- matrix(NA, nrow = ntests, ncol = nsim)
splower  <- matrix(NA, nrow = ntests, ncol = nsim)
spupper  <- matrix(NA, nrow = ntests, ncol = nsim)
ResD <- rep(NA, nsim)
DIC  <- rep(NA, nsim)

# Run model for each simulated dataset
for (j in 1:nsim) {
  mydatalist <- list(
    r = Y[j, , ntests + 1],
    y = Y[j, , 1:ntests],
```

```
    nobs = nobs,
    AcceptTest = rep(1, ntests)
  )

  fit <- jags(
    data = mydatalist,
    parameters.to.save = mymonitoredparamslist,
    model.file = "keddie_ll_ll.txt",
    n.chains = n.chains,
    n.iter = n.iter,
    n.burnin = n.burnin,
    n.thin = n.thin,
    progress.bar = "text"
  )

  # Convergence check
  if (any(fit$BUGSoutput$summary[2:13, 8] > 1.1) |
      any(fit$BUGSoutput$summary[2:13, 9] < 400)) {
    prevmedian[j] <- prevlower[j] <- prevupper[j] <- ResD[j] <- DIC[j] <-
NA
    semedian[, j] <- selower[, j] <- seupper[, j] <- spmedian[, j] <-
splower[, j] <- spupper[, j] <- NA
  } else {
    prevmedian[j] <- fit$BUGSoutput$median$prev
    prevlower[j]  <- fit$BUGSoutput$summary[2, 3]
    prevupper[j]  <- fit$BUGSoutput$summary[2, 7]

    semedian[, j] <- fit$BUGSoutput$median$sens
    selower[, j]  <- fit$BUGSoutput$summary[3:7, 3]
    seupper[, j]  <- fit$BUGSoutput$summary[3:7, 7]

    spmedian[, j] <- fit$BUGSoutput$median$spec
    splower[, j]  <- fit$BUGSoutput$summary[8:12, 3]
    spupper[, j]  <- fit$BUGSoutput$summary[8:12, 7]

    ResD[j] <- fit$BUGSoutput$mean$sumrd
    DIC[j]  <- ResD[j] + fit$BUGSoutput$pD
  }
}

rm(fit)

save.image(file = file.path(folder, "KEDDIE_LL_LL_2000.RData"))
print(Sys.time() - start)
```

JAGS script to fit the L-L model with correct interactions

The same L-L model assuming one shared pairwise interaction for Tests 1–4 within each disease state – originally fitted to data sets simulated from the latent trait DGM for the CDPN data – was used.

JAGS script to fit the L-L model with all interactions (in both groups) and hyperlasso priors

```
model {

  for (i in 1:32) {
```

```
  # Likelihood for observed counts
  r[i] ~ dpois(mu[i])
  mu[i] <- p[i] * nobs

  # Mixture model for disease status
  p[i] <- (1 - prev) * p1[i] + prev * p2[i]

  # Probabilities for non-diseased and diseased groups
  p1[i] <- y1[i] / sum(y1[])
  p2[i] <- y2[i] / sum(y2[])

  # Log-linear model for non-diseased group
  log(y1[i]) <- lambda_coef[1,1]*y[i,1] + lambda_coef[1,2]*y[i,2] +
                lambda_coef[1,3]*y[i,3] + lambda_coef[1,4]*y[i,4] +
                lambda_coef[1,5]*y[i,5] +
                lambda_int[1]*y[i,1]*y[i,2] +
                lambda_int[2]*y[i,1]*y[i,3] +
                lambda_int[3]*y[i,1]*y[i,4] +
                lambda_int[4]*y[i,1]*y[i,5] +
                lambda_int[5]*y[i,2]*y[i,3] +
                lambda_int[6]*y[i,2]*y[i,4] +
                lambda_int[7]*y[i,2]*y[i,5] +
                lambda_int[8]*y[i,3]*y[i,4] +
                lambda_int[9]*y[i,3]*y[i,5] +
                lambda_int[10]*y[i,4]*y[i,5]

  # Log-linear model for diseased group
  log(y2[i]) <- lambda_coef[2,1]*y[i,1] + lambda_coef[2,2]*y[i,2] +
                lambda_coef[2,3]*y[i,3] + lambda_coef[2,4]*y[i,4] +
                lambda_coef[2,5]*y[i,5] +
                lambda_int[11]*y[i,1]*y[i,2] +
                lambda_int[12]*y[i,1]*y[i,3] +
                lambda_int[13]*y[i,1]*y[i,4] +
                lambda_int[14]*y[i,1]*y[i,5] +
                lambda_int[15]*y[i,2]*y[i,3] +
                lambda_int[16]*y[i,2]*y[i,4] +
                lambda_int[17]*y[i,2]*y[i,5] +
                lambda_int[18]*y[i,3]*y[i,4] +
                lambda_int[19]*y[i,3]*y[i,5] +
                lambda_int[20]*y[i,4]*y[i,5]

  # Deviance residual
  rd[i] <- 2 * ((mu[i] - r[i]) + r[i] * log(r[i] / mu[i]))
}

sumrd <- sum(rd[])

# Prior for disease prevalence
prev ~ dbeta(1, 1)

# Diagnostic accuracy parameters
for (t in 1:5) {
  sens[t] <- inprod(y[1:32, t], y2[1:32]) / sum(y2[1:32])
  fpr[t]  <- inprod(y[1:32, t], y1[1:32]) / sum(y1[1:32])
  spec[t] <- 1 - fpr[t]
  youden[t] <- sens[t] + spec[t] - 1

  AcceptTest[t] ~ dbern(ifelse(youden[t] >= 0, 1, 0))

  for (d in 1:2) {
    q[d, t] ~ dbeta(1, 1)
```

```
      lambda_coef[d, t] <- logit(q[d, t])    # Main effects via logit(q)
    }
  }

  # Hyper-LASSO priors for interaction terms
  for (t in 1:20) {
    lambda_int[t] ~ ddexp(0, prec_lambda[t])          # Double-exponential
prior
    prec_lambda[t] <- 1 / sqrt(2 * tau_jl[t])         # Precision = 1 /
sqrt(2 * tau)
    tau_jl[t] ~ dgamma(0.5, inv_theta2)               # Local shrinkage
scale
  }

  # Global shrinkage parameter
  theta ~ dt(0, 1, 1) T(0,)                           # Half-Cauchy(0,1)
  inv_theta2 <- 1 / (theta^2)

}
```

JAGS script to fit the L-L model with all interactions (in both groups) and elastic net priors

```
model {

  for (i in 1:32) {

    # Likelihood for observed counts
    r[i] ~ dpois(mu[i])
    mu[i] <- p[i] * nobs

    # Mixture of non-diseased and diseased probabilities
    p[i] <- (1 - prev) * p1[i] + prev * p2[i]

    # Probability of pattern i given no disease / disease
    p1[i] <- y1[i] / sum(y1[])
    p2[i] <- y2[i] / sum(y2[])

    # Log-linear model for non-diseased population
    log(y1[i]) <- lambda_coef[1,1]*y[i,1] + lambda_coef[1,2]*y[i,2] +
                  lambda_coef[1,3]*y[i,3] + lambda_coef[1,4]*y[i,4] +
                  lambda_coef[1,5]*y[i,5] +
                  lambda_int[1]*y[i,1]*y[i,2] +
                  lambda_int[2]*y[i,1]*y[i,3] +
                  lambda_int[3]*y[i,1]*y[i,4] +
                  lambda_int[4]*y[i,1]*y[i,5] +
                  lambda_int[5]*y[i,2]*y[i,3] +
                  lambda_int[6]*y[i,2]*y[i,4] +
                  lambda_int[7]*y[i,2]*y[i,5] +
                  lambda_int[8]*y[i,3]*y[i,4] +
                  lambda_int[9]*y[i,3]*y[i,5] +
                  lambda_int[10]*y[i,4]*y[i,5]

    # Log-linear model for diseased population
    log(y2[i]) <- lambda_coef[2,1]*y[i,1] + lambda_coef[2,2]*y[i,2] +
                  lambda_coef[2,3]*y[i,3] + lambda_coef[2,4]*y[i,4] +
                  lambda_coef[2,5]*y[i,5] +
                  lambda_int[11]*y[i,1]*y[i,2] +
                  lambda_int[12]*y[i,1]*y[i,3] +
                  lambda_int[13]*y[i,1]*y[i,4] +
                  lambda_int[14]*y[i,1]*y[i,5] +
```

```
                  lambda_int[15]*y[i,2]*y[i,3] +
                  lambda_int[16]*y[i,2]*y[i,4] +
                  lambda_int[17]*y[i,2]*y[i,5] +
                  lambda_int[18]*y[i,3]*y[i,4] +
                  lambda_int[19]*y[i,3]*y[i,5] +
                  lambda_int[20]*y[i,4]*y[i,5]

    # Deviance residual
    rd[i] <- 2 * ((mu[i] - r[i]) + r[i] * log(r[i] / mu[i]))
  }

  sumrd <- sum(rd[])

  # Prior for disease prevalence
  prev ~ dbeta(1, 1)

  # Main effects for diagnostic tests
  for (t in 1:5) {
    sens[t] <- inprod(y[1:32, t], y2[1:32]) / sum(y2[1:32])
    fpr[t]  <- inprod(y[1:32, t], y1[1:32]) / sum(y1[1:32])
    spec[t] <- 1 - fpr[t]
    youden[t] <- sens[t] + spec[t] - 1

    AcceptTest[t] ~ dbern(ifelse(youden[t] >= 0, 1, 0))

    for (d in 1:2) {
      q[d, t] ~ dbeta(1, 1)
      lambda_coef[d, t] <- logit(q[d, t])
    }
  }

  # Global elastic net parameters
  lambda1 ~ dt(0, 1, 1) T(0,)
  lambda2 ~ dt(0, 1, 1) T(0,)

  upper <- 8 * lambda2 / (lambda1^2 + 1e-8)

  # Shrinkage priors for interaction terms
  for (t in 1:20) {
    tau_jl[t] ~ dgamma(0.5, upper) T(1,)
    inv_var_lambda[t] <- lambda2 * tau_jl[t] / (tau_jl[t] - 1 + 1e-8)
    lambda_int[t] ~ dnorm(0, inv_var_lambda[t])
  }

}
```

JAGS script to fit the L-L model with all interactions (in both groups) and regularized horseshoe priors

```
model {

  for (i in 1:32) {

    # Poisson likelihood for counts in the contingency table
    r[i] ~ dpois(mu[i])
    mu[i] <- p[i] * nobs

    # Mixture model for disease status
    p[i] <- (1 - prev) * p1[i] + prev * p2[i]
```

```
  # Probabilities conditional on disease status
  p1[i] <- y1[i] / sum(y1[])
  p2[i] <- y2[i] / sum(y2[])

  # Log-linear model for non-diseased population
  log(y1[i]) <- lambda_coef[1,1]*y[i,1] + lambda_coef[1,2]*y[i,2] +
                lambda_coef[1,3]*y[i,3] + lambda_coef[1,4]*y[i,4] +
                lambda_coef[1,5]*y[i,5] +
                lambda_int[1]*y[i,1]*y[i,2] +
                lambda_int[2]*y[i,1]*y[i,3] +
                lambda_int[3]*y[i,1]*y[i,4] +
                lambda_int[4]*y[i,1]*y[i,5] +
                lambda_int[5]*y[i,2]*y[i,3] +
                lambda_int[6]*y[i,2]*y[i,4] +
                lambda_int[7]*y[i,2]*y[i,5] +
                lambda_int[8]*y[i,3]*y[i,4] +
                lambda_int[9]*y[i,3]*y[i,5] +
                lambda_int[10]*y[i,4]*y[i,5]

  # Log-linear model for diseased population
  log(y2[i]) <- lambda_coef[2,1]*y[i,1] + lambda_coef[2,2]*y[i,2] +
                lambda_coef[2,3]*y[i,3] + lambda_coef[2,4]*y[i,4] +
                lambda_coef[2,5]*y[i,5] +
                lambda_int[11]*y[i,1]*y[i,2] +
                lambda_int[12]*y[i,1]*y[i,3] +
                lambda_int[13]*y[i,1]*y[i,4] +
                lambda_int[14]*y[i,1]*y[i,5] +
                lambda_int[15]*y[i,2]*y[i,3] +
                lambda_int[16]*y[i,2]*y[i,4] +
                lambda_int[17]*y[i,2]*y[i,5] +
                lambda_int[18]*y[i,3]*y[i,4] +
                lambda_int[19]*y[i,3]*y[i,5] +
                lambda_int[20]*y[i,4]*y[i,5]

  # Deviance residuals
  rd[i] <- 2 * ((mu[i] - r[i]) + r[i] * log(r[i] / mu[i]))
}

sumrd <- sum(rd[])

# Prior for disease prevalence
prev ~ dbeta(1, 1)

# Main effects: logit-transformed from beta priors
for (t in 1:5) {
  sens[t] <- inprod(y[1:32, t], y2[1:32]) / sum(y2[1:32])
  fpr[t]  <- inprod(y[1:32, t], y1[1:32]) / sum(y1[1:32])
  spec[t] <- 1 - fpr[t]
  youden[t] <- sens[t] + spec[t] - 1
  AcceptTest[t] ~ dbern(ifelse(youden[t] >= 0, 1, 0))

  for (d in 1:2) {
    q[d, t] ~ dbeta(1, 1)
    lambda_coef[d, t] <- logit(q[d, t])
  }
}

# Regularized horseshoe priors for interaction terms
for (t in 1:20) {
  lambda_int[t] ~ dnorm(0, prec_lambda[t])
```

```
    prec_lambda[t] <- 1 / (lambda * sigma2[t])
    sigma2[t] <- c2 * pow(delta[t], 2) / (c2 + pow(lambda, 2) *
pow(delta[t], 2))
    delta[t] ~ dt(0, 1, 1) T(0,)    # Half-Cauchy
  }

  lambda ~ dt(0, 1, 1) T(0,)        # Global shrinkage
  c2 <- 1 / invc2
  invc2 ~ dgamma(2, 8)              # Prior for inverse scale squared
}
```
